# Multi-stromal organoid co-culture modelling reveals epithelial–fibroblast heterogeneity in pancreatic cancer and pancreatitis

**DOI:** 10.1038/s41556-026-02057-w

**Published:** 2026-09-02

**Authors:** Wenlong Li, Muntadher Jihad, Eloise G. Lloyd, Marta Zaccaria, Gianluca Mucciolo, Gideon Nsubuga, Alwin Koonan-Lonappan, Joaquín Araos Henríquez, Priscilla S. W. Cheng, Sneha Harish, Sally Mills, Paul M. Johnson, Weike Luo, Alejandro Alonso Montero, Rebecca Brais, Alex Deamer, Ania M. Piskorz, Julia Jones, Jodi L. Miller, Bristi Basu, Paul D. W. Kirk, Mireia Vallespinos, Giulia Biffi

**Affiliations:** 1 University of Cambridge, Cancer Research UK Cambridge Institute, Li Ka Shing Centre, Cambridge, UK; 2 Cambridge University Hospitals NHS Foundation Trust, Addenbrooke’s Hospital, Cambridge, UK; 3 University of Cambridge, Department of Oncology, Cambridge, UK; 4 MRC Biostatistics Unit, Cambridge Institute of Public Health, University of Cambridge, Cambridge, UK

**Keywords:** Pancreatic cancer, Cancer microenvironment, Cancer models

## Abstract

Interactions between epithelial cells and fibroblasts influence disease progression and treatment response in pancreatic ductal adenocarcinoma (PDAC). While the diversity of fibroblasts in PDAC is increasingly recognized, it remains unclear how these cells differ from fibroblasts found in pancreatic inflammation. Chronic pancreatitis is a stroma-rich inflammatory disease and a risk factor for PDAC, making it a useful setting to study how epithelial cells and fibroblasts change during disease. Here we compare fibroblast diversity and epithelial–stromal interactions in pancreatitis and PDAC using human samples, mouse models and mouse pancreatitis-derived epithelial organoids. We also developed pancreatitis and PDAC organoid co-cultures containing pancreatic stellate cells, fibroblasts and mesothelial cells. Combining in vitro and in vivo models better reflected human disease than mouse models alone. Overall, our findings reveal distinct epithelial and fibroblast features in pancreatitis and PDAC and provide models to identify disease-specific markers and therapeutic vulnerabilities.

## Abstract (web summary)

Li, Jihad and colleagues develop an organoid co-culture system, alongside patient tissue and mouse model analyses, to model, characterize and compare cellular interactions between epithelia and stroma in pancreatitis versus pancreatic ductal adenocarcinoma.

## Main

The high mortality rate of pancreatic ductal adenocarcinoma (PDAC) is largely attributed to late diagnosis and treatment resistance, with contributions from an extensive non-cancerous stroma^1^. Within this stroma, cancer-associated fibroblasts (CAFs) are key determinants of PDAC progression and therapy response^2,3^. PDAC CAFs are phenotypically and functionally heterogeneous, typically classified as inflammatory CAFs (iCAFs), myofibroblastic CAFs (myCAFs) and antigen-presenting CAFs (apCAFs), with additional populations described within and across these states^4–8^. Whether fibroblasts in PDAC resemble those in non-malignant pancreatic diseases is less clear. Comparing CAFs in PDAC with fibroblasts in pancreatic conditions that predispose to this malignancy could identify cancer-specific fibroblast states relevant to interceptive, diagnostic and therapeutic PDAC strategies.

In humans, chronic pancreatitis is a persistent inflammatory state and a risk factor for PDAC^9^, characterized by increased fibrosis and extracellular matrix (ECM) deposition that contribute to disease progression^10^. The ECM of chronic pancreatitis shares features with that in PDAC in mouse models and human tissues^11^, potentially because activated, ECM-producing pancreatic stellate cells (PSCs) are, at least in part, precursors of PDAC CAFs and contributors to pancreatitis fibrosis^12–14^.

Mouse models have advanced our understanding of epithelial cell reprogramming during pancreatic injury and inflammation, and its contribution to malignant transformation^15–18^. However, fibroblast reprogramming during pancreatitis, and how fibroblast and epithelial cell–fibroblast crosstalk differs between pancreatitis and PDAC, remain underexplored despite evidence that pancreatitis fibroblasts are heterogeneous^14,19^. This is partly owing to the lack of pancreatitis models that enable dissection of epithelial cell–fibroblast crosstalk. The most commonly used mouse model involves caerulein-induced pancreatic injury, which promotes digestive enzyme release from acinar cells, acinar-to-ductal metaplasia (ADM) and fibrosis^20^. Additional pancreatitis models may enable orthogonal validation and capture a broader range of human pancreatitis- and PDAC-specific features.

Here, we aimed to identify epithelial and fibroblast features characteristic of pancreatic inflammation or malignancy by analysing mouse and human pancreatitis and PDAC tissues, alongside pancreatitis- and PDAC-derived organoid/multi-stroma co-culture models.

## Results

### Myofibroblasts are enriched in human and mouse PDAC relative to pancreatitis

**Fig. 1:**
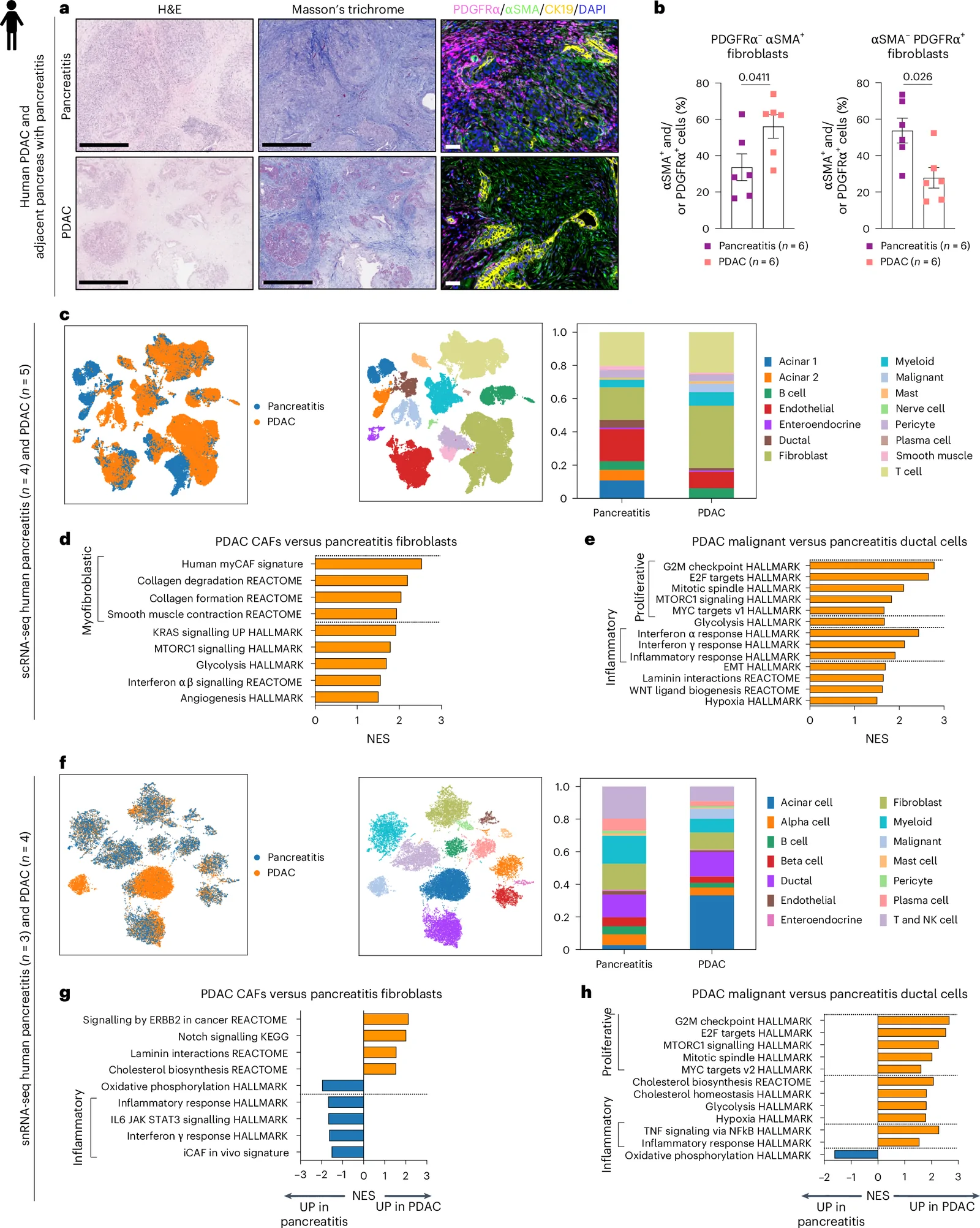
Myofibroblasts are more abundant in human PDAC relative to pancreatitis. **a**, Representative H&E and Masson’s trichrome (for collagen deposition) stains, as well as multiplex IF images of CK19 (yellow), αSMA (green), PDGFRα (pink) and DAPI (nuclear stain, blue) in human PDAC and adjacent pancreas tissue with chronic pancreatitis. Scale bars, 500 μm (H&E and Masson’s trichrome) or 50 μm (multiplex IF). **b**, The quantification of PDGFRα^−^αSMA^+^ (left) and αSMA^−^PDGFRα^+^ (right) CK19^−^DAPI^+^ fibroblasts relative to αSMA^+^ and/or PDGFRα^+^ CK19^−^DAPI^+^ fibroblasts in human PDAC (*n* = 6) and matched adjacent pancreas with chronic pancreatitis (*n* = 6) tissues, as assessed by multiplex IF analysis. Results show mean ± s.e.m. and *P* values from Mann–Whitney tests. **c**, Uniform manifold approximation and projection (UMAP) plots of cell types in the combined scRNA-seq dataset from human PDAC (*n* = 5) and chronic pancreatitis (*n* = 4) tissues. Different conditions (left) or cell types (middle) are colour coded. scRNA-seq data are from Dimitrieva et al.^19^. Right: cell-type contribution in human PDAC (*n* = 5) and pancreatitis (*n* = 4), represented as a bar plot showing proportions of the different cell type clusters in each condition. **d**, The significantly upregulated pathways identified by GSEA of human PDAC CAFs compared with pancreatitis fibroblasts, as assessed by MAST from the scRNA-seq dataset. The human myCAF signature is from Elyada et al.^5^. **e**, Significantly upregulated pathways identified by GSEA of human PDAC malignant cells compared with pancreatitis ductal cells, as assessed by MAST from the scRNA-seq dataset, and genes were ranked based on log_2_ fold change. **f**, UMAP plots of cell types in the combined snRNA-seq dataset from human PDAC (*n* = 4) and chronic pancreatitis (*n* = 3) tissues. Different conditions (left) and cell types (middle) are colour coded. snRNA-seq data are from Tosti et al. and Chen et al.^21,22^. Right: cell type contribution in human PDAC (*n* = 4) and pancreatitis (*n* = 3), represented as a bar plot showing proportions of the different cell clusters in each condition. **g**, The significantly upregulated and downregulated pathways identified by GSEA of human PDAC CAFs compared with pancreatitis fibroblasts, as assessed by MAST from the snRNA-seq dataset. The in vivo iCAF signature (orthologues) is from Elyada et al. (mouse scRNA-seq dataset)^5^. **h**, The significantly upregulated and downregulated pathways identified by GSEA of human PDAC malignant cells compared with pancreatitis ductal cells, as assessed by MAST from the snRNA-seq dataset. NES, normalized enrichment score. Source data

**Extended Data Fig. 1:**
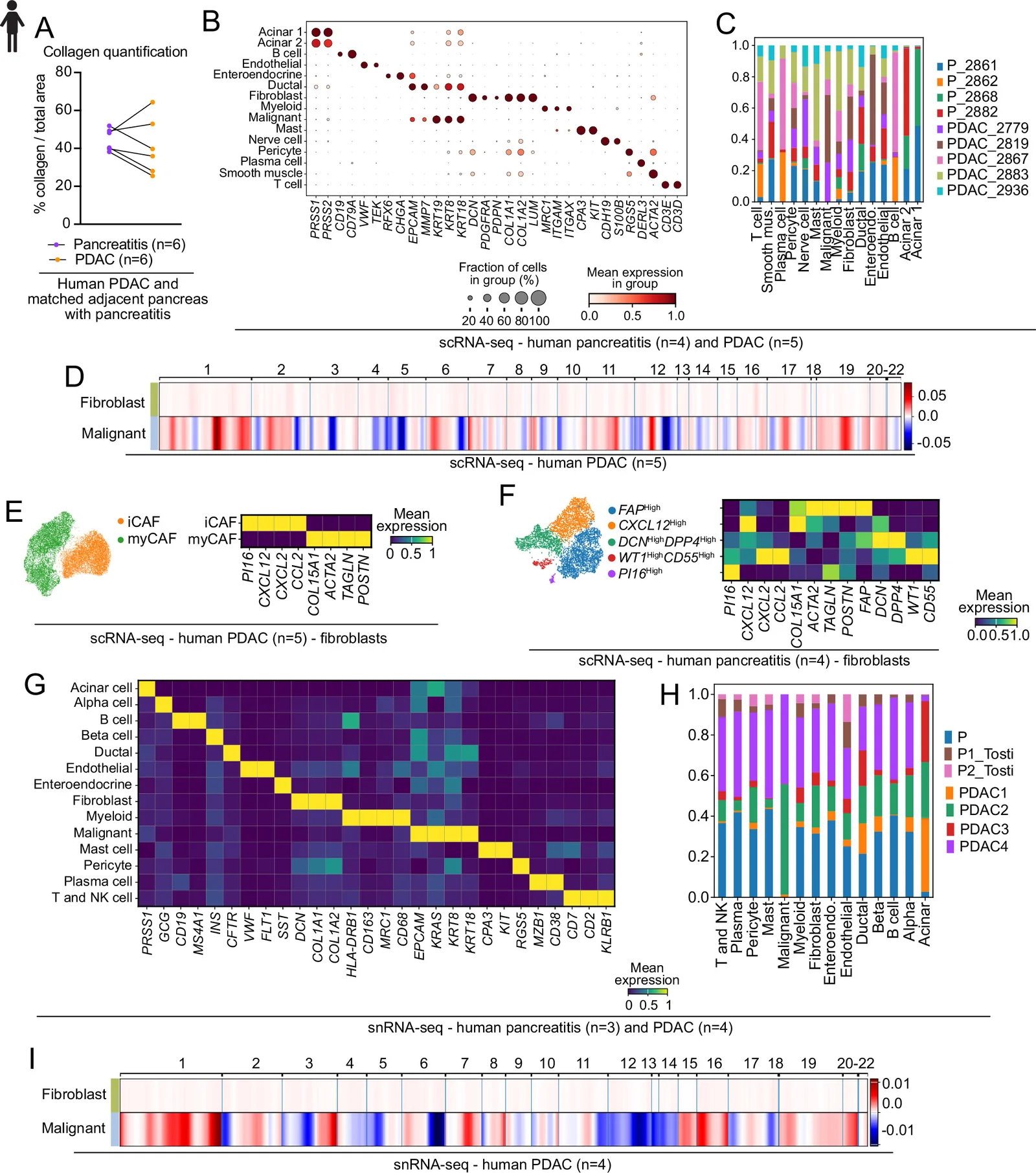
Myofibroblasts are more abundant in human PDAC relative to pancreatitis. **(A)** Quantification of Masson’s trichrome stain for collagen deposition in human PDAC (n = 6) and matched adjacent pancreas with chronic pancreatitis (n = 6) tissues. Results show values of matched tissue pairs, connected by a line. **(B)** Dot plot of scaled expression of cell type-specific markers in each cell cluster of human chronic pancreatitis (n = 4) and pancreatic ductal adenocarcinoma (PDAC, n = 5) tissues, as analysed by single-cell RNA-sequencing (scRNA-seq). The colour intensity represents the expression level, and the size of the dots represents the percentage of expressing cells. scRNA-seq data are from Dimitrieva et al. **(C)** Sample contribution to different cell types in human pancreatitis and PDAC analysed by scRNA-seq, represented as bar plot showing proportions of the different samples in each cell cluster. **(D)** Heatmap showing large-scale copy number variation (CNV) profile of the fibroblast and malignant cell clusters identified by scRNA-seq of human PDAC. The colour coding represents the CNV level based on a sliding window of 250 genes. Amplifications are shown in red, and deletions are shown in blue. The fibroblast cluster was used as reference cell cluster. **(E)** Left: Uniform Manifold Approximation and Projection (UMAP) plot of fibroblasts from human PDAC (n = 5). Different subsets are colour coded. Right: Heatmap of scaled expression of CAF subtype-specific markers in each fibroblast cluster of human PDAC. Data are scaled such that the cluster with the lowest average expression = 0 and the highest = 1 for each gene. **(F)** Left: UMAP plot of fibroblasts from human chronic pancreatitis (n = 4). Different subsets are colour coded. Right: Heatmap of scaled expression of fibroblast subtype-specific markers in each fibroblast cluster of human chronic pancreatitis. Data are scaled such that the cluster with the lowest average expression = 0 and the highest = 1 for each gene. **(G)** Heatmap of scaled expression of cell type-specific markers in each cell cluster of human chronic pancreatitis (n = 3) and PDAC (n = 4) tissues, as analysed by single-nuclei RNA-sequencing (snRNA-seq). Data are scaled such that the cluster with the lowest average expression = 0 and the highest = 1 for each gene. snRNA-seq data are from Tosti et al. and Chen et al. **(H)** Sample contribution to different cell types in human pancreatitis and PDAC analysed by snRNA-seq, represented as bar plot showing proportions of the different samples in each cell cluster. **(I)** Heatmap showing large-scale CNV profile of the fibroblast and malignant cell clusters identified by snRNA-seq of human PDAC. The colour coding represents the CNV level based on a sliding window of 250 genes. Amplifications are shown in red, and deletions are shown in blue. The fibroblast cluster was used as reference cell cluster. Source numerical data are available.

We first investigated whether fibroblast composition differs between PDAC and associated chronic pancreatitis in patient samples (Supplementary Table 1). Histological analyses of human PDAC tissues and matched adjacent pancreata with chronic pancreatitis revealed modest differences in overall collagen deposition, but distinct fibroblast populations, with higher expression of myofibroblast marker alpha smooth muscle actin (αSMA) in PDAC CAFs and higher levels of fibroblast marker platelet-derived growth factor receptor alpha (PDGFRα) in pancreatitis fibroblasts (Fig. 1a,b and Extended Data Fig. 1a). To assess whether epithelial cells and fibroblasts show disease-specific features in PDAC compared with pancreatitis from non-PDAC patients, we analysed a published single-cell RNA-sequencing (scRNA-seq) dataset of human PDAC (*n* = 5) and chronic pancreatitis^19^ (*n* = 4) (Fig. 1c, Extended Data Fig. 1b–d and Supplementary Table 2). myCAF and iCAF populations were identified in PDAC, whereas five fibroblast subsets were detected in pancreatitis: *WT1*^High^*CD55*^High^, *PI16*^High^, *FAP*^High^, *DCN*^High^*DPP4*^High^ and *CXCL12*^High^ (Extended Data Fig. 1e,f). Myofibroblastic signatures (for example, smooth muscle contraction and collagen formation) were upregulated in PDAC CAFs compared with pancreatitis fibroblasts, while proliferation-associated (for example, G2M checkpoint) and inflammatory (for example, inflammatory response) signatures were upregulated in PDAC malignant cells compared with pancreatitis ductal cells (Fig. 1d,e). To validate these findings, we integrated two published single-nuclei RNA-sequencing (snRNA-seq) datasets^21,22^ to generate a combined dataset of human PDAC (*n* = 4) and chronic pancreatitis (*n* = 3) (Fig. 1f, Extended Data Fig. 1g–i and Supplementary Table 3). This confirmed the upregulation of proliferation-associated and inflammatory pathways in PDAC malignant cells compared with pancreatitis ductal cells and revealed increased inflammatory pathways (for example, interferon gamma response) in pancreatitis fibroblasts relative to PDAC CAFs (Fig. 1g,h). Together, these findings indicate that epithelial and fibroblast transcriptomes differ markedly between human pancreatitis and PDAC, suggesting a more myofibroblastic phenotype in PDAC CAFs and a more inflammatory phenotype in pancreatitis fibroblasts.

**Fig. 2:**
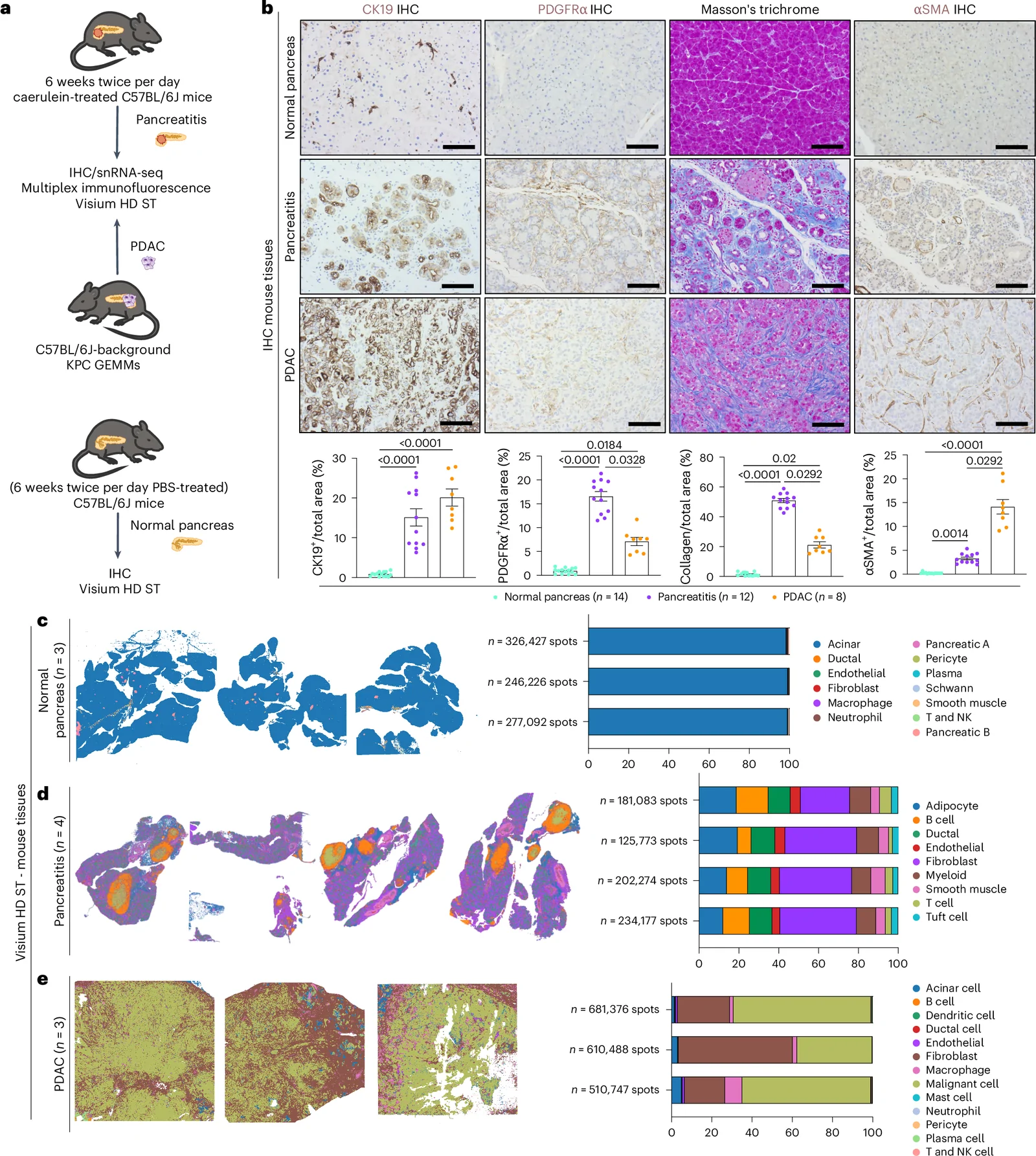
Myofibroblasts are more abundant in mouse PDAC relative to pancreatitis. **a**, A schematic of models and techniques used for analysis of normal pancreas (that is, from untreated or 6-week phosphate-buffered saline (PBS)-treated C57BL/6J mice), pancreatitis (that is, from 6-week caerulein-treated C57BL/6J mice) and PDAC (that is, from KPC GEMMs) tissues. **b**, Representative images and quantifications of the ductal marker cytokeratin 19 (CK19), the fibroblast marker PDGF receptor alpha (PDGFRα), Masson’s trichrome for collagen deposition and the myofibroblast marker alpha smooth muscle actin (αSMA) immunohistochemical stains of mouse normal pancreas (*n* = 14), pancreatitis (*n* = 12) and PDAC (*n* = 8) tissues. Pancreatitis samples derive from three independent treatment cohorts. Results show mean ± s.e.m. Plots show adjusted *P* values from Kruskal–Wallis tests. Scale bars, 50 μm. **c**–**e**, Visium HD spatial transcriptomic (ST) slide images (left) and corresponding deconvoluted cell type contribution represented as bar plots showing the proportions of the different cell clusters (right) in mouse normal pancreas (**c**; *n* = 3), pancreatitis (**d**; *n* = 4) and PDAC (**e**; *n* = 3) tissues. IHC, immunohistochemistry; ST, spatial transcriptomics. Source data

**Extended Data Fig. 2:**
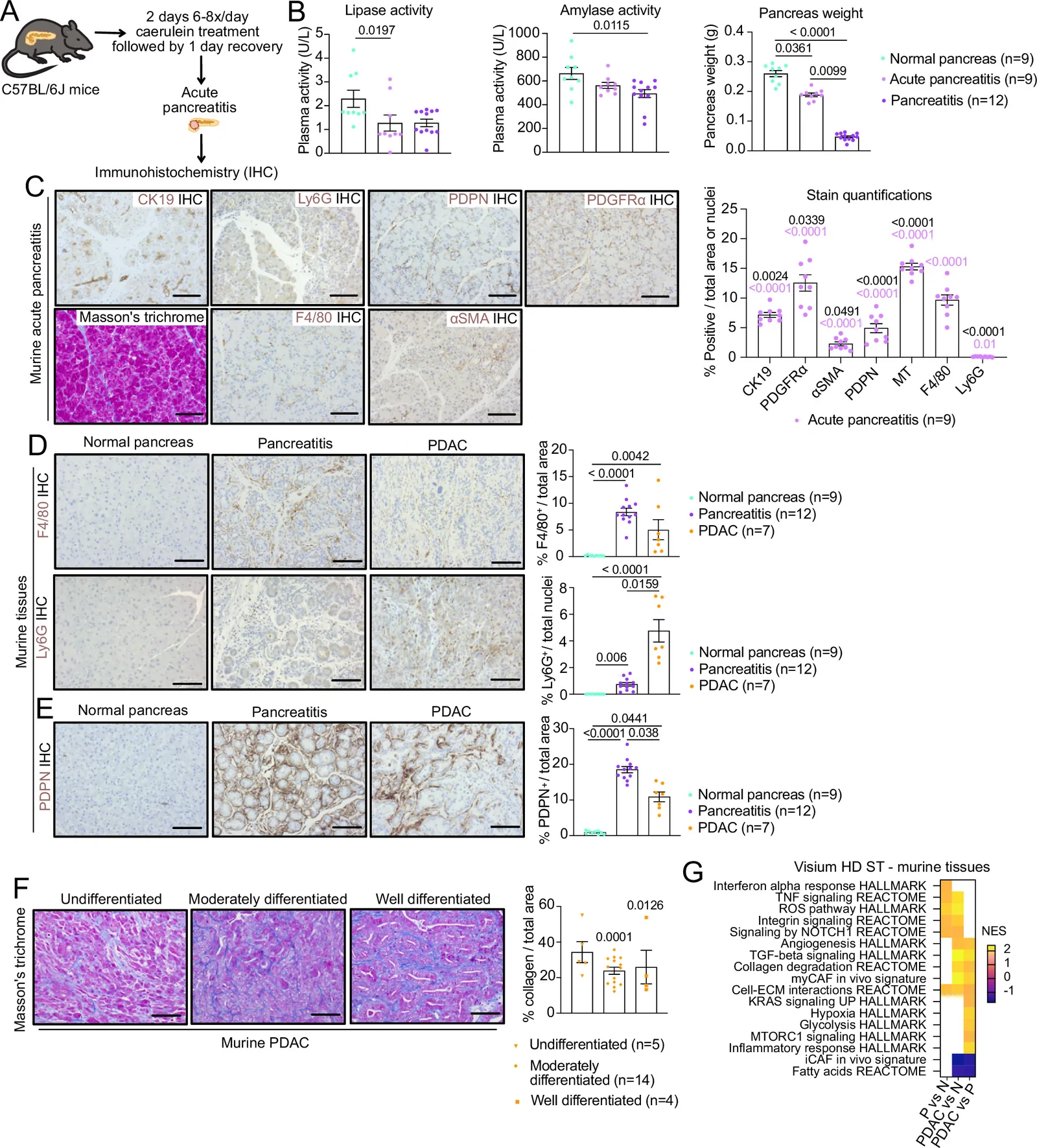
Myofibroblasts are more abundant in murine PDAC relative to pancreatitis. **(A)** Schematic of model and techniques used for the analysis of murine acute pancreatitis tissues. **(B)** Lipase (left) and amylase (middle) activity levels in plasma from normal pancreas (n = 9), acute pancreatitis (n = 9) and pancreatitis (n = 12) mouse models. Right: weights of pancreata from the same mouse models. Results show mean ± SEM. *P adj*, Kruskal-Wallis test. Pancreatitis and acute pancreatitis samples derive from two independent treatment cohorts. (**C**) Representative images and quantifications of the ductal marker keratin 19 (CK19), the fibroblast marker platelet-derived growth factor receptor alpha (PDGFRα), the myofibroblast marker alpha smooth muscle actin (αSMA), the fibroblast marker podoplanin (PDPN), the macrophage marker F4/80, the neutrophil marker Ly6G and Masson’s trichrome (for collagen deposition) stains of murine acute pancreatitis tissues (n = 9). Results show mean ± SEM. *P* (pink), Mann-Whitney test, indicate significant upregulation compared to normal pancreas tissues from Fig. 2 and Extended Data Fig. 2. *P* (black), Mann-Whitney test, indicate significant downregulation compared to pancreatitis tissues from Fig. 2 and Extended Data Fig. 2. Scale bars, 50 μm. Acute pancreatitis samples derive from two independent treatment cohorts. **(D-E)** Representative images and quantifications of F4/80 and Ly6G **(D)** and PDPN **(E)** stains of murine normal pancreas (n = 9), pancreatitis (n = 12) and PDAC (n = 7) tissues. Results show mean ± SEM. *P adj*, Kruskal-Wallis test. Scale bars, 50 μm. Pancreatitis samples derive from two independent treatment cohorts. (**F**) Representative images and quantifications of Masson’s trichrome stains of undifferentiated (n = 5), moderately differentiated (n = 14) and well differentiated (n = 4) areas from murine PDAC tissues (n = 17). *P adj*, Kruskal-Wallis test compared to murine pancreatitis tissues from Fig. 2. Scale bars, 50 μm. (**G**) Significantly upregulated and downregulated pathways identified by Gene Set Enrichment Analysis (GSEA) of pancreatitis relative to normal fibroblasts (left), PDAC CAFs compared to normal fibroblasts (middle) and PDAC CAFs compared to pancreatitis fibroblasts (right), as assessed by pseudobulk analysis of Visium HD spatial transcriptomics (ST). The *in vivo* myCAF and iCAF signatures are from Elyada et al. (murine scRNA-seq dataset). Blank spaces indicate when gene signatures were not significantly enriched. NES, normalized enrichment score. Source numerical data are available.

Given the limited availability of human chronic pancreatitis samples and datasets, we established mouse models of pancreatitis and compared them with mouse normal pancreas and PDAC tissues to improve understanding of epithelial and fibroblast differences between pancreatitis and PDAC (Fig. 2a and Extended Data Fig. 2a). PDACs were obtained from C57BL/6J-background *Kras*^LSL-G12D/+^; *Trp53*^LSL-R172H/+^; *Pdx1*-Cre (KPC) genetically engineered mouse models (GEMMs)^23^, while pancreatitis tissues were obtained from C57BL/6J mice treated with the cholecystokinin analogue caerulein^20^. We modelled pancreatitis and acute pancreatitis by treating mice with caerulein for 6 weeks or 2 days, respectively. Normal pancreata were obtained from C57BL/6J mice that were either untreated or injected with phosphate-buffered saline (vehicle) for 6 weeks. As expected, caerulein treatment reduced plasma amylase and lipase activity levels and pancreas weights in acute pancreatitis and pancreatitis mice compared with normal mice, consistent with acinar damage and pancreatic atrophy^24^ (Extended Data Fig. 2b). Pancreas weights were reduced the most in pancreatitis mice.

Pancreatitis is associated with loss of acinar cells through ADM, increased immune cell infiltration and fibrosis^25^. We therefore used immunohistochemistry to compare these features among pancreatitis, acute pancreatitis and normal pancreas tissues. Expression of the ductal marker cytokeratin 19 (CK19) was significantly increased in pancreatitis and acute pancreatitis compared with normal pancreas tissues, indicating elevated ADM (Fig. 2b and Extended Data Fig. 2c). Pancreatitis and acute pancreatitis also showed significantly increased immune cell infiltration compared with normal pancreas tissues, as indicated by increased expression of F4/80 and Ly6G, which mark macrophages and neutrophils, respectively (Extended Data Fig. 2d). Finally, fibrosis was significantly elevated in pancreatitis and acute pancreatitis compared with normal pancreas tissues, as detected using fibroblast markers PDGFRα, podoplanin (PDPN) and αSMA, and Masson’s trichrome stain for collagen (Fig. 2b and Extended Data Fig. 2c,e). Pancreatitis tissues also had higher levels of all these fibrosis markers than acute pancreatitis tissues (Extended Data Fig. 2c). These data are consistent with evidence that prolonged inflammatory regimens in mice better recapitulate features of the chronic inflammatory state associated with increased PDAC risk in patients^20^. Thus, we focused on pancreatitis (rather than acute pancreatitis) mouse models to further investigate fibroblast and epithelial cell differences between pancreatic inflammation and malignancy.

To evaluate stromal composition in mouse pancreatitis and PDAC, we analysed these tissues by immunohistochemistry. Levels of ADM and macrophage infiltration were similar between conditions, whereas neutrophil infiltration was significantly greater in PDAC compared with pancreatitis (Fig. 2b and Extended Data Fig. 2d). Fibroblast composition also differed: PDGFRα, PDPN and collagen were significantly higher in pancreatitis compared with PDAC tissues, whereas myofibroblast abundance (αSMA) was higher in PDAC, consistent with increased myofibroblastic signatures in human PDAC CAFs relative to pancreatitis fibroblasts (Fig. 2b, Extended Data Fig. 2e,f and Supplementary Table 4).

To further characterize normal pancreas, pancreatitis and PDAC tissues, we performed Visium HD spatial transcriptomics on a subset of samples analysed by immunohistochemistry (Supplementary Table 5). Normal pancreas contained few fibroblasts, ductal cells and immune cells, with acinar cells comprising the dominant population (Fig. 2c). By contrast, fibroblasts were the most abundant cell type in mouse pancreatitis, whereas malignant cells and fibroblasts dominated in PDAC (Fig. 2d,e). Pseudobulk analysis of fibroblasts revealed distinct transcriptional reprogramming in pancreatitis and PDAC relative to normal fibroblasts, with myofibroblastic signatures preferentially upregulated in PDAC CAFs (Extended Data Fig. 2g).

Together, these data suggest that epithelial and stromal cells differ between PDAC and pancreatitis tissues, with myofibroblasts being more abundant in mouse and human PDAC.

### Mouse models recapitulate epithelial and fibroblast features of human pancreatitis and PDAC

**Fig. 3:**
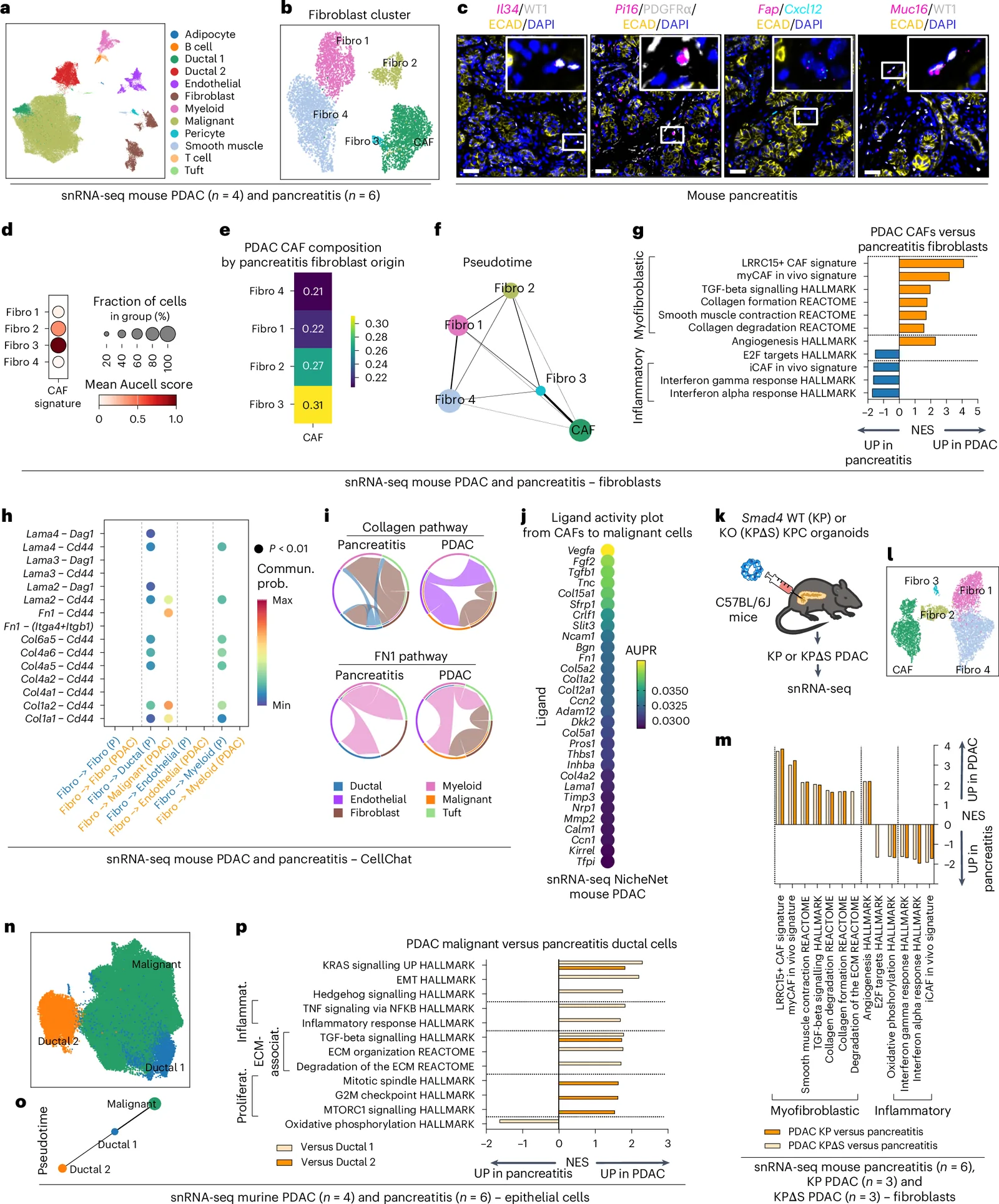
Pancreatitis and PDAC mouse models recapitulate epithelial and fibroblast features of the human disease. **a**, A UMAP plot of cell types identified by snRNA-seq in mouse PDAC (*n* = 4) and pancreatitis (*n* = 6). Different cell types are colour coded. Pancreatitis samples derive from one treatment cohort. **b**, A UMAP plot of fibroblasts in the combined snRNA-seq dataset from mouse PDAC and pancreatitis. Different subsets are colour coded. **c**, Representative multiplex RNA ISH and IF images of *Il34* (pink) and WT1 (white) to identify Fibro 1, *Pi16* (pink) and PDGFRα (white) to identify Fibro 2, *Cxcl12* (cyan) and *Fap* (pink) to identify Fibro 3 or *Muc16* (pink) and WT1 (white) to identify Fibro 4 in mouse pancreatitis tissues. DAPI (blue) and E-cadherin (ECAD; yellow). Scale bars, 20 μm. The data shown are representative of two experimental replicates (each with the same eight tissues). **d**, A dot plot of scaled AUCell score enrichment of the PDAC CAF signature in each pancreatitis fibroblast cluster. The PDAC CAF signature was obtained by comparing PDAC CAFs with pancreatitis fibroblasts from the snRNA-seq dataset. **e**, A heat map showing the fractional contribution of in vivo pancreatitis fibroblast subclusters to in vivo PDAC CAFs derived from averaged scANVI soft label probabilities. **f**, PAGA illustrates the inferred pseudotemporal trajectory among pancreatitis fibroblast subclusters and PDAC CAFs. Nodes (that is, circles) represent cell subsets, and their size is scaled to the number of cells. Edges (that is, lines) represent the strength of connectivity (that is, thickness). **g**, Significantly upregulated and downregulated pathways identified by GSEA of mouse PDAC CAFs compared with pancreatitis fibroblasts, as assessed by MAST. The in vivo myCAF and iCAF signatures are from Elyada et al. (mouse scRNA-seq dataset)^5^. The LRRC15^+^ CAF signature is from Dominguez et al.^8^. **h**, Selected ligand–receptor interactions and their strength based on CellChat analysis between fibroblasts and fibroblasts, epithelial cells, myeloids and endothelial cells in pancreatitis and PDAC. **i**, Selected collagen and fibronectin (FN1) pathways with different connections in mouse pancreatitis and PDAC, as assessed by CellChat analysis. **j**, A ligand activity plot of the top ligands in CAFs inferred to regulate target genes in malignant cells in mouse PDAC, as assessed by NicheNet analysis of snRNA-seq. **k**, A schematic of models and techniques used for the snRNA-seq analysis of mouse PDAC tissues derived from orthotopic transplantation models of *Smad4*-proficient (that is, wild-type (WT)) KRAS^G12D^ p53^R172H^ organoids (that is, KP PDAC) or *Smad4*-deficient (that is, knockout (KO)) KRAS^G12D^ p53^R172H^ organoids (that is, KPΔS PDAC). KP and KPΔS organoids were previously published in Lloyd et al.^28^. **l**, A UMAP plot of fibroblasts in the combined snRNA-seq dataset from mouse KP (*n* = 3) and KPΔS (*n* = 3) PDACs and pancreatitis (*n* = 6). Different subsets are colour coded. **m**, Significantly upregulated and downregulated pathways identified by GSEA of KP or KPΔS PDAC CAFs compared with pancreatitis fibroblasts, as assessed by MAST. The in vivo myCAF and iCAF signatures are from Elyada et al. (mouse scRNA-seq dataset)^5^. **n**, A UMAP plot of epithelial cells in the combined snRNA-seq dataset from mouse PDAC and pancreatitis. Different epithelial cell subsets are colour coded. **o**, PAGA illustrating the inferred pseudotemporal trajectory among pancreatitis Ductal 1 and Ductal 2 cells and PDAC malignant cells. Nodes (that is, circles) represent cell subsets, and their size is scaled to the number of cells. Edges (that is, lines) represent the strength of connectivity (that is, thickness). **p**, Significantly upregulated and downregulated pathways identified by GSEA of mouse PDAC malignant cells compared with pancreatitis Ductal 1 or Ductal 2 cells, as assessed by MAST. Inflammat, inflammatory; ECM−associat., ECM−associated; Proliferat., proliferative; Commun. prob., communication probability; AUPR, area under the precision-recall curve.

**Extended Data Fig. 3:**
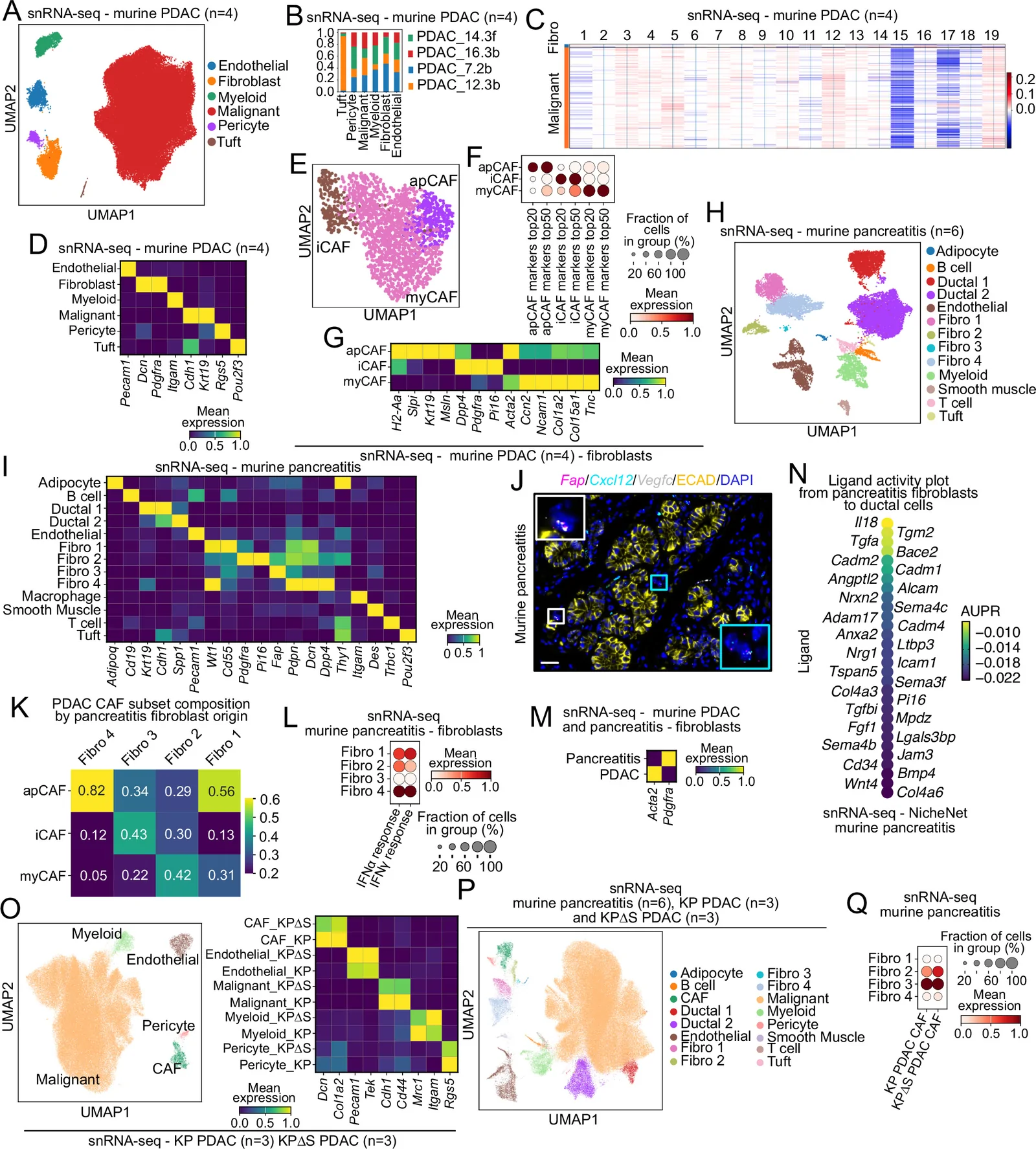
Pancreatitis and PDAC mouse models recapitulate fibroblast features of the human disease. **(A)** UMAP plot of cell types identified by snRNA-seq in murine PDAC (n = 4). Different cell types are colour coded. **(B)** Sample contribution to different cell types in murine PDAC (n = 4), represented as bar plot showing proportions of the different tumour samples in each cell cluster, as analysed by snRNA-seq. **(C)** Heatmap showing large-scale CNV profile of the fibroblast and epithelial malignant cell clusters identified by snRNA-seq of murine PDAC. The colour coding represents the CNV level based on a sliding window of 250 genes. Amplifications are shown in red, and deletions are shown in blue. The fibroblast cluster was used as reference cell cluster. **(D)** Heatmap of scaled expression of cell type-specific markers in each cell cluster of murine PDAC. Data are scaled such that the cluster with the lowest average expression = 0 and the highest = 1 for each gene. **(E)** UMAP plot of fibroblasts from murine PDAC (n = 4) coloured by CAF subclusters. **(F)** Dot plot of activity AUCell score (scaled) of PDAC CAF subtype signatures in each PDAC fibroblast cluster. CAF subtype signatures (that is, top 20 and top 50 markers) were derived from the snRNA-seq dataset of PDAC tumours from Mucciolo et al. **(G)** Heatmap of scaled expression of CAF subtype-specific markers in each fibroblast cluster of murine PDAC. Data are scaled such that the cluster with the lowest average expression = 0 and the highest = 1 for each gene. **(H)** UMAP plot of cell types identified by snRNA-seq in murine pancreatitis (n = 6). Different cell types are colour coded. Pancreatitis samples derive from one treatment cohort. **(I)** Heatmap of scaled expression of cell type-specific markers in each cell cluster of murine pancreatitis. Data are scaled such that the cluster with the lowest average expression = 0 and the highest = 1 for each gene. **(J)** Representative multiplex RNA *in situ* hybridization and immunofluorescence (IF) image of *Vegfc* (white), *Cxcl12* (cyan), *Fap* (pink) and DAPI (blue) to identify the Fibro 3 subset in murine pancreatitis tissues. E-cadherin, ECAD (yellow). Scale bar = 20 μm. The data shown are representative of two experimental replicates (each with the same 8 tissues). **(K)** Heatmap showing the fractional contribution of *in vivo* pancreatitis fibroblast subclusters to *in vivo* PDAC CAF subsets derived from averaged scANVI soft label probabilities. **(L)** Dot plot of activity AUCell score (scaled) of HALLMARK Interferon signatures in each fibroblast cluster from murine pancreatitis snRNA-seq dataset. **(M)** Heatmap of scaled expression of fibroblast markers (*Acta2*, *Pdgfra*) in fibroblasts from murine pancreatitis and PDAC from the snRNA-seq dataset. Data are scaled such that the cluster with the lowest average expression = 0 and the highest = 1 for each gene. **(N)** Ligand activity plot shows the top ligands of fibroblasts inferred to regulate target genes in ductal cells in murine pancreatitis, as assessed by NicheNet analysis of snRNA-seq. AUPR, area under the precision-recall curve. **(O)** Left: UMAP plot of cell types identified by snRNA-seq in murine KP (n = 3) and KPΔS (n = 3) PDACs. Different cell types are colour coded. Right: heatmap of scaled expression of cell type-specific markers in each cell cluster of murine KP and KPΔS PDAC. Data are scaled such that the cluster with the lowest average expression = 0 and the highest = 1 for each gene. **(P)** UMAP plot of cell types identified by snRNA-seq in murine KP PDAC (n = 3), KPΔS PDAC (n = 3) and pancreatitis (n = 6). Different cell types are colour coded. Pancreatitis samples are from Fig. 3. **(Q)** Dot plot of activity AUCell score (scaled) of KP and KPΔS PDAC CAF signatures in each pancreatitis fibroblast cluster.

To further investigate stromal differences between mouse pancreatitis and PDAC, we performed snRNA-seq of pancreatitis (*n* = 6) and GEMM-derived PDAC (*n* = 4) tissues (Fig. 3a and Supplementary Table 6). Malignant and non-malignant cell populations in GEMM-derived PDACs resembled those previously identified in organoid-derived PDACs^7,26^, including CAFs that sub-clustered into myCAF, iCAF and apCAF populations (Extended Data Fig. 3a–g). In pancreatitis, we identified four fibroblast subsets: ‘Fibro 1’ (*Wt1*^High^*Cd55*^High^), ‘Fibro 2’ (*Pdgfra*^High^), ‘Fibro 3’ (*Fap*^High^) and ‘Fibro 4’ (*Wt1*^High^*Dcn*^High^*Dpp4*^High^) (Fig. 3b and Extended Data Fig. 3h,i). Integration with multiplex RNA in situ hybridization (ISH) and immunofluorescence (IF) confirmed that these subsets are spatially interspersed and have distinct secretory programmes, including *Il34*^High^WT1^High^ Fibro 1, *Pi16*^High^PDGFRα^High^ Fibro 2, *Vegfc*^High^*Fap*^High^ and/or *Cxcl12*^High^*Fap*^High^ Fibro 3, and *Muc16*^High^WT1^High^ Fibro 4 (Fig. 3c and Extended Data Fig. 3i,j). Comparative snRNA-seq analyses showed that Fibro 3 was the most similar pancreatitis fibroblast subset to PDAC CAFs, on the basis of enrichment of the PDAC CAF signature (top 50 marker genes) (Fig. 3d) and semi-supervised single-cell annotation using variational inference (scANVI) mapping^27^ (Fig. 3e). Mapping to CAF subsets suggested that Fibro 3 was most similar to iCAFs (43%), Fibro 2 to myCAFs (42%) and Fibro 1 and Fibro 4 to apCAFs (56% and 82%) (Extended Data Fig. 3k). Pseudotemporal trajectory analysis further suggested that Fibro 3 represents a direct precursor state of PDAC CAFs (Fig. 3f).

To further compare mouse PDAC CAFs and pancreatitis fibroblasts, we assessed pathways altered in the snRNA-seq dataset. Consistent with human scRNA-seq analyses, myofibroblastic signatures were enriched in PDAC CAFs compared with pancreatitis fibroblasts, whereas inflammatory signatures were enriched in pancreatitis fibroblasts (Fig. 3g). Among these, interferon responses were highest in Fibro 4 (Extended Data Fig. 3l). Consistent with immunohistochemistry, snRNA-seq also showed increased *Pdgfra* expression in pancreatitis fibroblasts and higher *Acta2* expression (encoding αSMA) in PDAC CAFs (Extended Data Fig. 3m). CellChat analysis highlighted differences in ECM-associated ligands (for example, collagens and fibronectin (*Fn1*)) involved in fibroblast-to-epithelial cell signalling in pancreatitis and PDAC (Fig. 3h,i). NicheNet analysis further inferred a more dominant role for myofibroblast-related mediators (for example, *Tgfb1*, *Col15a1* and *Tnc*) in PDAC CAF-to-malignant cell crosstalk than in pancreatitis fibroblast-to-ductal cell crosstalk (Fig. 3j and Extended Data Fig. 3n).

Together, these findings raised the question of whether myofibroblastic features of PDAC CAFs are conserved across genetically distinct tumour contexts. Our previous work showed that distinct PDAC genetic profiles shape the malignant cell transcriptome and stroma^28,29^. We therefore tested whether increased myofibroblastic CAF features in KRAS^G12D^ p53-mutant PDACs relative to pancreatitis fibroblasts are also preserved in SMAD4-deficient KRAS^G12D^ p53-mutant PDACs. We focused on SMAD4 loss because it is frequent in human PDAC and we previously showed that SMAD4-deficient PDACs shift from myCAFs to iCAFs^28,30^. We established orthotopically grafted PDAC mouse models using either KPC GEMM-derived organoids (KP PDAC) or these organoids CRISPR-engineered to delete *Smad4*^28^ (KPΔS PDAC) (Fig. 3k). We generated and integrated snRNA-seq data from KP (*n* = 3) and KPΔS (*n* = 3) PDACs with the mouse pancreatitis dataset (Extended Data Fig. 3o,p and Supplementary Table 7). CAF signatures from both PDAC models (top 50 marker genes) were enriched in Fibro 3, consistent with KPC GEMMs (Extended Data Fig. 3q). CAFs from both KP and KPΔS PDACs also showed increased myofibroblastic signatures compared with pancreatitis fibroblasts, again mirroring KPC GEMMs (Fig. 3l,m).

We next asked whether fibroblasts from pancreatitis mouse models and PDAC GEMMs recapitulate human fibroblast gene expression patterns. Using human scRNA-seq and mouse snRNA-seq datasets, we focused on curated protein-coding orthologues. We identified 2,737 orthologues with significantly higher expression (*P* adjusted <0.05) in mouse PDAC CAFs compared with pancreatitis fibroblasts (‘mouse PDAC CAF orthologues’) and 3,011 orthologues with significantly higher expression in human PDAC CAFs compared with pancreatitis fibroblasts (‘human PDAC CAF orthologues’). Of the human PDAC CAF orthologues, 28% (*n* = 829/3,011) overlapped with mouse PDAC CAF orthologues (*P* = 3.47 × 10^−59^) (Supplementary Table 8). For pancreatitis fibroblast markers, we identified 6,125 mouse and 6,461 human orthologues with significantly higher expression in pancreatitis fibroblasts compared with PDAC CAFs (‘mouse pancreatitis fibroblast orthologues’ and ‘human pancreatitis fibroblast orthologues’, respectively). Of the human pancreatitis fibroblast orthologues, 54% (*n* = 3,494/6,461) overlapped with mouse pancreatitis fibroblast orthologues (*P* = 5 × 10^−256^) (Supplementary Table 8). Thus, these pancreatitis and PDAC mouse models significantly recapitulate gene markers of human pancreatitis and PDAC fibroblasts.

**Extended Data Fig. 4:**
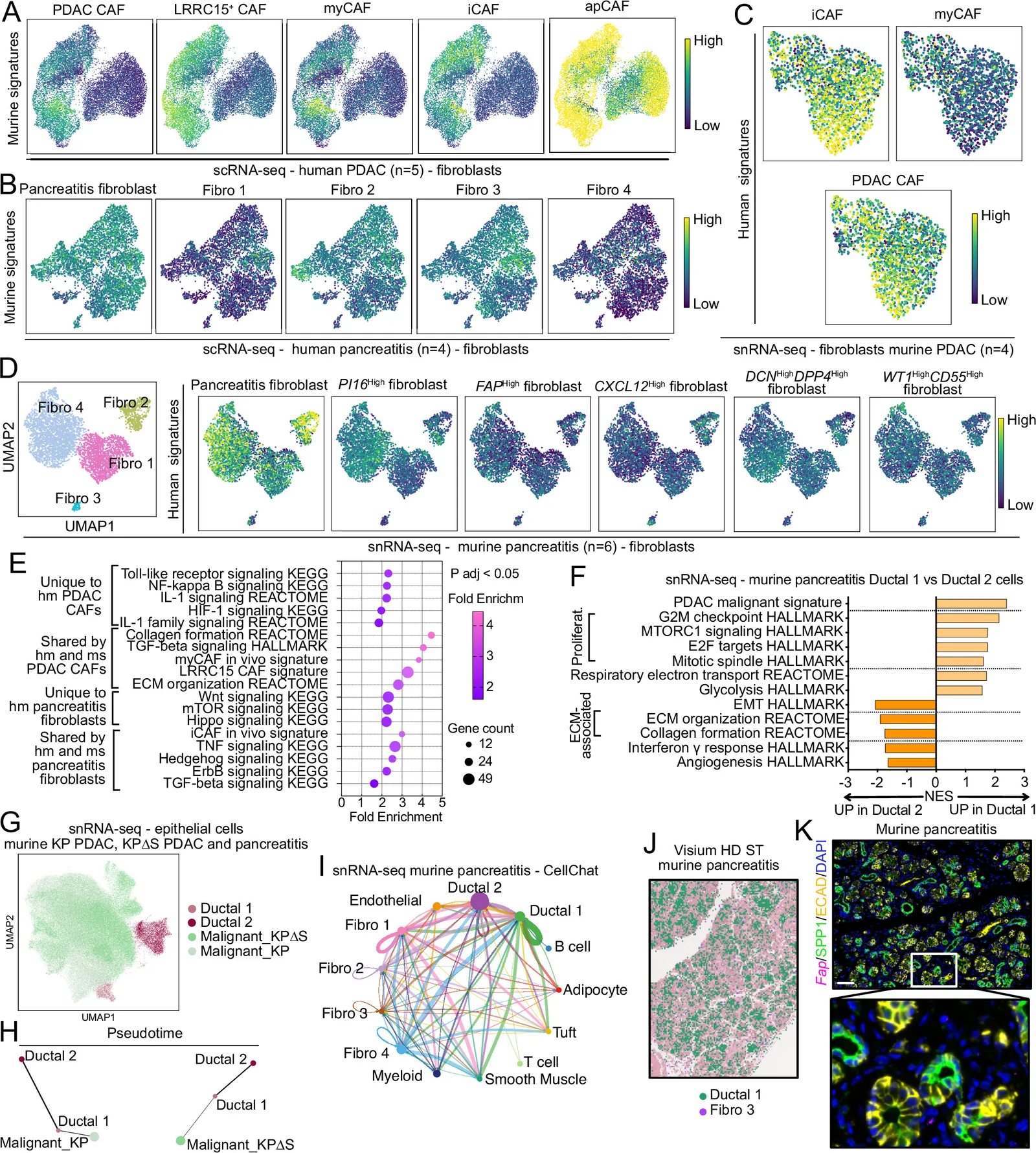
Pancreatitis and PDAC mouse models recapitulate fibroblast and epithelial features of the human disease. **(A)** UMAPs of human fibroblasts from the PDAC scRNA-seq dataset (n = 5) coloured by the activity (AUCell score) of murine PDAC CAF signatures (human orthologs), as identified by snRNA-seq, as well as the murine LRRC15^+^ CAF signature (from Dominguez et al). **(B)** UMAPs of human fibroblasts from the chronic pancreatitis scRNA-seq dataset (n = 4) coloured by the activity (AUCell score) of murine pancreatitis fibroblast signatures (human orthologs), as identified by snRNA-seq. **(C)** UMAPs of murine fibroblasts from the PDAC snRNA-seq dataset (n = 4) coloured by the activity (AUCell score) of human PDAC CAF signatures (mouse orthologs), as identified by scRNA-seq. **(D)** UMAPs of murine fibroblasts from the pancreatitis snRNA-seq dataset (n = 6) coloured by subset (left) or activity (AUCell score) of human pancreatitis fibroblast signatures (mouse orthologs), as identified by scRNA-seq. **(E)** Significantly enriched pathways (*P* adj < 0.05) based on the genes that are 1) shared by murine and human PDAC CAFs, 2) unique to human relative to murine PDAC CAFs, 3) shared by murine and human pancreatitis fibroblasts, and 4) unique to human relative to murine pancreatitis fibroblasts. *P* adj values were obtained from over-representation analysis performed using clusterProfiler enricher function, with all expressed genes in the dataset used as the background universe. **(F)** Significantly upregulated and downregulated pathways identified by GSEA of pancreatitis Ductal 1 compared to Ductal 2 cells, as assessed by Model-based Analysis of Single-cell Transcriptomics (MAST) from the snRNA-seq dataset. The PDAC malignant cell signature was obtained by comparing murine PDAC malignant cells to pancreatitis ductal cells from the snRNA-seq dataset. Proliferat., proliferative. **(G)** UMAP plot of epithelial cells in the combined snRNA-seq dataset from KP PDAC (n = 3), KPΔS PDAC (n = 3) and pancreatitis (n = 6). Different epithelial cell subsets are colour coded. **(H)** PAGA illustrating the inferred pseudotemporal trajectory among pancreatitis Ductal 1 and Ductal 2 cells and KP (left) or KPΔS (right) PDAC malignant cells. Nodes (that is, circles) represent cell subsets, and their size is scaled to the number of cells. Edges (that is, line) represent the strength of connectivity (that is, thickness). **(I)** Visualization of inferred cell-cell communication using CellChat showing the connections between all cell types identified in murine pancreatitis, as assessed by snRNA-seq. The thickness of the connection reflects the number of interactions. **(J)** Representative example of Visium HD ST slide image of murine pancreatitis tissue showing only Ductal 1 and Fibro 3 populations. **(K)** Representative multiplex RNA *in situ* hybridization and IF image of DAPI (blue), *Fap* (pink), SPP1 (green) and ECAD (yellow) to identify *Fap*^High^ Fibro 3, SPP1^Low^ECAD^+^ Ductal 1 and SPP1^High^ECAD^+^ Ductal 2 populations in murine pancreatitis tissues. Insert shows magnification. Scale bars = 50 μm. The data shown are representative of two experimental replicates (each with the same 8 tissues). SPP1 was chosen to distinguish between Ductal 1 and Ductal 2 cells based on the snRNA-seq data of murine pancreatitis tissues (Extended Data Fig. 3I).

Our analyses also suggested that some features of human PDAC CAFs and pancreatitis fibroblasts are not fully captured in mouse models. Mapping mouse snRNA-seq signatures (the top 50 markers compared with other fibroblast subsets) (Supplementary Table 9) onto the human scRNA-seq dataset using AUCell scoring^31^ showed that Fibro 2 and Fibro 3 aligned more closely with human pancreatitis fibroblasts than Fibro 1 and Fibro 4, whereas mouse CAF signatures better aligned with the human myCAF cluster (Extended Data Fig. 4a,b). Reciprocal mapping of human signatures (Supplementary Table 2) onto mouse snRNA-seq revealed that the human iCAF signature was better represented in mouse PDAC CAFs than the human myCAF signature (Extended Data Fig. 4c). Among human pancreatitis fibroblast signatures, the *PI16*^High^ and the *DCN*^High^*DPP4*^High^ subsets were more accurately captured by mouse models (Extended Data Fig. 4d). Pathway enrichment analysis of shared genes showed that mouse pancreatitis models capture both myofibroblastic and inflammatory signatures of human disease, whereas PDAC mouse models preferentially recapitulate human myofibroblastic programmes over inflammatory signatures (Extended Data Fig. 4e and Supplementary Table 8). This is notable because patients in our PDAC cohort had an average age of >65 years, and we recently showed that aged PDAC mouse models better recapitulate the biology of older patients, particularly inflammatory signatures, than the younger mice used here^32^. Incorporating age and genetic diversity into future models should therefore help capture the nuances of human fibroblast biology.

We next assessed whether epithelial gene expression differences between mouse pancreatitis and PDAC recapitulate those in humans. Analysis of mouse epithelial cell clusters identified two pancreatitis ductal cell populations, termed ‘Ductal 1’ and ‘Ductal 2’, and one PDAC malignant cell population (Fig. 3n). The PDAC malignant cell signature (top 50 marker genes) was enriched in Ductal 1 compared with Ductal 2 cells, suggesting that Ductal 1 cells are transcriptomically closer to PDAC malignant cells (Extended Data Fig. 4f). This was supported by pseudotemporal trajectory analysis, which inferred a dominant trajectory connecting pancreatitis Ductal 1 cells to PDAC malignant cells (Fig. 3o). Analysis of pancreatitis Ductal 1 and Ductal 2 cells with KP and KPΔS malignant cells suggested that this connection is independent of PDAC SMAD4 status (Extended Data Fig. 4g,h). CellChat analysis inferred greater communication between Fibro 3, which is transcriptionally closer to PDAC CAFs, and Ductal 1 cells than with other cell types (Extended Data Fig. 4i), potentially indicating an early shift towards malignant-like communication. We therefore asked whether this inferred interaction corresponded to a distinct spatial organization. Visium HD spatial transcriptomics showed that Fibro 3 and Ductal 1 populations were interspersed throughout pancreatitis tissues rather than confined to a discrete niche (Extended Data Fig. 4j). To validate these spatial relationships at single-cell resolution, we performed multiplex RNA ISH for *Fap* combined with IF for secreted phosphoprotein 1 (SPP1) and E-cadherin. Because *Spp1* was more highly expressed in Ductal 2 than Ductal 1 cells (Extended Data Fig. 3i), SPP1 enabled discrimination between ductal populations. This analysis confirmed that Fibro 3 do not form an isolated niche with Ductal 1 cells (Extended Data Fig. 4k). Instead, pancreatitis tissues show marked spatial heterogeneity, with SPP1^Low^ Ductal 1 and SPP1^High^ Ductal 2 cells often adjacent within the same ADM structures.

To further compare PDAC and pancreatitis epithelial cells, we analysed the malignant cluster against each pancreatitis ductal cluster. PDAC malignant cells upregulated proliferation-associated pathways compared with pancreatitis Ductal 2 cells, whereas ECM-associated and inflammatory signatures were enriched in PDAC malignant cells compared with Ductal 1 cells (Fig. 3p). Thus, mouse PDAC malignant cells capture proliferation- and inflammation-associated signatures also observed in human PDAC malignant cells relative to pancreatitis ductal cells.

Finally, we used a similar cross-species strategy to assess how well pancreatitis and PDAC epithelial cells in mouse models recapitulate human epithelial gene expression. We identified 3,804 orthologues with significantly higher expression in mouse PDAC malignant cells compared with pancreatitis ductal cells (‘mouse PDAC malignant orthologues’) and 1,751 orthologues with significantly higher expression in human PDAC malignant cells compared with pancreatitis ductal cells (‘human PDAC malignant orthologues’). Of the human PDAC malignant orthologues, 43% (*n* = 755/1,751) overlapped with mouse PDAC malignant orthologues (*P* = 1.1 × 10^−81^) (Supplementary Table 8). We also identified 5,378 mouse and 4,593 human orthologues with significantly higher expression in pancreatitis ductal cells compared with PDAC malignant cells (‘mouse pancreatitis ductal orthologues’ and ‘human pancreatitis ductal orthologues’, respectively). Of the human pancreatitis ductal orthologues, 47% (*n* = 2,160/4,593) overlapped with mouse pancreatitis ductal orthologues (*P* = 8.98 × 10^−115^) (Supplementary Table 8). These data indicate that pancreatitis and PDAC epithelial cells from mouse models significantly capture human gene expression patterns.

Together, these data reveal heterogeneous fibroblast and epithelial cell populations in pancreatitis and highlight candidate precursors of PDAC CAFs and malignant cells. They also support further comparative analyses of epithelial cells derived from our mouse models to investigate biology relevant to the respective human diseases.

### Pancreatitis organoids recapitulate in vivo ductal cell gene expression

**Fig. 4:**
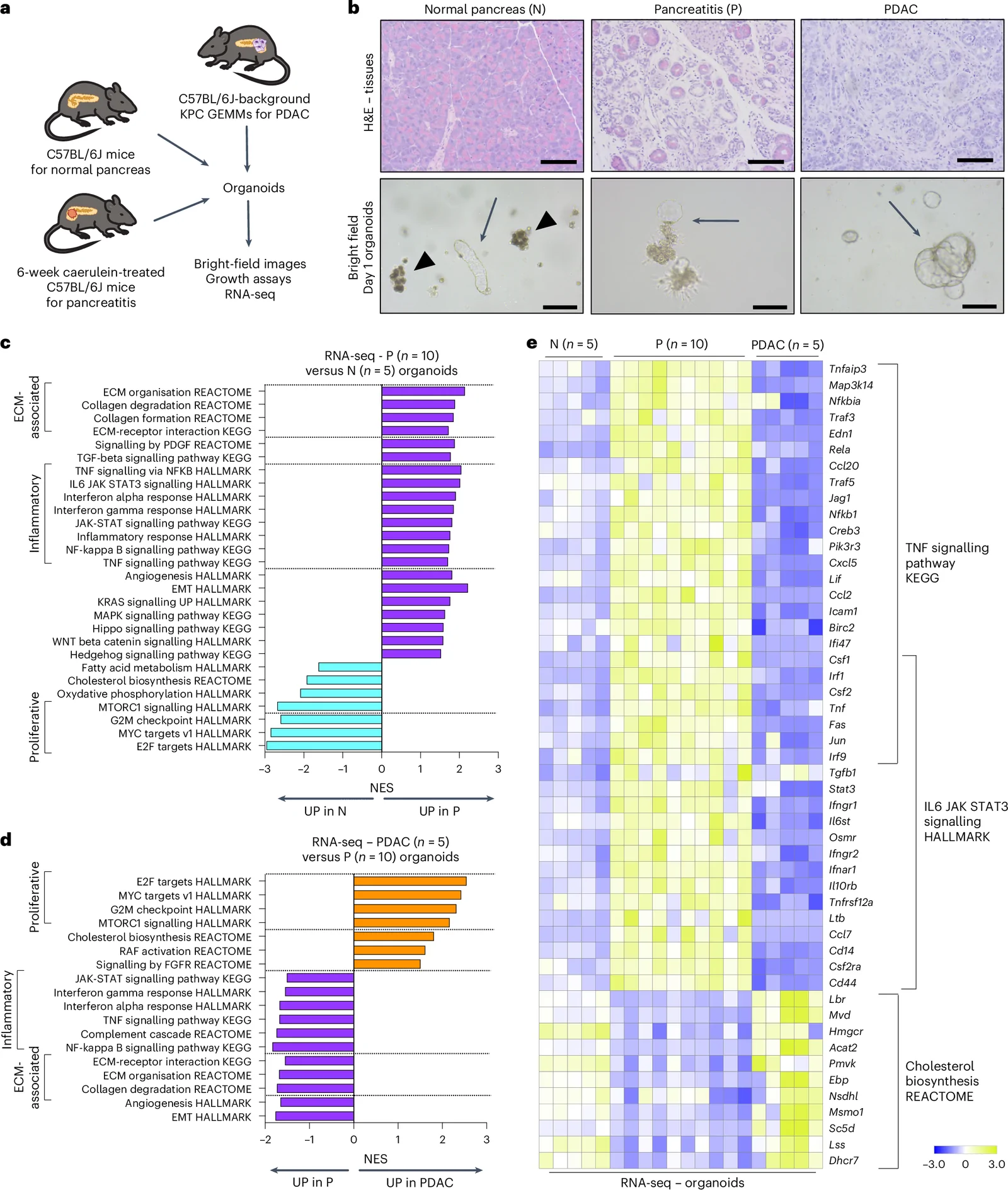
Pancreatitis-derived epithelial organoids recapitulate in vivo gene expression patterns of pancreatitis ductal cells. **a**, A schematic of models used for the generation of normal pancreas (that is, from untreated mice), pancreatitis (that is, from 6-week caerulein-treated mice) and PDAC (that is, from KPC GEMMs) organoids, as well as the techniques used for their analysis. Pancreatitis organoids were derived from one treatment cohort. **b**, Top: representative images of H&E stains of normal pancreas, pancreatitis and PDAC tissues. Scale bars, 50 μm. Bottom: bright-field images of epithelial organoids generated from normal pancreas (N), pancreatitis (P) and PDAC tissues at day 1 post-isolation. Scale bars, 100 μm. Arrows indicate budding organoids. Triangles indicate acinar cell clusters. **c**, Significantly upregulated and downregulated pathways identified by GSEA of P organoids (*n* = 10, *n* = 5 lines in two passages) compared with N organoids (*n* = 5), as assessed by RNA-seq. **d**, Significantly upregulated and downregulated pathways identified by GSEA of PDAC organoids (*n* = 5) compared with P organoids (*n* = 10, *n* = 5 lines in two passages), as assessed by RNA-seq. **e**, A heat map plot showing the normalized counts from RNA-seq of P (*n* = 10, *n* = 5 lines in two passages), N (*n* = 5) and PDAC (*n* = 5) organoids. Selected genes and pathways upregulated or downregulated across conditions are shown.

**Extended Data Fig. 5:**
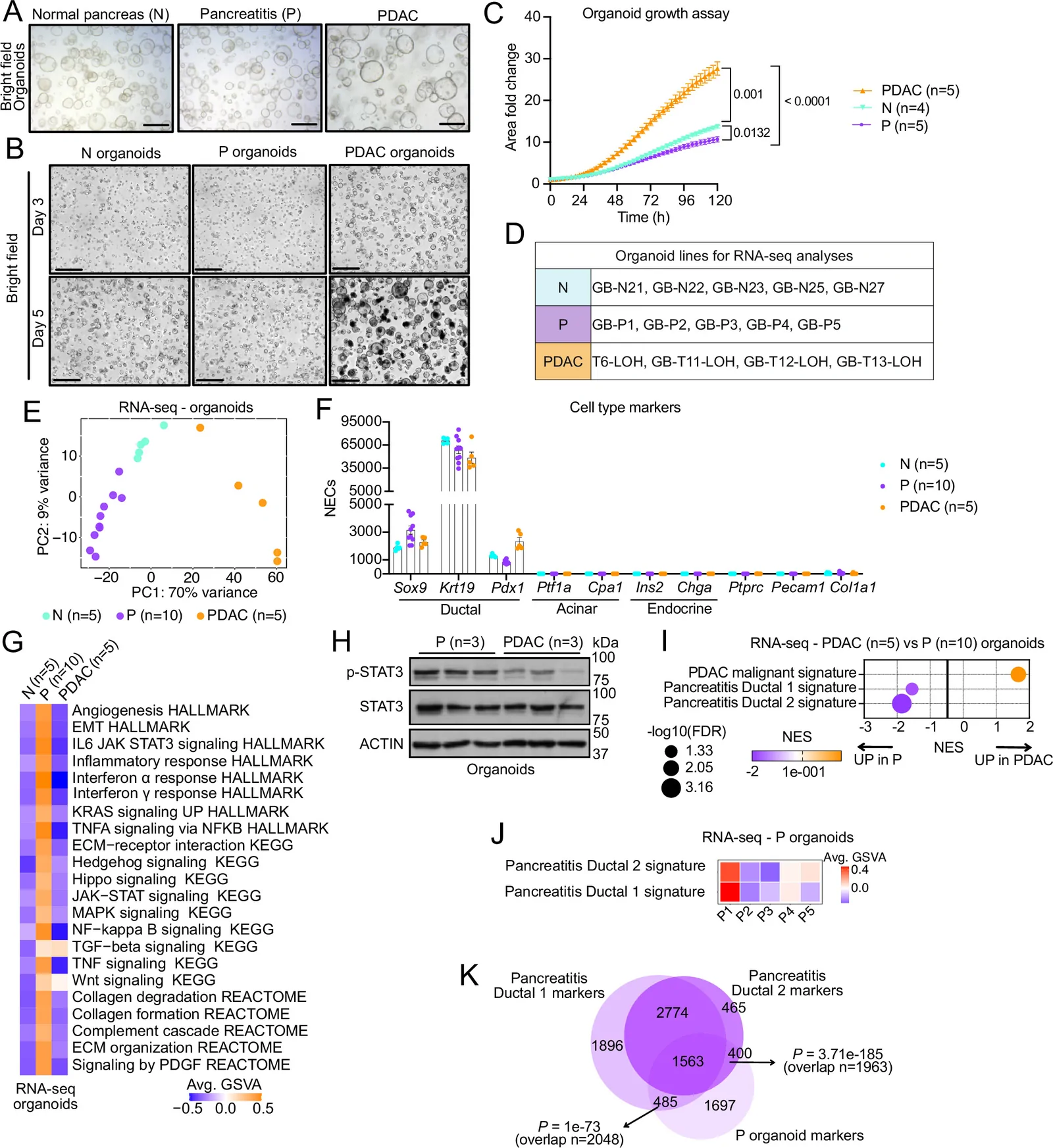
Pancreatitis-derived epithelial organoids capture *in vivo* gene expression patterns of pancreatitis ductal cells. **(A)** Bright-field images of organoids generated from normal pancreas (hereafter, termed N), pancreatitis (hereafter, termed P) and PDAC tissues after > 5 passages; scale bars = 200 μm. Pancreatitis organoids derive from the same treatment cohort. The data shown are representative of four experimental replicates (each with a different organoid line). **(B)** Bright field images (taken with an Incucyte organoid module) of N, P and PDAC organoids at day 3 and day 5 post-plating, as part of a growth assay; scale bars = 800 μm. The data shown are representative of five experimental replicates (each with four technical replicates) per condition. **(C)** Proliferation curves of murine N (n = 4), P (n = 5) and PDAC (n = 5, n = 4 lines with one in two passages) organoids in complete media. Data are from five independent experiments. Results show mean ± SEM. *P adj*, Kruskal-Wallis test for the last time point. **(D)** Summary table of the N, P and PDAC organoid lines analysed by RNA-sequencing (RNA-seq) after 3 days of culture in complete media. **(E)** Principal component analysis (PCA) of N (n = 5), P (n = 10, n = 5 lines in two passages) and PDAC (n = 5, n = 4 lines with one in two passages) organoids, as assessed by RNA-seq. **(F)** RNA-seq expression of ductal (*Sox9*, *Krt19*, *Pdx1*), acinar (*Ptf1a*, *Cpa1*), endocrine (*Ins2*, *Chga*), immune (*Ptprc*), endothelial (*Pecam1*) and fibroblast (*Col1a1*) markers in N (n = 5), P (n = 10, n = 5 lines in two passages) and PDAC (n = 5, n = 4 lines with one in two passages) organoids. Results show mean ± SEM. NECs, normalized expression counts. **(G)** Heatmap showing Gene Set Variation Analysis (GSVA) enrichment scores (averaged per group) of selected pathways in N, P and PDAC organoids, as assessed by RNA-seq. Pathways displayed correspond to pathways significantly upregulated in GSEA in P organoids (Fig. 4e and/or 4 F). **(H)** Western blot of phospho-STAT3 (p-STAT3) and STAT3 in P (n = 3) and PDAC (n = 3) organoids cultured in complete media for 3 days. ACTIN, loading control. The samples were obtained from two experiments, each comprising P and PDAC organoids. **(I)** GSEA of the murine pancreatitis Ductal 1 and Ductal 2 signatures and of the PDAC malignant signature in PDAC organoids compared to P organoids. The pancreatitis Ductal signatures were obtained by comparing pancreatitis Ductal 1 and Ductal 2 cells, respectively, to PDAC malignant cells from the murine snRNA-seq dataset. The PDAC malignant signature was obtained by comparing PDAC malignant cells to pancreatitis ductal cells from the murine snRNA-seq dataset. (**J**) Heatmap showing GSVA scores (averaged per P organoid line) of murine pancreatitis Ductal 1 and Ductal 2 signatures, as assessed by RNA-seq. (**K**) Venn diagrams of curated protein-coding genes significantly (*P* adj < 0.05) upregulated in P organoids cultured in complete media compared to PDAC organoids cultured in complete media (hereafter, ‘P organoid markers’) and murine pancreatitis Ductal 1 or Ductal 2 cells compared to PDAC malignant cells *in vivo* (hereafter, ‘pancreatitis Ductal 1 markers’ and ‘pancreatitis Ductal 2 markers’), as assessed by RNA-seq and snRNA-seq, respectively. Significance of the overlap between datasets was defined by hypergeometric test (with denominator n = 18,000). Source numerical data are available.

PDAC epithelial organoid/PSC co-cultures have advanced understanding of CAF heterogeneity by identifying iCAFs, myCAFs and malignant cell-derived mediators of their reprogramming^4,33^. We reasoned that an analogous pancreatitis model could distinguish fibroblast and epithelial features of inflammatory and malignant states. We therefore generated pancreatitis epithelial organoids^34^, hereafter ‘P organoids’, from pancreata of C57BL/6J mice treated with caerulein for 6 weeks, and compared them with normal pancreatic organoids (hereafter, ‘N organoids’) and PDAC organoids derived from C57BL/6J normal pancreata and KPC GEMM tumours, respectively (Fig. 4a). At day 1 post-isolation, N organoids showed the expected budding ductal-like morphology, and PDAC organoids formed large cystic clusters^35^ (Fig. 4b). By contrast, P organoids formed small, relatively disorganized cystic structures, suggesting a distinct origin from N organoids. After two to three passages, N, P and PDAC organoids had similar morphologies, although P organoids grew more slowly (Extended Data Fig. 5a–c). Consistently, RNA-seq showed enrichment of proliferation-associated signatures in N and PDAC organoids compared with P organoids (Fig. 4c,d, Extended Data Fig. 5d,e and Supplementary Table 10). As expected, N, P and PDAC organoids expressed ductal epithelial markers, including *Sox9*, *Pdx1* and *Krt19*^35^ (Extended Data Fig. 5f).

RNA-seq revealed marked transcriptomic differences among N, P and PDAC organoids. Compared with N organoids, P organoids upregulated ECM-associated and inflammatory pathways, including JAK-STAT signalling (Fig. 4c,e and Extended Data Fig. 5g). These pathways were also enriched in P organoids relative to PDAC organoids, consistent with higher STAT3 activation, indicated by phospho-STAT3 levels, in P compared with PDAC organoids (Fig. 4d,e and Extended Data Fig. 5g,h). Conversely, as expected, the PDAC malignant cell signature derived from snRNA-seq profiles was enriched in PDAC organoids (Extended Data Fig. 5i). Importantly, gene signatures upregulated in pancreatitis Ductal 1 and Ductal 2 snRNA-seq profiles relative to PDAC malignant cells were enriched in P organoids, although with heterogeneity across lines (Extended Data Fig. 5i,j). Accordingly, the 4,145 genes expressed at higher levels in P than PDAC organoids (‘P organoid markers’) significantly overlapped with genes enriched in mouse Ductal 1 and Ductal 2 snRNA-seq profiles relative to PDAC malignant cells (‘pancreatitis Ductal 1 markers’ and ‘pancreatitis Ductal 2 markers’, respectively) (Extended Data Fig. 5k and Supplementary Table 8).

Together, these findings show that P organoids retain features of in vivo pancreatitis ductal cells and provide a model to investigate epithelial cell–fibroblast crosstalk in pancreatitis.

### Fibroblast signalling more strongly shapes pancreatitis ductal cells than PDAC malignant cells

**Fig. 5:**
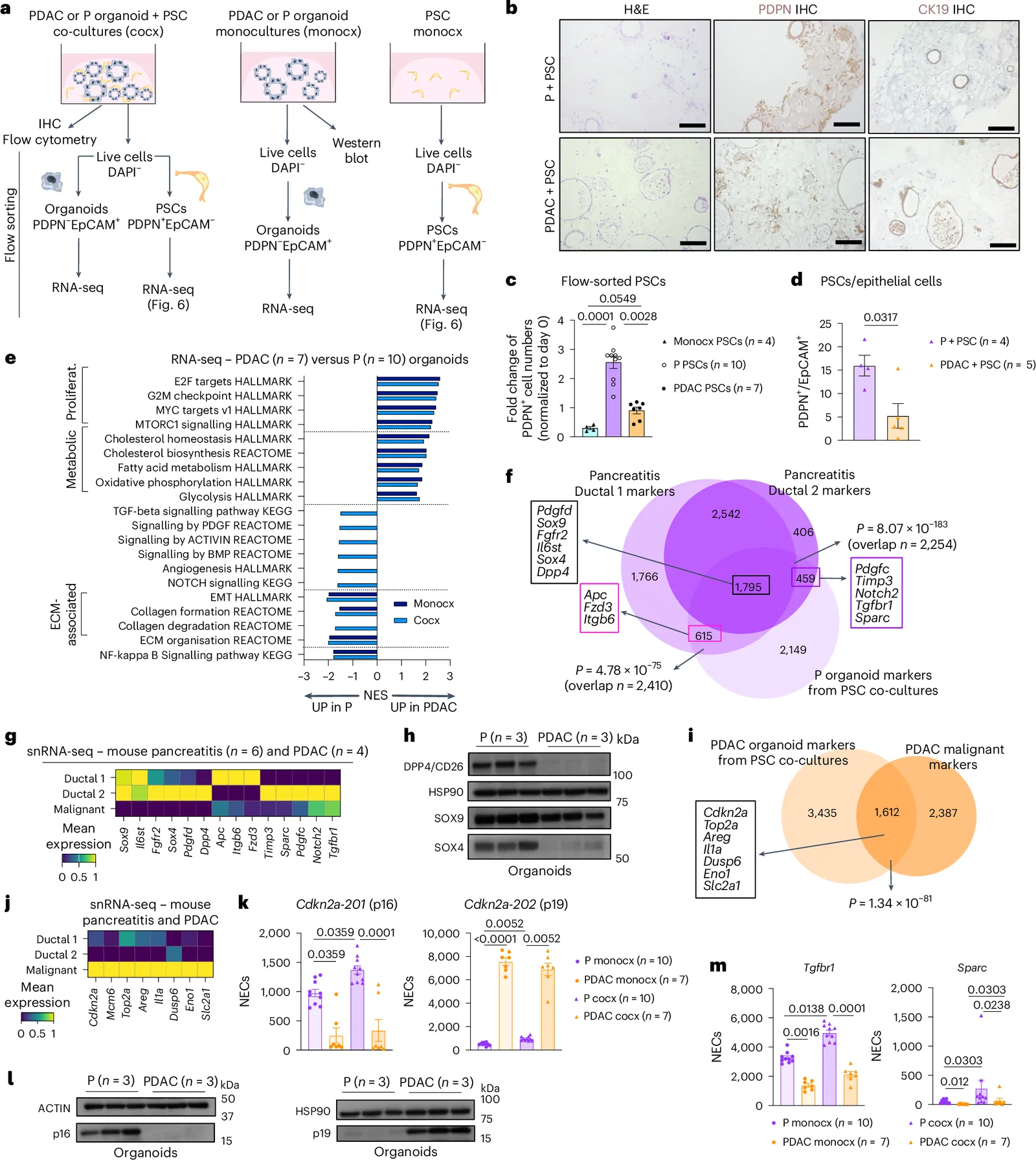
The transcriptome of pancreatitis ductal cells is more influenced by fibroblast signalling compared with PDAC malignant cells. **a**, A schematic of the flow sorting strategy of organoid and PSC monocultures (monocx) and P or PDAC organoid/PSC co-cultures (cocx) in reduced media (RM; that is, 5% FBS/DMEM). **b**, Representative images of H&E, podoplanin (PDPN) and CK19 stains of P or PDAC cocx after 7 days in culture. Scale bars, 100 μm. The data shown are representative of five P cocx and six PDAC cocx from three independent experiments. **c**, The fold change in numbers of PSCs (PDPN^+^EpCAM^−^) flow-sorted from monocx (*n* = 4, *n* = 2 lines in two passages) or from P (*n* = 10 PSC/organoid pairs) or PDAC (*n* = 7 PSC/organoid pairs) cocx after 3.5 days in culture relative to the number of PSCs plated at day 0. Each data point corresponds to a biological replicate. Results show mean ± s.e.m. Adjusted *P* values from Kruskal–Wallis tests. **d**, The fibroblast (PDPN^+^EpCAM^−^):epithelial cell (EpCAM^+^PDPN^−^) ratio as assessed by flow cytometry of P (*n* = 4 PSC/organoid pairs) or PDAC (*n* = 5 PSC/organoid pairs) cocx after 3.5 days in culture. Results show mean ± s.e.m. *P* value from a Mann–Whitney test. **e**, Significantly upregulated and downregulated pathways identified by GSEA of PDAC organoids in monocx (*n* = 7, *n* = 4 lines with three in two passages) or cocx (*n* = 7 PSC/organoid pairs) compared with P organoids in monocx (*n* = 10, *n* = 5 lines in two passages) or cocx (*n* = 10 PSC/organoid pairs), respectively, as assessed by RNA-seq. **f**, Venn diagrams of curated protein-coding genes significantly (adjusted *P* <0.05) upregulated in P organoids in cocx compared with PDAC organoids in cocx (‘P organoid markers from PSC co-cultures’) and mouse pancreatitis Ductal 1 or Ductal 2 cells compared with PDAC malignant cells in vivo (‘pancreatitis Ductal 1 markers’ and ‘pancreatitis Ductal 2 markers’), as assessed by RNA-seq and snRNA-seq, respectively. Significance of the overlap between datasets was defined by a hypergeometric test (with a denominator of *n* = 18,000). **g**, A heat map of the scaled expression of selected genes differentially upregulated in mouse pancreatitis ductal cells in mouse pancreatitis and PDAC epithelial clusters. Data are scaled such that the cluster with the lowest average expression is 0 and the highest is 1 for each gene. **h**, Western blot analysis of DPP4, SOX9 and SOX4 in P (*n* = 3) and PDAC (*n* = 3) organoids cultured in RM for 3 days. HSP90 is the loading control. **i**, Venn diagrams of curated protein-coding genes significantly upregulated in mouse PDAC malignant cells compared with pancreatitis ductal cells in vivo (‘PDAC malignant markers’) and mouse PDAC organoids in cocx compared with mouse P organoids in cocx (‘PDAC organoid markers from PSC co-cultures’), as assessed by snRNA-seq and RNA-seq, respectively. The significance of the overlap between datasets was defined by hypergeometric test (with a denominator of *n* = 18,000). **j**, A heat map of the scaled expression of genes differentially upregulated in mouse PDAC malignant cells in mouse pancreatitis and PDAC ductal clusters. Data are scaled such that the cluster with the lowest average expression is 0 and the highest is 1 for each gene. **k**, The RNA-seq expression of *Cdkn2a-201* (encoding p16) and *Cdkn2a-202* (encoding p19) in P monocx (*n* = 10, *n* = 5 lines in two passages), P cocx (*n* = 10 PSC/organoid pairs), PDAC monocx (*n* = 4 lines with three samples in two passages) or PDAC cocx (*n* = 7 PSC/organoid pairs). Each data point corresponds to a biological replicate. Results show mean ± s.e.m. Adjusted *P* values from Kruskal–Wallis tests. **l**, Western blot analysis of p19 and p16 in P (*n* = 3) and PDAC (*n* = 3) organoids cultured in RM for 3 days. ACTIN and HSP90 were used as loading controls. **m**, RNA-seq expression of *Tgfbr1* and *Sparc* in P monocx (*n* = 10, *n* = 5 lines in two passages), P cocx (*n* = 10 PSC/organoid pairs), PDAC monocx (*n* = 4 lines with three samples in two passages) or PDAC cocx (*n* = 7 PSC/organoid pairs). Each data point corresponds to a biological replicate. Results show mean ± s.e.m. Adjusted *P* values from Kruskal–Wallis tests. NECs, normalized expression counts.

**Extended Data Fig. 6:**
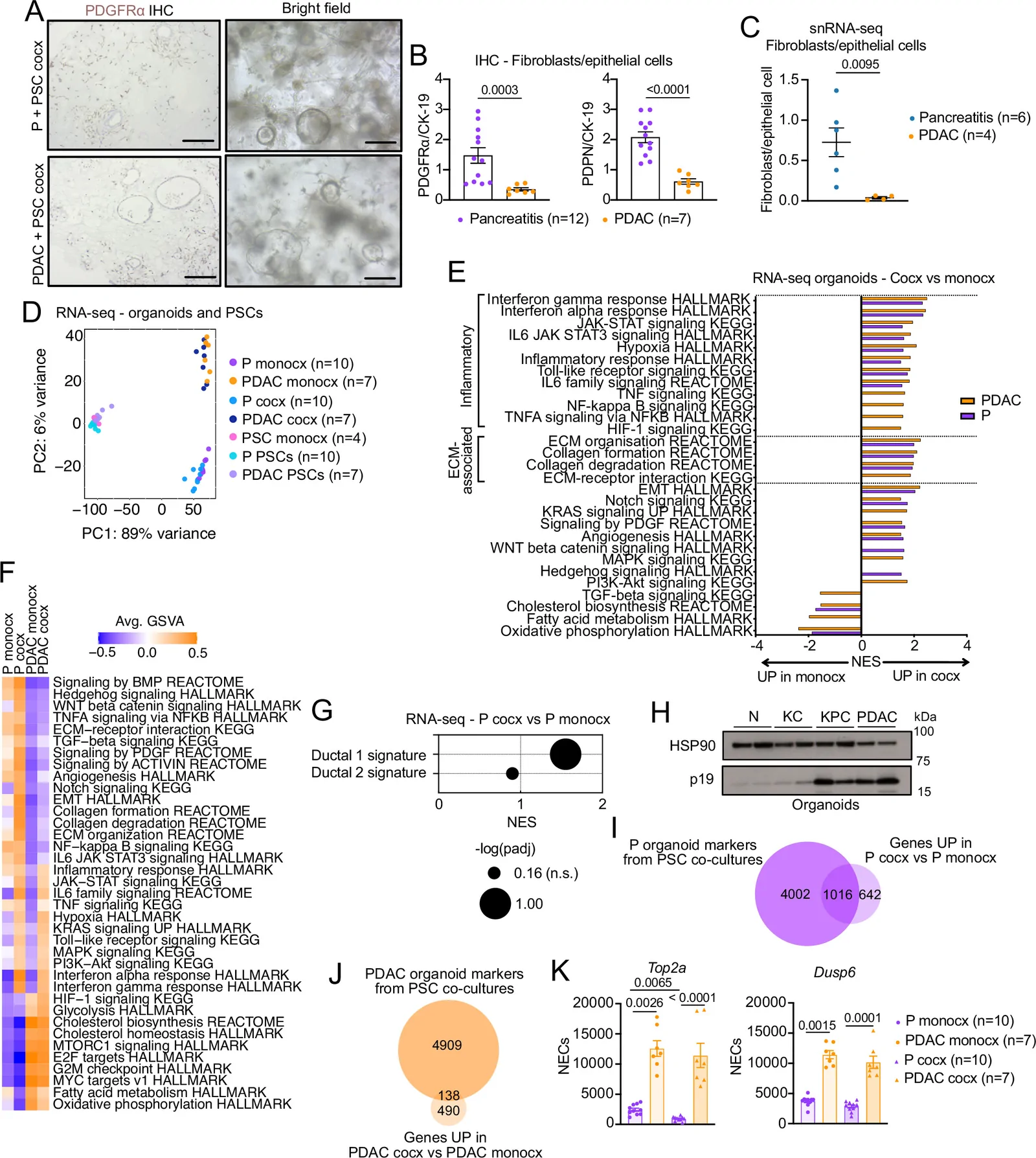
The transcriptome of pancreatitis ductal cells is more influenced by fibroblast signalling compared to PDAC malignant cells. (**A**) Left: Representative images of PDGFRα stains (left) or bright field (right) of P and PDAC organoid/pancreatic stellate cell (PSC) co-cultures (cocx) after 7 days in culture. Scale bars = 100 μm. The data shown are representative of 5 P cocx and 6 PDAC cocx from three independent experiments. (**B**) Fibroblast (PDGFRα^+^)/epithelial cell (CK19^+^) ratio (left) or fibroblast (PDPN^+^)/epithelial cell (CK19^+^) ratio (right) as assessed by immunohistochemical analysis of murine pancreatitis (n = 12) or PDAC (n = 7) tissues (from Fig. 2). Results show mean ± SEM. *P*, Mann-Whitney test. (**C**) Ratio of fibroblast to epithelial cell numbers per sample as determined by snRNA-seq analysis of murine pancreatitis (n = 6) and PDAC (n = 4) tissues (from Fig. 3). Results show mean ± SEM. *P*, Mann-Whitney test. (**D**) PCA of P organoids in monocultures (that is, P monocx, n = 10, n = 5 lines in two passages) or in co-culture with PSCs (that is, P cocx, n = 10 PSC/organoid pairs), PDAC organoids in monocultures (that is, PDAC monocx, n = 4 lines with three samples in two passages) or in co-culture with PSCs (that is, PDAC cocx, n = 7 PSC/organoid pairs), and PSC monocultures (that is, monocx PSCs, n = 2 lines in two passages) and in co-culture with PDAC organoids (that is, PDAC PSCs, n = 7 PSC/organoid pairs) or P organoids (that is, P PSCs, n = 10 PSC/organoid pairs), as assessed by RNA-seq. (**E**) Significantly upregulated and downregulated pathways identified by GSEA of P or PDAC organoids in cocx compared to their respective monocx, as assessed by RNA-seq. (**F**) Heatmap showing GSVA enrichment scores (averaged per group) of selected pathways in P and PDAC monocx and cocx, as assessed by RNA-seq. Pathways displayed correspond to pathways significantly altered in GSEA (Fig. 5e and/or Extended Data Fig. 5G). (**G**) GSEA of the murine pancreatitis Ductal 1 (top) and Ductal 2 (bottom) signatures in P cocx compared to P monocx. The pancreatitis Ductal 2 signature was not significantly different between conditions. (**H**) Western blot of p19 in N (n = 2) and PDAC organoids (n = 2), as well as PDAC organoids that have not undergone loss of heterozygosity of the wild-type *Trp53* allele (that is, KPC organoids, n = 2) and pre-cancerous pancreatic intraepithelial neoplasia organoids from KC mice (KC organoids, n = 2) cultured in reduced media for 3 days. ACTIN, loading control. The samples were obtained in two experiments, each comprising P and PDAC organoids. (**I**) Venn diagrams of curated protein-coding significantly (*P* adj < 0.05) upregulated genes in P cocx compared to PDAC cocx (hereafter, ‘P organoid markers from PSC co-cultures’) and curated protein-coding significantly upregulated genes in P cocx compared to P monocx. (**J**) Venn diagrams of curated protein-coding significantly (*P* adj < 0.05) upregulated genes in PDAC cocx compared to P cocx (hereafter, ‘PDAC organoid markers from PSC co-cultures’) and curated protein-coding significantly upregulated genes in PDAC cocx compared to PDAC monocx. (**K**) RNA-seq expression of *Top2a* and *Dusp6* in P monocx (n = 10, n = 5 lines in two passages), P cocx (n = 10 PSC/organoid pairs), PDAC monocx (n = 4 lines with three samples in two passages) or PDAC cocx (n = 7 PSC/organoid pairs). Each data point corresponds to a biological replicate. Results show mean ± SEM. *P adj*, Kruskal-Wallis test. Source numerical data and unprocessed blots are available.

To investigate epithelial–fibroblast crosstalk in PDAC and pancreatitis, we analysed five culture conditions: P organoids, PDAC organoids or PSCs alone, to assess cell-intrinsic gene expression, and P or PDAC organoids co-cultured with PSCs, to assess the impact of fibroblast signalling on epithelial gene expression (Fig. 5a,b and Extended Data Fig. 6a). PSC numbers were higher in P co-cultures than in PDAC co-cultures or PSC monocultures, indicating that pancreatitis epithelial cells promote PSC expansion more effectively than PDAC malignant cells (Fig. 5c). Consistent with mouse tissue immunohistochemistry and snRNA-seq, P co-cultures also showed higher fibroblast-to-epithelial cell ratios than PDAC co-cultures (Fig. 5d and Extended Data Fig. 6b,c). Thus, P and PDAC organoid/PSC co-cultures capture epithelial–fibroblast features characteristic of pancreatitis and PDAC in vivo.

Consistent with tissue transcriptomic analyses, proliferation-associated signatures were enriched in PDAC compared with P organoids, regardless of culture condition (Fig. 5e, Extended Data Fig. 6d and Supplementary Table 11). By contrast, pathways upregulated in P relative to PDAC organoids were largely PSC-dependent. These included PDGF, transforming growth factor beta (TGF-β) and NOTCH signalling, known mediators of epithelial–stromal crosstalk^2^. Although both P and PDAC organoids upregulated inflammatory and ECM-associated pathways in co-culture, these enrichments were highest in P organoid co-cultures (Extended Data Fig. 6e,f). Thus, both organoid types respond to PSC-derived cues, but distinct epithelial–fibroblast crosstalk operates in pancreatitis and PDAC. Finally, the mouse pancreatitis Ductal 1, but not Ductal 2, signature was enriched in P organoids from co-cultures relative to monocultures, suggesting that the in vivo Ductal 1 state may partly depend on stromal signalling (Extended Data Fig. 6g).

To determine whether PSC co-cultures induced epithelial gene expression patterns resembling mouse pancreatitis and PDAC tissues, we applied our cross-model strategy. We identified 5,018 genes expressed at higher levels in P than PDAC co-cultures (‘P organoid markers from PSC co-cultures’) and overlapped them with ‘pancreatitis Ductal 1 markers’ and ‘pancreatitis Ductal 2 markers’ defined from mouse snRNA-seq profiles. P organoid markers from PSC co-cultures significantly overlapped with both pancreatitis Ductal 1 and Ductal 2 markers (Fig. 5f and Supplementary Table 8). This also identified genes upregulated in both ductal populations, or selectively in Ductal 1 or Ductal 2 cells, relative to PDAC malignant cells (Fig. 5f,g). Consistent with pathway enrichments in P compared with PDAC co-cultures, these included ligands and receptors involved in PDGF (for example, *Pdgfd* and *Pdgfc*), TGF-β (for example, *Tgfbr1* and *Sparc*) and NOTCH (for example, *Notch2*) signalling.

We next asked whether P organoids from PSC co-cultures recapitulate ductal gene patterns from human pancreatitis tissues. We identified 4,553 orthologues of the P organoid markers from PSC co-cultures (‘P organoid orthologues from PSC co-cultures’) and compared them with human pancreatitis ductal orthologues. Overall, 39% (*n* = 1,777/4,593) of human pancreatitis ductal orthologues overlapped with P organoid orthologues from PSC co-cultures (Supplementary Table 8). Of these, 55% (*n* = 977/1,777) also overlapped with mouse pancreatitis ductal orthologues (for example, *SOX9*, *SOX4*, *DPP4*, *PDGFD*, *FGFR2* and *IL6ST*) (Supplementary Table 8). SOX9 marks ADM and is expressed in PDAC^36,37^, SOX4 maintains pancreatic epithelial progenitor identity^38^, while DPP4 remains less defined in diseased and malignant pancreas^39^. We validated higher SOX9, SOX4 and DPP4 protein levels in P compared with PDAC organoids (Fig. 5h). This cross-species, cross-model comparison also identified 800 human pancreatitis ductal orthologues shared with P organoid orthologues from PSC co-cultures but not with in vivo mouse pancreatitis ductal orthologues (for example, *STAT3*, *NFKB1* and *MMP24*) (Supplementary Table 8), suggesting that P organoid/PSC co-cultures complement mouse models for dissecting human pancreatitis ductal gene patterns.

We then asked whether PDAC organoids from PSC co-cultures recapitulate malignant cell profiles from PDAC tissues. Genes upregulated in PDAC compared with P organoid co-cultures (‘PDAC organoid markers from PSC co-cultures’; *n* = 5,047) significantly overlapped with mouse PDAC malignant markers (Fig. 5i,j and Supplementary Table 8). Orthologues of PDAC organoid markers from PSC co-cultures also overlapped with 46% (*n* = 798/1,751) of human PDAC malignant orthologues (Supplementary Table 8), of which 47% (*n* = 372/798) were also upregulated in mouse PDAC malignant orthologues. Shared orthologues included proliferative (for example, *TOP2A*) and metabolic (for example, *SLC2A1*) markers, as well as PDAC-enriched markers such as *GCNT3* and *MUC5AC*^35^. *CDKN2A*, which encodes human p16 and p14 through alternative reading frames and is frequently lost in PDAC^30^, was also upregulated in mouse and human PDAC malignant cells compared with pancreatitis ductal cells in vitro and in vivo (Fig. 5i,j and Supplementary Table 8). Separate transcript analysis showed that *Cdkn2a-202* (encoding p19, the mouse orthologue of p14), but not *Cdkn2a-201* (encoding p16), was upregulated in PDAC compared with P organoids, and protein analysis confirmed higher p19, but not p16, in PDAC organoids (Fig. 5k,l). To determine whether mutant KRAS or p53 drives p19 upregulation in PDAC, we compared p19 protein abundance across a panel of organoid models, including *Kras*^LSL-G12D/+^; *Pdx1*-Cre (KC) GEMM-derived pancreatic intraepithelial neoplasia organoids, and KPC GEMM-derived PDAC organoids that had not undergone p53 loss of heterozygosity (LOH; hereon, ‘KPC organoids’). Comparing N, KC, KPC and PDAC organoids showed that p19 increased modestly from N to KC organoids and was highest in KPC and PDAC organoids, suggesting an association between *Kras*/*Trp53* status and increased p19 in PDAC malignant cells relative to pancreatitis ductal cells (Extended Data Fig. 6h).

PSCs affected P organoid gene expression more than PDAC organoid gene expression (Fig. 5e). Whereas only 1,042 genes differed between PDAC organoid mono- and co-cultures, 3,389 differed between P mono- and co-cultures (Supplementary Table 11). To estimate how many P and PDAC organoid markers from PSC co-cultures were regulated by PSC-derived cues, we compared co-culture markers with genes upregulated in each organoid type in co-culture relative to monoculture. This identified 20% (*n* = 1,016/5,018) of P organoid markers from PSC co-cultures that were also induced by PSC-derived cues (for example, *Tgfbr1* and *Sparc*) (Fig. 5m and Extended Data Fig. 6i). By contrast, only 3% (*n* = 138/5,047) of PDAC organoid markers from PSC co-cultures were induced by PSC-derived cues, and most PDAC organoid markers from PSC co-cultures (for example, *Top2a* and *Dusp6*) were unchanged between mono- and co-cultures (Extended Data Fig. 6j,k).

Together, P and PDAC organoid/PSC co-cultures recapitulate in vivo gene expression patterns of mouse and human epithelial cells in pancreatitis and PDAC, and indicate that pancreatitis ductal cell transcriptomes are more strongly shaped by fibroblast signalling than PDAC malignant cell transcriptomes.

### Organoid/PSC co-cultures partially capture in vivo fibroblast differences in pancreatitis and PDAC

**Fig. 6:**
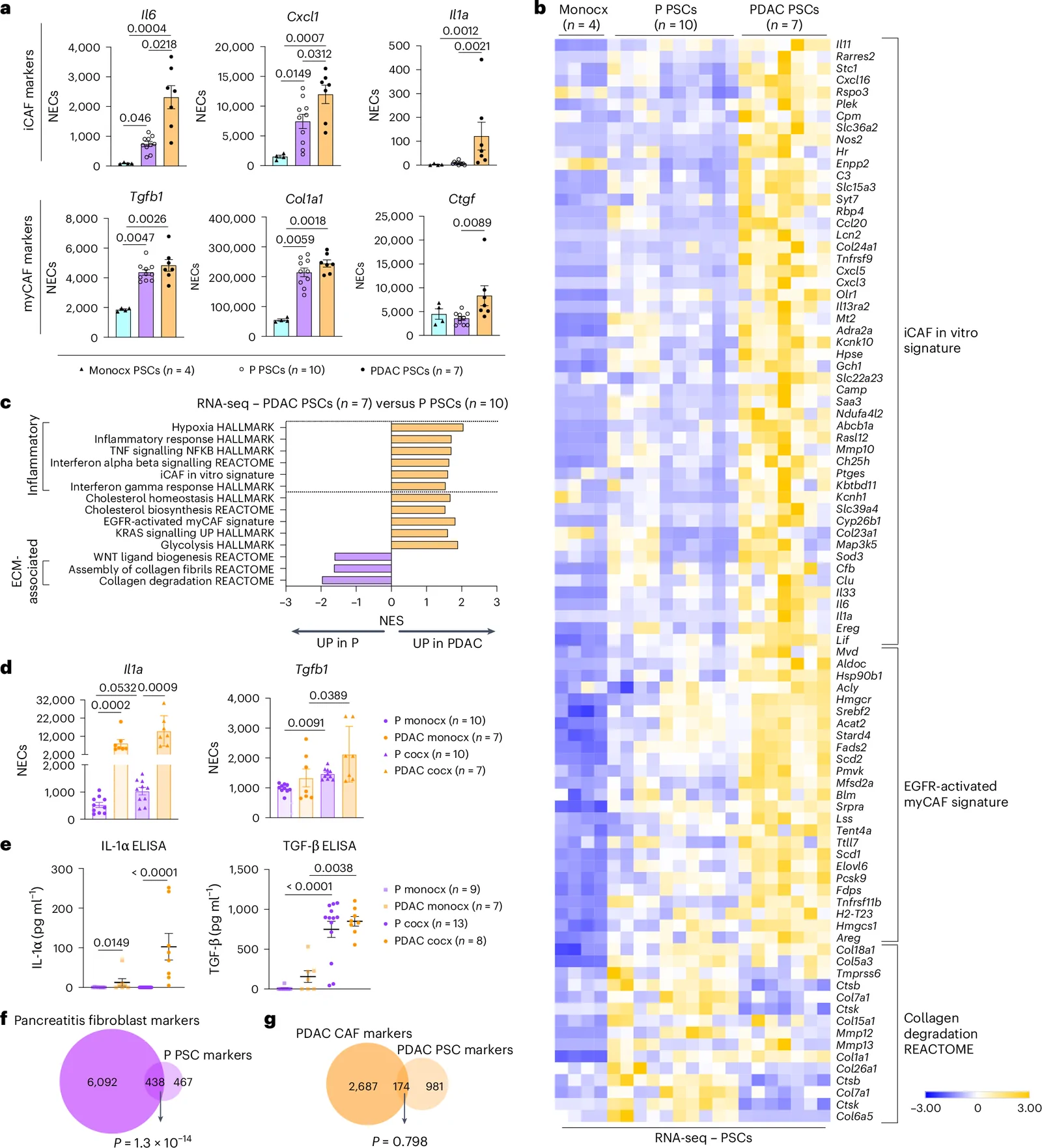
Organoid/PSC co-cultures only partially recapitulate the in vivo transcriptomic differences of pancreatitis and PDAC fibroblasts. **a**, The RNA-seq expression of iCAF (*Il1a*, *Il6* and *Cxcl1*) and myCAF (*Tgfb1*, *Col1a1* and *Ctgf*) markers in PSCs in monocx (that is, monocx PSCs; *n* = 4, *n* = 2 lines in two passages) and in PSCs in cocx with P (that is, P PSCs; *n* = 10 PSC/organoid pairs) or PDAC (that is, PDAC PSCs; *n* = 7 PSC/organoid pairs) organoids. Each data point corresponds to a biological replicate. Results show mean ± s.e.m. Adjusted *P* values from Kruskal–Wallis tests. **b**, A heat map showing the normalized counts from RNA-seq of monocx PSCs (*n* = 4, *n* = 2 lines in two passages), P PSCs (*n* = 10 PSC/organoid pairs) and PDAC PSCs (*n* = 7 PSC/organoid pairs). Selected genes and pathways upregulated or downregulated across conditions are shown. **c**, Significantly upregulated and downregulated pathways identified by GSEA of PDAC PSCs (*n* = 7 PSC/organoid pairs) compared with P PSCs (*n* = 10 PSC/organoid pairs), as assessed by RNA-seq. The iCAF in vitro signature is from Öhlund et al.^4^. The EGFR-activated myCAF signature is from Mucciolo et al.^7^. **d**, The RNA-seq expression of *Il1a* and *Tgfb1* in P monocx (*n* = 10, *n* = 5 lines in two passages), P cocx (*n* = 10 PSC/organoid pairs), PDAC monocx (*n* = 4 lines with three samples in two passages) or PDAC cocx (*n* = 7 PSC/organoid pairs). Each data point corresponds to a biological replicate. Results show means ± s.e.m. Adjusted *P* values are from Kruskal–Wallis tests. **e**, An ELISA of interleukin 1 alpha (IL-1α, left) and transforming growth factor beta (TGF-β, right) from media of P monocx (*n* = 9, *n* = 5 lines with four in two passages), PDAC monocx (*n* = 7, *n* = 4 lines with three in two passages), P cocx (*n* = 13 PSC/organoid pairs) and PDAC cocx (*n* = 8 PSC/organoid pairs). Results show means ± s.e.m. Adjusted *P* values are from Kruskal–Wallis tests. **f**, Venn diagrams of curated protein-coding genes significantly upregulated in P PSCs compared with PDAC PSCs (‘P PSC markers’), and mouse pancreatitis fibroblasts compared with PDAC CAFs in vivo (‘pancreatitis fibroblast markers’), as assessed by RNA-seq and snRNA-seq, respectively. The significance of the overlap between datasets was defined by hypergeometric tests (with a denominator of 18,000). **g**, Venn diagrams of curated protein-coding genes significantly upregulated in PDAC PSCs compared with P PSCs (‘PDAC PSC markers’), and mouse PDAC CAFs compared with mouse pancreatitis fibroblasts in vivo (‘PDAC CAF markers’), as assessed by RNA-seq and snRNA-seq, respectively. The significance of the overlap between datasets was defined by hypergeometric tests (with a denominator of 18,000).

**Extended Data Fig. 7:**
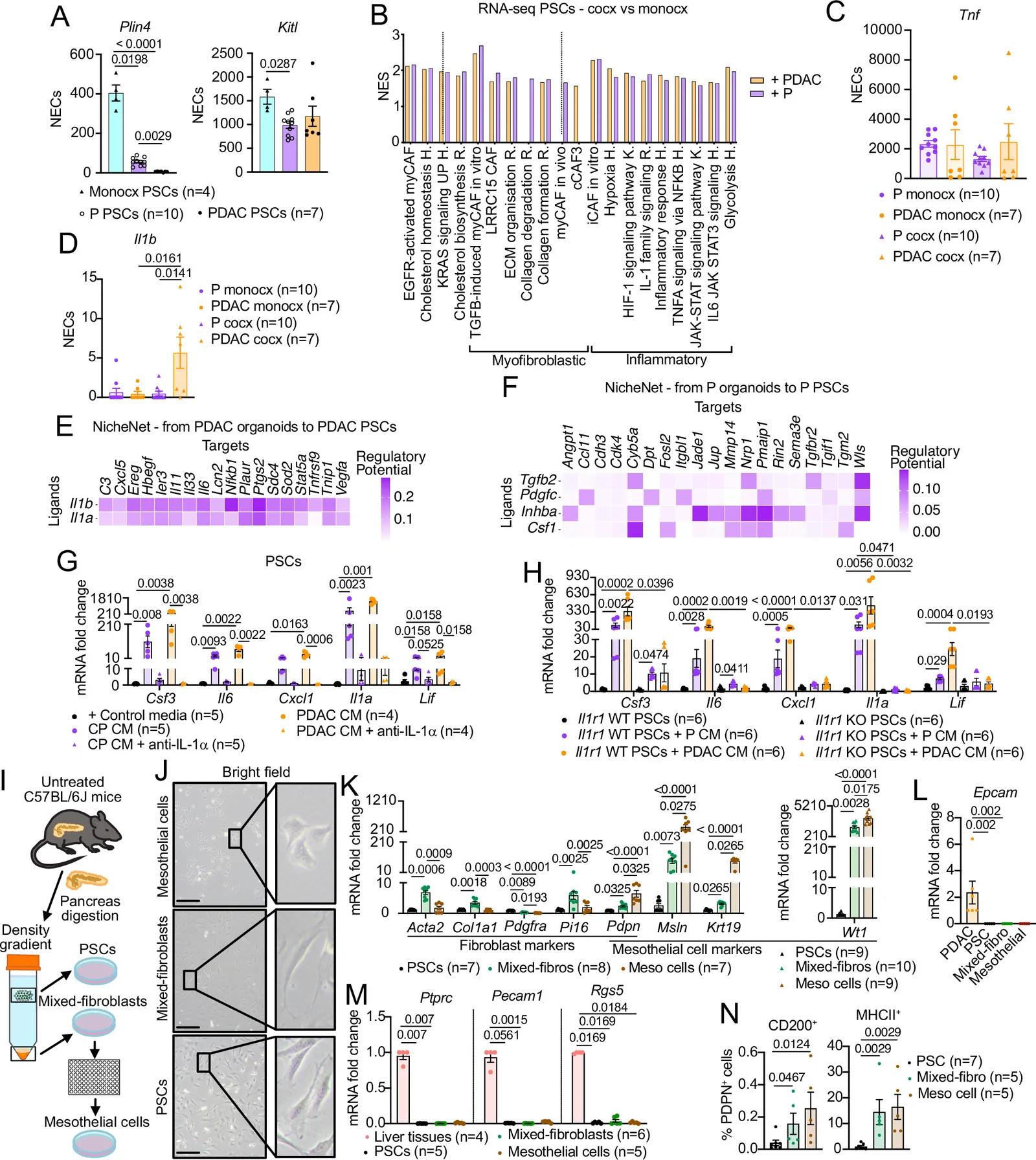
Organoid/PSC co-cultures partially recapitulate the *in vivo* transcriptome of pancreatitis and PDAC fibroblasts. (**A**) RNA-seq expression of quiescent fibroblast markers in monocx PSCs (n = 4, n = 2 lines in two passages) and co-cultures with P (n = 10 PSC/organoid pairs) or PDAC (n = 7 PSC/organoid pairs) organoids. Each data point corresponds to a biological replicate. Results show mean ± SEM. *P adj*, Kruskal-Wallis test. (**B**) Significantly upregulated pathways identified by GSEA of PSCs cultured with PDAC or P organoids compared to monocx PSCs, as assessed by RNA-seq. (**C-D**) RNA-seq expression of *Tnf* (**C**) or *Il1b* (**D**) in P monocx (n = 10, n = 5 lines in two passages), P cocx (n = 10 PSC/organoid pairs), PDAC monocx (n = 4 lines with three samples in two passages) or PDAC cocx (n = 7 PSC/organoid pairs). Each data point corresponds to a biological replicate. Results show mean ± SEM. *P adj*, Kruskal-Wallis test. (**E-F**) Ligand–target heatmaps show top selected ligands of (**E**) PDAC organoids inferred to regulate target genes in co-cultured PSCs, or of (**F**) P organoids inferred to regulate target genes in co-cultured PSCs, as assessed by NicheNet analysis. (**G-H**) qPCR analysis of *Csf3, Il6*, *Cxcl1*, *Il1a* and *Lif* (**G**) in PSCs cultured for 4 days in Matrigel with reduced media (n = 5, n = 3 lines with two in two passages) or with P (n = 5) or PDAC (n = 4) organoid-conditioned media (CM) in the presence of a neutralizing antibody targeting IL-1α or an IgG control, or (**H**) in *Il1r1* wild-type (WT) or knockout (KO) PSCs cultured for 4 days in Matrigel with reduced media (n = 6, n = 3 PSCs in two technical replicates) or with P (n = 6, n = 3 PSC/organoid pairs in two technical replicates) or PDAC (n = 6, n = 3 PSC/organoid pairs in two technical replicates) organoid–CM. Results show mean ± SEM. *P adj*, Kruskal-Wallis test. (**I**) Schematic illustrating how pancreatic stromal cells were generated. (**J**) Bright field images of murine PSCs, mixed-fibroblasts and mesothelial cells cultured in 2D. Scale bars = 100 μm. (**K**) Left: RNA expression levels of fibroblast (*Acta2*, *Col1a1*, *Pdgfra*, *Pi16, Pdpn*) and mesothelial cell (*Pdpn*, *Msln*, *Krt19*) markers in PSCs (n = 7, n = 3 lines with two in three passages), mixed-fibroblasts (n = 8, n = 4 lines with two in three passages) and mesothelial cells (n = 7, n = 3 lines with two in three passages) cultured in 2D. Right: RNA expression levels of the mesothelial cell marker *Wt1* in PSCs (n = 9, n = 3 lines with two in four passages), mixed-fibroblasts (n = 10, n = 4 lines with two in four passages) and mesothelial cells (n = 9, n = 3 lines with two in four passages) cultured in 2D. Results show mean ± SEM. *P adj*, Kruskal-Wallis test. (**L**) RNA expression levels of an epithelial cell marker in PSCs (n = 5, n = 3 lines with two in two passages), mixed-fibroblasts (n = 6, n = 4 lines with two in two passages) and mesothelial cells (n = 5, n = 3 lines with two in two passages) cultured in 2D, and in PDAC organoids (that is, PDAC, n = 6, n = 4 lines with two in two passages). Results show mean ± SEM. *P adj*, Kruskal-Wallis test. (**M**) RNA expression levels of immune cell (*Ptprc*), endothelial cell (*Pecam1*) and pericyte (*Rgs5*) markers in PSCs (n = 5, n = 3 lines with two in two passages), mixed-fibroblasts (that is, mixed-fibros, n = 6, n = 4 lines with two in two passages) and mesothelial cells (that is, mesothelials, n = 5, n = 3 lines with two in two passages) cultured in 2D, and in liver tissue (n = 4). Results show mean ± SEM. *P adj*, Kruskal-Wallis test. (**N**) Left: flow cytometry for the mesothelial cell marker CD200 for PSCs (n = 7, n = 3 lines with two in three passages), mixed-fibroblasts (n = 5, n = 3 lines with two in two passages) and mesothelial cells (n = 5, n = 3 lines with two in two passages) cultured in 2D. Right: flow cytometry for major histocompatibility complex class II (MHC-II) for the same cell lines cultured in 2D with 100 ng/mL recombinant mouse interferon gamma (IFNγ) for 48 h. Data are from three independent experiments. Results show mean ± SEM. *P adj*, Kruskal-Wallis test. Source numerical data are available.

We next asked how pancreatitis and PDAC epithelial cells differentially reprogramme fibroblasts by analysing PSCs from monocultures and from co-cultures with P or PDAC organoids. PSCs were activated in both co-culture settings relative to monoculture, with reduced quiescent fibroblast markers (*Plin4* and *Kitl*) and increased inflammatory (*Il6* and *Cxcl1*) and myofibroblastic (*Tgfb1* and *Col1a1*) markers^4,12,33,40^ (Fig. 6a and Extended Data Fig. 7a). Consistently, inflammatory and myofibroblastic signatures were enriched in both PSC co-cultures compared with PSC monocultures (Extended Data Fig. 7b and Supplementary Table 12). However, inflammatory markers (*Il1a*, *Il6* and *Cxcl1*) and pathways were higher in PSCs from PDAC co-cultures (Fig. 6a–c).

To identify mediators of this enhanced fibro-inflammatory phenotype, we examined known epithelial-derived ligands that induce inflammatory fibroblast and myofibroblast states in PDAC. Malignant cell-secreted TGF-β induces PDAC myCAF formation, whereas tumour necrosis factor (TNF, encoded by *Tnf*) and interleukin 1 alpha and beta (IL-1α and IL-1β) drive iCAF formation^7,33^. *Tnf* and *Tgfb1* expression, and secreted TGF-β protein levels, were comparable in P and PDAC organoid mono- and co-cultures (Fig. 6d,e and Extended Data Fig. 7c). By contrast, *Il1a* expression and secreted IL-1α protein levels were higher in PDAC organoid mono- and co-cultures, while *Il1b* increased only in PDAC co-cultures (Fig. 6d,e and Extended Data Fig. 7d). NicheNet analysis also inferred *Il1a* and *Il1b* as candidate mediators of PDAC organoid-to-PSC crosstalk (Extended Data Fig. 7e,f). We therefore tested whether IL-1 signalling contributes to the stronger inflammatory state of PSCs from PDAC co-cultures. Conditioned media from both P and PDAC organoids induced inflammatory markers (*Il1a*, *Cxcl1*, *Csf3*, *Lif* and *Il6*) in PSCs, with higher levels induced by PDAC organoids (Extended Data Fig. 7g). IL-1α neutralization attenuated this phenotype in both conditions, most strongly in the PDAC condition, and *Il1r1* knockout PSCs showed impaired inflammatory-marker induction compared with wild-type controls, with the greatest impairment in the PDAC condition (Extended Data Fig. 7g,h). Thus, elevated IL-1α production in PDAC, relative to P organoids, contributes to the enhanced inflammatory phenotype of co-cultured PSCs.

We next asked whether P or PDAC organoids induced PSC gene expression profiles resembling fibroblasts in pancreatitis and PDAC tissues. Genes upregulated in PSCs from P compared with PDAC co-cultures (‘P PSC markers’) significantly overlapped with mouse pancreatitis fibroblast markers, and orthologues of P PSC markers (‘P PSC orthologues’) overlapped with human pancreatitis fibroblast orthologues (Fig. 6f and Supplementary Table 8). By contrast, genes upregulated in PSCs from PDAC compared with P co-cultures (‘PDAC PSC markers’) did not significantly overlap with mouse PDAC CAF markers (Fig. 6g and Supplementary Table 8). Orthologues of PDAC PSC markers (‘PDAC PSC orthologues’) also did not overlap with human PDAC CAF orthologues (Supplementary Table 8). This limited overlap is consistent with the absence, in PDAC co-cultured PSCs, of pathways such as myofibroblastic signatures that were enriched in PDAC CAFs from mouse and human tissues relative to pancreatitis fibroblasts.

Thus, P and PDAC organoid/PSC co-cultures only partially recapitulate in vivo fibroblast gene expression differences in pancreatitis and PDAC, suggesting that additional stromal cell types may be needed to better capture fibroblast phenotypes seen in vivo.

### Cross-model analysis identifies epithelial markers of pancreatitis and PDAC

Previous work showed that PSCs generate only a subset of PDAC CAFs, with pancreatic fibroblasts and mesothelial cells also serving as CAF precursors^6,8,13,41–43^. We reasoned that pancreatitis fibroblasts may also arise from multiple origins. Indeed, mouse pancreatitis snRNA-seq revealed four fibroblast subsets, two of which expressed mesothelial markers (for example, *Wt1*) (Extended Data Fig. 3i). We therefore optimized organoid co-culture models to better mimic in vivo fibroblast heterogeneity in pancreatitis and PDAC.

During PSC line generation, we also established mixed-fibroblast and mesothelial cell lines from normal pancreata of untreated C57BL/6J mice (Extended Data Fig. 7i). In monolayer culture, mixed-fibroblasts resembled PSCs, whereas mesothelial cells showed cobblestone-like morphology^44^ (Extended Data Fig. 7j). Mixed-fibroblast and mesothelial lines expressed fibroblastic (*Acta2*, *Col1a1*, *Pdgfra* and *Pi16*) and mesothelial (*Msln*, *Wt1* and *Krt19*) markers, respectively, and lacked immune, endothelial, pericyte and epithelial markers (Extended Data Fig. 7k–m). Both lines had higher CD200 than PSCs, especially mesothelial cells, and showed higher major histocompatibility complex type II (MHCII) after interferon-γ (IFNγ) stimulation (Extended Data Fig. 7n). Together, these data suggest that mixed-fibroblast lines comprise fibroblasts, mesothelial cells and, potentially, PSCs.

**Fig. 7:**
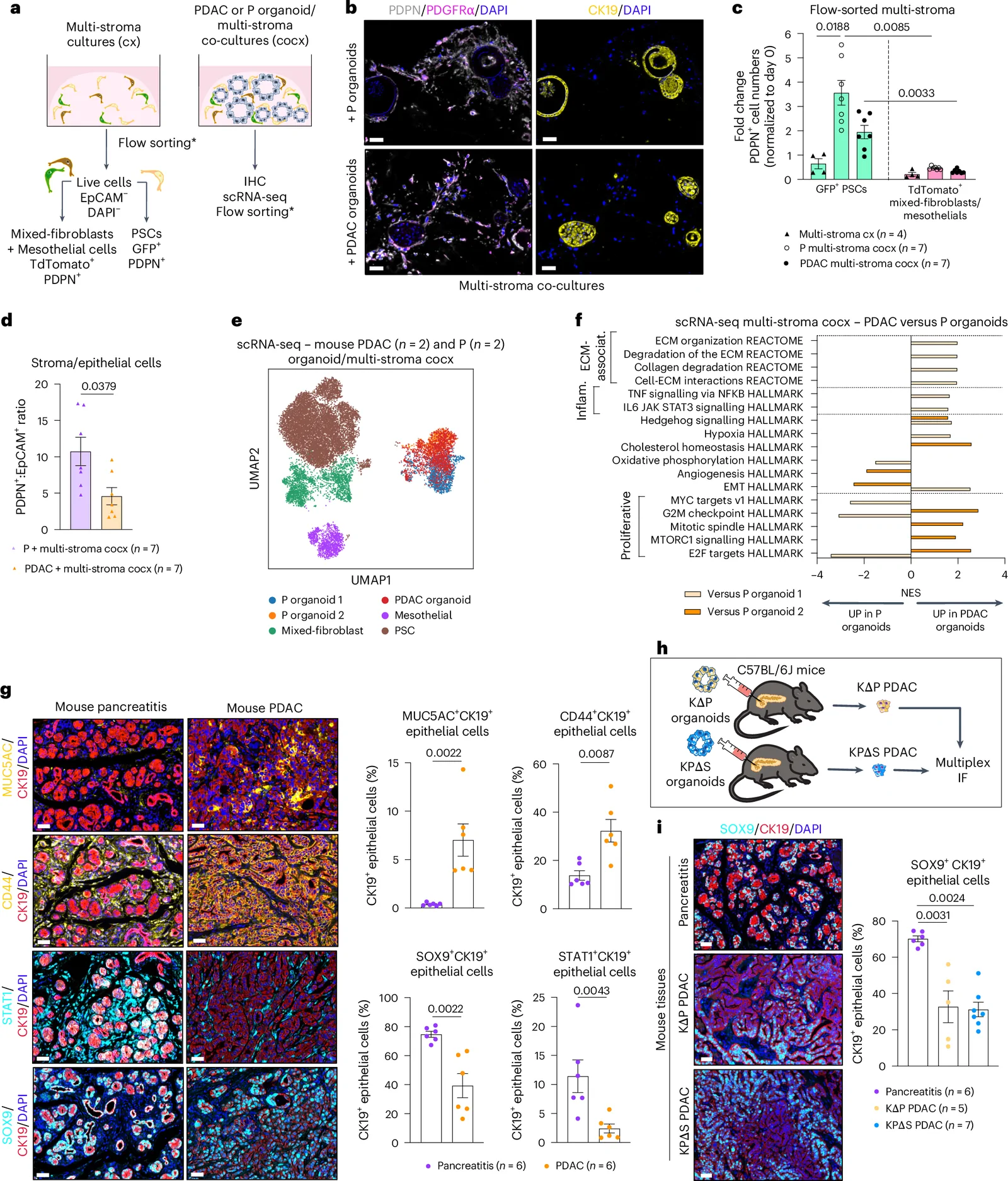
Cross-model analysis pinpoints epithelial cell markers of pancreatitis and PDAC. **a**, A schematic of analyses of P or PDAC organoids co-cultured with PSCs, mixed-fibroblasts and mesothelial cells (that is, P or PDAC organoid/multi-stroma cocx) in RM (right), or PSCs, mixed-fibroblasts and mesothelial cells cultured without organoids (that is, multi-stroma cultures, cx) in RM (left). **b**, Representative multiplex IF images of PDPN and PDGFRα (left) or CK19 (right) stains of P or PDAC organoid/multi-stroma co-cultures after 7 days in culture. Scale bars, 50 μm. **c**, The fold change of GFP-positive PSCs (GFP^+^PDPN^+^EpCAM^−^) and tdTomato-positive mixed-fibroblasts and mesothelial cells (tdTomato^+^PDPN^+^EpCAM^−^) flow-sorted from multi-stroma cx (*n* = 4, *n* = 2 PSC/mixed-fibroblast/mesothelial combination in two passages) or from P (*n* = 7 multi-stroma/organoid pairs) or PDAC (*n* = 7 multi-stroma/organoid pairs) multi-stroma cocx after 3.5 days in culture relative to numbers of respective GFP^+^ or tdTomato^+^ cells plated at day 0. Each data point corresponds to a biological replicate. Results show means ± s.e.m. Adjusted *P* values from Kruskal–Wallis tests. **d**, The multi-stroma (PDPN^+^EpCAM^−^)/epithelial cell (EpCAM^+^PDPN^−^) ratio as assessed by flow sorting of P (*n* = 7 multi-stroma/organoid pairs) or PDAC (*n* = 7 multi-stroma/organoid pairs) multi-stroma cocx after 3.5 days in culture. Results show means ± s.e.m. *P* value from Mann–Whitney tests. **e**, A UMAP plot of cell subsets in the combined scRNA−seq dataset of PDAC (*n* = 2) and P (*n* = 2) organoid/multi-stroma cocx after 3.5 days in culture. Different cell subsets are colour coded. **f**, Significantly upregulated and downregulated pathways identified by GSEA of PDAC organoids compared with P organoids 1 or P organoids 2, as assessed by MAST from the scRNA−seq dataset of organoid/multi-stroma cocx. **g**, Left: representative multiplex IF images of mucin MUC5AC (yellow), CK19 (red) and DAPI (blue); CD44 (yellow), CK19 (red) and DAPI (blue); SOX9 (cyan), CK19 (red) and DAPI (blue); or STAT1 (cyan), CK19 (red) and DAPI (blue) in mouse pancreatitis and PDAC tissues. Scale bars, 50 μm. Right: the quantification of MUC5AC^+^CK19^+^DAPI^+^ or cytoplasmic-CD44^+^CK19^+^DAPI^+^ or SOX9^+^CK19^+^DAPI^+^ or STAT1^+^CK19^+^DAPI^+^αSMA^−^PDGFRα^−^ cells relative to DAPI^+^CK19^+^αSMA^−^PDGFRα^−^ epithelial cells in mouse pancreatitis (*n* = 6) and PDAC (*n* = 6) tissues. Results show means ± s.e.m. *P* values from Mann–Whitney tests. Pancreatitis samples derive from three independent treatment cohorts. **h**, A schematic of models and techniques used for the analysis of mouse PDAC tissues derived from orthotopic transplantation models of KRAS^G12D^, p53-null organoids (that is, KΔP PDAC)^29^ or *Smad4* KO KRAS^G12D^, p53^R172H^ organoids^28^ (that is, KPΔS PDAC; as in Fig. 3). **i**, Representative multiplex IF images of SOX9 (cyan), CK19 (red) and DAPI (blue) in mouse pancreatitis, KΔP PDAC and KPΔS PDAC tissues. Scale bars, 50 μm. Quantification of SOX9^+^CK19^+^αSMA^−^PDGFRα^−^DAPI^+^ cells relative to DAPI^+^CK19^+^αSMA^−^PDGFRα^−^ epithelial cells in mouse pancreatitis (*n* = 6), KΔP PDAC (*n* = 5) and KPΔS PDAC (*n* = 7) tissues, as assessed by multiplex IF analysis. Pancreatitis samples derive from three independent treatment cohorts. Results show means ± s.e.m. Adjusted *P* values from Kruskal–Wallis tests. Inflam., inflammatory; ECM-associat., ECM-associated.

**Extended Data Fig. 8:**
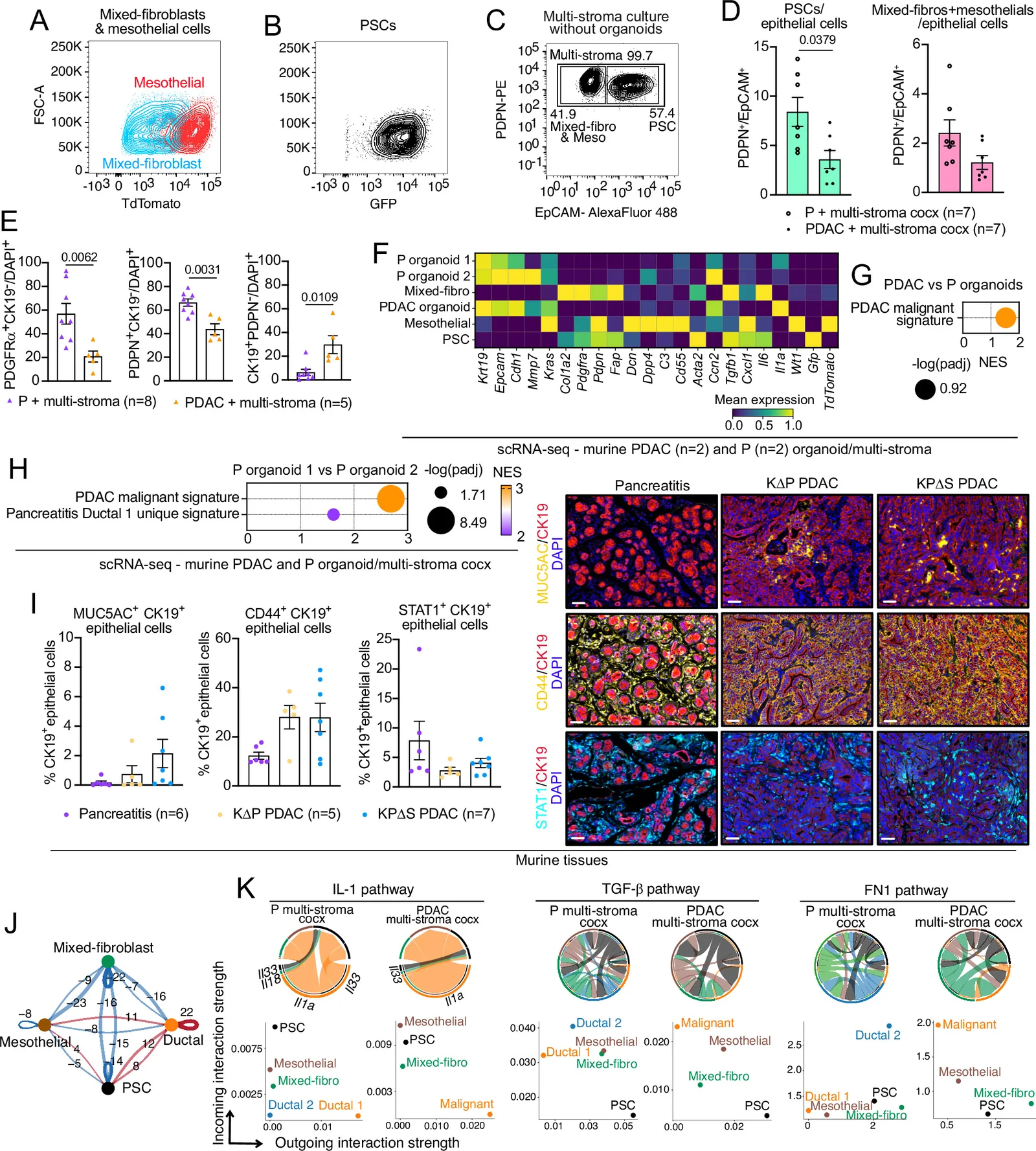
Cross-model analysis pinpoints epithelial cell markers of pancreatitis and PDAC. (**A-B**) Representative flow cytometry plots of TdTomato^+^ mixed-fibroblasts and mesothelial cells (**A**) and green fluorescent protein-positive (GFP^+^) PSCs (**B**) cultured in 2D. (**C**) Flow cytometry of multi-stroma culture (that is, PSCs, mesothelial cells and mixed-fibroblasts, without organoids). PSCs are GFP^+^ and mixed-fibroblasts and mesothelial cells are TdTomato^+^. (**D**) Left: PSC (GFP^+^PDPN^+^EpCAM^−^)/epithelial cell (EpCAM^+^PDPN^−^) ratio as assessed by flow sorting of P (n = 7 multi-stroma/organoid pairs) or PDAC (n = 7 multi-stroma/organoid pairs) multi-stroma cocx after 3.5 days in culture. Results show mean ± SEM. *P*, Mann-Whitney test. Right: Mixed-fibroblasts and mesothelial cells (TdTomato^+^PDPN^+^EpCAM^−^)/epithelial cell (EpCAM^+^PDPN^−^) ratio as assessed by flow sorting of P (n = 7 multi-stroma/organoid pairs) or PDAC (n = 7 multi-stroma/organoid pairs) multi-stroma cocx after 3.5 days in culture. Results show mean ± SEM. No significance, as assessed by Mann-Whitney test. (**E**) Quantification of PDGFRα^+^CK19^−^ (left), PDPN^+^CK19^−^ (middle) and CK19^+^PDPN^−^ (right) DAPI^+^ cells in P (n = 8 multi-stroma/organoid pairs) or PDAC (n = 5 multi-stroma/organoid pairs) organoid/multi-stroma co-cultures, as assessed by multiplex IF. Results show mean ± SEM. *P*, Mann-Whitney test. (**F**) Heatmap of scaled expression of cell subset specific markers of murine P and PDAC organoid/multi-stroma co-cultures as assessed by scRNA−seq. Data are scaled such that the cluster with the lowest average expression = 0 and the highest = 1 for each gene. (**G**) GSEA of the PDAC malignant signature in PDAC organoids compared to P organoids from organoid/multi-stroma co-cultures. The PDAC malignant cell signature was obtained by comparing murine PDAC malignant cells to pancreatitis ductal cells from the snRNA−seq dataset. (**H**) GSEA of the PDAC malignant signature and of the pancreatitis Ductal 1-unique signature in P organoid 1 compared to P organoid 2 cells from organoid/multi-stroma co-cultures. The pancreatitis Ductal 1-unique signature was obtained by comparing pancreatitis Ductal 1 to Ductal 2 cells from the snRNA−seq dataset of murine tissues. (**I**) Left: quantifications of MUC5AC^+^CK19^+^, cytoplasmic-CD44^+^CK19^+^ and STAT1^+^CK19^+^ DAPI^+^αSMA^−^PDGFRα^−^ cells relative to DAPI^+^CK19^+^αSMA-PDGFRα^−^ epithelial cells in murine pancreatitis (n = 6), KΔP PDAC (n = 5) and KPΔS PDAC (n = 7) tissues, as assessed by multiplex IF analysis. Results show mean ± SEM. *P adj*, Kruskal-Wallis test. Pancreatitis samples derive from three independent treatment cohorts. Right: representative multiplex IF images of mucin MUC5AC (yellow), CK19 (red) and DAPI (nuclear stain, blue), CD44 (yellow), CK19 (red) and DAPI (blue) and STAT1 (cyan), CK19 (red) and DAPI (blue) in murine pancreatitis, KΔP PDAC and KPΔS PDAC tissues. Scale bars = 50 μm. (**J**) Visualization of inferred cell-cell communication using CellChat showing the difference in connections between cell types in PDAC organoid/multi-stroma co-cultures relative to P organoid/multi-stroma co-cultures, as assessed by scRNA-seq. Blue indicates connections that are lost in PDAC. Red indicates connections that are gained in PDAC. The thickness of the connection reflects the number of interactions. (**K**) Top panels: selected IL-1, TGF-β and fibronectin/FN1 pathways with different connections in PDAC organoid/multi-stroma co-cultures and P organoid/multi-stroma co-cultures, as assessed by CellChat analysis. Bottom panels: Scatter plots showing the cell types most involved in outgoing and incoming signalling. Source numerical data are available.

To model epithelial crosstalk with heterogeneous stroma, we co-cultured P or PDAC organoids with green fluorescent protein (GFP)^+^ PSCs, TdTomato^+^ mixed-fibroblasts and TdTomato^+^ mesothelial cells, hereafter ‘multi-stroma’ co-cultures (Fig. 7a,b and Extended Data Fig. 8a–c). PSC numbers significantly increased in P, but not PDAC, multi-stroma co-cultures relative to multi-stroma cultures (that is, stromal cells cultured without organoids), indicating that pancreatitis epithelial cells promote PSC expansion more effectively than PDAC malignant cells even in complex stroma (Fig. 7c). PSCs also outnumbered mixed-fibroblasts and mesothelial cells in both co-cultures, and P co-cultures had higher stroma-to-epithelial ratios than PDAC co-cultures, driven mainly by PSC expansion (Fig. 7c,d and Extended Data Fig. 8d,e).

We next assessed how multi-stroma shaped P and PDAC organoid transcriptomes by scRNA-seq of co-cultures containing GFP^+^ PSCs, TdTomato^+^ mixed-fibroblasts, TdTomato^+^ mesothelial cells and P or PDAC organoids (Fig. 7e and Extended Data Fig. 8f). Consistent with the epithelial heterogeneity observed in mouse tissues, we identified two P organoid clusters (‘P organoid 1’ and ‘P organoid 2’) and one PDAC organoid cluster (Fig. 7e). The PDAC malignant signature was higher in PDAC than P organoids, and higher in P organoid 1 than P organoid 2 cells, while pancreatitis Ductal 1 markers were enriched in P organoid 1 compared with P organoid 2 cells (Extended Data Fig. 8g,h). Proliferation-associated pathways were upregulated in PDAC organoids relative to P organoid 2 cells, whereas ECM-associated and inflammatory signatures were enriched relative to P organoid 1 cells (Fig. 7f and Supplementary Table 13). Thus, while inflammatory signatures are higher in P than PDAC organoids in monoculture, stromal co-culture reverses this pattern, inducing PDAC organoid inflammatory programmes that mirror those enriched in PDAC relative to pancreatitis epithelial cells in tissues. This suggests that stromal input is required for in vivo-like inflammatory pathway activation in PDAC malignant cells.

We then compared P and PDAC organoid markers from multi-stroma co-cultures with epithelial markers from tissues using our cross-model and cross-species strategy. P organoid markers from multi-stroma co-cultures overlapped with mouse pancreatitis Ductal 1 and Ductal 2 markers, whereas PDAC organoid markers from multi-stroma co-cultures overlapped with mouse PDAC malignant markers (Supplementary Table 8). P and PDAC organoid orthologues from multi-stroma co-cultures also overlapped with human pancreatitis ductal and PDAC malignant orthologues, respectively (Supplementary Table 8).

Because PSC and multi-stroma co-cultures both captured in vivo epithelial profiles, we integrated transcriptomic datasets from mouse models and both co-culture systems to define shared epithelial markers. This identified 177 pancreatitis ductal markers (for example, *Sox9* and *Stat1*) and 254 PDAC malignant markers (for example, *Muc5ac* and *Cd44*) (Supplementary Table 14). Multiplex IF confirmed higher MUC5AC^+^CK19^+^ and CD44^+^CK19^+^ epithelial cells in mouse PDAC than pancreatitis tissues, whereas SOX9^+^CK19^+^ and STAT1^+^CK19^+^ epithelial cells were enriched in pancreatitis (Fig. 7g). Because PDAC genetics can alter malignant cell features^28,29^, we tested whether marker differences between pancreatitis and KRAS^G12D^ p53-mutant (KP) PDAC were maintained in other genetic contexts. We analysed orthotopic KRAS^G12D^ p53-null (KΔP) or SMAD4-deficient KP (KPΔS) PDAC models (Fig. 7h). MUC5AC^+^CK19^+^ and CD44^+^CK19^+^ epithelial populations were also higher, though less markedly, in KΔP and KPΔS PDACs than pancreatitis (Extended Data Fig. 8i). STAT1^+^CK19^+^ cells were moderately increased in pancreatitis compared with these PDACs, while SOX9^+^CK19^+^ cells were significantly enriched in pancreatitis (Fig. 7i and Extended Data Fig. 8i). Thus, PDAC malignant cells across genetic contexts share features that distinguish them from inflamed non-malignant epithelial cells, while retaining genotype-specific profiles.

Altogether, combining in vitro and in vivo models enables identification of epithelial markers differentially enriched in pancreatitis and PDAC tissues.

### Multi-stromal co-cultures better mimic fibroblast heterogeneity in pancreatitis and PDAC

**Fig. 8:**
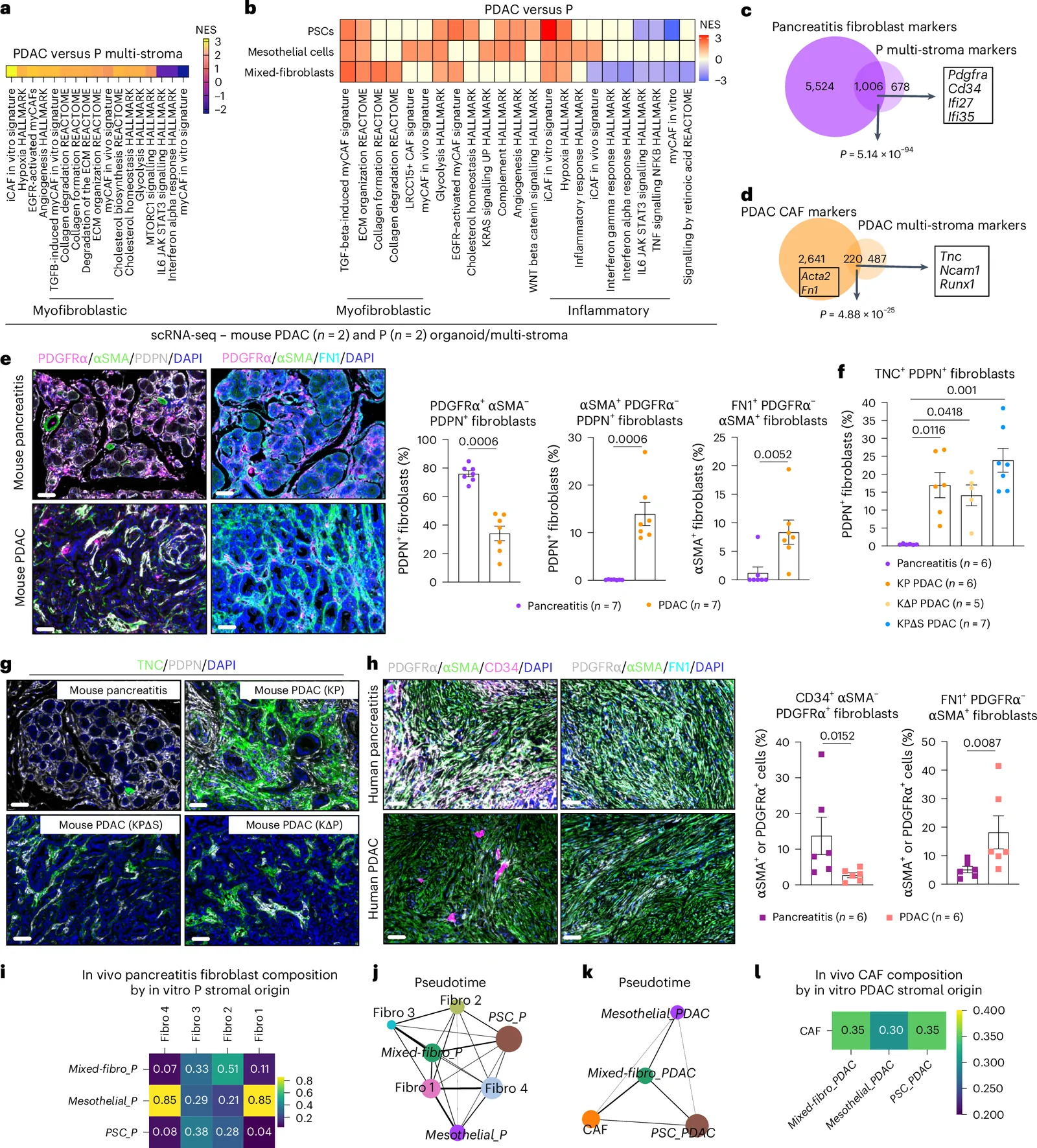
Multi-stromal co-cultures better mimic the fibroblast heterogeneity of pancreatitis and PDAC. **a**, Significantly upregulated and downregulated pathways identified by GSEA of multi-stroma cultured with PDAC organoids compared with multi-stroma cultured with P organoids, as assessed by MAST from the scRNA-seq dataset of organoid/multi-stroma cocx cultured with PDAC (PDAC multi-stroma, *n* = 2) or P (P multi-stroma, *n* = 2) organoids. The in vivo myCAF signature is from Elyada et al. (mouse scRNA-seq dataset)^5^. iCAF and myCAF in vitro signatures are from Öhlund et al.^4^. TGF-β-induced and EGFR-activated myCAF signatures are from Mucciolo et al.^7^. **b**, Significantly upregulated and downregulated pathways identified by GSEA of PSCs, mixed-fibroblasts or mesothelial cells cultured with PDAC organoids compared with these cells cultured with P organoids, as assessed by MAST from the scRNA-seq dataset of PDAC (*n* = 2) or P (*n* = 2) organoid/multi-stroma co-cultures. The LRRC15^+^ CAF signature is from Dominguez et al.^8^. **c**,**d**, Venn diagrams of curated protein-coding genes significantly upregulated in P multi-stroma compared with PDAC multi-stroma (‘P multi-stroma markers’), as assessed by scRNA-seq, and pancreatitis fibroblast markers (**c**) or in PDAC multi-stroma compared with P multi-stroma (‘PDAC multi-stroma markers’), as assessed by scRNA-seq, and PDAC CAF markers (**d**). The significance of the overlap between datasets was defined by hypergeometric tests (with a denominator of 18,000). **e**, Left: representative multiplex IF images of PDPN (white), αSMA (green), PDGFRα (pink) and DAPI (blue), or FN1 (cyan), αSMA (green), PDGFRα (pink) and DAPI (blue) in mouse pancreatitis and PDAC tissues. Scale bars, 50 μm. Right: the quantification of PDGFRα^+^αSMA^−^PDPN^+^ (left) and αSMA^+^PDGFRα^−^PDPN^+^ (middle) CK19^−^DAPI^+^ cells relative to PDPN^+^CK19^−^DAPI^+^ fibroblasts, and of FN1^+^PDGFRα^−^αSMA^+^DAPI^+^CK19^−^ cells relative to αSMA^+^CK19^−^DAPI^+^ fibroblasts (right) in mouse pancreatitis (*n* = 7) and PDAC (*n* = 7) tissues. Results show means ± s.e.m. *P* values from Mann–Whitney tests. Pancreatitis samples derive from two independent treatment cohorts. **f**, The quantification of TNC^+^PDPN^+^DAPI^+^CK19^−^ fibroblasts relative to PDPN^+^DAPI^+^CK19^−^ fibroblasts in mouse pancreatitis (*n* = 6), KP PDAC (*n* = 6), KΔP PDAC (*n* = 5) and KPΔS PDAC (*n* = 7) tissues, as assessed by multiplex IF analysis. Results show means ± s.e.m. Adjusted *P* values from Kruskal–Wallis tests. Pancreatitis samples derive from three independent treatment cohorts. **g**, Representative multiplex IF images of TNC (green), PDPN (white) and DAPI (blue) in mouse pancreatitis, KP PDAC, KΔP PDAC and KPΔS PDAC tissues. Scale bars, 50 μm. **h**, Left: representative multiplex IF images of αSMA (green), PDGFRα (white), CD34 (pink) and DAPI (blue), or αSMA (green), PDGFRα (white), FN1 (cyan) and DAPI (blue) in human PDAC and adjacent pancreata with chronic pancreatitis tissues. Scale bars, 50 μm. Right: the quantification of CD34^+^αSMA^−^PDGFRα^+^DAPI^+^CK19^−^ cells or of FN1^+^PDGFRα^−^αSMA^+^DAPI^+^CK19^−^ cells relative to αSMA^+^ and/or PDGFRα^+^ DAPI^+^CK19^−^ fibroblasts in human PDAC (*n* = 6) and adjacent pancreata with chronic pancreatitis (*n* = 6) tissues. Results show means ± s.e.m. *P* values from Mann–Whitney tests. **i**, A heat map showing the fractional contribution of in vitro scRNA-seq stromal cells from P organoid/multi-stroma cocx to in vivo snRNA-seq pancreatitis fibroblast subtypes, derived from averaged scANVI soft label probabilities. **j**,**k**, PAGA illustrating the inferred pseudotemporal trajectory among in vitro P stromal cells and in vivo pancreatitis fibroblast subtypes (**j**) or among in vitro PDAC stromal cells and in vivo PDAC CAFs (**k**). Nodes (that is, circles) represent cell subsets, and their size is scaled to the number of cells. Edges (that is, lines) represent the strength of connectivity (that is, thickness). **l**, A heat map showing the fractional contribution of in vitro scRNA-seq stromal cells from PDAC organoid/multi-stroma cocx to in vivo snRNA-seq PDAC CAFs, derived from averaged scANVI soft label probabilities.

After validating that P and PDAC organoid/multi-stroma co-cultures recapitulate in vivo epithelial features, we asked whether they better mirror fibroblast transcriptomes than PSC co-cultures. Using *Gfp*, *TdTomato*, and fibroblast or mesothelial markers, we identified PSCs, mixed-fibroblasts and mesothelial cells in the scRNA-seq dataset (Extended Data Fig. 8f). We then compared multi-stroma co-cultured with PDAC organoids (‘PDAC multi-stroma’) to multi-stroma co-cultured with P organoids (‘P multi-stroma’). Myofibroblastic signatures were enriched in PDAC multi-stroma, as seen in mouse and human PDAC CAFs in vivo, but not in PSCs cultured with PDAC organoids (Fig. 8a and Supplementary Table 13).

We next asked whether pancreatitis and PDAC epithelial cells differentially reprogramme stromal cells of distinct origins. PDAC organoids induced myofibroblastic and inflammatory signatures across PSCs, mixed-fibroblasts and mesothelial cells (Fig. 8b). However, cell type-specific responses were evident. Mixed-fibroblasts and PSCs, but not mesothelial cells, downregulated additional inflammatory pathways when cultured with PDAC versus P organoids (Fig. 8b). Mixed-fibroblasts, but not PSCs or mesothelial cells, also downregulated interferon responses and upregulated collagen-associated pathways when cultured with PDAC versus P organoids. These mixed-fibroblast-specific changes were also seen in PDAC CAFs versus pancreatitis fibroblasts from mouse tissues, suggesting that mixed-fibroblasts contribute to in vivo gene signatures.

CellChat analysis suggested that PDAC malignant cells communicate more with PSCs, but less with mixed-fibroblasts and mesothelial cells, than P ductal cells (Extended Data Fig. 8j). It also revealed changes in TGF-β, FN1 and IL-1 pathways (Extended Data Fig. 8k). Within the IL-1 pathway, *Il1a* was inferred as the dominant malignant-to-stromal ligand in PDAC organoid/multi-stroma co-cultures, supporting our organoid monoculture and organoid/PSC co-culture findings.

**Extended Data Fig. 9:**
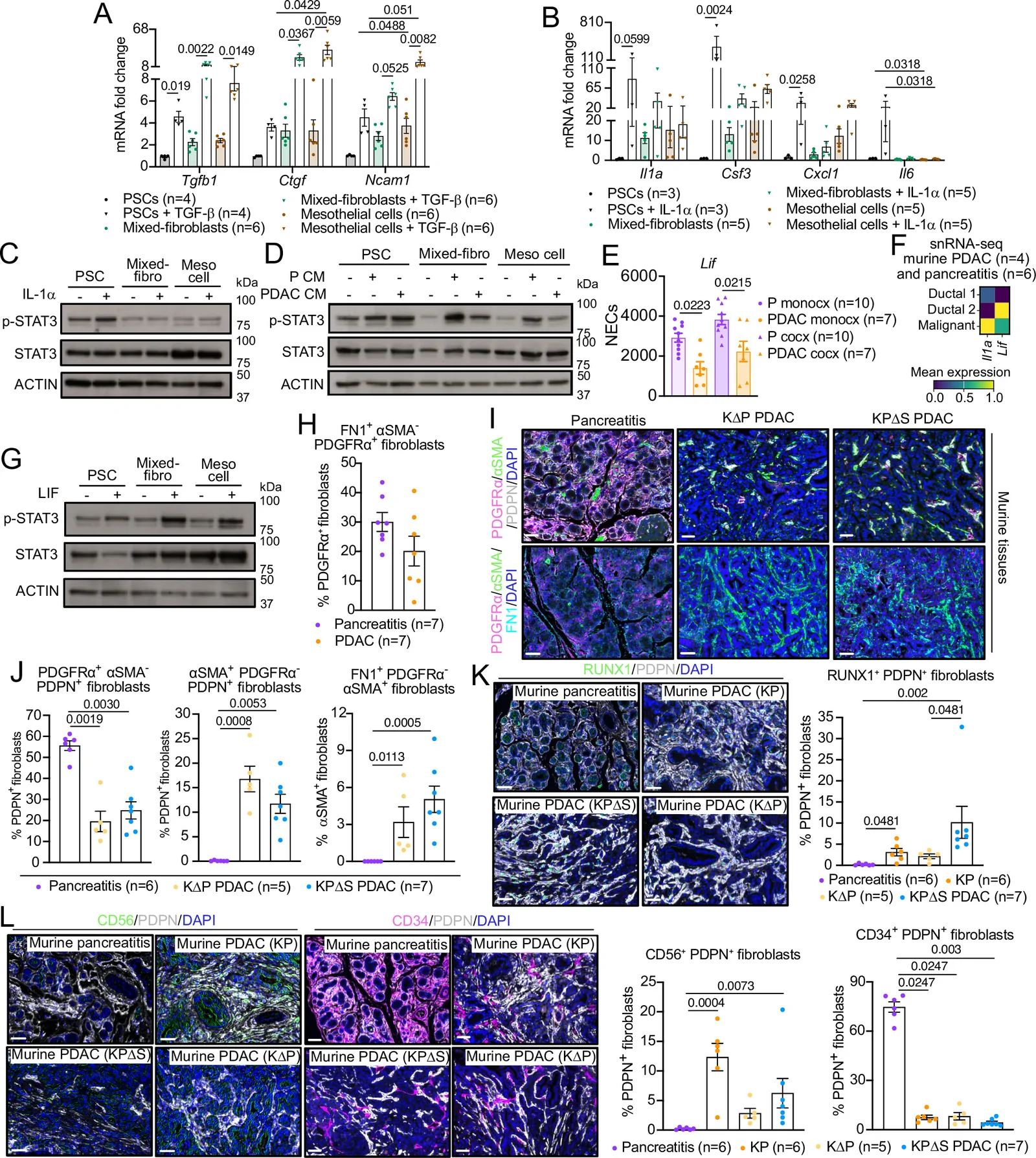
Multi-stromal/organoid co-cultures better mimic fibroblast heterogeneity of pancreatitis and PDAC. (**A-B**) qPCR analysis of (**A**) *Tgfb1, Ctgf* and *Ncam1* in PSCs (n = 4, n = 3 lines with one from two passages), mixed-fibroblasts (n = 6, n = 4 lines with two from two passages) and mesothelial cells (n = 6, n = 3 lines in two passages) cultured in Matrigel with reduced media with or without 20 ng/mL TGF-β for 4 days, or (**B**) *Il1a*, *Csf3, Cxcl1* and *Il6* in PSCs (n = 3), mixed-fibroblasts (n = 5, n = 3 lines with two in two passages) and mesothelial cells (n = 5, n = 3 lines with two in two passages) cultured in Matrigel with reduced media with or without 1-2 ng/mL IL-1α for 4 days. Results show mean ± SEM. *P adj*, Kruskal-Wallis test. (**C**) Western blot of p-STAT3 and STAT3 in PSCs, mixed-fibroblasts and mesothelial cells cultured in Matrigel with reduced media with or without 1 ng/mL IL-1α for 4 days. ACTIN, loading control. The samples were obtained and processed simultaneously. Data shown are representative of two experimental replicates (each one with different cell lines). (**D**) Western blot of p-STAT3 and STAT3 in PSCs, mixed-fibroblasts and mesothelial cells cultured for 4 days in Matrigel with reduced media, P or PDAC organoid-CM for 4 days. ACTIN, loading control. The samples were from two experiments and processed simultaneously. Data shown are representative of three experimental replicates (each one with different cell lines). (**E**) RNA-seq expression of *Lif* in P monocx (n = 10, n = 5 lines in two passages), P cocx (n = 10 PSC/organoid pairs), PDAC monocx (n = 4 lines with three samples in two passages) or PDAC cocx (n = 7 PSC/organoid pairs). Each data point represents a biological replicate. Results show mean ± SEM. *P adj*, Kruskal-Wallis test. (**F**) Heatmap of scaled expression of *Lif* in murine pancreatitis and PDAC ductal clusters. Data are scaled such that the cluster with the lowest average expression = 0 and the highest = 1 for each gene. (**G**) Western blot of p-STAT3 and STAT3 in PSCs, mixed-fibroblasts and mesothelial cells cultured for 4 days in Matrigel with reduced media with or without 5 ng/mL LIF. ACTIN, loading control. The samples were obtained and processed simultaneously. Data shown are representative of two experimental replicates (each one with different cell lines). (**H**) Quantification of FN1^+^αSMA^−^PDGFRα^+^DAPI^+^CK19^−^ cells relative to PDGFRα^+^CK19^−^DAPI^+^ fibroblasts in murine pancreatitis (n = 7) and PDAC (n = 7), as assessed by multiplex IF analysis. Results show mean ± SEM. No significance observed using Mann-Whitney test. Pancreatitis samples derive from two treatment cohorts. (**I**) Representative multiplex IF images of PDPN (white), αSMA (green), PDGFRα (pink) and DAPI (blue), or of FN1 (cyan), αSMA (green), PDGFRα (pink) and DAPI (blue), in murine pancreatitis, and KΔP, KPΔS PDACs. Scale bars = 50 μm. (**J**) Quantification of PDGFRα^+^αSMA^−^ CK19^−^PDPN^+^DAPI^+^ and αSMA^+^PDGFRα^−^CK19^−^PDPN^+^DAPI^+^ cells relative to PDPN^+^CK19^−^DAPI^+^ fibroblasts and of FN1^+^αSMA^+^PDGFRα^−^DAPI^+^CK19^−^ cells relative to αSMA^+^CK19^−^DAPI^+^ fibroblasts in murine pancreatitis (n = 6), KΔP PDAC (n = 5) and KPΔS PDAC (n = 7), as assessed by multiplex IF analysis. Results show mean ± SEM. *P adj*, Kruskal-Wallis test. Pancreatitis samples derive from three treatment cohorts. (**K**) Representative multiplex IF images of PDPN (white), RUNX1 (green) and DAPI (blue) in murine pancreatitis, PDAC from KPC GEMMs (KP PDAC), KΔP PDAC and KPΔS PDAC. Scale bars = 50 μm. Quantification of nuclear-RUNX1^+^PDPN^+^DAPI^+^CK19^−^ cells relative to PDPN^+^CK19^−^DAPI^+^ fibroblasts in murine pancreatitis (n = 6), KP PDAC (n = 6), KΔP PDAC (n = 5) and KPΔS PDAC (n = 7). Results show mean ± SEM. *P adj*, Kruskal-Wallis test. Pancreatitis samples derive from three treatment cohorts. (**L**) Representative multiplex IF images of PDPN (white), CD56 (green) and DAPI (blue) or of PDPN (white), CD34 (pink) and DAPI (blue) in murine pancreatitis, and KP, KΔP, KPΔS PDACs. Scale bars = 50 μm. Quantification of CD56^+^PDPN^+^DAPI^+^CK19^−^ and CD34^+^PDPN^+^DAPI^+^CK19^−^ cells relative to PDPN^+^CK19^−^DAPI^+^ fibroblasts in murine pancreatitis (n = 6), KP PDAC (n = 6), KΔP PDAC (n = 5) and KPΔS PDAC (n = 7). Results show mean ± SEM. *P adj*, Kruskal-Wallis test. Pancreatitis samples derive from three treatment cohorts. Source numerical data and unprocessed blots are available.

To understand how mixed-fibroblasts and mesothelial cells help mirror in vivo myofibroblastic enrichment, we treated PSCs, mixed-fibroblasts and mesothelial cells with TGF-β or IL-1α to induce myofibroblastic or inflammatory markers, respectively^33^. Mixed-fibroblasts and mesothelial cells were more responsive to TGF-β, upregulating myofibroblastic markers (*Tgfb1*, *Ctgf*, *Ncam1*, encoding CD56) more strongly than PSCs (Extended Data Fig. 9a). Conversely, PSCs were more responsive to IL-1α, showing greater induction of inflammatory markers than mixed-fibroblasts and mesothelial cells (Extended Data Fig. 9b). Consistently, STAT3 activation, a downstream effector of IL-1 signalling in PDAC iCAFs^33^, was higher in PSCs than in other stromal cells after IL-1α treatment (Extended Data Fig. 9c). STAT3 activation was also higher in PSCs cultured with PDAC than P organoid-conditioned media, whereas mixed-fibroblasts and mesothelial cells showed the opposite pattern (Extended Data Fig. 9d). Thus, mixed-fibroblasts and mesothelial cells are less responsive to IL-1α, favouring enrichment of myofibroblastic features in PDAC.

Our data suggest that other P organoid-secreted factors may promote inflammatory phenotypes, including JAK/STAT activation, in mixed-fibroblasts and mesothelial cells. We focused on leukaemia inhibitory factor (LIF), a potent STAT3 activator previously shown to mediate STAT3 activation in PDAC iCAFs^33^. *Lif* expression was higher in P organoid monocultures and co-cultures than in PDAC counterparts, and was higher in pancreatitis Ductal 2 cells than Ductal 1 cells or PDAC malignant cells (Extended Data Fig. 9e,f). LIF treatment robustly induced STAT3 activation in both mixed-fibroblasts and mesothelial cells (Extended Data Fig. 9g). Together, these data show that stromal cells of distinct origins respond differently to epithelial-derived cues in pancreatitis and PDAC, underscoring the importance of stromal heterogeneity in disease modelling.

We next tested whether P and PDAC organoid/multi-stroma co-cultures better capture fibroblast markers from mouse and human tissues using our cross-model and cross-species strategy. We identified genes upregulated in P versus PDAC multi-stroma (‘P multi-stroma markers’) and in PDAC versus P multi-stroma (‘PDAC multi-stroma markers’) and overlapped these with pancreatitis fibroblast and PDAC CAF markers, respectively. Both overlaps were larger than those observed for PSC co-cultures: 48% of P PSC markers (*n* = 438/905) (Fig. 6f) and 60% of P multi-stroma markers (*n* = 1,006/1,684) (Fig. 8c) overlapped with pancreatitis fibroblast markers (Supplementary Table 8). Similarly, only 15% of PDAC PSC markers overlapped with PDAC CAF markers (*n* = 174/1,155; *P* = 0.798) (Fig. 6g), whereas 31% of PDAC multi-stroma markers did so significantly (*n* = 220/707; *P* = 4.88 × 10^−25^) (Fig. 8d and Supplementary Table 8). Human cross-species comparisons showed similar significant overlap of P PSC orthologues and P multi-stroma orthologues with human pancreatitis fibroblast orthologues, whereas PDAC multi-stroma orthologues, unlike PDAC PSC orthologues, significantly overlapped with human PDAC CAF orthologues (Supplementary Table 8). We then used organoid/multi-stroma and mouse datasets to identify fibroblast markers enriched in pancreatitis (for example, *PDGFRA* and *CD34*) or PDAC (for example, *ACTA2*, *NCAM1*, *TNC*, *FN1* and *RUNX1*) for protein validation (Supplementary Table 14). Multiplex IF confirmed increased PDGFRα^+^αSMA^−^PDPN^+^ fibroblasts in pancreatitis and αSMA^+^PDGFRα^−^PDPN^+^ fibroblasts in PDAC (Fig. 8e). FN1^+^PDGFRα^−^αSMA^+^ fibroblasts were also more abundant in PDAC than pancreatitis, whereas FN1^+^αSMA^−^PDGFRα^+^ fibroblasts were not significantly different, findings validated in KΔP and KPΔS PDAC tissues (Fig. 8e and Extended Data Fig. 9h–j). RUNX1^+^PDPN^+^, TNC^+^PDPN^+^ and CD56^+^PDPN^+^ fibroblasts were enriched in PDAC relative to pancreatitis regardless of SMAD4 status, and CD34^+^PDPN^+^ fibroblasts were enriched in pancreatitis across all PDAC comparisons (Fig. 8f,g and Extended Data Fig. 9k,l). In human PDAC and matched adjacent chronic pancreatitis tissues, CD34^+^αSMA^−^PDGFRα^+^ fibroblasts were enriched in chronic pancreatitis, while FN1^+^PDGFRα^−^αSMA^+^ fibroblasts were enriched in PDAC (Fig. 8h and Supplementary Table 1). Thus, organoid/multi-stroma co-cultures better recapitulate disease-specific in vivo fibroblast heterogeneity than organoid/PSC co-cultures and help identify discrete stromal populations.

**Extended Data Fig. 10:**
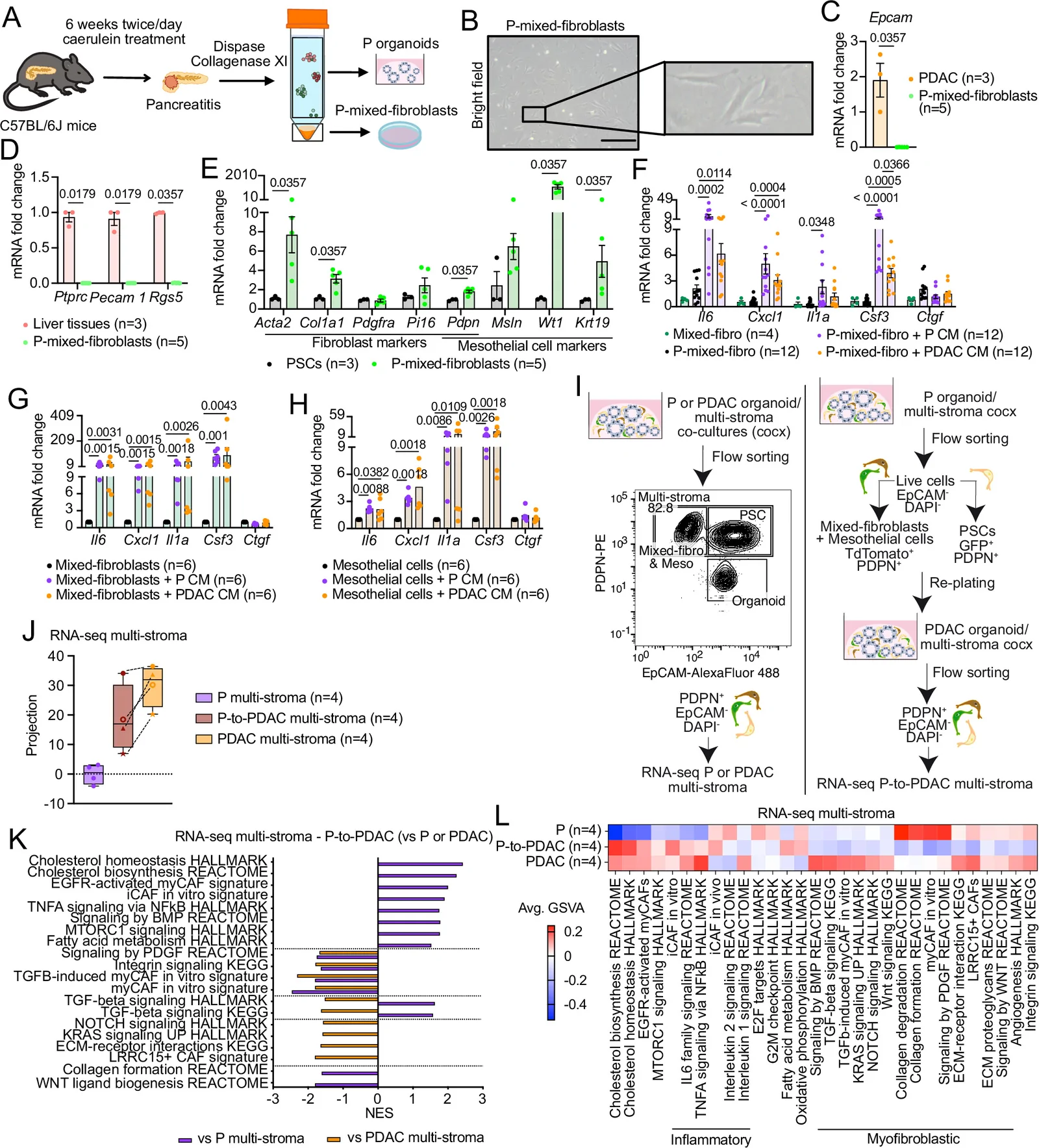
Pancreatitis-reprogrammed fibroblasts can be shaped by PDAC cues. (**A**) Schematic illustrating how pancreatitis mixed-fibroblast lines (hereafter, P-mixed-fibroblasts) were generated and validated. (**B**) Bright field images of murine P-mixed-fibroblasts cultured in 2D. Scale bar = 100 μm. Insert shows magnification. (**C**) RNA expression levels of epithelial cell marker (*Epcam*) in P-mixed-fibroblasts (n = 5) cultured in 2D and in PDAC organoids (that is, PDAC, n = 3). Results show mean ± SEM. *P adj*, Kruskal-Wallis test. (**D**) RNA expression levels of immune cell (*Ptprc*), endothelial cell (*Pecam1*) and pericyte (*Rgs5*) markers in P-mixed-fibroblasts (n = 5, n = 3 lines with two in two passages) cultured in 2D and in liver tissues (n = 3). Results show mean ± SEM. *P adj*, Kruskal-Wallis test. (**E**) RNA expression levels of fibroblast (*Acta2*, *Col1a1*, *Pdgfra*, *Pi16, Pdpn*) and mesothelial cell (*Pdpn*, *Msln*, *Wt1*, *Krt19*) markers in PSCs (n = 3) and P-mixed-fibroblasts (n = 5, n = 3 lines with two in two passages) cultured in 2D. Each data point corresponds to a biological replicate. Results show mean ± SEM. *P adj*, Kruskal-Wallis test. (**F**) qPCR analysis of *Il6*, *Cxcl1*, *Il1a*, *Csf3* and *Ctgf* in mixed-fibroblasts cultured for 4 days in Matrigel with reduced media (n = 4), as well as in P-mixed-fibroblasts cultured in Matrigel with reduced media (n = 12, n = 4 lines in three passages) or with P (n = 12, n = 6 line/organoid pairs in two passages) or PDAC (n = 12, n = 6 line/organoid pairs in two passages) organoid CM. Results show mean ± SEM. *P adj*, Kruskal-Wallis test. (**G**) qPCR analysis of *Il6*, *Cxcl1*, *Il1a*, *Csf3* and *Ctgf* in mixed-fibroblasts cultured for 4 days in Matrigel with reduced media (n = 6, n = 3 lines in two passages), or with P (n = 6, n = 3 lines in two passages) or PDAC (n = 6, n = 3 lines in two passages) organoid CM. Results show mean ± SEM. *Padj*, Kruskal-Wallis test. (**H**) qPCR analysis of *Il6*, *Cxcl1*, *Il1a*, *Csf3* and *Ctgf* in mesothelial cells cultured for 4 days in Matrigel with reduced media (n = 6, n = 3 lines in two passages), or with P (n = 6, n = 3 lines in two passages) or PDAC (n = 6, n = 3 lines in two passages) organoid CM. Results show mean ± SEM. *P adj*, Kruskal-Wallis test. (**I**) Schematic of RNA−seq analyses of flow-sorted multi-stroma from P organoid/multi-stroma cocx (that is, P multi-stroma, n = 4), PDAC organoid/multi-stroma cocx (that is, PDAC multi-stroma, n = 4) and sequential P-to-PDAC organoid/multi-stroma cocx (that is, multi-stroma cultured with PDAC organoids after being cultured with P organoids, n = 4) in RM. (**J**) Projection of P multi-stroma, P-to-PDAC multi-stroma and PDAC multi-stroma along P-PDAC transcriptional axes derived from RNA−seq data. Data are presented as a box-and-whisker plot where the box represents the 25^th^-75^th^ percentiles, the whiskers represent min to max and the middle line represents the median, while each point represents an individual sample. (**K**) Significantly upregulated and downregulated pathways identified by GSEA of P-to-PDAC multi-stroma (n = 4) compared to P multi-stroma (n = 4, purple) or PDAC multi-stroma (n = 4, orange), as assessed by RNA−seq. (**L**) Heatmap showing GSVA enrichment scores (averaged per group) of selected pathways in P multi-stroma, P-to-PDAC multi-stroma and PDAC multi-stroma, as assessed by RNA−seq. Source numerical data are available.

Given that chronic pancreatitis is a PDAC risk factor, we asked whether inflammation-reprogrammed stromal cells respond differently to PDAC-derived signals than naïve stromal cells. We established mixed-fibroblast lines from pancreatitis mice (‘P-mixed-fibroblasts’) and confirmed their elongated morphology and absence of immune, endothelial, pericyte or epithelial markers (Extended Data Fig. 10a–d). Similar to naïve mixed-fibroblasts, P-mixed-fibroblasts expressed higher myofibroblastic and mesothelial markers than PSCs (Extended Data Fig. 10e). Unlike naïve PSCs, mixed-fibroblasts and mesothelial cells, P-mixed-fibroblasts upregulated inflammatory markers more strongly in response to P than PDAC organoid-conditioned media (Extended Data Figs. 10f–h and 7g). We then compared flow-sorted multi-stromal populations from sequential P-to-PDAC co-cultures (‘P-to-PDAC multi-stroma’) with those from standard P or PDAC multi-stroma co-cultures (Extended Data Fig. 10i and Supplementary Table 15). Along the P-to-PDAC transcriptional axis, P-to-PDAC multi-stroma occupied an intermediate state between P and PDAC multi-stroma (Extended Data Fig. 10j). P-to-PDAC multi-stroma upregulated myofibroblastic and inflammatory signatures compared with P multi-stroma but remained less myofibroblastic than PDAC multi-stroma (Extended Data Fig. 10k,l). Thus, pancreatitis fibroblasts can be reprogrammed by malignant cues while retaining features of their inflammatory state.

To infer potential stromal cells of origin, we mapped in vitro-derived PSCs, mixed-fibroblasts and mesothelial cells from P or PDAC co-cultures onto in vivo pancreatitis fibroblast or PDAC CAF references using semi-supervised scANVI. In pancreatitis, Fibro 1 and Fibro 4 strongly overlapped with mesothelial cells (85%), with minor similarity to mixed-fibroblasts (7–11%) or PSCs (4–8%) (Fig. 8i). Fibro 2 was most similar to mixed-fibroblasts (51%), whereas Fibro 3 showed similarity to both mixed-fibroblasts (33%) and PSCs (38%) (Fig. 8i). Pseudotemporal analysis further suggested a stronger connection between mixed-fibroblasts and Fibro 3 (Fig. 8j). PDAC CAFs instead showed a more composite signature, and pseudotemporal analysis suggested a stronger connection between mixed-fibroblasts and PDAC CAFs (Fig. 8k,l). Although in vivo lineage tracing will be needed to define stromal origins definitively, these findings support multiple potential origins for pancreatitis fibroblasts and PDAC CAFs.

In summary, P and PDAC organoid/multi-stroma co-cultures better capture in vivo fibroblast gene expression in pancreatitis and PDAC and highlight potential contributions of distinct stromal cell types to fibroblast populations in tissues.

## Discussion

Our study uncovers previously unappreciated aspects of fibroblast heterogeneity and epithelial cell–fibroblast crosstalk in mouse and human pancreatitis and PDAC. We show that PDAC and pancreatitis differ in epithelial and fibroblast features, with myofibroblastic signatures enriched in PDAC CAFs, and that multi-stromal organoid co-cultures better capture disease-relevant epithelial and fibroblast states than organoid/PSC co-cultures. These models identify disease-specific epithelial reprogramming of stromal cells of distinct origins, infer potential in vivo origins of PDAC and pancreatitis fibroblasts, and support protein-level validation of cross-species and cross-model markers enriched in PDAC or pancreatitis.

Human chronic pancreatitis samples without PDAC are rare, and available scRNA-seq/snRNA-seq datasets often lack epithelial cells or fibroblasts, underscoring the value of mouse and in vitro models. Our cross-species analyses showed that mouse models and multi-stroma co-cultures capture many, but not all, human disease features, suggesting that some human fibroblast programmes, including inflammatory signatures in PDAC CAFs, remain underrepresented. These differences may reflect immune composition, tumour kinetics, inflammation duration, malignant cell states, mouse age or genetics, all of which can shape epithelial and fibroblast features and epithelial–stromal crosstalk^28,32,45,46^. Future studies should therefore further incorporate genetic and age-related diversity.

Because chronic pancreatitis increases PDAC risk, defining disease-specific epithelial–fibroblast crosstalk could inform PDAC interception, diagnosis and therapy. Our data suggest that pancreatitis fibroblasts can be reprogrammed by malignant cues while retaining a more inflammatory and less myofibroblastic state, potentially reflecting an intermediate stromal state during PDAC evolution in inflamed pancreas. Although further in vivo studies are needed, our findings suggest that malignant–stromal interactions differ depending on whether PDAC arises in normal or inflamed tissue. PDAC stromal-specific markers also appear more consistently conserved across genetic profiles than malignant cell-specific markers when compared with pancreatitis, supporting stromal biomarkers for early PDAC detection in these high-risk patients. We also identify candidate epithelial–fibroblast signalling axes, including IL-1 and LIF/STAT3, whose combined targeting could be tested for effects on PDAC progression, interception or prevention, especially as reducing chronic pancreatitis may lower PDAC risk^47,48^. Multi-stromal organoid co-cultures may provide tractable platforms for testing such targets and probing stromal-to-stromal crosstalk. Future versions could extend culture duration, adjust stromal ratios, distinguish mesothelial cells and mixed-fibroblasts fluorescently, and include additional PDAC genotypes plus endothelial and immune cells.

We used caerulein-based models because they are widely used to study pancreatitis in mice, but additional models, including GEMMs carrying hereditary pancreatitis mutations, could complement this work^20,49,50^. We focused on 6-week caerulein treatment because it produced stronger fibrosis and ADM, but shorter acute pancreatitis regimens also alter fibroblast and epithelial transcriptomes^51^. Whether epithelial–fibroblast crosstalk differs between acute and prolonged pancreatitis remains important, because chronic, but not acute, pancreatitis increases PDAC risk in humans^52^. Analysing caerulein-treated KC GEMMs could also clarify which epithelial-to-fibroblast signals depend on oncogenic mutations, given that PDAC genetics shape epithelial–stromal communication^28,46^.

In PDAC, PDGFRα and αSMA mark iCAF- and myCAF-enriched states, respectively^53^. Here, PDGFRα^+^αSMA^−^ fibroblasts were enriched in pancreatitis, whereas αSMA^+^PDGFRα^−^ fibroblasts were enriched in PDAC, consistent with more inflammatory pancreatitis fibroblasts and more myofibroblastic PDAC CAFs. Myofibroblastic features may therefore be more malignancy-specific, but therapeutic targeting requires caution: broad depletion of collagen I or αSMA^+^ cells could promote PDAC progression^54,55^, whereas selective targeting of myCAF subsets could reduce metastasis and therapy resistance^7,56^. Our study also links pancreatitis fibroblast subsets to markers with known disease roles: WT1 is enriched in Fibro 1 and Fibro 4, and WT1^+^ stromal cells can contribute to tumour-promoting PDAC CAFs^57^; Fibro 4 co-expresses *Muc16* and interferon-response genes; Fibro 1 expresses *Il34*; Fibro 3 expresses *Cxcl12* and *Vegfc*, suggesting immunomodulatory and angiogenic roles^58^; and *Pi16*^High^*Pdgfra*^High^ Fibro 2 may correspond to progenitor-like universal fibroblasts^43^.

MyCAF and iCAF states first identified in PDAC were later found in other malignancies, suggesting that insights from pancreatitis fibroblast heterogeneity may inform other inflammatory diseases, and vice versa. Fibroblast heterogeneity is also described across inflammatory conditions^59–63^. For example, in rheumatoid arthritis, *Fap* and *Pdpn* mark fibroblasts broadly, whereas *Thy1* expression defines populations with distinct roles^59^. In pancreatitis fibroblasts, *Thy1* and *Fap* are heterogeneous, whereas *Pdpn* is more uniform, suggesting that future perturbation studies may reveal shared fibroblast functions across inflammatory diseases.

## Methods

### Ethical regulations

Animal procedures were reviewed by the Cancer Research UK Cambridge Institute (CRUK-CI) Animal Welfare and Ethical Review Body, approved by the UK Home Office and conducted under project license PP4778090 in accordance with institutional and national regulations. Patients’ specimens were acquired and analysed under the Pancrea_tive protocol (reference: REC21/WM/0119), which was approved by the West Midlands – Black Country Research Ethics Committee.

### Human tissues

Human tissues for histological analyses were obtained from surgical resections performed at Addenbrooke’s Hospital (Cambridge, UK). Written informed consent was acquired from patients before specimens’ acquisition. Participants were not compensated. Samples were anonymized to the researchers. Available clinical information and pathology annotations are provided in Supplementary Table 1.

### Mouse models

Male and female C57BL/6J mice (strain number 632, Charles River, RRID:IMSR_JAX:000664) were purchased from Charles River Laboratories. Male and female C57BL/6J-background *Kras*^LSL-G12D/+^; *Trp53*^R172H/+^; *Pdx1*-Cre (KPC) GEMMs^23^ were bred in-house and palpated from 2 months of age to monitor tumour formation, which was confirmed by ultrasound-based imaging. All animals were housed according to the UK Home Office Code of Practice for the Housing and Care of Experimental Animals under strict barrier conditions, maintaining defined microbiological health status and absence of ectoparasites. Mice were kept at 21 °C and 55% relative humidity on a 12-h light/dark cycle, with expanded rodent diet (Labdiet) and filtered water ad libitum. Environmental enrichment included nesting material, a retreat area and chew blocks.

To model pancreatitis and acute pancreatitis, 8–9-week-old C57BL/6J mice were treated with the cholecystokinin analogue caerulein (C9026, Sigma-Aldrich) in endotoxin-free Dulbecco′s PBS (TMS-012-A, Sigma-Aldrich). To model acute pancreatitis, nine male mice were administered 100 μg kg^−1^ buprenorphine (57446/4003, Virbac) subcutaneously 1 h before 6–8 hourly intraperitoneal injections of 75 μg kg^−1^ caerulein per day for 2 days (modified from Lugea et al.^64^). Then, 48 h after the first caerulein dose on the second day of treatment, samples were harvested. To model pancreatitis, 17 male and 5 female mice were treated with 250 μg kg^−1^ caerulein twice daily for 6 weeks.

To establish orthotopic transplantation mouse models of PDAC, single cells (10,000 cells per mouse) prepared from organoid cultures were resuspended as a 35 μl suspension of 50% Matrigel in PBS and injected into the pancreas of 8–10-week-old male (all KPΔS, all KΔP and all but two KP) or female (two KP for multiplex IF) C57BL/6J mice. KPΔS PDAC organoids were established from KPC GEMM−derived PDACs that had undergone LOH of the *Trp53* WT allele^29^, and were CRISPR-modified to delete *Smad4*^28^. KΔP PDAC organoids (T113) were derived from *Kras*^*LSL-G12D/+*^; *Trp53*^*fl/fl*^; *Pdx1*-*Cre* (KPPC) GEMM PDACs^29^. No maximal tumour size/burden is defined in our project licence, mice are euthanized when they show severe clinical signs (for example, lethargy, hypothermia) or before.

### Amylase and lipase activity

Mice were anaesthetized with isoflurane, cardiac puncture was performed and blood was collected in Multivette 600 K3E tubes containing EDTA (15.1671, Sarstedt). Plasma was isolated by centrifugation at 9,600*g* for 6 min at 4 °C, and stored at −80 °C until analysis. Amylase and lipase activity levels in plasma were measured using kits from Abcam (Ab102523 and Ab102524, respectively).

### Normal, pancreatitis and PDAC organoids

All mouse PDAC organoids used in this study have undergone LOH of the *Trp53* WT allele^29^, apart from the KPC organoid line used in Extended Data Fig. 6h. Pancreatic intraepithelial neoplasia KC organoids (C57BL/6J-background) were previously described^29^. PDAC KPC organoids (C57BL/6J-background) T6-LOH, T8-LOH and GB-T12-LOH were previously described^7,29^. New mouse normal pancreas (GB-N21-23, GB-N25 and GB-N27), pancreatitis (GB-P1-5) and PDAC (GB-T11-LOH and GB-T13-LOH) organoids were established from male mice^7,29^. In brief, C57BL/6J-background male caerulein-treated pancreata or KPC tumours were digested with collagenase type XI (C9407, Sigma-Aldrich) and dispase (17105-041, Gibco). A few modifications were applied to generate pancreatitis organoids^34^. GB-T11-LOH and GB-T13-LOH were cultured with 10 μM Nutlin-3a (SML0580, Sigma-Aldrich) from passage 0 (ref. ^7^). Please note that contrary to other PDAC organoids used in this study, GB-T11-LOH organoids retain expression of p16. Organoids were propagated in complete media^35^ at 37 °C with 5% CO_2_.

### PSCs, mixed-fibroblasts, mesothelial cells and P-mixed-fibroblasts

Mouse PSCs (SV40-immortalized, C57BL/6J-background, PSC4, PSC5 and GB-PSC23) were previously described and were established from the normal pancreata of untreated C57BL/6J mice^4,7^. *Il1r1* KO (PSC4 G1, guide 5; PSC5 C9, guide 2) PSCs were also previously described^7,33^. New mouse pancreatic mixed-fibroblasts (GB-Fibro27, GB-Fibro28, GB-Fibro39 and GB-Fibro40) and mesothelial cells (GB-PSC36-M-H11, GB-PSC36-M-G1 and GB-PSC36-M-G3) were obtained while generating additional PSC lines from the normal pancreata of untreated male and female C57BL/6J mice, respectively. In brief, for each line, two and a half pancreata from C57BL/6J mice were run on a density gradient centrifugation method with Histodenz (D2158, Sigma-Aldrich) and Gey’s Balanced Salt Solution (G9779, Sigma-Aldrich)^4^. While PSCs were established from the layer that forms in between the two interphases, mixed-fibroblasts were established by outgrowth from the pellets following centrifugation. Mesothelial cells were established by enriching for cobblestone-like cells found in mixed-fibroblast preparations by single-cell plating in 96-well plates. P-mixed-fibroblasts (GB-P-Fibro1 to 5) derive from the pancreata of pancreatitis mouse models and were established by outgrowth of settled partially undigested material while generating pancreatitis organoids (from the supernatant). Mixed-fibroblasts, mesothelial cells and P-mixed-fibroblasts were immortalized using the pLVX-SV40 LT-IRES-tdTomato plasmid from Professor Tuveson^4^. PSCs, mixed-fibroblasts, mesothelial cells and P-mixed-fibroblasts were cultured in DMEM (41966029, Gibco) containing 5% FBS at 37 °C with 5% CO_2_. Cell line authentication for stromal cell lines was performed using short tandem repeat analysis at the CRUK-CI in 2023 (PSC4 and PSC5) and 2025 (GB-Fibro27, GB-Fibro28, GB-PSC36-M-H11 and GB-PSC36-M-G1). The results confirmed 92% similarity across these cell lines. *Mycoplasma* testing was performed before each freezing and always tested negative.

### In vitro cell treatments and organoid-conditioned media experiments

PSCs, mixed-fibroblasts and mesothelial cells were treated in Matrigel in reduced media (that is, 5% FBS/DMEM), organoid-conditioned media or reduced media with 20 ng ml^−1^ human TGF-β1 (T7039, Sigma), 1–2 ng ml^−1^ mouse IL-1α (400-ML-005/CF, R&D Systems) and 5 ng ml^−1^ mouse LIF (250-02-25UG, ThermoFisher) for as long as specified in the figure legends. PSCs were cultured with P or PDAC organoid-conditioned media with 3 μg ml^−1^ IL-1α neutralizing antibody (MAB4001, R&D Systems; RRID:AB_2124216) or 3 μg ml^−1^ IgG isotype control (400902, BioLegend) for as long as specified in the figure legends.

### Reverse transcription qPCR analyses

RNA (200 ng to 1 μg) of cell lines, PDAC organoids and mouse liver tissues was reverse transcribed using TaqMan reverse transcription reagents (N808 0234, Applied Biosystems). Quantitative PCR (qPCR) was performed using gene-specific TaqMan probes (ThermoFisher) (Supplementary Table 16) and TaqMan master mix (4440040, Applied Biosystems).

### ELISA

Conditioned media samples were collected from P and PDAC organoids cultured alone or in co-culture with PSCs in reduced media for 3.5 days. Enzyme-linked immunosorbent assays (ELISAs) were used to detect mouse IL-1α (MLA00, R&D Systems) and TGF-β1 (BMS608-4, ThermoFisher).

### Organoid growth assays

For organoid growth assays, 5,000 single cells were plated in 50 μl of 100% Matrigel on 24-well plates (Corning/Nunc) and cultured in 500 μl of complete media^35^. Growth was followed for 120 h with an Incucyte organoid module (Sartorius) with four measurements of the organoid area per condition every 3 h. Data were normalized to the first measurement on day 0.

### Western blot analyses

Cells were harvested in Cell Recovery Solution (354253, Corning) supplemented with protease inhibitors (11836170001, Roche) and phosphatase inhibitors (4906837001, Roche) and incubated for 30 min on ice. Cells were pelleted at 1,500*g* for 5 min and lysed in 0.1% Triton X-100, 15 mmol l^−1^ NaCl, 0.5 mmol l^−1^ EDTA, 5 mmol l^−1^ Tris, pH 7.5 supplemented with protease inhibitors (11836170001, Roche) and phosphatase inhibitors (4906837001, Roche). Cells were incubated on ice for 30 min, briefly vortexed and pelleted at 16,800*g* for 10 min at 4 °C. The protein concentration of the collected supernatant was determined using the DC protein assay (5000113-5, Bio-Rad). Standard procedures were used for western blotting. Primary antibodies are listed in Supplementary Table 16. Total proteins were blotted after stripping the respective phosphorylated proteins. In brief, membranes were incubated with a stripping buffer (100 mM glycine with 1% SDS in water, pH 2.5) on a shaker for 20 min at room temperature, washed three times with 0.1% Tween 20 in TBS before blocking and following standard procedures for western blotting. Proteins were detected using HRP-conjugated secondary antibodies (Jackson ImmunoResearch Laboratories). Western blots shown are representative examples and have been repeated with at least two lines per condition.

### Immunohistochemical and histological analyses

Standard procedures were used for tissues and co-cultures. Primary antibodies are listed in Supplementary Table 16. Hematoxylin (H-3404-100; Vector Lab) was used as nuclear counterstain. PDPN immunohistochemistry was performed by the CRUK-CI Histopathology core (J.L.M.). Sections were dewaxed and rehydrated on Leica’s automated ST5020 Multistainer. Staining was performed on Leica’s automated Bond-III platform in conjunction with their Polymer Refine Detection System (DS9800) and a modified version of their standard template. Antigen retrieval (AR) was performed in sodium citrate (Leica’s Epitope Retrieval solution 1, AR9961) for 20 min at 100 °C. The primary antibody was diluted in 1% donkey serum in 300 mM TBS-0.05% Tween. The secondary antibody (anti-Syrian hamster, Jackson ImmunoResearch 307-006-003 and Polymer from Leica Kit) was diluted to 5.2 μg ml^−1^ in Bond diluent. Dehydration and clearing were performed on Leica’s automated ST5020 before sections were mounted on Leica’s coverslipper CV5030 with DPX Mountant for Histology (Sigma-Aldrich, 06522-500 ml). Masson’s trichrome and hematoxylin and eosin (H&E) stains were performed according to standard protocols by the CRUK-CI Histopathology core. Following 1-h baking at 60 °C, sections were dewaxed and rehydrated on Leica automated ST5020. The staining was performed on Leica’s automated Bond-III platform as above. AR was performed at 100 °C for 20 min in citrate or Tris–EDTA for F4/80 or PDGFRα, respectively. AR for αSMA was performed at 100 °C for 10 min in Tris–EDTA. AR for CK19 was performed with proteinase K at 37 °C using the Leica’s Bond enzyme concentrate (AR9551). Protein block (X090930-2, Dako) was applied for PDGFRα, CK19 and F4/80. The anti-rat secondary antibody (A110-322A, Bethyl Laboratories; RRID: AB_10681533) was applied for CK19 and F4/80, and DAB Enhancer (AR9432, Leica) was used for all. Dehydration, clearing and mounting were performed as above. Stained sections were scanned with Aperio ScanScope CS and analysed using ImageScope software (RRID:SCR_014311) positive pixel count algorithms or using HALO (RRID:SCR_018350). Tissue images were obtained with an Axio Vert.A1 (ZEISS). Mouse PDAC undifferentiated, moderately differentiated and well-differentiated areas, as well as human PDAC and chronic pancreatitis regions, were annotated by a pathologist (R.B.) before downstream analyses. The percentage of collagen area was determined by calculating the percentage of blue pixels relative to the entire stained area. To quantify αSMA, PDPN, PDGFR-α, CK19 and F4/80 stains, the percentage of positive pixels was calculated relative to the entire stained section. To quantify Ly6G, the percentage of positive nuclei was calculated relative to the total number of nuclei. Necrotic areas were removed before quantification. Stains and quantifications were performed blindly before data visualisation.

### Multiplex IF and RNA ISH analyses

Primary antibodies used are listed in Supplementary Table 16. Paraffin embedded tissue sections were dewaxed and rehydrated as per standard procedures then washed in deionized water. Tissue sections went through six rounds of AR, followed by blocking, incubation with primary antibody, secondary antibody and opal fluorophore. AR was performed in AR pH9 1X Tris–EDTA buffer (ab93684, Abcam) or AR pH6 1X AR6 buffer (AR600250ML, Akoya Biosciences) and boiling in a microwave for 20 min. After cooling, tissue slides were washed in deionized water by rocking twice for 2 min and then 2 min in PBS + 0.1% Tween20 (PBS-T). Tissue sections were outlined using a hydrophobic barrier pen and incubated in blocking buffer (ARD1001EA, Akoya Biosciences) at room temperature for 15 min in a humidified chamber. Slides were incubated with primary antibody for 45 min at room temperature or 4 °C overnight. Washing was performed by rocking the slides in PBS-T for 3 ×2 min. This was followed by incubation with secondary antibody (ARH1001EA, Akoya Biosciences) for 15 min at room temperature, washing and incubation with opal fluorophore for 12 min. Slides were washed again then immersed in AR buffer to begin the cycle from microwaving to fluorophore incubation for other primary antibodies. After the last round of staining incubation with opal fluorophore 780, DAPI was added to the tissues for 5 min for tissues or for 15 min for co-cultures. After washing, slides were mounted in ProLong Gold Antifade mountant (P36930, ThermoFisher) and 1.5-mm coverslips (2980-245; Corning). Slides were then imaged using Vectra Polaris. Staining was analysed using QuPath^65^. All sections were set to the same signal thresholds per channel, annotated by area to analyse and cells were detected using DAPI signal to identify each nucleus using the cell detection tool. Positivity for the markers of interest was defined by signal threshold in the object classifier tool and all parameters applied to each cohort remained the same. All buffers, anti-rabbit and anti-mouse secondary antibodies, Opal dyes and DAPI used were supplied in the Opal 6-Plex manual detection kit (NEL861001KT, Akoya Biosciences). Additional goat anti-rat secondary (MP-7444-15, Vector) was used for antibodies raised in rat and goat anti-Syrian hamster secondary (1:1,000; ab6892, Abcam; RRID:AB_955427) was used for antibodies raised in Syrian hamster.

RNA ISH was performed by the Histopathology Core at the CRUK-CI (J.J.) followed by multiplex IF. RNAscope 2.5 LS Probes (Advanced Cell Diagnostics, ACD) used were: Mm Fap (423888), Mm Cxcl12-C2 (422718-C2), Mm Vegfc-C3 (492708-C3), Mm Il34 (428208), Mm Pi16-C2 (451318-C2) and Mm Muc16-C3 (567778-C3). In brief, 3-µm FFPE sections of mouse pancreatitis tissues were baked for 1 h at 60 °C before loading onto a Bond RX instrument (Leica Biosystems). Slides were deparaffinized and rehydrated on board before heat pre-treatment with Epitope Retrieval Solution 2 (AR9640, Leica Biosystems) at 95 °C for 15 min, before a protease-free reagent step for 30 min at ambient temperature. RNA ISH was performed using ACD RNAscope 2.5 LS Multiplex Reagent Kit (322800). All buffers, anti-rabbit and anti-mouse secondary antibodies, Opal dyes and DAPI used were supplied in the Opal 6-Plex manual detection kit (NEL861001KT, Akoya biosciences), additional horse anti-goat secondary (30116, Vector) was used for antibodies raised in goat. The slides were imaged on the PhenoImager HT (Akoya biosciences) to create whole-slide images. Images were scanned at 40× magnification, with a resolution of 0.25 µm per pixel.

### Flow cytometry and cell sorting

Primary antibodies used are listed in Supplementary Table 16. For 3D monocultures or co-cultures, following single-cell digestion of co-cultures, cells were blocked on ice with CD16/CD32 in PBS for 15 min before staining for 30 min on ice.

PSCs, mixed-fibroblasts and mesothelial cells were cultured in monolayer and analysed by flow cytometry for CD200-APC or treated with 100 ng ml^−1^ recombinant mouse interferon gamma (IFNγ; 315-05, PeproTech)^66^ for 48 h and then analysed by flow cytometry for MHCII−BV785.

PDAC or P organoids were cultured alone or with 35,000 PSCs per Matrigel dome for 3.5 days in reduced media^4,7,28^. For flow cytometric analysis, cells were stained with anti-mouse CD326 (EpCAM)-AlexaFluor 488, PDPN-APC/Cy7 and LIVE/DEAD Fixable Blue Dead Cell stain. Cells were resuspended in PBS and analysed on a CYTEK AURORA spectral flow cytometer. Flow analyses were performed blindly using FlowJo 10.8.2 (RRID: SCR_008520) before plotting the data for visualization. For sorting of epithelial cells and fibroblasts, cells were stained with anti-mouse CD326 (EpCAM)-AlexaFluor 488 and PDPN-PE. Cells were resuspended in PBS with DAPI and sorted with a BD FACSMelody cell sorter.

For cell sorting of P, PDAC and P−to-PDAC organoid/multi-stroma co-cultures, we used GFP^+^ *Rosa26* KO PSCs carrying the LentisgRNA-EFS-GFP-neo (LRGN) plasmid^7^ and TdTomato^+^ mixed-fibroblasts and mesothelial cells, which were immortalized using the pLVX-SV40 LT-IRES-tdTomato plasmid^4^. PDAC or P organoids were co-cultured with 30,000 stromal cells (10,000 each of PSCs, mixed-fibroblasts and mesothelial cells) per Matrigel dome for 3.5 days in reduced media, and stained and sorted as above. For P-to-PDAC co-cultures, P organoids were co-cultured with 30,000 stromal cells as above. Cells were stained with anti-mouse CD326 (EpCAM)-APC/Cyanine7 and PDPN-PE, resuspended in PBS with DAPI and sorted with a BD FACSMelody cell sorter. PSCs were flow-sorted as GFP^+^PDPN^+^EpCAM^−^DAPI^−^ cells, while mixed-fibroblasts and mesothelial cells were flow-sorted as GFP^−^PDPN^+^EpCAM^−^DAPI^−^ cells. To establish P-to-PDAC co-cultures, PDAC organoids were co-cultured with 30,000 flow-sorted stromal cells (10,000 PSCs and 20,000 combined mixed-fibroblasts/mesothelial cells) per Matrigel dome for 3.5 days in reduced media before processing and sorting as organoid/PSC co-cultures.

### Bulk RNA-seq analyses

Samples were collected in 1 ml of TRIzol Reagent (15596018, Invitrogen). Although only two PSC lines were used, these samples do not represent simple technical replicates. Each co-culture condition involved a distinct PSC–organoid pairing, while PSC monocultures were derived from independent flow-sorting experiments using lines at different passages. RNA was extracted using the PureLink RNA mini kit (12183018A, Invitrogen). RNA concentration was measured using a Qubit and RNA quality was assessed on a TapeStation 4200 (Agilent) using the Agilent RNA ScreenTape kit. mRNA library preparations were performed using 55 μl of 10 ng ml^−1^ per sample (RNA integrity number ≥8.8). Illumina libraries were sequenced on a NovaSeq6000 except for cells from organoid/multi-stroma co-cultures, which were sequenced on a NovaSeqX. Raw FASTQ files were aligned to the GRCm38 (release 109, kmer 23) reference genome using the quasi-alignment tool Salmon^67^. The resulting transcript quantification files were combined, and transcript-level estimates were aggregated to the gene level using the R package tximport^68^. Genes with fewer than ten total counts across all samples were excluded from downstream analyses. Differential expression analysis (DEA) was performed using the R package DESeq2^69^. Genes with an adjusted *P* value <0.05 were considered significantly differentially expressed. Gene set enrichment analysis (GSEA) was performed using the R package clusterProfiler^70^ against the Hallmark, Reactome and C2 canonical pathway collection (C2.cp.v5.1) downloaded from the Molecular Signatures Database (MSigDB)^71^. Genes were pre-ranked on the basis of the metric: −log_10_(*P* value) × sign(log_2_(fold change)). Pathways were considered significantly enriched at a false discovery rate <0.25 and an absolute normalized enrichment score (|NES|) >1.5. Gene set variation analysis (GSVA) was performed using the GSVA R package^72^, utilizing the average GSVA score across samples per condition.

Analyses are included in Supplementary Table 10 (organoids in complete media), Supplementary Tables 11 and 12 (organoids and PSCs from co-cultures) and Supplementary Table 15 (organoids and multi-stroma from co-cultures).

To quantify the transcriptional state transition among P, P-to-PDAC and PDAC multi-stroma conditions, a transcriptional axis between P and PDAC was defined using the variance-stabilized transformation from DESeq2^69^. Centroids representing the mean expression profile for each condition were computed, and the vector connecting the P and PDAC centroids was used to define the direction of transcriptional change. Individual samples were projected onto this axis via scalar projection, yielding a continuous score representing their relative position between the P and PDAC transcriptional states. Higher projection scores indicate greater similarity to the PDAC state, whereas lower scores indicate greater similarity to the P state.

### scRNA-seq analyses of organoid/multi-stroma co-cultures

PDAC or P organoids were co-cultured with 30,000 stromal cells (10,000 each of GFP^+^ *Rosa26* KO PSCs and TdTomato^+^ mixed-fibroblasts and mesothelial cells) per Matrigel dome for 3.5 days in reduced media, as described in the ‘Flow cytometry and cell sorting’ section. 10x Genomics 3′ V4 4-Plex (that is, on-chip multiplexing; OCM) libraries were generated following the 3′ OCM protocol utilizing recommended reagents and conditions. Libraries were quality controlled via Qubit and TapeStation then pooled to 10 nM, loaded via qPCR targeting the P5/P7 adaptors and sequenced to a depth of ~40,000 reads per cell on the NovaSeqX Plus device. Each OCM FASTQ file contains four samples. To demultiplex them, CellRanger (10x Genomics) ‘multi’ function was used following the recommended workflow and a modified Mouse mm10 (2020-A) reference genome that included the reporter genes, *Tdtomato* and *Gfp*. From the resulting output, the raw_feature_bc_matrix files were used as input for CellBender to remove ambient RNA, with an fpr of 0.01 (ref. ^73^). Each sample underwent standard quality control using Scanpy^74^. For preliminary quality control filtering, low-quality cells and outliers were excluded on the basis of total counts (1st and 99th percentiles), gene counts (<100 genes) and mitochondrial gene content (>5%). Doublets were detected, and later removed, using the SOLO model implemented in scvi-tools^27,75^. Finally, all samples were concatenated into a single *Anndata* object and integrated using scvi-tools^27^ using ‘SampleID’ as a batch key. After integration, data was clustered using the Leiden algorithm based on the top 2,000 highly variable genes and the top 30 PCs^76^. Cell clusters were annotated on the basis of known gene marker and reporter gene expression. DEA was applied using the model-based analysis of single-cell transcriptomics (MAST) method^77^, incorporating sample ID and batch ID (when applicable) as a latent variable. Genes for GSEA were ranked on the basis of the log_2_ fold change derived from this analysis. Analyses are included in Supplementary Table 13.

### Single-nuclei isolation and RNA-seq of mouse tissues

Single nuclei isolation of pancreatitis and PDAC tissues was performed as previously described^26^. We submitted 35K nuclei for PDAC and 70K for pancreatitis tissues. These samples were processed using the same pipeline for OCM samples above. To validate malignant cell identity, copy number variation (CNV) analysis was performed using a python implementation of inferCNV (Trinity CTAT Project; https://github.com/broadinstitute/inferCNV) to estimate the copy number status across cell types with a fibroblast cell cluster as reference and a 250-gene window size. Metadata and analyses are included in Supplementary Tables 6 and 7.

### NicheNet

NichenetR^78^ was applied to bulk RNA-seq and snRNA-seq data. Genes of interest were defined from the corresponding DEA as significant DEGs (adjusted *P* <0.05, |log_2_fold change|>1).

### CellChat

CellChat R package^79^ was utilized on Seurat objects with default recommended settings.

### Mapping stromal populations to in vivo fibroblasts

To map transcriptional correspondence between in vitro stromal cell populations and in vivo pancreatitis fibroblast or PDAC CAF states, we used a probabilistic framework based on scVI and scANVI, implemented via scArches^27,80^. A reference model was trained on in vivo fibroblast and CAF subclusters using 4,000 highly variable genes (a latent dimension of 20) and extended to a scANVI classifier with subcluster labels and an ‘Unknown’ placeholder to maintain label consistency. In vitro stromal cells were analysed separately for pancreatitis and PDAC conditions. Each query AnnData object was initialized as ‘Unknown’, projected into the reference latent space via scArches and fine-tuned for 20 epochs. For each in vitro cell, soft label probabilities indicated transcriptional similarity to in vivo fibroblast or CAF subtypes. In addition, in vivo pancreatitis fibroblast subclusters were projected onto in vivo CAF references to assess potential state transitions. Cluster-level mean probabilities were summarized as normalized confusion-matrix heat maps. All analyses were done on a Mac M1 processor and the accelerator was set to ‘mps’.

### PAGA

Pseudotemporal trajectories of annotated cell types were inferred using partition-based graph abstraction (PAGA)^81^, implemented in Scanpy via the sc.tl.paga function.

### Gene signature scoring

Marker gene lists were identified for multiple cell clusters from the scRNA-seq and snRNA-seq datasets using the rank_genes_groups function in Scanpy and ‘wilcoxon’ as the preferred method^74^. Genes with *P* < 0.05 and expression in at least 10% of cells within the target cluster were retained. Gene lists used are included in Supplementary Tables 2, 6,7 and 9. The AUCell method that is implemented in the decoupleR python library was used to enrich gene lists on sc/snRNA-seq data^31,82^.

### Visium HD spatial transcriptomics of mouse tissues

#### Image processing and spatial registration

Histological and CytAssist images were prepared following 10x Genomics Space Ranger specifications. Whole-slide H&E images in SVS format were converted to TIFF format at full resolution (downsampling factor of 1) using QuPath^65^ to preserve native pixel density. Owing to morphological variability and sample handling inconsistencies, manual alignment between CytAssist images and H&E images was performed to ensure precise spatial correspondence. The resulting spatial registration transformation matrix was exported as a JSON file and provided as input to the Space Ranger pipeline.

#### Read alignment and spatial binning

Raw FASTQ files, paired with registered CytAssist and H&E images, were processed using Space Ranger v4.0.1 (10x Genomics). Sequencing reads were aligned to the mm10 (2020-A) reference genome using the Mouse Probe Set v2 for feature quantification. To enhance signal robustness while maintaining near single-cell resolution, gene expression counts were aggregated into 8-µm spatial bins for all downstream analyses.

#### Quality control and deconvolution analysis

Spatially aware quality control was performed using SpotSweeper^83^ to identify and remove low-quality capture locations. Default parameters were applied, adjusting the number of nearest neighbours (*k* = 48) to accommodate the square grid geometry of the Visium HD platform. Cell-type deconvolution was performed using the Python package flashdeconv^84^ with default parameters to estimate cellular composition within individual capture locations. To maximize tissue-specific resolution, context-dependent reference datasets were used. PDAC samples were deconvolved against a mouse PDAC scRNA-seq dataset^32^; normal pancreatic tissues utilized a mouse endocrine cell scRNA-seq dataset (control samples)^85^ combined with non-malignant cells from a mouse PDAC scRNA-seq dataset^32^; pancreatitis samples utilized the mouse snRNA-seq dataset generated in this study.

#### Downstream analysis

Downstream normalization, dimensionality reduction, clustering and visualization, were performed using standard spatial transcriptomics workflows in Scanpy. Spatial data handling and visualization were conducted using Squidpy^86^ alongside the spatialdata, spatialdata-io and spatialdata-plot Python packages^87^. For DEA of fibroblast cluster spatial bins, a pseudobulk aggregation approach was applied. Spatial bins assigned to fibroblasts were subsetted and raw counts were aggregated at the sample level. The DEA was performed using default parameters of decoupleR and PyDESeq2^69,82,88^. GSEA was performed as done for bulk RNA-seq. Metadata and analyses are included in Supplementary Table 5.

### scRNA-seq of human chronic pancreatitis and PDAC tissues

Raw FASTQ files of four human chronic pancreatitis (excluding the autoimmune sample) and five human PDAC (untreated) samples were obtained from Dimitrieva et al.^19^ and are accessible at the database of Genotypes and Phenotypes (dbGaP) under study accession number phs003751.v1.p1. The sample metadata and dataset analysis are presented in Supplementary Table 2. scRNA-seq analysis was performed as above but using the reference genome GRCH38 (refdata-gex-GRCh38-2024-A).

### snRNA-seq of human chronic pancreatitis and PDAC tissues

Count matrices of four human PDAC and three human chronic pancreatitis samples were obtained using two different publicly available datasets (‘Data availability’ section) and were downloaded and read into Scanpy. Preliminary quality control filtering was performed as done for the organoid/multi-stroma co-cultures. Samples were integrated using scVI, with ‘DataID’ used as a batch key. Cells were clustered using Leiden clustering based on 3,000 highly variable genes and the top 30 PCs and annotated on the basis of known markers. The sample metadata and dataset analysis are included in Supplementary Table 3.

### Pathway enrichment analysis

Enricher function from clusterprofiler^70^ was used to perform pathway enrichment analysis on genes (based on EntrezID) shared by in vivo mouse and human PDAC CAFs or in vivo mouse and human pancreatitis fibroblasts, as well as on genes unique to human PDAC CAFs or human pancreatitis fibroblasts relative to their mouse counterparts (Extended Data Fig. 4e). Gene lists are included in Supplementary Table 8.

### Hypergeometric tests and the Jaccard index

Hypergeometric tests, odds ratios and the Jaccard index were calculated for overlapping genes between two gene lists using the GeneOverlap R package and are listed in Supplementary Table 8. The universe was set to 18,000 (for mouse comparisons) or 16,076 (for non-redundant mouse-to-human orthologues for cross-species comparisons) to represent the number of curated protein-coding genes and including *CDKN2A* and *CDKN2B* (https://bioconductor.org/packages/release/bioc/html/GeneOverlap.html).

### Statistics and reproducibility

Statistical analyses, data processing and visualization were carried out using R, Python, bash, Morpheus (https://software.broadinstitute.org/morpheus) and GraphPad Prism software. Statistical analyses between two groups were performed using non-parametric two-sided Mann–Whitney tests. Statistical analyses between three or more groups were performed using a non-parametric one-way analysis of variance (ANOVA) Kruskal–Wallis test followed by Dunn’s post hoc controlling for multiple comparisons with the Benjamini–Hochberg false discovery rate correction. These statistical methods are non-parametric and therefore do not require assumptions of normality or equal variances. The Jaccard index and hypergeometric test were used to calculate the significance of overlaps. All statistical details of experiments are specified in the figure legends and/or panel figures, including how significance was defined and how many independent experimental replicates where performed, when relevant. Exact *P* or *q* values (calculated in R) are provided in the Source data whenever figure panels report <0.0001 (calculated in GraphPad Prism). Each measurement/replicate (*n*) shown corresponds to a distinct sample (that is, individual mice, cell lines, organoid lines, cell/organoid pairs or different passages of the same organoid/cell line, as indicated in the figure legends) unless otherwise specified. Potential covariates (for example, batch ID, organoid type and cell line) were addressed and modelled as fixed effects within the analytical design (DESeq2 formula design) or regressed out to account for known sources of technical variation during batch-effect correction. A file containing numerical source data not included in Supplementary Tables 1–16 is available. No statistical method was used to predetermine sample size. Sample sizes were determined on the basis of similar prior studies showing they were sufficient for biologically meaningful conclusions^7,28^. No data were excluded from the analyses. The experiments were typically neither randomized nor carried out blindly (for example, because pancreatitis and PDAC models are clearly distinguishable) to avoid swapping of organoid lines. Randomization was performed for mice treated with caerulein or PBS. Data analyses were blind whenever possible (for example, multiplex IF analyses across PDACs with distinct genetics).

### Reporting summary

Further information on research design is available in the Nature Portfolio Reporting Summary linked to this article.

## Online content

Any methods, additional references, Nature Portfolio reporting summaries, source data, extended data, supplementary information, acknowledgements, peer review information; details of author contributions and competing interests; and statements of data and code availability are available at https://doi.org/10.1038/s41556-026-02057-w.

## Supplementary information

Reporting Summary

Peer Review File

Supplementary Table 1 Human pancreatitis and PDAC tissues for histological analyses.

Supplementary Table 2 scRNA−seq of human pancreatitis and PDAC.

Supplementary Table 3 snRNA-seq of human pancreatitis and PDAC.

Supplementary Table 4 Mouse PDAC differentiation status scoring.

Supplementary Table 5 Visium HD spatial transcriptomics of mouse normal, pancreatitis and PDAC.

Supplementary Table 6 snRNA-seq of mouse pancreatitis and PDAC (KPC GEMM).

Supplementary Table 7 snRNA-seq of mouse pancreatitis and PDAC (KP and KPDS orthotopic models).

Supplementary Table 8 Jaccard analyses of overlaps of differentially expressed genes from in vitro and in vivo mouse pancreatitis and PDAC.

Supplementary Table 9 Mouse fibroblast signatures.

Supplementary Table 10 RNA-sequencing of mouse normal, P and PDAC organoids in complete media.

Supplementary Table 11 RNA-sequencing of mouse organoids sorted from P and PDAC monocultures or co-cultures with PSCs in reduced media.

Supplementary Table 12 RNA-seq of mouse PSCs sorted from monocultures or co-cultures with P and PDAC organoids.

Supplementary Table 13 scRNA-seq of P and PDAC organoid/multi-stroma co-cultures.

Supplementary Table 14 Cross-model overlaps.

Supplementary Table 15 RNA-seq of mouse multi-stroma sorted from co-cultures with P or PDAC organoids, or after sequential co-culture with P and PDAC organoids.

Supplementary Table 16 List of qPCR probes and antibodies.

## Source data

Source Data Fig. 1 Numerical source data.

Source Data Fig. 2 Unprocessed western blots.

## Data Availability

All sequencing data generated in this study have been deposited in the Gene Expression Omnibus. Visium HD spatial transcriptomics data (Fig. 2) are under accession number GSE329811. snRNA-seq (Fig. 3) are available under accession numbers GSE311531 and GSE329665. Bulk RNA-seq data supporting Figs. 4–6 are available under accession number GSE311286. Multi-stroma co-culture scRNA-seq data (Figs. 7 and 8) are available under the accession number GSE311279. Bulk RNA-seq data supporting Extended Data Fig. 10 are available under accession number GSE329640. Publicly available datasets analysed in this study were obtained from previously published sources. The scRNA-seq data supporting Fig. 1 are accessible at dbGaP (study accession no. phs003751.v1.p1). The snRNA-seq data supporting Fig. 1 are available at the National Genomics Data Center (accession no. HRA006180; Chen et al.^21^) and the Charité CellBrowser (Tosti et al.^22^). Numerical source data and unprocessed gels are provided with this paper. Representative images of immunoblotting, immunohistochemistry and multiplexed IF are provided. All other data supporting the findings from this study and step-by-step protocols are available from the corresponding author on reasonable request. Source data are provided with this paper.

## Code availability

All analyses were performed using publicly available software packages. No custom code was used in this study.
