# Supplementary material for: Multi-stromal organoid co-culture modelling reveals epithelial–fibroblast heterogeneity in pancreatic cancer and pancreatitis
Source: Nat Cell Biol. 2026 Sep 2;28(10):2119–37. doi: 10.1038/s41556-026-02057-w (PMC13661236; doi:10.1038/s41556-026-02057-w)
Supplement: Supplementary file 1 [file 41556_2026_2057_MOESM1_ESM.pdf]

nature portfolio

Corresponding author(s): Giulia Biffi

Last updated by author(s): Jul 7, 2026

# Reporting Summary

Nature Portfolio wishes to improve the reproducibility of the work that we publish. This form provides structure for consistency and transparency in reporting. For further information on Nature Portfolio policies, see our Editorial Policies and the Editorial Policy Checklist.

## Statistics

For all statistical analyses, confirm that the following items are present in the figure legend, table legend, main text, or Methods section.

| n/a | Confirmed | |
|---|---|---|
| ☐ | ☒ | The exact sample size (*n*) for each experimental group/condition, given as a discrete number and unit of measurement |
| ☐ | ☒ | A statement on whether measurements were taken from distinct samples or whether the same sample was measured repeatedly |
| ☐ | ☒ | The statistical test(s) used AND whether they are one- or two-sided<br>*Only common tests should be described solely by name; describe more complex techniques in the Methods section.* |
| ☐ | ☒ | A description of all covariates tested |
| ☐ | ☒ | A description of any assumptions or corrections, such as tests of normality and adjustment for multiple comparisons |
| ☐ | ☒ | A full description of the statistical parameters including central tendency (e.g. means) or other basic estimates (e.g. regression coefficient) AND variation (e.g. standard deviation) or associated estimates of uncertainty (e.g. confidence intervals) |
| ☐ | ☒ | For null hypothesis testing, the test statistic (e.g. *F*, *t*, *r*) with confidence intervals, effect sizes, degrees of freedom and *P* value noted<br>*Give P values as exact values whenever suitable.* |
| ☒ | ☐ | For Bayesian analysis, information on the choice of priors and Markov chain Monte Carlo settings |
| ☒ | ☐ | For hierarchical and complex designs, identification of the appropriate level for tests and full reporting of outcomes |
| ☒ | ☐ | Estimates of effect sizes (e.g. Cohen's *d*, Pearson's *r*), indicating how they were calculated |

*Our web collection on statistics for biologists contains articles on many of the points above.*

## Software and code

Policy information about availability of computer code

| | |
|---|---|
| Data collection | IncuCyte S3;<br>ClarioStar Plus 6.20;<br>Zeiss 2.3 blue edition;<br>BD FACSChorus 3.0;<br>BD FACSDiva v9.9;<br>CYTEK AURORA SpectroFlo 3.3.0 (12282023);<br>Aperio Scan CS;<br>LI-COR Acquisition 2.1;<br>Axio Vert.A1 (ZEISS).<br><br>All single-nuclei RNA-sequencing and single-cell RNA-sequencing data in the study were processed using CellRanger pipeline (v8.0.1).<br>Bulk RNA-sequencing data were processed using quasi-alignment salmon tool (v1.9.0). |
| Data analysis | IncuCyte IC5117;<br>CLARIOstar MARS 4.20;<br>Affinity Designer 2.5.2;<br>GraphPad Prism v10.6.1;<br>Visual Studio Code 1.106.3;<br>Aperio ImageScope v12.4.6.5003;<br>HALO v4.0 (RROD:SCR_018350); |

QuPath v0.6.0;
FlowJo 10.8.2 (RRID:SCR_008520).

Single-nuclei and single-cell RNA-sequencing: CellRanger v8.0.1, CellBender v0.3.0, Scanpy v1.11.1, scvi-tools v1.3.3, infercnvpy v0.6.0, decoupler v2.1.1, scipy v1.11.2, scikit-learn v1.5.2, scCODA v.0.1.9, CellChat v2.2.0, NichenetR v 2.2.1.1.
Gene set enrichment analysis: MSigDB v7.1, org.Mm.eg.db v2.1, org.Hs.eg.db v2.1, clusterprofiler v4.18.2, enrichplot v1.30.3.
Bulk RNA-sequencing: Salmon v1.9.0, tximport v 1.38.1, apeglm v1.32.0, Deseq2 v1.50.2.
Spatial Transcriptomics Visium HD: spatialdata v0.5.0, squidpy v1.8.1, spatialdata-io v.3.0, spatialdata-plot v0.2.12, flashdeconv v0.1.6, spaceranger v4.0.1
Data wrangling and visualization: R v4.5.1, tidyverse v2.0.0, tidyr v1.3.1, dplyr v.1.1.4, ggplot2 v4.0.1, janitor v2.2.1, tibble v3.3.0, python 3.9.18, seaborn 0.12.2, pandas v2.1.0, numpy v1.24.4, matplotlib 3.7.2.

Code Availability
This paper does not report original code.
All analyses were performed using publicly available software packages. No custom code was used in this study.

For manuscripts utilizing custom algorithms or software that are central to the research but not yet described in published literature, software must be made available to editors and reviewers. We strongly encourage code deposition in a community repository (e.g. GitHub). See the Nature Portfolio guidelines for submitting code & software for further information.

# Data

Policy information about availability of data

All manuscripts must include a data availability statement. This statement should provide the following information, where applicable:

- Accession codes, unique identifiers, or web links for publicly available datasets
- A description of any restrictions on data availability
- For clinical datasets or third party data, please ensure that the statement adheres to our policy

Data Availability
(1) All RNA-sequencing, single-cell RNA-sequencing, single-nuclei RNA-sequencing and Visium HD spatial transcriptomics data generated in this study have been deposited at the GEO. In vivo murine single-nuclei RNA-sequencing data is available under GSE311531 and GSE329665, in vitro multi-stroma co-culture single-cell RNA-sequencing is under GSE311279, in vitro PSC co-culture and organoid and PSC monoculture bulk RNA-sequencing is under GSE311286 and P, PDAC and P-to-PDAC multi-stroma bulk RNA-sequencing under GSE329640; Visium HD spatial trancriptomics data is under GSE329811.
(2) Human single-cell RNA-sequencing data were downloaded from a deposited dbGaP Study (phs003751.v1.p1) using sratoolkit v3.0.0.
Human snRNA-seq data are available via https://ngdc.cncb.ac.cn/gsa-human/ under accession number HRA006180 and via https://singlecell.charite.de/cellbrowser/pancreas/.
(3) All unique/stable reagents generated in this study will be made available from the corresponding author with a completed Materials Transfer Agreement.
(4) Numerical source data and unprocessed gels are provided with this paper. Representative images of immunoblotting, immunohistochemistry and multiplexed IF are provided. All other data supporting the findings from this study and step-by-step protocols are available from the corresponding author on reasonable request.

# Research involving human participants, their data, or biological material

Policy information about studies with human participants or human data. See also policy information about sex, gender (identity/presentation), and sexual orientation and race, ethnicity and racism.

| | |
|---|---|
| Reporting on sex and gender | The sex of six donors for human PDAC and matched adjacent pancreas with chronic pancreatitis tissues is described in Supplementary Table 1. Donors from both sexes are included in this study. |
| Reporting on race, ethnicity, or other socially relevant groupings | All available information about the donors (sex and age) is listed in Supplementary Table 1. |
| Population characteristics | See above |
| Recruitment | Human PDAC and adjacent pancreas with chronic pancreatitis tissues were obtained from surgical resections performed at Addenbrooke's hospital (Cambridge, UK). Written informed consent was acquired from patients before specimens' acquisition. Information about ethic approval is provided in the manuscript. Formalin-fixed paraffin-embedded (FFPE) specimens were bio-banked by the Human Research Tissue Bank. PDAC and chronic pancreatitis areas were annotated by a pathologist. |
| Ethics oversight | Patients' specimens were acquired and analysed under the Pancrea_tive protocol (reference: REC21/WM/0119), which was approved by the West Midlands – Black Country Research Ethics Committee. |

Note that full information on the approval of the study protocol must also be provided in the manuscript.

# Field-specific reporting

Please select the one below that is the best fit for your research. If you are not sure, read the appropriate sections before making your selection.

☒ Life sciences ☐ Behavioural & social sciences ☐ Ecological, evolutionary & environmental sciences

For a reference copy of the document with all sections, see nature.com/documents/nr-reporting-summary-flat.pdf

# Life sciences study design

All studies must disclose on these points even when the disclosure is negative.

| | |
|---|---|
| Sample size | No statistical method was used to predetermine sample size. Sample sizes of mice were informed by preliminary pilot experiments with caerulein while adhering to the 3Rs (Replacement. Reduction. Refinement) to minimize unnecessary animal use or determined based on similar prior studies showing they were sufficient for biologically meaningful conclusions (e.g. Mucciolo et al 2024, Lloyd et al 2025). Exact sample sizes are stated in the figure legends and/or figure panels, and at least 3 biological replicates were performed for each experiment. |
| Data exclusions | No data was excluded from the analyses. |
| Replication | All experimental replications, where applicable, were indicated in the main text and figure legends. |
| Randomization | Randomization was performed for mice treated with caerulein or PBS; for other experiments; randomisation was not applicable because experiments were performed with organoids, pancreatic stellate cells, mixed-fibroblasts, mesothelial cells, which were clearly grouped based on genotypes, phenotypes, and/or treatments. |
| Blinding | For animal welfare reasons and experimental design (e.g. pancreatitis and PDAC models are clearly distinguishable, to avoid swapping of organoid lines), researchers were not blinded to mouse treatment or organoid experiments during sample collection. Blinding was performed whenever possible (e.g. for flow cytometry, immunohistochemistry and multiplex immunofluorescence staining and analyses). |

# Reporting for specific materials, systems and methods

We require information from authors about some types of materials, experimental systems and methods used in many studies. Here, indicate whether each material, system or method listed is relevant to your study. If you are not sure if a list item applies to your research, read the appropriate section before selecting a response.

## Materials & experimental systems

| n/a | Involved in the study | |
|---|---|---|
| ☐ | ☒ | Antibodies |
| ☐ | ☒ | Eukaryotic cell lines |
| ☒ | ☐ | Palaeontology and archaeology |
| ☐ | ☒ | Animals and other organisms |
| ☒ | ☐ | Clinical data |
| ☒ | ☐ | Dual use research of concern |
| ☒ | ☐ | Plants |

## Methods

| n/a | Involved in the study | |
|---|---|---|
| ☒ | ☐ | ChIP-seq |
| ☐ | ☒ | Flow cytometry |
| ☒ | ☐ | MRI-based neuroimaging |

## Antibodies

Antibodies used

Western blot:
Primary antibody
1. ACTIN (8456; D18C11; Cell Signaling Technology; LOT:5; RRID:AB_10998774, dilution 1:1000)
2. p-STAT3 (9145; D3A7; Cell Signaling Technology; LOT:54; RRID:AB_2491009; dilution 1:2000; )
3. STAT3 (9139; 124H6; Cell Signaling Technology; LOT:10; RRID:AB_331757; dilution 1:1000)
4. p16 (211542; EPR20418; Abcam; LOT:1112952-21; RRID:AB_2891084; dilution 1:1000)
5. p19 (77184; E9X4Z; Cell Signaling Technology; LOT:1; dilution 1:1000)
6. SOX9 (AB5535; Merck; LOT: 4163018; RRID:AB_2239761; dilution 1:2000)
7. DPP4 (187048; EPR18215; Abcam; LOT: 1100174-2; RRID:AB_3695693; dilution 1:1000)
8. HSP90 (07-2174; Millipore; LOT: 3054621; RRID:AB_10807022; dilution 1:1000)
9. SOX4 (C15310129; Hologic Diagenode; LOT: A574-004; dilution 1:1000)
Secondary antibodies:
1. Peroxidase-AffiniPure Donkey Anti-Rabbit IgG (H+L) (711-035-152-JIR-0.5ml; Jackson ImmunoResearch Laboratories; dilution 1:5000)
2. Peroxidase-AffiniPure Donkey Anti-Mouse IgG (H+L) (715-035-150-JIR-0.5ml; Jackson ImmunoResearch Laboratories; dilution 1:5000)

Immunohistochemical analyses
Primary antibodies

1. alphaSMA (ab5694; Abcam; LOT:1038192-3; RRID:AB_2223021; dilution 1:500)
2. PDPN (127401; BioLegend; LOT:B465902; RRID:AB_1089186; dilution 1:250)
3. PDGFR-alpha (3174; Cell Signaling Technology; LOT:9; RRID:AB_2162345; dilution 1:250)
4. CK19 (TROMA-III; DSHB; LOT:11/6/25-262ug/mL; RRID:AB_2133570; dilution 1:200)
5. Ly6G (127602; BioLegend; LOT:B418822; RRID:AB_1089180; dilution 1:750)
6. F4/80 (MCA497; Serotec; LOT:154568; RRID:AB_323279; dilution 1:20)
Secondary antibodies:
1. anti-rat secondary antibody (A110-322A; Bethyl Laboratories; RRID: AB_10681533)
2. ImmPRESS[R] HRP Horse Anti-Rabbit IgG, Mouse adsorbed Polymer Detection Kit, Peroxidase (VEC-MP-7401-50; Vector)
3. ImmPRESS[R] HRP Goat Anti-Rat IgG, Mouse adsorbed Polymer Detection Kit, Peroxidase (MP-7444-15; Vector)
4. anti-Syrian hamster secondary antibody (307-006-003; Jackson ImmunoResearch)

Multiplex immunofluorescence
Primary antibodies:
1. alphaSMA (ab5694; Abcam; LOT:1038192-3; RRID:AB_2223021; dilution 1:400)
2. PDPN (127403; 8.1.1; BioLegend; LOT:B465902; RRID:AB_1134221; dilution 1:200)
3. PDGFR-alpha (3174; D1E1E; Cell Signaling Technology; LOT:9; RRID:AB_2162345; dilution 1:500)
4. FN1 (Ab2413; Abcam; LOT:1132602-1; RRID:AB_2262874; dilution 1:100)
5. CK19 (TROMA-III; DSHB; LOT:11/6/25-262ug/mL; RRID:AB_2133570; dilution 1:200)
6. SOX9 (AB5535; Merck; LOT:4163018; RRID:AB_2239761; dilution 1:2000)
7. CD34 (ab81289; EP373Y; Abcam; LOT:1071545-55; RRID:AB_1640331; dilution 1:500)
8. RUNX1 (MA5-50434; HL2211; ThermoFisher; LOT:80043346; RRID:AB_3094219; dilution 1:100)
9. CD44 (14-0441-82; IM7;ThermoFisher; LOT:3210456; RRID: AB_467246; dilution 1:300)
10. TNC (ab108930; EPR4219; Abcam; LOT:1117887-5; RRID:AB_10865908; dilution 1:200)
11. CD56 (99746; E7X9M; Cell Signaling Technology; LOT:3; RRID:AB_2868490; dilution 1:100)
12. ECAD (610182; Clone 36; BD Biosciences; LOT:5127200; RRID: AB_397581; dilution 1:1000)
13. WT1 (ab89901; CAN-R9(IHC)-56-2; Abcam; LOT:1092014-53; dilution 1:200)
14. SPP1 (AF808; R&D systems; LOT:BDO0924121; RRID:AB_2194992; dilution 1:500)
15. STAT1 (14994; Cell Signaling Technology; LOT:5; RRID:AB_2737027; dilution 1:500)
16. MUC5AC (Ab3649; Abcam; LOT:1063601-94; RRID:AB_2146844; dilution 1:500)
Secondary antibodies:
1. Anti-rabbit, anti-hamster, and anti-mouse secondary antibodies, opal dye and DAPI used were supplied in the Opal 6-Plex manual detection kit (NEL861001KT; Akoyabiosciences)
2. ImmPRESS[R] HRP Goat Anti-Rat IgG, Mouse adsorbed Polymer Detection Kit, Peroxidase (MP-7444-15; Vector)
3. ImmPRESS[R] HRP Horse Anti-Goat IgG Polymer Reagent (30116; Vector)
4. Goat anti-Syrian hamster secondary antibody (ab6892; Abcam; RRID:AB_955427)

Flow cytometry
1. CD16/CD32 (553142; 2.4G2 (RUO); BD Bioscience; LOT:4008991; RRID:AB_394657; dilution 1:50)
2. CD200-APC (123809; OX-90; Biolegend; LOT:B370920; RRID:AB_10900996; dilution 1:100)
3. I-A/I-E-BV785 (107645; M5/114.15.2; Biolegend; LOT:B409405; RRID:AB_2565977; dilution 1:100)
4. CD326 (EpCAM)-AlexaFluor 488 (118210; G8.8; Biolegend; LOT:B434047; RRID:AB_1134099; dilution 1:100)
5. PDPN-APC/Cy7 (127418; 8.1.1; Biolegend; LOT:B429687; RRID:AB_2629804; dilution 1:100)
6. CD326 (EpCAM)-APC/Cyanine7 (118217; G8.8; Biolegend; LOT:B363792; RRID: AB_1501158; dilution 1:100)
7. PDPN-PE (127408; 8.1.1; Biolegend; LOT:B366389; RRID:AB_2161928; dilution 1:100)

Validation

Whenever possible, antibodies were validated by our histology core facility using negative controls. Some of the staining patterns in our manuscript confirm previous publications:
1. Ohlund, D. et al. Distinct populations of inflammatory fibroblasts and myofibroblasts in pancreatic cancer. J Exp Med 214, 579–596 (2017).
2. Mucciolo, G. et al. EGFR-activated myofibroblasts promote metastasis of pancreatic cancer. Cancer Cell https://doi.org/10.1016/j.ccell.2023.12.002 (2023) doi:10.1016/j.ccell.2023.12.002.
3. Lloyd, E. G. et al. SMAD4 and KRAS Status Shape Cancer Cell-Stromal Crosstalk and Therapeutic Response in Pancreatic Cancer. Cancer Research https://doi.org/10.1158/0008-5472.CAN-24-2330 (2025) doi:10.1158/0008-5472.CAN-24-2330.
4. Biffi, G. et al. IL1-Induced JAK/STAT Signaling Is Antagonized by TGFbeta to Shape CAF Heterogeneity in Pancreatic Ductal Adenocarcinoma. Cancer Discov 9, 282–301 (2019).
5. Boj, S. F. et al. Organoid models of human and mouse ductal pancreatic cancer. Cell 160, 324–338 (2015).
6. Kopp, J. L. et al. Identification of Sox9-dependent acinar-to-ductal reprogramming as the principal mechanism for initiation of pancreatic ductal adenocarcinoma. Cancer Cell 22, 737–750 (2012).
7. David, C. J. et al. TGF-β Tumor Suppression through a Lethal EMT. Cell 164, 1015–1030 (2016).
8. Vilallongue, N. et al. Guidance landscapes unveiled by quantitative proteomics to control reinnervation in adult visual system. Nat Commun 13, 6040 (2022).
9. Kuonen, F. et al. c-FOS drives reversible basal to squamous cell carcinoma transition. Cell Reports 37, 109774 (2021).
10. Mulholland, E. J. et al. Epithelial GREMLIN1 disrupts intestinal epithelial-mesenchymal crosstalk to induce a wnt-dependent ectopic stem cell niche through stromal remodelling. Nat Commun 16, 5167 (2025).

## Eukaryotic cell lines

Policy information about cell lines and Sex and Gender in Research

Cell line source(s)

KPC PDAC organoids were generated from independent male (T6, GB-T13, T6LOH, T8LOH, GB-T11LOH, GB-T12LOH, GB-

| | |
|---|---|
| Cell line source(s) | T13LOH) or female (Smad4 WT and KO T69a, i.e. KP or KPdeltaS organoids) C57BL/6J-background KPC (KrasLSL-G12D/+; Trp53R172H/+; Pdx1-Cre) mice. KdeltaP PDAC organoids (T113) were derived from C57BL/6J-background KPPC (KrasLSL-G12D/+; Trp53fl/fl; Pdx1-Cre) mice.<br>KC organoid lines (PanIN3 and PanIN7) were generated from independent female and male C57BL/6J-background KC (KrasLSL-G12D/+; Pdx1-Cre) mice, respectively.<br>Normal organoids (GB-N21-23, GB-N25 and GB-N27) were generated from the healthy pancreas isolated from independent untreated male C57BL/6J mice.<br>Pancreatitis-derived organoids (GB-P1-5) were generated from the chronic pancreatitis tissues isolated from independent male C57BL/6J mice treated with caerulein (250 ng/g) twice daily for 6 weeks.<br>Murine pancreatic stellate cells (PSCs) (SV40-immortalized, C57BL/6J background, PSC4, PSC5, and PSC23) were previously described. PSC23 were isolated from male mice.<br>Murine pancreatic mixed-fibroblasts (GB-Fibro27, GB-Fibro28, GB-Fibro39, and GB-Fibro40) and pancreatic mesothelial cells (GB-PSC36-M-H11, GB-PSC36-M-G1, and GB-PSC36-M-G3) were obtained while generating additional PSC lines from male and female untreated C57BL/6J mice, respectively. Two and a half pancreata from independent mice were used for each line.<br>Murine pancreatitis mixed-fibroblasts (P-mixed-fibroblasts) were obtained while generating GB-P organoids from the chronic pancreatitis tissues isolated from independent male C57BL/6J mice treated with caerulein (250 ng/g) twice daily for 6 weeks. |
| Authentication | Authentication was performed on PSCs, pancreatic and pancreatitis mixed-fibroblasts and pancreatic mesothelial cells using the murine Short Tandem Repeat (STR) Cell Line Authentication assay. |
| Mycoplasma contamination | Mycoplasma testing for cell lines was performed prior to each freezing. The mycoplasma results were always negative. |
| Commonly misidentified lines<br>(See ICLAC register) | No commonly misidentified cell line was used in this study. |

## Animals and other research organisms

Policy information about studies involving animals; ARRIVE guidelines recommended for reporting animal research, and Sex and Gender in Research

| | |
|---|---|
| Laboratory animals | Mus musculus. Male and female C57BL/6J (strain number 632, Charles River, RRID:IMSR_JAX:000664) were purchased from the Charles River Laboratory (7-8-week-old at arrival). C57BL/6J-background KPC (KrasLSL-G12D/+; Trp53R172H/+; Pdx1-Cre) mice were bred in the Institute and palpated to follow tumour formation from two months of age. Male C57BL/6J mice were orthotopically grafted with KdeltaP or KPdeltaS PDAC organoids, female and male C57BL/6J mice were orthotopically grafted with KP PDAC organoids. Mice were palpated weekly follow tumour formation from two weeks after the transplantation until the experimental endpoint. |
| Wild animals | The study did not involve wild animals. |
| Reporting on sex | Male and female mice were used in pilot experiments of mice with treated PBS and chronic pancreatitis regimen. No significant differences were observed between male and female mice in histological analyses of both normal pancreas and chronic pancreatitis tissues. Thus, studies were carried mainly using male mice. Sex based analysis for histological analyses were not included but can be provided upon request. |
| Field-collected samples | The study did not involve samples collected from the field. |
| Ethics oversight | All animal procedures and studies were reviewed by the Cancer Research UK Cambridge Institute (CRUK-CI) AWERB, approved by the Home Office and conducted under project license number PP4778090 in accordance with relevant institutional and national guidelines and regulations. |

Note that full information on the approval of the study protocol must also be provided in the manuscript.

## Plants

| | |
|---|---|
| Seed stocks | *Report on the source of all seed stocks or other plant material used. If applicable, state the seed stock centre and catalogue number. If plant specimens were collected from the field, describe the collection location, date and sampling procedures.* |
| Novel plant genotypes | *Describe the methods by which all novel plant genotypes were produced. This includes those generated by transgenic approaches, gene editing, chemical/radiation-based mutagenesis and hybridization. For transgenic lines, describe the transformation method, the number of independent lines analyzed and the generation upon which experiments were performed. For gene-edited lines, describe the editor used, the endogenous sequence targeted for editing, the targeting guide RNA sequence (if applicable) and how the editor was applied.* |
| Authentication | *Describe any authentication procedures for each seed stock used or novel genotype generated. Describe any experiments used to assess the effect of a mutation and, where applicable, how potential secondary effects (e.g. second site T-DNA insertions, mosiacism, off-target gene editing) were examined.* |

## Flow Cytometry

### Plots

Confirm that:

☒ The axis labels state the marker and fluorochrome used (e.g. CD4-FITC).

☒ The axis scales are clearly visible. Include numbers along axes only for bottom left plot of group (a 'group' is an analysis of identical markers).

☒ All plots are contour plots with outliers or pseudocolor plots.

☒ A numerical value for number of cells or percentage (with statistics) is provided.

### Methodology

| | |
|---|---|
| Sample preparation | For P and PDAC organoid/PSC co-cultures, PDAC or P-derived organoids were cultured alone or co-cultured with 35,000 of PCSs per Matrigel dome for 3.5 days in reduced media (i.e., 5% FBS DMEM) for flow cytometric and cell sorting analyses.<br><br>For P and PDAC organoid/multi-stroma co-cultures, PDAC or P organoids were co-cultured with 30,000 stromal cells (i.e., 10,000 each of PSCs, mixed-fibroblasts, and mesothelial cells) per Matrigel dome for 3.5 days in reduced media (i.e., 5% FBS DMEM). Sorting of P or PDAC organoid/multistroma co-cultures was performed following 3.5 days culture.<br><br>Following single cell digestion of co-cultures, cells were blocked on ice with CD16/CD32 Pure 2.4G2 (1:50; 553142, BD Bioscience; RRID: AB_394657) in 2% FBS in PBS for 15 min. Then cells were stained for 30 min on ice with anti-mouse CD326 (EpCAM)-AlexaFluor 488 (1:100; 118210; G8.8; BioLegend; RRID:AB_1134099) and PDPN-PE (1:100; 127408; 8.1.1; BioLegend; RRID:AB_2161928). Cells were resuspended in PBS with DAPI. |
| Instrument | BD FACSMelody Cell Sorter. |
| Software | BD FACSChorus Software Version 3. |
| Cell population abundance | For co-culture of P or PDAC organoids with PSCs, EpCAM- AlexaFluor488+ (organoids) cells represent between 4.39% to 70.7% and PDPN-PE+ (PSCs) cell represent 28.2% to 94.9% of single live cells in co-culture experiments.<br><br>For co-culture of P, PDAC, or P-to-PDAC organoids with multi-stroma, PDPN-PE+ (multi-stroma) cells represent 89.5-95.2%, 64.8-89.8%, and 62.7-84.8% of single live cells in multi-stroma co-culture experiments, respectively. |
| Gating strategy | Single cells were identified based on Forward scatter (FSC) vs. side scatter (SSC). Then live cells were determined by DAPI-negative gating. After that, we sorted<br>1. P and PDAC organoid/PSC co-cultures<br>(1) PE-positive (i.e. with expression of PDPN), AlexaFluor 488-negative (i.e. without expression of EpCAM) PSCs and (2) PE-negative (i.e. without expression of PDPN), AlexaFluor 488-positive (i.e. with expression of EpCAM) organoids/malignant cells.<br>2. P and PDAC organoid/multi-stroma co-cultures<br>(1) PE-positive (i.e. with expression of PDPN) PSCs, mixed fibroblasts and mesothelial cells. |

☒ Tick this box to confirm that a figure exemplifying the gating strategy is provided in the Supplementary Information.
