# Supplementary material for: Multi-stromal organoid co-culture modelling reveals epithelial–fibroblast heterogeneity in pancreatic cancer and pancreatitis
Source: Nat Cell Biol. 2026 Sep 2;28(10):2119–37. doi: 10.1038/s41556-026-02057-w (PMC13661236; doi:10.1038/s41556-026-02057-w)

ED Figure 5H

p-STAT3 (top band) and ACTIN (middle band)

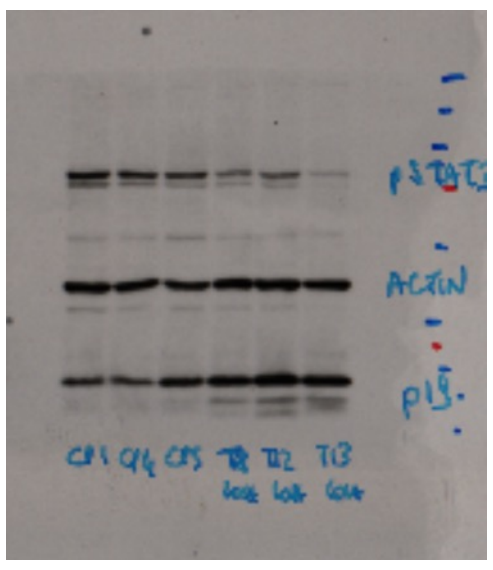

STAT3 (re-strip of p-STAT3)

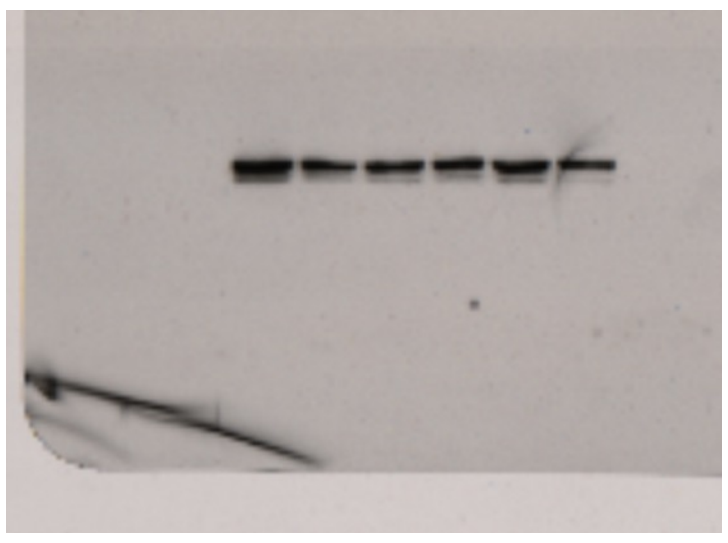

ED Figure 6H

20 sec – HSP90 (top band)

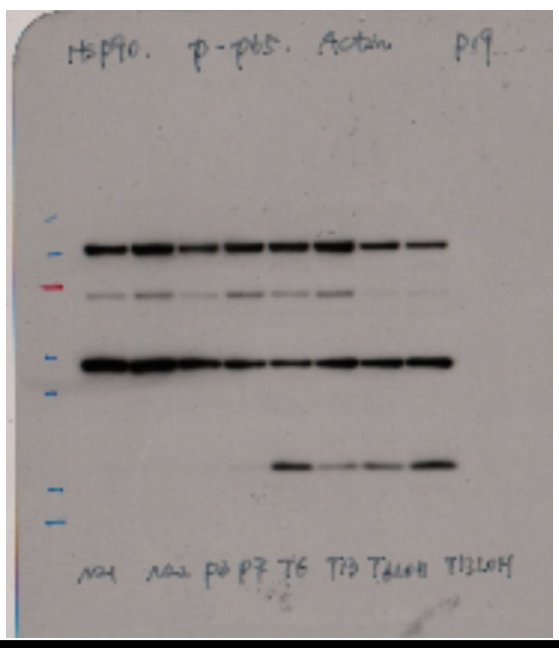

3 min – p19 (bottom band)

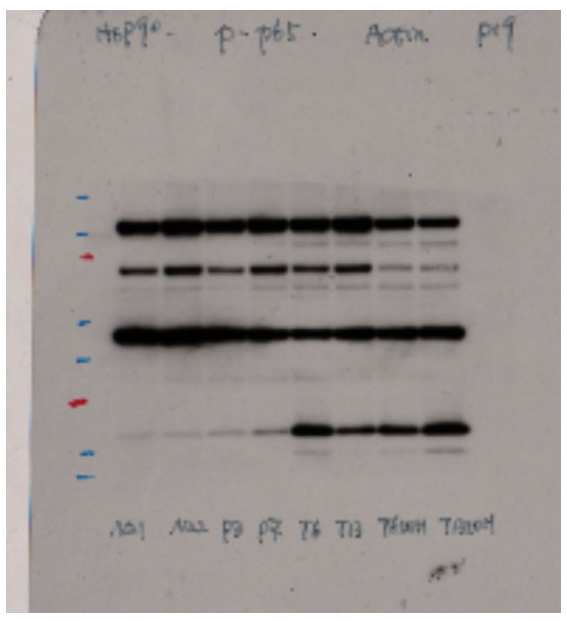

Figure 5H and 5L (same protein samples)

SOX4 and HSP90 – 5H

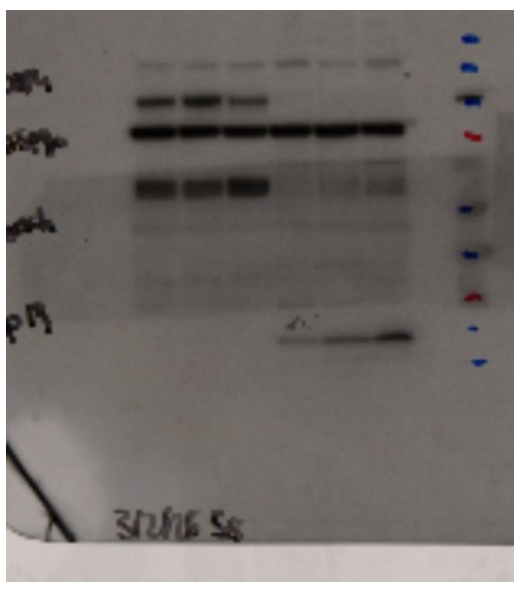

5 sec exposure

HSP90 (top band) For p19 – 5L

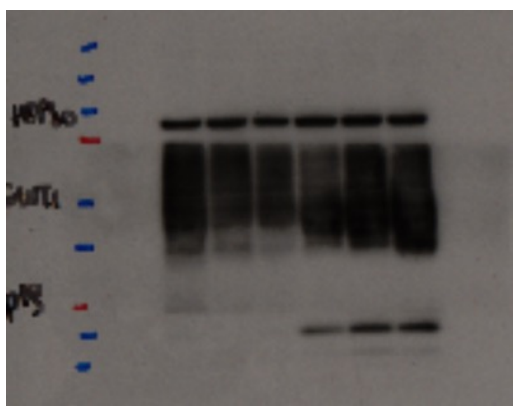

5 sec exposure

SOX9 and DPP4 – 5H

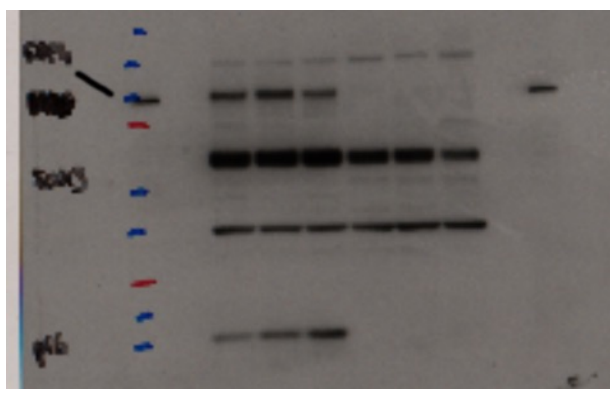

10 sec exposure

P16 – 5L

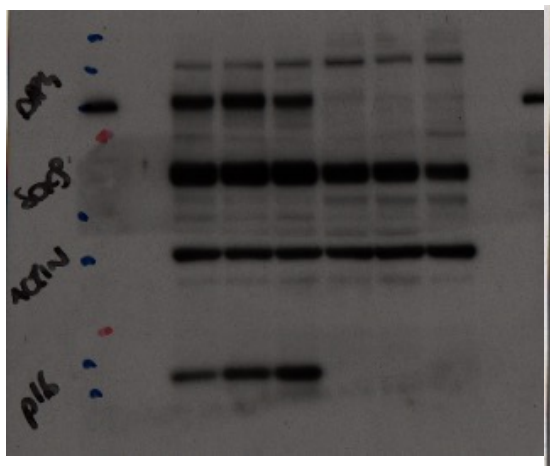

P19 – 5L

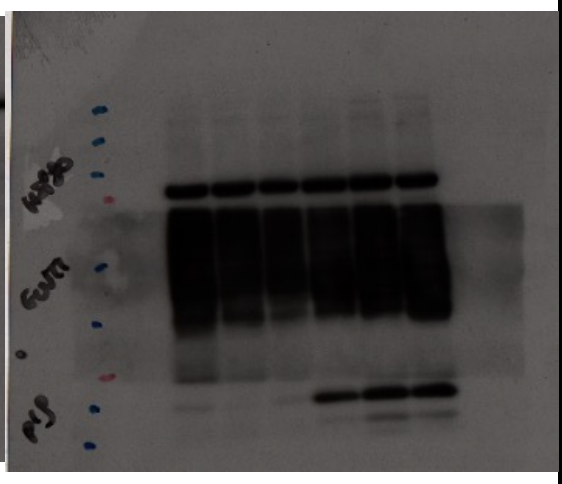

30 sec exposure

ED Figure 9C

p-STAT3 (middle band)

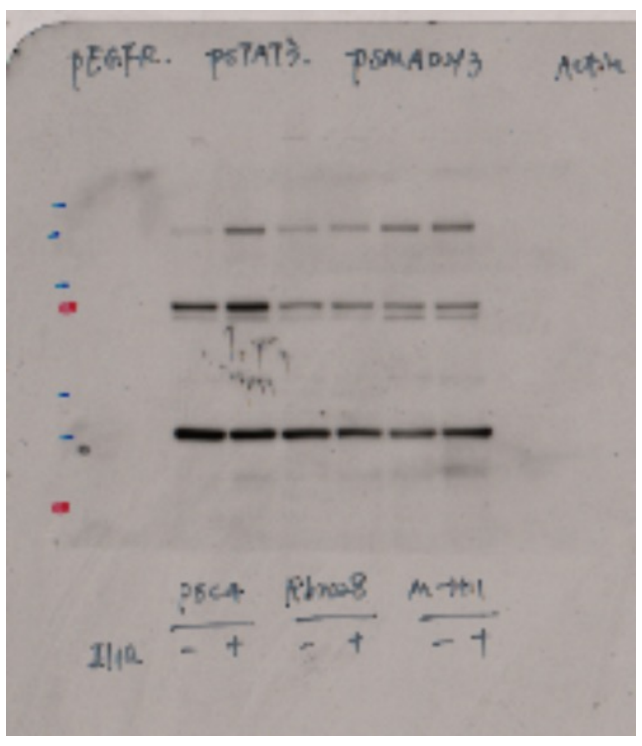

1 min exposure

ACTIN (bottom band)

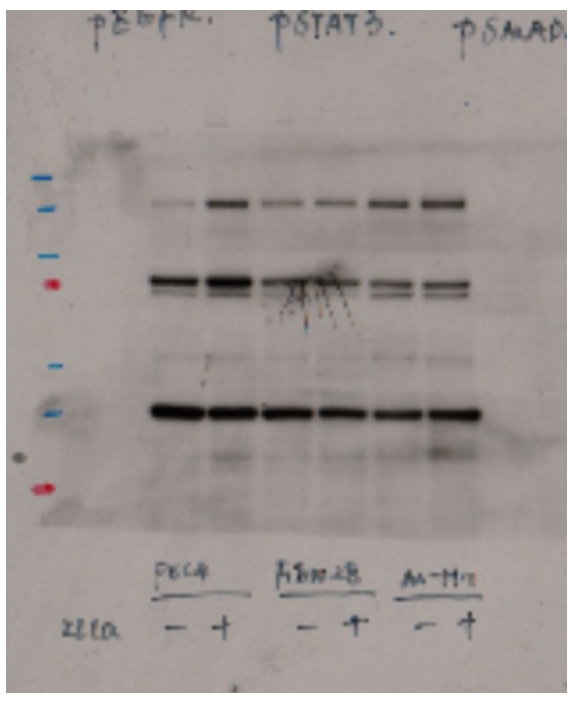

3 min exposure

STAT3 (re-strip of p-STAT3) (middle band)

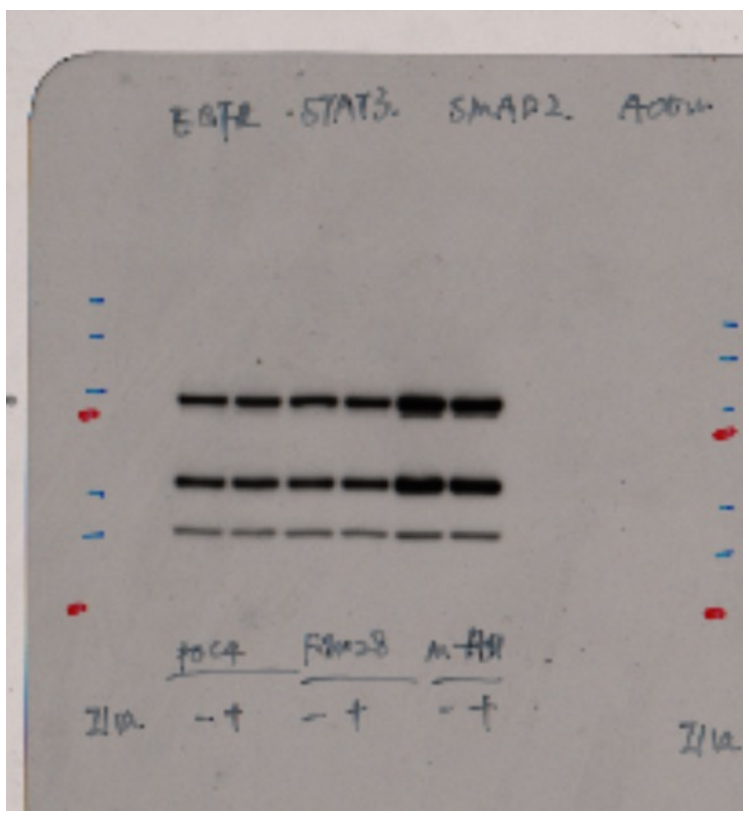

ED Figure 9D

p-STAT3 (middle band) and ACTIN (bottom band)

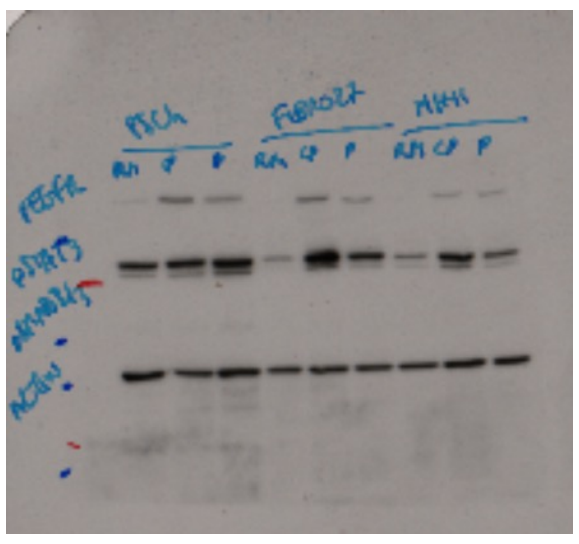

STAT3 (re-strip of p-STAT3)

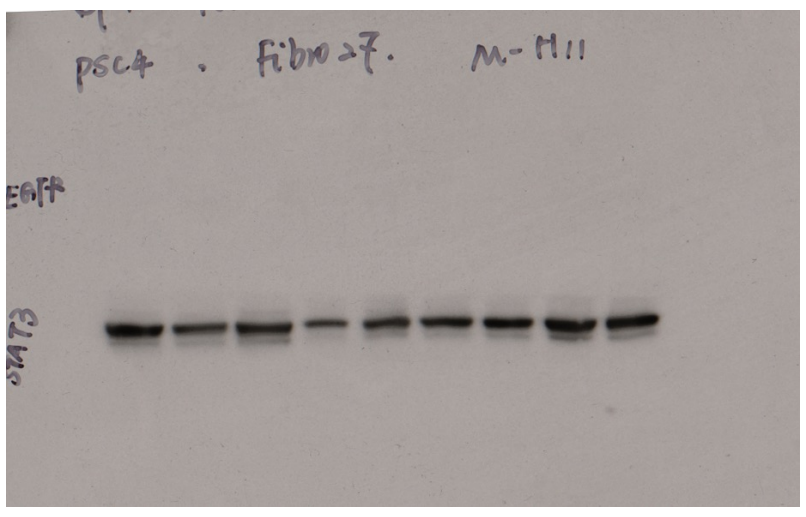

ED Figure 9G

p-STAT3 (top band)

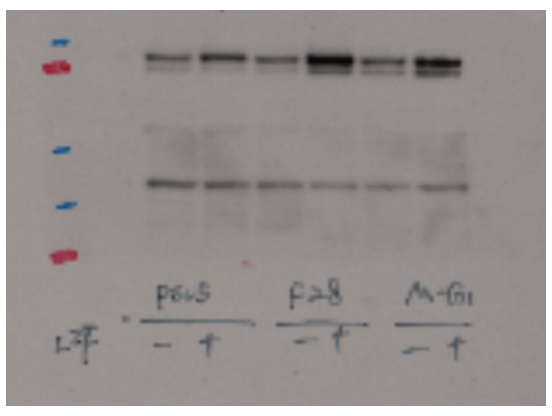

1 min exposure

ACTIN (bottom band)

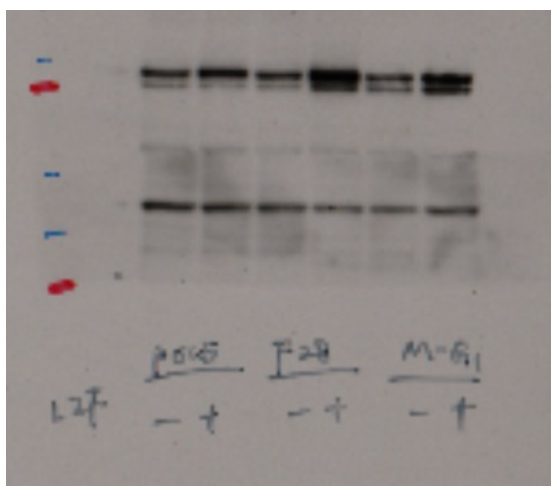

3 min exposure

STAT3 (re-strip of p-STAT3)

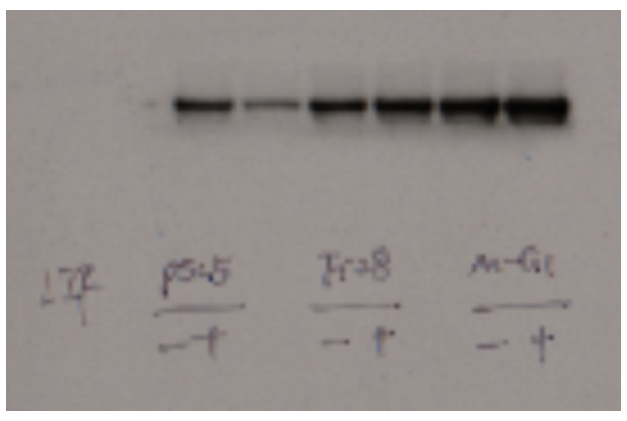

Supplement: Supplementary file 2 [file 41556_2026_2057_MOESM20_ESM.pdf]
