# Supplementary material for: Multi-stromal organoid co-culture modelling reveals epithelial–fibroblast heterogeneity in pancreatic cancer and pancreatitis
Source: Nat Cell Biol. 2026 Sep 2;28(10):2119–37. doi: 10.1038/s41556-026-02057-w (PMC13661236; doi:10.1038/s41556-026-02057-w)
Supplement: Supplementary file 3 [file 41556_2026_2057_MOESM2_ESM.pdf]

nature portfolio

Peer Review File

Multi-stromal organoid co-culture modelling reveals epithelial-fibroblast heterogeneity in pancreatic cancer and pancreatitis

Corresponding Author: Dr Giulia Biffi

This file contains all editorial decision letters in order by version, followed by all author rebuttals in order by version.

Version 0:

Decision Letter:

*Please delete the link to your author homepage if you wish to forward this email to co-authors.

Dear Dr Biffi,

Your manuscript, "Multi-stromal organoid co-cultures model pancreatic cancer and pancreatitis epithelial cell-fibroblast heterogeneity", has now been seen by 3 referees, who are experts in PDAC, stroma (referee 1); PDAC, modeling (referee 2); and PDAC (referee 3). As you will see from their comments (attached below) they find this work of potential interest, but have raised substantial concerns, which in our view would need to be addressed with considerable revisions before we can consider publication in Nature Cell Biology.

Nature Cell Biology editors discuss the referee reports in detail within the editorial team, including the chief editor, to identify key referee points that should be addressed with priority, and requests that are overruled as being beyond the scope of the current study. To guide the scope of the revisions, I have listed these points below. We are committed to providing a fair and constructive peer-review process, so please feel free to contact me if you would like to discuss any of the referee comments further.

In particular, it would be essential to:

A) Provide further characterization and comparison of pancreatitis and PDAC as questioned by the reviewers:

Reviewer 1
"The direct comparison of activated fibroblast markers in mouse models of pancreatitis and PDAC in Figures 1 and ED Figure 1 are central to the authors' claims and would benefit from a bit of clarification. Specifically, the PDPN staining in ED Figure 1N could likely be improved to reduce background—this seems worthwhile as PDPN is such a commonly used marker for activated fibroblasts or CAFs in studies of mouse pancreas. In Figure 1K, the trichrome staining is certainly convincing, but the comparison of collagen abundance between pancreatitis and PDAC may be helped by the added granularity of separately quantifying poorly and moderately or well differentiated tumor regions. As malignant ductal or glandular structures generally have more myofibroblastic density and ECM in their vicinity than poorly differentiated tumor regions, the comparison to metaplastic ductal regions in pancreatitis may show comparable collagen abundance to well/moderately differentiated PDAC but less in poorly differentiated areas. This seems a meaningful addition in line with the authors' key claims which could presumably be done by parsing histologically distinct regions of KPC tissues the authors have in hand."

"Through their analyses in Figure 2, the authors nicely display Fibro 3 and Ductal 1 as the transcriptional nearest neighbors to PDAC CAFs and malignant epithelial cells, respectively, from the pancreatitis setting. Do these populations (Fibro 3 and Ductal 1) spatially localize near each other in pancreatitis tissues? And does ligand-receptor analysis suggest that these two clusters communicate preferentially with each other? While identification of each population is a nice addition, some implication of crosstalk between these defined epithelial and stromal cells might implicate a precancerous or cancer-like niche in the setting of inflammation."

"Comparing Figure 2N to Figure 3E, 3F, and ED Figure 3F, it seems a number of fibroinflammatory pathways upregulated in PDAC epithelial cells compared to pancreatitis are reversed in the organoids (i.e., higher in pancreatitis-derived organoids). Do these signatures align specifically with the Ductal 1 signature from intact pancreatitis tissues, perhaps suggesting a preferential outgrowth of these malignant-like epithelial cells during P organoid preparation? These results do not detract from the authors' conclusions, but some further explanation of differences between epithelial states in vivo and in vitro would help frame the conclusions."

"In depicting the multi-stroma co-cultures in Figure 6, it would be meaningful to indicate the relative abundance of these cell populations using their fluorescent and cell surface markers over the course of co-culture (analogous to analyses depicted in Figure 4C & 4D). It seems reasonably likely that pancreatitis- and PDAC-derived organoids differentially select for expansion of defined mesenchymal populations, which would be helpful in interpreting these results and identifying axes for future study."

Reviewer 2
"Moreover, while pancreatitis and PDAC epithelial cells favor distinct fibroblast states, it remains unclear whether these states are modifiable. In particular, it is not addressed whether, and to what extent, PDAC cells can reprogram fibroblasts that were previously

shaped by a pancreatitis environment. This is particularly relevant given that pancreatitis is a major risk factor for PDAC. To address this gap, the authors should perform sequential co-culture experiments, in which fibroblasts are first co-cultured with pancreatitis organoids and subsequently co-cultured with PDAC organoids. Such a sequential co-culture model would more closely mimic patients who have chronic pancreatitis and later develop PDAC.
From a therapeutic standpoint, whether pancreatitis-educated fibroblasts resist or permit PDAC-driven reprogramming has major implications. Resistance would suggest that inflammatory fibroblasts in the pancreatitis environment may be protective, arguing against broad fibroblast-targeting strategies. In contrast, if pancreatitis-associated fibroblasts could be easily hijacked by PDAC cells, this would point to an early window for fibroblast-targeted therapy to prevent establishment of tumor-promoting CAFs, i.e., an interception strategy."

Reviewer 3
"While this is a well-performed cross-species comparison of murine and human epithelial – fibroblast cells in PDAC and CP, a limitation is the considerable small dataset of human samples (n=5 and 4 respectively) as well as the more uniform KPC model, thus challenging generalizability across various PDACs."

B) Strengthen the mechanistic aspect of the work as suggested by the reviewers:

Reviewer 2
"...However, the mechanistic basis of these differences remains largely correlative. The manuscript would be elevated to the level of Nature Cell Biology by deeper exploration of specific signaling interactions. For instance, can the authors identify which tumor-derived factors (e.g. TGF-β, Hedgehog, PDGF ligands) drive the myofibroblastic shift, or conversely which inflammatory signals dominate in pancreatitis co-cultures? Demonstrating causation – for example, by inhibiting a candidate pathway in co-culture and showing a shift in fibroblast phenotype – would provide more concrete mechanistic insight beyond transcriptomic correlations. At a minimum, a more detailed discussion of how epithelial cells reprogram fibroblasts (perhaps referencing known pathways like TGF-β or IL-1 signaling in CAF activation) would help contextualize the findings. Overall, the study's data only hint at important mechanisms of tumor–stroma communication but clarifying these mechanisms would elevate the impact and justify publication in Nature Cell Biology."

Reviewer 3
"This work analyses transcriptomes and respective genes and pathways at multiple levels, yet it is unclear what the identified differences in gene expression really mean. The authors argue for guiding preventative, diagnostic and therapeutic strategies for PDAC by better understanding of epithelial-fibroblast crosstalk. However, beyond the mostly transcriptomic analysis, any functional evidence for key genes/pathways to be candidates for differences between CP and PDAC fibroblast regulation is missing."

"While the authors describe four Fibro populations in pancreatitis, CAFs are not subclassified. (e.g. Fig. 2H). While the authors cite recent work on distinct CAF populations beyond myCAF and iCAF (e.g. LRRC15+ cells), no further insights are shared regarding novel useful signatures for specific fibroblast populations/phenotypes which could be of interest, and which could be validated in external data sets as well as functionally regarding their mechanistic role."

C) All other referee concerns pertaining to strengthening existing data, providing controls, methodological details, clarifications and textual changes as applicable should also be addressed.

D) Finally please pay close attention to our guidelines on statistical and methodological reporting (listed below) as failure to do so may delay the reconsideration of the revised manuscript. In particular please provide:

- a Supplementary Figure including unprocessed images of all gels/blots in the form of a multi-page pdf file. Please ensure that blots/gels are labeled and the sections presented in the figures are clearly indicated.

- a Supplementary Table including all numerical source data in Excel format, with data for different figures provided as different sheets within a single Excel file. The file should include source data giving rise to graphical representations and statistical descriptions in the paper and for all instances where the figures present representative experiments of multiple independent repeats, the source data of all repeats should be provided.

We would be happy to consider a revised manuscript that would satisfactorily address these points, unless a similar paper is published elsewhere, or is accepted for publication in Nature Cell Biology in the meantime.

When revising the manuscript please:

- ensure that it conforms to our format instructions and publication policies (see below and www.nature.com/nature/authors/).

- provide a point-by-point rebuttal to the full referee reports verbatim, as provided at the end of this letter.

- provide the completed Editorial Policy Checklist (found here https://www.nature.com/authors/policies/Policy.pdf), and Reporting Summary (found here https://www.nature.com/authors/policies/ReportingSummary.pdf). This is essential for reconsideration of the manuscript and these documents will be available to editors and referees in the event of peer review. For more information see http://www.nature.com/authors/policies/availability.html or contact me.

Nature Cell Biology is committed to improving transparency in authorship. As part of our efforts in this direction, we are now requesting that all authors identified as 'corresponding author' on published papers create and link their Open Researcher and Contributor Identifier (ORCID) with their account on the Manuscript Tracking System (MTS), prior to acceptance. ORCID helps the scientific community achieve unambiguous attribution of all scholarly contributions. You can create and link your ORCID from the home page of the MTS by clicking on 'Modify my Springer Nature account'. For more information please visit please visit www.springernature.com/orcid.

Please submit the revised manuscript files and the point-by-point rebuttal to the referee comments using this link:

Link Redacted

*This url links to your confidential home page and associated information about manuscripts you may have submitted or be reviewing for us. If you wish to forward this email to co-authors, please delete the link to your homepage.

We would like to receive a revised submission within six months. We would be happy to consider a revision even after this timeframe, however if the resubmission deadline is missed and the paper is eventually published, the submission date will be the date when the revised manuscript was received.

We hope that you will find our referees' comments, and editorial guidance helpful. Please do not hesitate to contact me if there is anything you would like to discuss.

Best wishes,

Zhe Wang

Zhe Wang, PhD
Senior Editor
Nature Cell Biology

Tel: +44 (0) 207 843 4924
email: zhe.wang@nature.com

Reviewers' Comments:

Reviewer #1 (Remarks to the Author):

In this manuscript by Li, Jihad and colleagues, the authors interrogate and compare epithelial-mesenchymal crosstalk in the contexts of pancreatitis and pancreatic cancer. The authors have performed rigorous, extensive comparisons of human and mouse disease states, together with development and analysis of a new co-culture model which captures heterocellular crosstalk in culture. This is a very timely study, as we have come a long way as a field in our appreciation of CAF heterogeneity in PDAC but have yet to meaningfully extend these findings to pancreatitis. Further, pancreatitis is a well-appreciated risk factor for PDAC, but careful cellular and molecular comparisons between these conditions have not been performed comprehensively, highlighting the novelty of this work. The molecular analyses here are poised to be hypothesis-generating and of great interest to the field in motivating functional studies of paracrine signaling in the development of pancreatitis and PDAC. However, the study is also poised to be highly impactful on its own by describing the first "multi-stroma" co-culture system capturing distinct mesenchymal cells of origin for activated fibroblasts, the first pancreatitis-derived organoid model that faithfully recapitulates the heterogeneity and complexity of the disease in intact tissues, and the first detailed comparison of the stromal reaction in human and mouse pancreatitis versus PDAC. The study also represents a meaningful conceptual advance in linking mesenchymal lineage heterogeneity to physiologically relevant heterogeneity in the epithelial compartment. A limited number of additional analyses would help to support the authors' central claims, particularly in establishing core differences between disease states across species.
Specific comments:
1. The direct comparison of activated fibroblast markers in mouse models of pancreatitis and PDAC in Figures 1 and ED Figure 1 are central to the authors' claims and would benefit from a bit of clarification. Specifically, the PDPN staining in ED Figure 1N could likely be improved to reduce background—this seems worthwhile as PDPN is such a commonly used marker for activated fibroblasts or CAFs in studies of mouse pancreas. In Figure 1K, the trichrome staining is certainly convincing, but the comparison of collagen abundance between pancreatitis and PDAC may be helped by the added granularity of separately quantifying poorly and moderately or well differentiated tumor regions. As malignant ductal or glandular structures generally have more myofibroblastic density and ECM in their vicinity than poorly differentiated tumor regions, the comparison to metaplastic ductal regions in pancreatitis may show comparable collagen abundance to well/moderately differentiated PDAC but less in poorly differentiated areas. This seems a meaningful addition in line with the authors' key claims which could presumably be done by parsing histologically distinct regions of KPC tissues the authors have in hand.
2. The snRNA-seq from mouse PDAC as shown in Figure 2A-2D appears heavily dominated by the malignant (epithelial) compartment, suggesting substantial dropout of stromal populations during sample preparation. To ensure differences between disease states are not due to bias in cell type retention during nuclear isolation, it would be helpful here to select a small number of key differentially expressed genes (or inferred differentially activated pathways) and confirm the snRNA-seq data by immunohistochemical staining on tissues, or by fibroblast isolation and qPCR.
3. Through their analyses in Figure 2, the authors nicely display Fibro 3 and Ductal 1 as the transcriptional nearest neighbors to PDAC CAFs and malignant epithelial cells, respectively, from the pancreatitis setting. Do these populations (Fibro 3 and Ductal 1) spatially localize near each other in pancreatitis tissues? And does ligand-receptor analysis suggest that these two clusters communicate preferentially with each other? While identification of each population is a nice addition, some implication of crosstalk between these defined epithelial and stromal cells might implicate a precancerous or cancer-like niche in the setting of inflammation.
4. Comparing Figure 2N to Figure 3E, 3F, and ED Figure 3F, it seems a number of fibroinflammatory pathways upregulated in PDAC epithelial cells compared to pancreatitis are reversed in the organoids (i.e., higher in pancreatitis-derived organoids). Do these signatures align specifically with the Ductal 1 signature from intact pancreatitis tissues, perhaps suggesting a preferential outgrowth of these malignant-like epithelial cells during P organoid preparation? These results do not detract from the authors' conclusions, but some further explanation of differences between epithelial states in vivo and in vitro would help frame the conclusions.
5. In depicting the multi-stroma co-cultures in Figure 6, it would be meaningful to indicate the relative abundance of these cell populations using their fluorescent and cell surface markers over the course of co-culture (analogous to analyses depicted in Figure 4C & 4D). It seems reasonably likely that pancreatitis- and PDAC-derived organoids differentially select for expansion of defined mesenchymal populations, which would be helpful in interpreting these results and identifying axes for future study.

Reviewer #2 (Remarks to the Author):

The authors investigate how pancreatic cancer versus chronic pancreatic inflammation differentially shape epithelial-stromal interactions. This study integrates analyses of human and murine pancreatitis and pancreatic ductal adenocarcinoma (PDAC) samples with novel multi-stromal organoid co-cultures that include pancreatic stellate cells (PSCs), fibroblasts, and mesothelial cells. Using these models, the authors demonstrate that malignant and inflammatory epithelial cells drive distinct fibroblast heterogeneity and crosstalk. For example, chronic pancreatitis fibroblasts are shown to express higher levels of fibrotic/inflammatory markers such as PDGFRα and podoplanin, whereas PDAC cancer-associated fibroblasts (CAFs) exhibit a more myofibroblastic phenotype with elevated αSMA expression. The multi-stromal organoid co-cultures successfully recapitulate many in vivo gene expression features of pancreatitis and PDAC stromal cells. Cross-species single-cell RNA sequencing comparisons indicate that a combination of these in vitro and in vivo systems better captures human disease signatures than traditional models, and they reveal condition-specific stromal reprogramming signals (e.g. differential PDGF, TGF-β, NF-κB pathway activation). Notably, the authors infer that different stromal cell origins (PSC-derived vs. mesothelial-derived fibroblasts) preferentially contribute to distinct fibroblast subpopulations in PDAC and pancreatitis. Overall, this comprehensive study provides useful insights into how malignant vs. inflammatory epithelial cells reprogram the surrounding stromal compartment, and it offers a complementary platform to identify disease-specific fibroblast markers and epithelial-stromal dependencies.
Major Comments
The comparative approach between PDAC and pancreatitis models is novel, and it yields important insights into epithelial-stromal crosstalk. In particular, the finding that pancreatic cancer organoids induce a more myofibroblastic, collagen-producing fibroblast state (with reduced interferon-signaling) compared to pancreatitis organoids is compelling. This indicates that cancer cells send distinct cues to stromal cells. However, the mechanistic basis of these differences remains largely correlative. The manuscript would be elevated to the level of Nature Cell Biology by deeper exploration of specific signaling interactions. For instance, can the authors identify which tumor-derived factors (e.g. TGF-β, Hedgehog, PDGF ligands) drive the myofibroblastic shift, or conversely which inflammatory signals dominate in pancreatitis co-cultures? Demonstrating causation – for example, by inhibiting a candidate pathway in co-culture and showing a shift in fibroblast phenotype – would provide more concrete mechanistic insight beyond transcriptomic correlations. At a minimum, a more detailed discussion of how epithelial cells reprogram fibroblasts (perhaps referencing known pathways like TGF-β or IL-1 signaling in CAF activation) would help contextualize the findings. Overall, the study's data only hint at important mechanisms of tumor–stroma communication but clarifying these mechanisms would elevate the impact and justify publication in Nature Cell Biology.

Moreover, while pancreatitis and PDAC epithelial cells favor distinct fibroblast states, it remains unclear whether these states are modifiable. In particular, it is not addressed whether, and to what extent, PDAC cells can reprogram fibroblasts that were previously shaped by a pancreatitis environment. This is particularly relevant given that pancreatitis is a major risk factor for PDAC. To address this gap, the authors should perform sequential co-culture experiments, in which fibroblasts are first co-cultured with pancreatitis organoids and subsequently co-cultured with PDAC organoids. Such a sequential co-culture model would more closely mimic patients who have chronic pancreatitis and later develop PDAC.
From a therapeutic standpoint, whether pancreatitis-educated fibroblasts resist or permit PDAC-driven reprogramming has major implications. Resistance would suggest that inflammatory fibroblasts in the pancreatitis environment may be protective, arguing against broad fibroblast-targeting strategies. In contrast, if pancreatitis-associated fibroblasts could be easily hijacked by PDAC cells, this would point to an early window for fibroblast-targeted therapy to prevent establishment of tumor-promoting CAFs, i.e., an interception strategy.

The experimental design is comprehensive, employing both in vivo mouse models and in vitro organoid co-cultures, as well as human tissue data. The use of multiple independent organoid lines (five pancreatitis-derived organoid lines and two PDAC organoid lines) supports reproducibility. That said, the authors should ensure that all key findings are supported by adequate biological replicates and statistical analysis. For example, when quantifying fibroblast versus epithelial markers in co-cultures or in mice, are results consistent across different organoid lines and animals? In the co-culture experiments, it appears that pancreatitis organoids had a higher fibroblast-to-epithelial ratio than PDAC organoids – it would be helpful to report the replicate numbers and variance for such observations. Additionally, the study might benefit from including a normal pancreas baseline. While the focus is on disease vs. inflammation, showing how "normal" organoid–stroma interactions look would contextualize the changes seen in pancreatitis and PDAC. Minor point: please clarify in the text whether "fibroblasts" (as opposed to PSCs) refers to pancreatic fibroblasts isolated from healthy or other sources.

A strength of the study is the integration of mouse and human data to demonstrate relevance of the models. The authors show that many fibroblast gene signatures from human pancreatitis and PDAC are captured in the corresponding mouse models, though the overlap is far from complete (only ~28% of human PDAC CAF markers overlap with mouse CAF markers, vs ~54% overlap for pancreatitis fibroblast markers). This partial concordance should be better highlighted as it suggests that, while the murine models mimic broad patterns, certain human-specific fibroblast programs are missing. As such the manuscript would benefit from a more nuanced discussion of these species differences. For instance, are there fibroblast subtypes present in human PDAC that mice lack, or vice versa? The authors conclude that the mouse models "significantly capture" human fibroblast markers, which is fair, but they should also acknowledge the limitations – e.g., the 72% of human PDAC CAF genes that did not overlap. It would be constructive to discuss possible reasons for the disparities … differences in disease stage, immune context, or technical factors in single-cell data (the immune issue is perhaps the most impt to emphasize). Encouragingly, the cross-species analysis extends to epithelial cells, identifying analogues of "Ductal 1" vs "Ductal 2" populations, which strengthens the validity of the organoid approach. Overall, the inclusion of human data increases the translational relevance of the findings, but the authors should avoid over-interpretation of the cross-species concordance and should clarify where the models might fall short of human biology as some may interpret the message as these models would replace other types of models … which they do not … all models bring visibility to the biological complexity of human disease. To conclude otherwise is somewhat naïve.

That said, the use of multi-stromal organoid co-cultures (including PSCs, mesothelial cells, and fibroblasts) is innovative and goes beyond simpler two-cell type models. This approach clearly improved the recapitulation of in vivo fibroblast heterogeneity – the authors show that multi-stroma co-cultures capture pancreatitis and PDAC fibroblast features better than organoid cultures with PSCs alone. By incorporating multiple stromal sources, the authors also explore the intriguing idea that different CAF subpopulations may derive from different lineage origins (e.g. a mesothelial-derived subset vs. a PSC-derived subset). The data (e.g., transcriptional overlap of certain "Fibro" clusters with mesothelial signatures at ~85%) indeed suggest a mesothelial origin for some fibroblasts, consistent with recent lineage-tracing studies. However, the inference of distinct stromal origin contributions to CAF subtypes, while exciting, should be

interpreted with great caution. In the absence of lineage tracing or specific in vivo fate-mapping, the link between a fibroblast's in vitro behavior and its origin remains indirect. The authors need to temper these conclusions – for example, phrasing that mesothelial cells "can give rise to" certain fibroblast subsets is an over-interpretation of the correlative data. It would suffice to say the results support the possibility of multiple origins. Additionally, the authors should discuss the limitations of the multi-stromal model: despite including three stromal cell types, it is critical to acknowledge that the model still omits immune and endothelial components that are known to influence fibroblast activation. The co-cultures were conducted for only ~3.5 days, which may not fully capture long-term differentiation or the maintenance of certain CAF phenotypes. Acknowledging these caveats would provide a balanced perspective on how closely the in vitro system can mimic the complex tumor microenvironment. More specifically, while immune signatures and inflammatory fibroblasts are discussed extensively (e.g. interferon response) in this study, immune cells are not directly incorporated into the co-culture model systems. Therefore, fibroblast subsets that depend on immune microenvironment may be missing or under-represented. The authors should acknowledge that the fibroblast states observed in their co-culture models may differ from those in immune microenvironment.

The manuscript touches on the translational implications of distinguishing fibroblast responses in chronic pancreatitis versus PDAC. This is a key point – identifying stromal differences could have diagnostic or therapeutic relevance. For example, the finding that pancreatitis is enriched in PDPN+/PDGFRα+ fibroblasts whereas PDAC has more αSMA+ myofibroblasts might be leveraged as a biomarker signature to help pathologists distinguish cancer from benign inflammation in preoperative biopsies. The authors also note distinct ligand–receptor interactions (such as PDGF and TGF-β signaling pathways) that are preferentially active in one condition over the other. One would like to know if these pathways could be targeted: e.g., could inhibiting TGF-β or PDGF signaling selectively alter the tumor-associated stroma without impairing normal injury repair? While such therapeutic experiments are beyond the scope of this study, the authors should expand their discussion on how the insights gained might guide clinical strategies. For instance, they could discuss whether any of the identified gene markers or pathways have existing inhibitors or if they correlate with patient outcomes in PDAC. Additionally, the co-culture platform itself could be a valuable tool for drug testing in the future – this could be mentioned as a forward-looking statement. Emphasizing the clinical context (e.g., chronic pancreatitis patients are at elevated risk for PDAC) and how understanding stromal reprogramming might inform surveillance or prevention would also strengthen the impact. In summary, the findings are potentially relevant to improving PDAC diagnosis and treatment, but the manuscript should do a slightly better job at articulating these real-world connections so that a broad Nature Cell Biology audience appreciates the significance.

Minor Comments
The KPC PDAC model used in this study is driven by KRAS G12D expression and Trp53 LOH and R172H gain of function mutation. However, the pancreatitis model is driven by caerulein treatment and lack of genetic drivers. The author should acknowledge this limitation and discuss the extent to which these genetic drivers may induce fibroblast reprogramming.

In this study, fibroblast subsets are extensively characterized by transcriptomic analysis without functional characterization (e.g., ECM deposition for myCAFs, cytokine secretion for iCAFs). Thus, it is less clear whether the observed transcriptional differences of fibroblasts translate into biologically meaningful differences. The authors should validate the function of fibroblast subsets at minimal by IHC analysis.

The terminology for fibroblast subpopulations should be clarified for readers. Throughout the text, terms like "PSCs," "fibroblasts," "CAFs," and "mesothelial cells" are all used – sometimes interchangeably in co-cultures. It would help to explicitly define each cell source (e.g., PSCs isolated from pancreas, vs. "fibroblasts" from another tissue or an immortalized line) in the Methods or Results. This will prevent confusion, especially when interpreting which cell type is driving a given effect in co-culture.

Please provide details on the sample sizes and replicates for key experiments.

Some of the figure presentations could be improved for clarity … labeling, font sizes.

Line 153: "FapHigh Fibro 3 subset was most similar to CAFs" should read "……most similar to PDAC CAFs.

Overall, the manuscript presents a wealth of interesting data but also raises several questions that should be addressed. I recommend a major revision prior to publication in Nature Cell Biology. The central topic of how stromal-epithelial interactions differ between cancer and inflammation is highly relevant and the experimental framework is strong. However, as detailed above, there are significant issues regarding mechanistic depth, data interpretation, and presentation that need improvement. With revisions that address the major comments (particularly providing clearer mechanistic links and acknowledging limitations), I would strongly support the manuscript for NCB as the work has potential impact in the pancreatic cancer and stromal biology field.

Reviewer #3 (Remarks to the Author):

In this manuscript, Li et al investigate the interaction and heterogeneity of fibroblasts communicating with either malignant (PDAC) or inflammatory (chronic pancreatitis, CP) epithelial cells. Overall, this is a timely approach given the increasingly recognized role of fibroblasts contributing significantly to disease phenotypes. As such, the authors present a cross-species cross-disease comparative approach dissecting the (largely transcriptomic) characteristics of epithelial and stromal cells ex vivo and in vitro using organoid-based model systems.

While this is a comprehensive analysis of (mainly) various in vitro models, the usefulness or key novel insights remain a bit elusive. Specific issues are:

- This work analyses transcriptomes and respective genes and pathways at multiple levels, yet it is unclear what the identified differences in gene expression really mean. The authors argue for guiding preventative, diagnostic and therapeutic strategies for PDAC by better understanding of epithelial-fibroblast crosstalk. However, beyond the mostly transcriptomic analysis, any functional evidence for key genes/pathways to be candidates for differences between CP and PDAC fibroblast regulation is missing.
- Overall, the methodology and quality of data and its representation is well described and performed. In some experiments, the sample

size analyzed is considerable small (e.g. n=2 with 2 technical replicates in quite some experimental approaches).
- While this is a well-performed cross-species comparison of murine and human epithelial – fibroblast cells in PDAC and CP, a limitation is the considerable small dataset of human samples (n=5 and 4 respectively) as well as the more uniform KPC model, thus challenging generalizability across various PDACs.
- Establishment and thorough transcriptomic characterization of organoid co-culture models (especially for CP) is an advancement. Yet the question remains how much such a model reflects the very heterogeneous nature of PDAC (e.g. hypoxia-rich and -poor areas, metastatic lesions vs. primary, etc). While this manuscript can certainly not address many of them in detail, the very descriptive listing of genes and pathways and their overlap (or not) between the used models may fall short of convincingly showing an advance for the model systems in reflecting important disease contributions.
- While the authors describe four Fibro populations in pancreatitis, CAFs are not subclassified. (e.g. Fig. 2H). While the authors cite recent work on distinct CAF populations beyond myCAF and iCAF (e.g. LRRC15+ cells), no further insights are shared regarding novel useful signatures for specific fibroblast populations/phenotypes which could be of interest, and which could be validated in external data sets as well as functionally regarding their mechanistic role.
- How do classical and basal-like PDAC cells respectively interact with (and reprogram?) the different fibroblast populations in the co-culture model?
- Do the authors consider transdifferentiation e.g. of epithelial (or mesothelial) cells into distinct fibroblast populations to occur? A robust cell-lineage approach would be of interest to further clarify the extent of such here.

GUIDELINES FOR MANUSCRIPT SUBMISSION TO NATURE CELL BIOLOGY

READABILITY OF MANUSCRIPTS – Nature Cell Biology is read by cell biologists from diverse backgrounds, many of whom are not native English speakers. Authors should aim to communicate their findings clearly, explaining technical jargon that might be unfamiliar to non-specialists, and avoiding non-standard abbreviations. Titles and abstracts should concisely communicate the main findings of the study, and the background, rationale, results and conclusions should be clearly explained in the manuscript in a manner accessible to a broad cell biology audience. Nature Cell Biology uses British spelling.

MANUSCRIPT FORMAT – please follow the guidelines listed in our Guide to Authors regarding manuscript formats at Nature Cell Biology.

TITLE – should be no more than 100 characters including spaces, without punctuation and avoiding technical terms, abbreviations, and active verbs..

AUTHOR NAMES – should be given in full.

AUTHOR AFFILIATIONS – should be denoted with numerical superscripts (not symbols) preceding the names. Full addresses should be included, with US states in full and providing zip/post codes. The corresponding author is denoted by: "Correspondence should be addressed to [initials]."

ABSTRACT AND MAIN TEXT – please follow the guidelines that are specific to the format of your manuscript, as listed in our Guide to Authors (http://www.nature.com/ncb/pdf/ncb_gta.pdf) Briefly, Nature Cell Biology Articles, Resources and Technical Reports have 3500 words, including a 150 word abstract, and the main text is subdivided in Introduction, Results, and Discussion sections. Nature Cell Biology Letters have up to 2500 words, including a 180 word introductory paragraph (abstract), and the text is not subdivided in sections.

ACKNOWLEDGEMENTS – should be kept brief. Professional titles and affiliations are unnecessary. Grant numbers can be listed.

AUTHOR CONTRIBUTIONS – must be included after the Acknowledgements, detailing the contributions of each author to the paper (e.g. experimental work, project planning, data analysis etc.). Each author should be listed by his/her initials.

FINANCIAL AND NON-FINANCIAL COMPETING INTERESTS – the authors must include one of three declarations: (1) that they have no financial and non-financial competing interests; (2) that they have financial and non-financial competing interests; or (3) that they decline to respond, after the Author Contributions section. This statement will be published with the article, and in cases where financial and non-financial competing interests are declared, these will be itemized in a web supplement to the article. For further details please see https://www.nature.com/licenceforms/nrg/competing-interests.pdf.

REFERENCES – are limited to a total of 70 for Articles, Resources, Technical Reports; and 40 for Letters. This includes references in the main text and Methods combined. References must be numbered sequentially as they appear in the main text, tables and figure legends and Methods and must follow the precise style of Nature Cell Biology references. References only cited in the Methods should be numbered consecutively following the last reference cited in the main text. References only associated with Supplementary Information (e.g. in supplementary legends) do not count toward the total reference limit and do not need to be cited in numerical continuity with references in the main text. Only published papers can be cited, and each publication cited should be included in the numbered reference list, which should include the manuscript titles. Footnotes are not permitted.

METHODS – Nature Cell Biology publishes methods online. The methods section should be provided as a separate Word document, which will be copyedited and appended to the manuscript PDF, and incorporated within the HTML format of the paper.

Methods should be written concisely, but should contain all elements necessary to allow interpretation and replication of the results. As a

guideline, Methods sections typically do not exceed 3,000 words. The Methods should be divided into subsections listing reagents and techniques. When citing previous methods, accurate references should be provided and any alterations should be noted. Information must be provided about: antibody dilutions, company names, catalogue numbers and clone numbers for monoclonal antibodies; sequences of RNAi and cDNA probes/primers or company names and catalogue numbers if reagents are commercial; cell line names, sources and information on cell line identity and authentication. Animal studies and experiments involving human subjects must be reported in detail, identifying the committees approving the protocols. For studies involving human subjects/samples, a statement must be included confirming that informed consent was obtained. Statistical analyses and information on the reproducibility of experimental results should be provided in a section titled "Statistics and Reproducibility".

All Nature Cell Biology manuscripts submitted on or after March 21 2016 must include a Data availability statement at the end of the Methods section. For Springer Nature policies on data availability see http://www.nature.com/authors/policies/availability.html; for more information on this particular policy see http://www.nature.com/authors/policies/data/data-availability-statements-data-citations.pdf. The Data availability statement should include:

• Accession codes for primary datasets (generated during the study under consideration and designated as "primary accessions") and secondary datasets (published datasets reanalysed during the study under consideration, designated as "referenced accessions"). For primary accessions data should be made public to coincide with publication of the manuscript. A list of data types for which submission to community-endorsed public repositories is mandated (including sequence, structure, microarray, deep sequencing data) can be found here http://www.nature.com/authors/policies/availability.html#data.

• Unique identifiers (accession codes, DOIs or other unique persistent identifier) and hyperlinks for datasets deposited in an approved repository, but for which data deposition is not mandated (see here for details http://www.nature.com/sdata/data-policies/repositories).

• At a minimum, please include a statement confirming that all relevant data are available from the authors, and/or are included with the manuscript (e.g. as source data or supplementary information), listing which data are included (e.g. by figure panels and data types) and mentioning any restrictions on availability.

• If a dataset has a Digital Object Identifier (DOI) as its unique identifier, we strongly encourage including this in the Reference list and citing the dataset in the Methods.

We recommend that you upload the step-by-step protocols used in this manuscript to protocols.io. More details can found at https://www.protocols.io/help/publish-articles.

DISPLAY ITEMS – main display items are limited to 6-8 main figures and/or main tables for Articles, Resources, Technical Reports; and 5 main figures and/or main tables for Letters. For Supplementary Information see below.

FIGURES – Colour figure publication costs $600 for the first, and $300 for each subsequent colour figure. All panels of a multi-panel figure must be logically connected and arranged as they would appear in the final version. Unnecessary figures and figure panels should be avoided (e.g. data presented in small tables could be stated briefly in the text instead).

All imaging data should be accompanied by scale bars, which should be defined in the legend.
Cropped images of gels/blots are acceptable, but need to be accompanied by size markers, and to retain visible background signal within the linear range (i.e. should not be saturated). The boundaries of panels with low background have to be demarked with black lines. Splicing of panels should only be considered if unavoidable, and must be clearly marked on the figure, and noted in the legend with a statement on whether the samples were obtained and processed simultaneously. Quantitative comparisons between samples on different gels/blots are discouraged; if this is unavoidable, it should only be performed for samples derived from the same experiment with gels/blots were processed in parallel, which needs to be stated in the legend.

Figures should be provided at approximately the size that they are to be printed at (single column is 86 mm, double column is 170 mm) and should not exceed an A4 page (8.5 x 11"). Reduction to the scale that will be used on the page is not necessary, but multi-panel figures should be sized so that the whole figure can be reduced by the same amount at the smallest size at which essential details in each panel are visible. In the interest of our colour-blind readers we ask that you avoid using red and green for contrast in figures. Replacing red with magenta and green with turquoise are two possible colour-safe alternatives. Lines with widths of less than 1 point should be avoided. Sans serif typefaces, such as Helvetica (preferred) or Arial should be used. All text that forms part of a figure should be rewritable and removable.

We accept files from the following graphics packages in either PC or Macintosh format:

- For line art, graphs, charts and schematics we prefer Adobe Illustrator (.AI), Encapsulated PostScript (.EPS) or Portable Document Format (.PDF). Files should be saved or exported as such directly from the application in which they were made, to allow us to restyle them according to our journal house style.

- We accept PowerPoint (.PPT) files if they are fully editable. However, please refrain from adding PowerPoint graphical effects to objects, as this results in them outputting poor quality raster art. Text used for PowerPoint figures should be Helvetica (preferred) or Arial.

- We do not recommend using Adobe Photoshop for designing figures, but we can accept Photoshop generated (.PSD or .TIFF) files only if each element included in the figure (text, labels, pictures, graphs, arrows and scale bars) are on separate layers. All text should be editable in 'type layers' and line-art such as graphs and other simple schematics should be preserved and embedded within 'vector smart objects' - not flattened raster/bitmap graphics.

- Some programs can generate Postscript by 'printing to file' (found in the Print dialogue). If using an application not listed above, save the file in PostScript format or email our Art Editor, Allen Beattie for advice (a.beattie@nature.com).

Regardless of format, all figures must be vector graphic compatible files, not supplied in a flattened raster/bitmap graphics format, but

should be fully editable, allowing us to highlight/copy/paste all text and move individual parts of the figures (i.e. arrows, lines, x and y axes, graphs, tick marks, scale bars etc.). The only parts of the figure that should be in pixel raster/bitmap format are photographic images or 3D rendered graphics/complex technical illustrations.

All placed images (i.e. a photo incorporated into a figure) should be on a separate layer and independent from any superimposed scale bars or text. Individual photographic images must be a minimum of 300+ DPI (at actual size) or kept constant from the original picture acquisition and not decreased in resolution post image acquisition. All colour artwork should be RGB format.

FIGURE LEGENDS – must not exceed 350 words for each figure to allow fit on a single printed NCB page together with the figure. They must include a brief title for the whole figure, and short descriptions of each panel with definitions of the symbols used, but without detailing methodology.

TABLES – main tables should be provided as individual Word files, together with a brief title and legend. For supplementary tables see below.

SUPPLEMENTARY INFORMATION – Supplementary information is material directly relevant to the conclusion of a paper, but which cannot be included in the printed version in order to keep the manuscript concise and accessible to the general reader. Supplementary information is an integral part of a Nature Cell Biology publication and should be prepared and presented with as much care as the main display item, but it must not include non-essential data or text, which may be removed at the editor's discretion. All supplementary material is fully peer-reviewed and published online as part of the HTML version of the manuscript. Supplementary Figures and Supplementary Notes are appended at the end of the main PDF of the published manuscript.

Supplementary items should relate to a main text figure, wherever possible, and should be mentioned sequentially in the main manuscript, designated as Supplementary Figure, Table, Video, or Note, and numbered continuously (e.g. Supplementary Figure 1, Supplementary Figure 2, Supplementary Table 1, Supplementary Table 2 etc.).

Unprocessed scans of all key data generated through electrophoretic separation techniques need to be presented in a supplementary figure that should be labelled and numbered as the final supplementary figure, and should be mentioned in every relevant figure legend. This figure does not count towards the total number of figures and is the only figure that can be displayed over multiple pages, but should be provided as a single file, in PDF or TIFF format. Data in this figure can be displayed in a relatively informal style, but size markers and the figures panels corresponding to the presented data must be indicated.

The total number of Supplementary Figures (not including the "unprocessed scans" Supplementary Figure) should not exceed the number of main display items (figures and/or tables (see our Guide to Authors and March 2012 editorial http://www.nature.com/ncb/authors/submit/index.html#suppinfo; http://www.nature.com/ncb/journal/v14/n3/index.html#ed). No restrictions apply to Supplementary Tables or Videos, but we advise authors to be selective in including supplemental data.

Each Supplementary Figure should be provided as a single page and as an individual file in one of our accepted figure formats and should be presented according to our figure guidelines (see above). Supplementary Tables should be provided as individual Excel files. Supplementary Videos should be provided as .avi or .mov files up to 50 MB in size. Supplementary Figures, Tables and Videos much be accompanied by a separate Word document including titles and legends.

GUIDELINES FOR EXPERIMENTAL AND STATISTICAL REPORTING

REPORTING REQUIREMENTS – To improve the quality of methods and statistics reporting in our papers we have recently revised the reporting checklist we introduced in 2013. We are now asking all life sciences authors to complete two items: an Editorial Policy Checklist (found here https://www.nature.com/authors/policies/Policy.pdf) that verifies compliance with all required editorial policies and a reporting summary (found here https://www.nature.com/authors/policies/ReportingSummary.pdf) that collects information on experimental design and reagents. These documents are available to referees to aid the evaluation of the manuscript. Please note that these forms are dynamic 'smart pdfs' and must therefore be downloaded and completed in Adobe Reader. We will then flatten them for ease of use by the reviewers. If you would like to reference the guidance text as you complete the template, please access these flattened versions at http://www.nature.com/authors/policies/availability.html.

STATISTICS – Wherever statistics have been derived the legend needs to provide the n number (i.e. the sample size used to derive statistics) as a precise value (not a range), and define what this value represents. Error bars need to be defined in the legends (e.g. SD, SEM) together with a measure of centre (e.g. mean, median). Box plots need to be defined in terms of minima, maxima, centre, and percentiles. Ranges are more appropriate than standard errors for small data sets. Wherever statistical significance has been derived, precise p values need to be provided and the statistical test used needs to be stated in the legend. Statistics such as error bars must not be derived from n<3. For sample sizes of n<5 please plot the individual data points rather than providing bar graphs. Deriving statistics from technical replicate samples, rather than biological replicates is strongly discouraged. Wherever statistical significance has been derived, precise p values need to be provided and the statistical test stated in the legend.

Information on how many times each experiment was repeated independently with similar results needs to be provided in the legends and/or Methods for all experiments, and in particular wherever representative experiments are shown.

We strongly recommend the presentation of source data for graphical and statistical analyses as a separate Supplementary Table, and request that source data for all independent repeats are provided when representative experiments of multiple independent repeats, or averages of two independent experiments are presented. This supplementary table should be in Excel format, with data for different figures provided as different sheets within a single Excel file. It should be labelled and numbered as one of the supplementary tables,

titled “Statistics Source Data”, and mentioned in all relevant figure legends.

--------- Please don't hesitate to contact NCB@nature.com should you have queries about any of the above requirements ---------

Version 1:

Decision Letter:

Our ref: NCB-A60487A

12th June 2026

Dear Dr. Biffi,

Thank you for submitting your revised manuscript "Multi-stromal organoid co-cultures model pancreatic cancer and pancreatitis epithelial-fibroblast heterogeneity" (NCB-A60487A). It has now been seen by the original referees and their comments are below. The reviewers find that the paper has improved in revision, and therefore we'll be happy in principle to publish it in Nature Cell Biology, pending minor revisions to satisfy the referees' final requests and to comply with our editorial and formatting guidelines.

If the current version of your manuscript is in a PDF format, please email us a copy of the file in an editable format (Microsoft Word or LaTex)-- we can not proceed with PDFs at this stage.

We are now performing detailed checks on your paper and will send you a checklist detailing our editorial and formatting requirements in a couple of weeks. Please do not upload the final materials and make any revisions until you receive this additional information from us.

Meanwhile, please modify the manuscript file and figures according to editorial guidelines below:
1. For better legibility, please ensure that all figures adhere to a maximum page size of roughly 180mm wide x 200mm high to fit standard page format and use a font size of no smaller than 7pt Arial or Helvetica throughout the figures.
2. Please upload the method to protocols.io, and cite it early in the paper when introducing the method and throughout where appropriate. Please also mention it in the Data Availability Statement.
3. Numerical source data and unprocessed gels should be provided a separate file, not as Supplementary Table or Supplementary Figure.

Thank you again for your interest in Nature Cell Biology Please do not hesitate to contact me if you have any questions.

Sincerely,

Zhe Wang, PhD
Senior Editor
Nature Cell Biology

Tel: +44 (0) 207 843 4924
email: zhe.wang@nature.com

Reviewer #1 (Remarks to the Author):

The authors have very thoroughly addressed my comments on the original submission.

Reviewer #2 (Remarks to the Author):

I have reviewed the revised manuscript and the authors’ point-by-point response. In my view, the authors have addressed the reviewer concerns satisfactorily. The revision includes substantial new experimental and analytical work, including additional tissue validation, spatial analyses, improved quantification, mechanistic interrogation of epithelial–stromal signaling, sequential co-culture experiments, and a more balanced discussion of model limitations.

I do not see any major outstanding concerns. The remaining caveats are appropriately acknowledged and do not materially weaken the central conclusions. I would support acceptance of the revised manuscript.

Reviewer #3 (Remarks to the Author):

In the revised manuscript and the rebuttal, the authors provide a lot of additional data and analyses to address my and the other reviewers ´ issues. This includes a wealth of new data and methodology (e.g. spatial transcriptomics) as well as more mechanistic data. The authors should be gratulated for their important work and provision of the revised and new data. I agree with the authors that the figures R3.9 and 3.11 may not be included in the manuscript. Overall, I support acceptance of this work for publication in NCB.

INTERNAL USE ONLY AIP modules required: ["Life Science"]

Version 2:

Decision Letter:

Dear Dr Biffi,

I am pleased to inform you that your manuscript, "Multi-stromal organoid co-culture modelling reveals epithelial-fibroblast heterogeneity in pancreatic cancer and pancreatitis", has now been accepted for publication in Nature Cell Biology.

Thank you for sending us the final manuscript files to be processed for print and online production, and for returning the manuscript checklists and other forms. Your manuscript will now be passed to our production team who will be in contact with you if there are any questions with the production quality of supplied figures and text.

Over the next few weeks, your paper will be copyedited to ensure that it conforms to Nature Cell Biology style. Once your paper is typeset, you will receive an email with a link to choose the appropriate publishing options for your paper and our Author Services team will be in touch regarding any additional information that may be required.

After the grant of rights is completed, you will receive a link to your electronic proof via email with a request to make any corrections within 48 hours. If, when you receive your proof, you cannot meet this deadline, please inform us at rjsproduction@springernature.com immediately.

You will not receive your proofs until the publishing agreement has been received through our system.

Due to the importance of these deadlines, we ask that you please let us know now whether you will be difficult to contact over the next month. If this is the case, we ask you provide us with the contact information (email, phone and fax) of someone who will be able to check the proofs on your behalf, and who will be available to address any last-minute problems.

If you have any questions about our publishing options, costs, Open Access requirements, or our legal forms, please contact ASJournals@springernature.com

Once your paper has been scheduled for online publication, the Nature press office will be in touch to confirm the details. An online order form for reprints of your paper is available at https://www.nature.com/reprints/author-reprints.html. All co-authors, authors' institutions and authors' funding agencies can order reprints using the form appropriate to their geographical region.

Publication is conditional on the manuscript not being published elsewhere and on there being no announcement of this work to any media outlet until the online publication date in Nature Cell Biology.

You may wish to make your media relations office aware of your accepted publication, in case they consider it appropriate to organize some internal or external publicity. Once your paper has been scheduled you will receive an email confirming the publication details. This is normally 3-4 working days in advance of publication. If you need additional notice of the date and time of publication, please let the production team know when you receive the proof of your article to ensure there is sufficient time to coordinate. Further information on our embargo policies can be found here: https://www.nature.com/authors/policies/embargo.html

Please note that *Nature Cell Biology* is a Transformative Journal (TJ). Authors may publish their research with us through the traditional subscription access route or make their paper immediately open access through payment of an article-processing charge (APC). Authors will not be required to make a final decision about access to their article until it has been accepted. Find out more about Transformative Journals

Authors may need to take specific actions to achieve compliance with funder and institutional open access mandates. If your research is supported by a funder that requires immediate open access (e.g. according to Plan S principles or the NIH public access policy) then you should select the gold OA route, and we will direct you to the compliant route where possible. Because authors warrant under our subscription licensing terms that they haven't committed to licensing any version of their article under a licence inconsistent with the terms of our agreement – including the applicable embargo period – publication under the subscription model isn't suitable for authors whose funders require no embargo.

To assist our authors in disseminating their research to the broader community, our SharedIt initiative provides you with a unique shareable link that will allow anyone (with or without a subscription) to read the published article. Recipients of the link with a subscription will also be able to download and print the PDF.

If your paper includes color figures, please be aware that in order to help cover some of the additional cost of four-color reproduction, Nature Portfolio charges our authors a fee for the printing of their color figures. Please contact our offices for exact pricing and details.

As soon as your article is published, you will receive an automated email with your shareable link.

If you have not already done so, we strongly recommend that you upload the step-by-step protocols used in this manuscript to protocols.io (https://protocols.io), an open online resource that allows researchers to share their detailed experimental know-how. All uploaded protocols are made freely available and are assigned DOIs for ease of citation. Protocols and Nature Portfolio journal papers in which they are used can be linked to one another, and this link is clearly and prominently visible in the online versions of both. Authors who performed the specific experiments can act as primary authors for the Protocol as they will be best placed to share the methodology details, but the Corresponding Author of the present research paper should be included as one of the authors. By uploading your Protocols onto protocols.io, you are enabling researchers to more readily reproduce or adapt the methodology you use, as well as

increasing the visibility of your protocols and papers. You can also establish a dedicated workspace to collect your lab Protocols. Further information can be found at https://www.protocols.io/help/publish-articles.

You can use a single sign-on for all your accounts, view the status of all your manuscript submissions and reviews, access usage statistics for your published articles and download a record of your refereeing activity for the Nature Portfolio.

Nature Cell Biology encourages authors presenting evidence for cell, biological, molecular, and genetic interactions to consider communicating these findings using Biofactoid (https://biofactoid.org/). This tool helps users share a searchable representation of interactions (e.g. binding, gene expression, post-translational modification) between genes, gene products, or chemicals. Information added to Biofactoid, with author attribution, is shared on social media and public databases, such as Pathway Commons, where it can be discovered and analyzed in the context of a large and growing corpus of knowledge.

Please feel free to contact us if you have any questions.

With kind regards,

Zhe Wang, PhD
Senior Editor
Nature Cell Biology

Tel: +44 (0) 207 843 4924
email: zhe.wang@nature.com

Click here if you would like to recommend Nature Cell Biology to your librarian
http://www.nature.com/subscriptions/recommend.html#forms

** Visit the Springer Nature Editorial and Publishing website at www.springernature.com/editorial-and-publishing-jobs for more information about our career opportunities. If you have any questions please click here.**

Open Access This Peer Review File is licensed under a Creative Commons Attribution 4.0 International License, which permits use, sharing, adaptation, distribution and reproduction in any medium or format, as long as you give appropriate credit to the original author(s) and the source, provide a link to the Creative Commons license, and indicate if changes were made.
In cases where reviewers are anonymous, credit should be given to 'Anonymous Referee' and the source.
The images or other third party material in this Peer Review File are included in the article's Creative Commons license, unless indicated otherwise in a credit line to the material. If material is not included in the article's Creative Commons license and your intended use is not permitted by statutory regulation or exceeds the permitted use, you will need to obtain permission directly from the copyright holder.
To view a copy of this license, visit https://creativecommons.org/licenses/by/4.0/

Point-by-point response to reviewers' comments

Reviewer #1 (Remarks to the Author):

In this manuscript by Li, Jihad and colleagues, the authors interrogate and compare epithelial-mesenchymal crosstalk in the contexts of pancreatitis and pancreatic cancer. The authors have performed rigorous, extensive comparisons of human and mouse disease states, together with development and analysis of a new co-culture model which captures heterocellular crosstalk in culture. This is a very timely study, as we have come a long way as a field in our appreciation of CAF heterogeneity in PDAC but have yet to meaningfully extend these findings to pancreatitis. Further, pancreatitis is a well-appreciated risk factor for PDAC, but careful cellular and molecular comparisons between these conditions have not been performed comprehensively, highlighting the novelty of this work. The molecular analyses here are poised to be hypothesis-generating and of great interest to the field in motivating functional studies of paracrine signaling in the development of pancreatitis and PDAC. However, the study is also poised to be highly impactful on its own by describing the first "multi-stroma" co-culture system capturing distinct mesenchymal cells of origin for activated fibroblasts, the first pancreatitis-derived organoid model that faithfully recapitulates the heterogeneity and complexity of the disease in intact tissues, and the first detailed comparison of the stromal reaction in human and mouse pancreatitis versus PDAC. The study also represents a meaningful conceptual advance in linking mesenchymal lineage heterogeneity to physiologically relevant heterogeneity in the epithelial compartment. A limited number of additional analyses would help to support the authors' central claims, particularly in establishing core differences between disease states across species.

We thank the Reviewer for their supportive and insightful assessment of our work. We appreciate their comments, which have helped us clarify and strengthen our central conclusions and expand the scope of the study.

Critique R1.1: *The direct comparison of activated fibroblast markers in mouse models of pancreatitis and PDAC in Figures 1 and ED Figure 1 are central to the authors' claims and would benefit from a bit of clarification. Specifically, the PDPN staining in ED Figure 1N could likely be improved to reduce background—this seems worthwhile as PDPN is such a commonly used marker for activated fibroblasts or CAFs in studies of mouse pancreas. In Figure 1K, the trichrome staining is certainly convincing, but the comparison of collagen abundance between pancreatitis and PDAC may be helped by the added granularity of separately quantifying poorly and moderately or well differentiated tumor regions. As malignant ductal or glandular structures generally have more myofibroblastic density and ECM in their vicinity than poorly differentiated tumor regions, the comparison to metaplastic ductal regions in pancreatitis may show comparable collagen abundance to well/moderately differentiated PDAC but less in poorly differentiated areas. This seems a meaningful addition in line with the authors' key claims which could presumably be done by parsing histologically distinct regions of KPC tissues the authors have in hand.*

Response R1.1: We thank the Reviewer for these constructive and insightful suggestions, which enabled us to validate, strengthen and expand our original conclusions.

1) To further improve PDPN immunohistochemistry (IHC) and reduce background staining, we changed our approach (from manual IHC) and worked with our Institute's core facility to optimise a protocol on Leica's automated Bond-III platform. This involved increasing staining stringency and antibody dilution. Using this optimised protocol, we replaced all original PDPN IHC images and now

include representative examples in **revised Extended Data (ED) Figures 2E** and **2K** (e.g., **Figure R1.1A**).

This improved staining enabled us to reduce the stringency of the quantification algorithm. Importantly, the updated analyses in **revised ED Figures 2F** and **2L** (e.g., **Figure R1.1B**): 1) validate our original conclusions by confirming increased PDPN abundance in pancreatitis and PDAC compared with normal pancreas, as well as higher PDPN abundance in pancreatitis relative to PDAC; and 2) allow more comprehensive detection of PDPN-positive cells, revealing PDPN abundance comparable to that of PDGFRα (**Fig. 2E** of the revised manuscript). The corresponding Methods section has been updated.

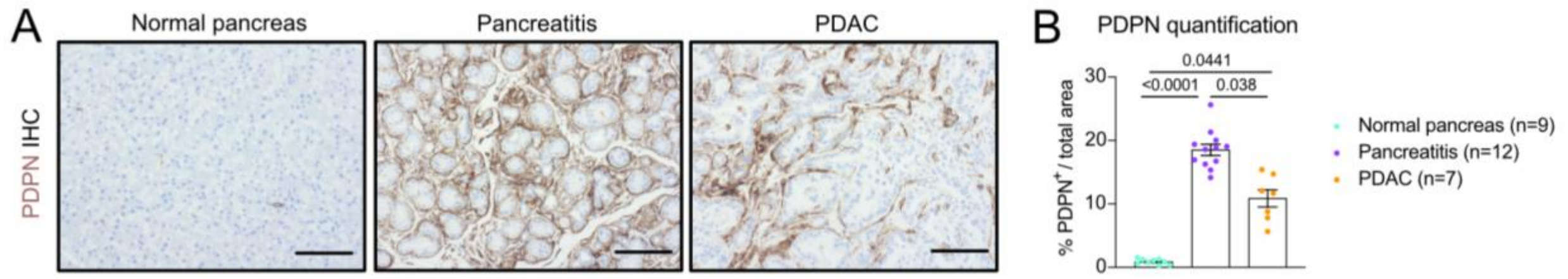

Figure R1.1. A-B. Representative images **(A)** and quantifications **(B)** of PDPN stains of normal pancreas (n=9), pancreatitis (n=12) and PDAC (n=7) murine tissues. Results show mean ± SEM. *P adj*, Kruskal-Wallis test. Scale bars, 50 μm. Pancreatitis samples derive from two independent treatment cohorts.

2) To address the Reviewer's suggestion to stratify collagen deposition by tumour differentiation state, a pathologist (R.B.) first annotated all PDAC tumours analysed in the original manuscript. We found that most tumours in the initial dataset were uniformly moderately differentiated, and these collagen quantifications are shown in **Figure R1.2A** (for review purposes).

To better capture a broader range of PDAC differentiation states, we subsequently analysed nine additional PDAC tumours following pathologist annotation (**Figures R1.2B-C**). These new data are included in **new ED Figures 2M-N** of the revised manuscript, and detailed information on all 17 tumours analysed is provided in **new Supplementary Table 4**. Importantly, these expanded analyses: 1) confirm our original conclusion that collagen abundance is higher in pancreatitis also across well-differentiated PDAC regions; and 2) do not reveal significant differences in collagen deposition among undifferentiated, moderately differentiated or well differentiated PDAC areas of our mouse models.

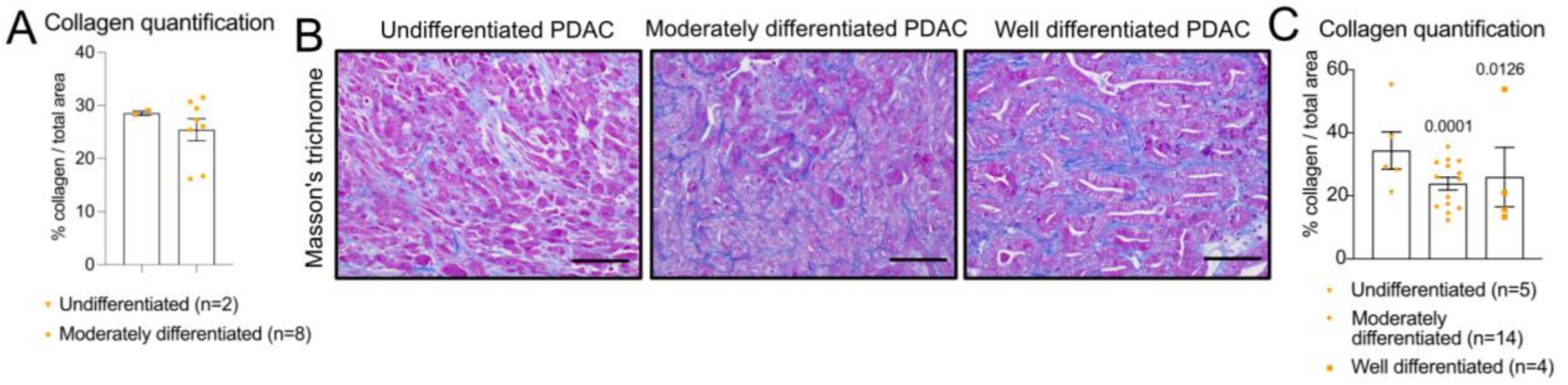

Figure R1.2. A. quantifications of Masson's trichrome stains of undifferentiated (n=2) and moderately differentiated (n=8) areas from the murine PDAC tissues analysed in the original manuscript (n=8). **B.** Representative images and quantifications of Masson's trichrome stains of undifferentiated (n=5), moderately differentiated (n=14) and well differentiated (n=4) areas from murine PDAC tissues (n=17). *P adj*, Kruskal-Wallis test compared to murine pancreatitis tissues from Figure 2. Scale bars, 50 μm.

Critique R1.2: *The snRNA-seq from mouse PDAC as shown in Figure 2A-2D appears heavily dominated by the malignant (epithelial) compartment, suggesting substantial dropout of stromal populations during sample preparation. To ensure differences between disease states are not due to bias in cell type retention during nuclear isolation, it would be helpful here to select a small number of key differentially expressed genes (or inferred differentially activated pathways) and confirm the snRNA-seq data by immunohistochemical staining on tissues, or by fibroblast isolation and qPCR.*

Response R1.2: We agree with the Reviewer that independent validation of gene expression differences identified by snRNA-seq is important. In the original manuscript, we had already included a number of orthogonal validation approaches. Specifically, in Figures 4, 6, and 7, we presented multiplex immunofluorescence (IF) analyses of murine pancreatitis and PDAC tissues for key differentially expressed genes (DEGs), including epithelial markers (*Sox9*, *Stat1*, *Muc5ac*) and fibroblast markers (*Pdgfra*, *Acta2*, *Fn1*), as well as western blot validation in organoids (SOX9, p19). Nonetheless, we appreciate the Reviewer's suggestion that this validation could be further expanded.

Due to poor cell viability following pancreatitis tissue digestion, we were unable to perform transcriptomic analyses of freshly isolated pancreatitis fibroblasts. Thus, to further mitigate concerns regarding cell-type retention bias during nuclei isolation, we have substantially expanded both our protein-level and transcriptomic validation in the revised manuscript, as detailed below.

1) **Expanded *in vitro* validation of epithelial cell DEGs.** In **revised Figure 5H**, we included new western blot analyses in pancreatitis (P) and PDAC organoids validating additional PDAC epithelial-upregulated DEGs identified by snRNA-seq, namely *Dpp4* and *Sox4* (**Figure R1.3A**).
2) **Expanded *in vivo* validation of epithelial cell DEGs.** In **new Figures 7K-L**, we included multiplex IF validation of CD44, an additional epithelial marker identified as differentially expressed in PDAC malignant cells by snRNA-seq (**Figures R1.3B-C**). Moreover, for all previously validated epithelial markers (SOX9, STAT1, MUC5AC), as well as CD44, we extended multiplex IF analyses to two additional murine PDAC models ($KRAS^{G12D}$ p53-null and SMAD4-deficient $KRAS^{G12D}$ p53-mutant), in response to Reviewer #3 (**new ED Figures 8J-P**; e.g., **Figure R3.5**), further strengthening the robustness of our findings.
3) **Expanded validation of fibroblast DEGs.** In **new Figures 8J-K** and **new ED Figures 9O-T**, we present multiplex IF analyses validating additional fibroblast DEGs identified by snRNA-seq, including myofibroblastic markers (*Tnc*, *Ncam1* encoding CD56, and *Runx1*) and a pancreatitis-associated fibroblast marker (*Cd34*) (**Figures R1.3D-K**). As with epithelial markers, all previously validated fibroblast markers (*Pdgfra*, *Acta2*, *Fn1*) and newly added markers were also assessed across the two additional murine PDAC models (**new ED Figures 9L-N**; **Figure R3.3**), reinforcing the generalisability of these observations.
4) **Spatial transcriptomic validation.** To directly address concerns regarding sampling bias and cell-type representation, we performed Visium HD spatial transcriptomics on murine PDAC and pancreatitis tissues. These analyses confirmed a high abundance of malignant cells within the PDAC core regions used for snRNA-seq, as well as a higher fibroblast-to-ductal cell ratio in pancreatitis (**Figures R1.3L-M**). We additionally analysed normal murine pancreas tissues (**new Figure 2J**; **Figure R2.4**), in response to Reviewer #2, with all spatial data included in **new Figures 2J-L**, **new ED Figures 2O-R** and **new Supplementary Table 5**.
5) ***In situ* validation of pancreatitis fibroblast and ductal subsets.** We further performed multiplex RNA *in situ* hybridization (ISH) combined with immunofluorescence to validate fibroblast subset identity and marker co-expression in murine pancreatitis tissues. This confirmed multiple fibroblast

populations identified by snRNA-seq, including *Il34*^High^WT1^High^ Fibro 1, *Pi16*^High^PDGFRα^High^ Fibro 2, *Vegfc*^High^*Fap*^High^ and/or *Cxcl12*^High^*Fap*^High^ Fibro 3 and *Muc16*^High^WT1^High^ Fibro 4 (e.g., **Figure R1.3N**), as well as SPP1^Low^ Ductal 1 and SPP1^High^ Ductal 2 populations (**Figure R1.4C**). These data are included in **new Figures 3C-D**, **new ED Figure 3K** and **new ED Figure 4L**.

Taken together, these expanded protein-level, spatial, and transcriptomic validation approaches directly address concerns regarding the original snRNA-seq strategy. They substantially strengthen our conclusion that cross-species and cross-model transcriptomic analyses can be robustly leveraged to identify disease-specific markers for validation in pancreatitis and PDAC tissues.

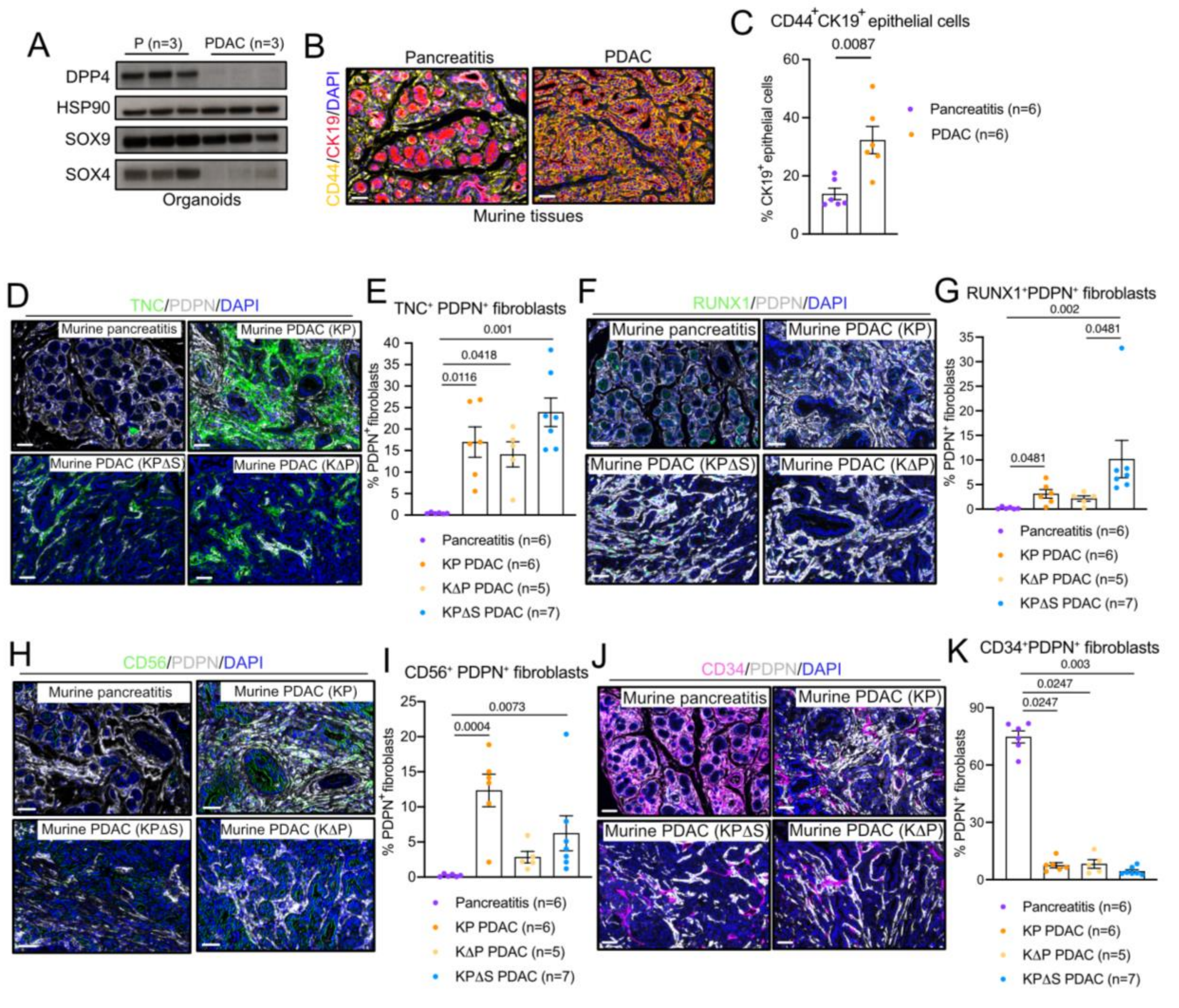

Figure R1.3 [Part 1]. A. Western blot analysis of DPP4, SOX9 and SOX4 in P (n=3) and PDAC (n=3) organoids cultured in RM for 3 days. HSP90, loading control. **B.** Representative multiplex immunofluorescence (IF) images of CD44 (yellow), CK19 (red) and DAPI (blue) in murine pancreatitis and PDAC tissues. Scale bars = 50 µm. **C.** Quantification of cytoplasmic-CD44^+^CK19^+^DAPI^+^αSMA^-^PDGFRα^-^ cells relative to DAPI^+^CK19^+^αSMA^-^PDGFRα^-^ epithelial cells in murine pancreatitis (n=6) and PDAC (n=6) tissues. Results show mean ± SEM. *P*, Mann-Whitney test. **D-K.** Representative multiplex IF images of PDPN (white) and DAPI (blue), as well as tenascin (TNC, green, **D**) or RUNX1 (green, **F**) or CD56 (green, **H**) or CD34 (pink, **J**) in murine pancreatitis, PDAC tissues from KPC GEMMs (i.e., KP PDAC), as well as PDAC tissues derived from orthotopic transplantation models of KRAS^G12D^, p53-null organoids (i.e., KΔP PDAC) or *Smad4*-deleted, KRAS^G12D^, p53^R172H^ organoids (i.e., KPΔS PDAC). Scale bars = 50 µm. Quantification of TNC^+^PDPN^+^DAPI^+^CK19^-^ cells **(E)** or nuclear-RUNX1^+^PDPN^+^DAPI^+^CK19^-^ cells **(G)** or CD56^+^PDPN^+^DAPI^+^CK19^-^ cells **(I)** or CD34^+^PDPN^+^DAPI^+^CK19^-^ cells **(K)** relative to PDPN^+^DAPI^+^CK19^-^ fibroblasts in murine pancreatitis (n=6), KP PDAC (n=6), KΔP PDAC (n=5) and KPΔS PDAC (n=7) tissues. Results show mean ± SEM. *P adj*, Kruskal-Wallis test.

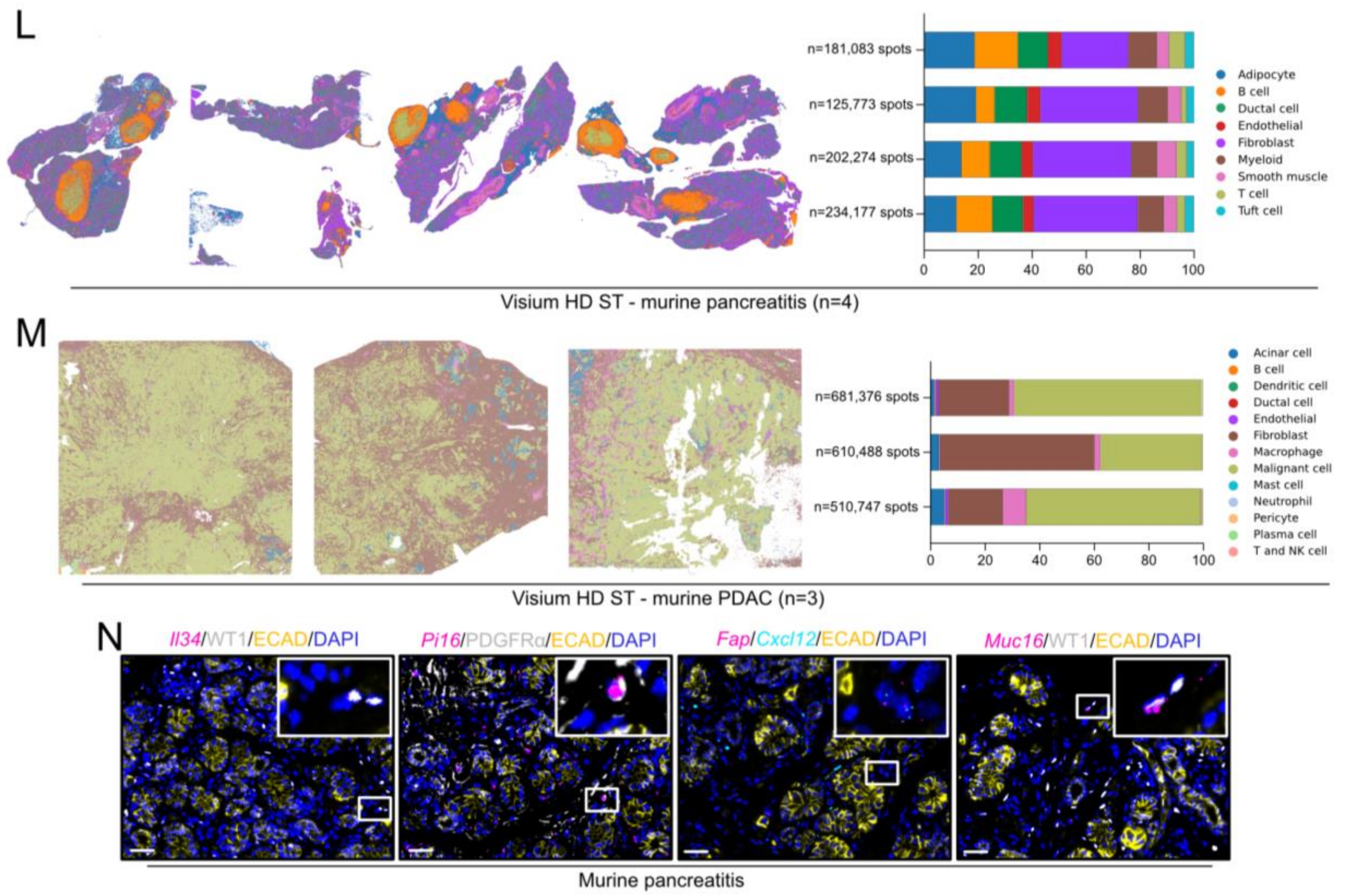

Figure R1.3 [Part 2]. L. Visium HD spatial transcriptomics (ST) slide images (left) and corresponding deconvoluted cell type contribution (right) represented as bar plots showing the proportions of the different cell clusters in each murine pancreatitis tissue (n=4). **M.** Visium HD ST slide images (left) and corresponding deconvoluted cell type contribution (right) represented as bar plots showing the proportions of the different cell clusters in each murine PDAC tissue (n=3). **N.** Representative multiplex IF and RNA *in situ* hybridization images of *Il34* (pink) and WT1 (white) to identify the Fibro 1 subset (far left), *Pi16* (pink) and PDGFRα (white) to identify the Fibro 2 subset (left), *Cxcl12* (cyan) and *Fap* (pink) to identify the Fibro 3 subset (right) or *Muc16* (pink) and WT1 (white) to identify the Fibro 4 subset (far right) in murine pancreatitis tissues. DAPI (blue), ECAD (yellow). Scale bars = 20 μm.

Critique R1.3: *Through their analyses in Figure 2, the authors nicely display Fibro 3 and Ductal 1 as the transcriptional nearest neighbors to PDAC CAFs and malignant epithelial cells, respectively, from the pancreatitis setting. Do these populations (Fibro 3 and Ductal 1) spatially localize near each other in pancreatitis tissues? And does ligand-receptor analysis suggest that these two clusters communicate preferentially with each other? While identification of each population is a nice addition, some implication of crosstalk between these defined epithelial and stromal cells might implicate a precancerous or cancer-like niche in the setting of inflammation.*

Response R1.3: We thank the reviewer for this insightful suggestion, which prompted us to further explore spatial organisation and intercellular communication between pancreatitis epithelial and stromal populations that are transcriptionally closest to their PDAC counterparts.

1) We performed ligand-receptor interaction analysis using CellChat on the murine pancreatitis snRNA-seq dataset. This analysis revealed a stronger degree of inferred communication between *Fap*^{High} Fibro 3 and Ductal 1 cells compared to Ductal 2 cells (**Figure R1.4A**; **new ED Figure 4J**). Given that Fibro 3 is transcriptionally closest to PDAC CAFs and Ductal 1 cells are closest to PDAC malignant cells, this interaction pattern may reflect an early shift towards a malignant-like communication programme in the context of prolonged inflammation.
2) To assess whether these populations form a defined spatial niche within pancreatitis tissues, we first performed Visium HD spatial transcriptomic analyses of murine pancreatitis samples (n=4) now included in **new Figure 2K** of the revised manuscript. Spatial mapping demonstrated that Ductal 1 and Fibro 3 cells are broadly interspersed with other epithelial and stromal populations rather than co-localising within restricted regions (**Figure R1.4B**; **new ED Figure 4K**).
3) To definitively validate these spatial relationships at single-cell resolution, we performed multiplex RNA ISH combined with immunofluorescence. Leveraging our snRNA-seq data, we identified *Spp1* as being more highly expressed in Ductal 2 than in Ductal 1 cells (**revised ED Figure 3I**), enabling spatial discrimination between these ductal populations. Multiplex RNA ISH for *Fap* combined with IF for E-cadherin (ECAD) and SPP1 in murine pancreatitis tissues confirmed that *Fap*^{High} Fibro 3 cells do not form an isolated or preferential spatial niche with Ductal 1 cells (**Figure R1.4C**). Instead, the tissue exhibits marked spatial heterogeneity, with SPP1^{Low} Ductal 1 and SPP1^{High} Ductal 2 cells often located adjacent to one another within the same acinar-to-ductal metaplasia structures. These data are included in **new ED Figure 4L**.

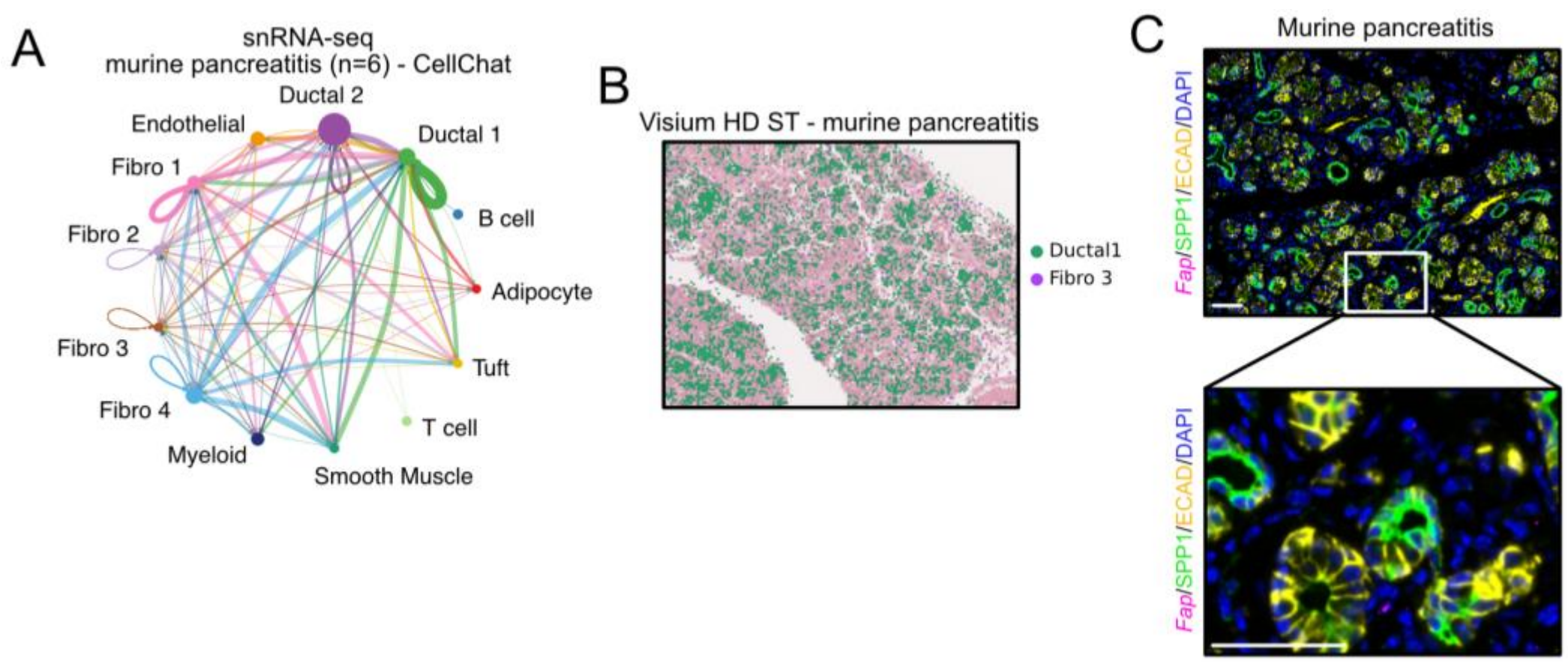

Figure R1.4. A. Cell-cell communication analysis using CellChat showing the connections between all cell types identified in murine pancreatitis, as assessed by snRNA-seq. The thickness of the connection reflects the number of interactions. **B.** Representative Visium HD spatial transcriptomics (ST) slide image showing Ductal 1 and Fibro 3 populations in murine pancreatitis. **C.** Representative multiplex IF and RNA *in situ* hybridization image of DAPI (blue), *Fap* (pink), SPP1 (green) and ECAD (yellow) to identify *Fap*^{High} Fibro 3, SPP1^{Low}ECAD^{+} Ductal 1 and SPP1^{High}ECAD^{+} Ductal 2 populations in murine pancreatitis tissues. Bottom panel: insert. Scale bars = 50 μm. SPP1 was chosen to distinguish between Ductal 1 and Ductal 2 cells based on the snRNA-seq data of murine pancreatitis tissues (Extended Data Figure 3I).

Critique R1.4: *Comparing Figure 2N to Figure 3E, 3F, and ED Figure 3F, it seems a number of fibroinflammatory pathways upregulated in PDAC epithelial cells compared to pancreatitis are reversed in the organoids (i.e., higher in pancreatitis-derived organoids). Do these signatures align specifically with the Ductal 1 signature from intact pancreatitis tissues, perhaps suggesting a preferential outgrowth of these malignant-like epithelial cells during P organoid preparation? These results do not detract from the authors' conclusions, but some further explanation of differences between epithelial states in vivo and in vitro would help frame the conclusions.*

Response R1.4: We thank the Reviewer for this thoughtful comment, which allowed us to clarify and expand our original conclusions regarding similarities between epithelial states *in vivo* and *in vitro*. In the original manuscript, we compared transcriptional programmes between Ductal 1 and Ductal 2 epithelial populations and found that inflammatory pathways upregulated in PDAC malignant cells relative to pancreatitis Ductal 1 cells, and they were not significantly different between Ductal 1 and Ductal 2 subsets (e.g., HALLMARK inflammatory response and HALLMARK TNFA signaling via NFKB; **Figure 3P** and **ED Figure 4F**). We also showed that pancreatitis (P) organoids are enriched for both Ductal 1 and Ductal 2 signatures (**ED Figure 5I**). Importantly, although inflammatory pathways enriched in PDAC epithelial cells *in vivo* were reduced in PDAC organoid monocultures compared with P organoid monocultures, as noted by the reviewer, these same pathways were enriched in PDAC organoids relative to P organoids in multi-stroma co-cultures (e.g., HALLMARK TNFA signaling via NFκB, **Figure 7F**). To make these relationships clearer, we have revised the text and added new analyses, as detailed below.

1) To better highlight the closer concordance between epithelial states *in vivo* and in multi-stroma co-cultures, and to clarify that the induction of these inflammatory signatures is stroma-dependent, we expanded the text and added the following sentence in the revised manuscript: '*As PDAC organoid monocultures did not upregulate inflammatory pathways relative to P organoid monocultures, these findings suggest that the in vivo upregulation of specific inflammatory pathways (e.g., NF-κB signalling) in PDAC malignant cells requires stromal input.'* (lines 657-660).
2) To complement the gene set enrichment analyses in ED Figure 5I, which showed enrichment of both ductal 1 and ductal 2 signatures in P organoids, we assessed the overlap between differentially expressed genes in P organoids and *in vivo* murine pancreatitis Ductal 1 and Ductal 2 marker sets. These analyses confirm our original conclusion that P organoids significantly recapitulate features of both pancreatitis Ductal 1 and Ductal 2 cells (**Figure R1.5A**). These data are included in **new ED Figure 5K** and **revised Supplementary Table 8**.
3) Finally, we performed Gene Set Variation Analysis (GSVA) to evaluate potential heterogeneity across P organoid lines. Although we observed line-to-line variability in enrichment scores for Ductal 1 and Ductal 2 signatures, these results did not reveal a consistent preferential outgrowth of Ductal 1 over Ductal 2 states (**Figure R1.5B**). These data are included in **new ED Figure 5J**.

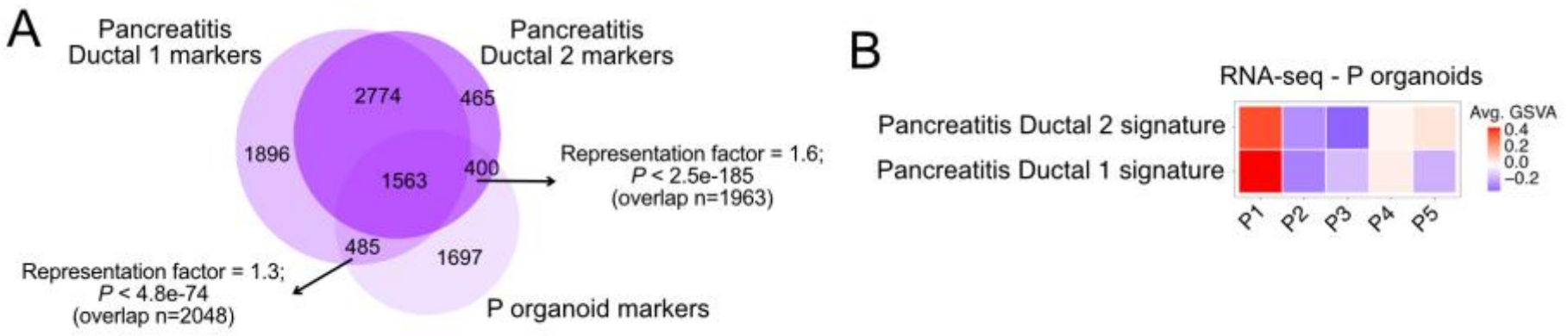

Figure R1.5. A. Venn diagrams of curated protein-coding genes significantly (*P* adj < 0.05) upregulated in P organoids cultured in complete media compared to PDAC organoids in cultured in complete media (P organoid markers) and murine pancreatitis Ductal 1 or Ductal 2 cells compared to PDAC malignant cells *in vivo* (pancreatitis Ductal 1 or 2 markers), as assessed by RNA-seq and snRNA-seq, respectively. Significance of the overlap between datasets was defined by hypergeometric test (with denominator n=18,000). **B.** Heatmap showing Gene Set Variation Analysis (GSVA) enrichment scores (averaged per P organoid line) of murine pancreatitis Ductal 1 and Ductal 2 signatures, as assessed by RNA-seq.

Critique R1.5: *In depicting the multi-stroma co-cultures in Figure 6, it would be meaningful to indicate the relative abundance of these cell populations using their fluorescent and cell surface markers over the course of co-culture (analogous to analyses depicted in Figure 4C & 4D). It seems reasonably likely that pancreatitis- and PDAC-derived organoids differentially select for expansion of defined mesenchymal populations, which would be helpful in interpreting these results and identifying axes for future study.*

Response R1.5: We thank the Reviewer for this suggestion. To address this point, we performed additional analyses and expanded the discussion to better interpret cell population dynamics within the multi-stroma co-culture system.

1) We first quantified the stromal-to-epithelial cell ratio in P organoid/multi-stroma and PDAC organoid/multi-stroma co-cultures. Consistent with our *in vivo* analyses and findings from pancreatic stellate cell (PSC) co-cultures, we observed a higher stroma/epithelial ratio in P organoid/multi-stroma co-cultures relative to PDAC organoid/multi-stroma co-cultures (**Figure R1.6A**). These data are now included in **new Figure 7D**.
2) We next assessed the abundance of GFP$^+$PDPN$^+$ cells (PSCs) and TdTomato$^+$PDPN$^+$ cells (mixed-fibroblasts - corresponding to the fibroblasts described in the original manuscript, as clarified in response to Reviewer #2 - and mesothelial cells) across three conditions: (1) multi-stroma cultures without organoids (n=4), (2) P organoid/multi-stroma co-cultures (n=7) and (3) PDAC organoid/multi-stroma co-cultures (n=7). These analyses revealed that P organoids more strongly promote expansion of PSCs relative to PDAC organoids, consistent with our earlier observations in PSC-only co-cultures (**Figures R1.6B-C**). In contrast, no significant difference was observed in the abundance of mixed-fibroblasts/mesothelial cells between conditions. These data are included in **new Figure 7C** and **new ED Figure 8D**).
3) Following the Reviewer's suggestion, we attempted to further resolve mesothelial cells and mixed-fibroblasts using multiple cell-surface markers differentially expressed between these populations. However, despite several efforts, we were unable to identify a marker that consistently captured the majority of each population with sufficient specificity to allow confident separation. Examples of the markers analysed are shown in **Figure R1.7** (for review only). We have therefore added a discussion point highlighting the future need for stromal cell lines expressing distinct fluorescent reporters to enable their separation in multi-stroma co-cultures (lines 940-942).

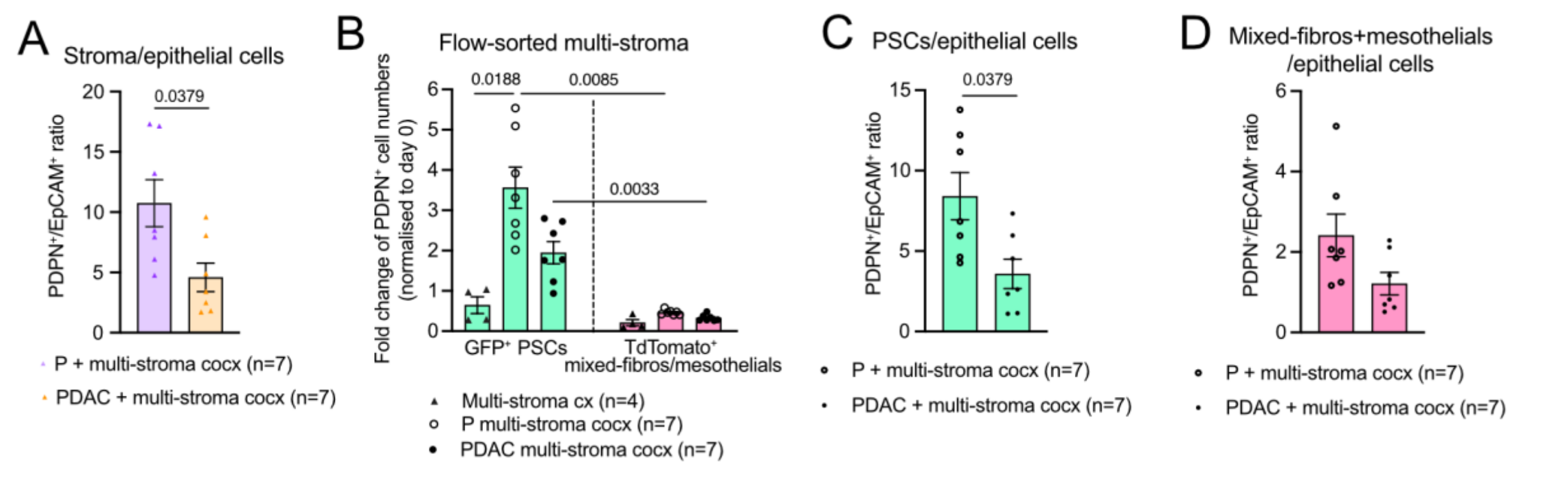

Figure R1.6. A. Stroma/epithelial cell ratio as assessed by flow sorting of P or PDAC multi-stroma cocx. Results show mean ± SEM. *P*, Mann-Whitney test. **B.** Fold change numbers of GFP$^+$PDPN$^+$EpCAM$^-$ PSCs and tdTomato$^+$PDPN$^+$EpCAM$^-$ mixed-fibroblasts and mesothelial cells flow-sorted from multi-stroma cx or from P or PDAC multi-stroma cocx relative to numbers of respective GFP$^+$ or tdTomato$^+$ cells plated. Results show mean ± SEM. *P adj*, Kruskal-Wallis test. **C-D.** PSC (GFP$^+$PDPN$^+$EpCAM$^-$)/epithelial cell ratio **(C)** or Mixed-fibroblasts and mesothelial cells (TdTomato$^+$PDPN$^+$EpCAM$^-$)/epithelial cell ratio **(D)** as assessed by flow sorting of P or PDAC multi-stroma cocx. Results show mean ± SEM. *P*, Mann-Whitney test.

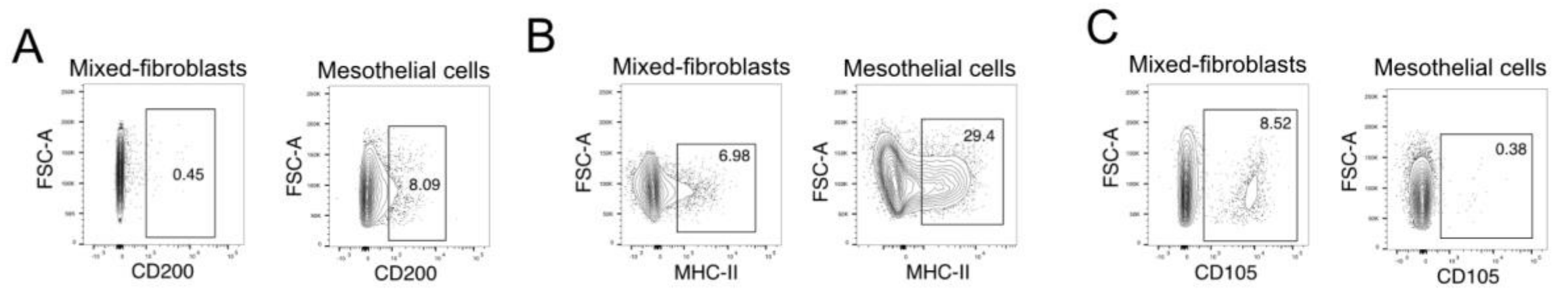

Figure R1.7. A-C. Representative contour flow plots of CD200 **(A)**, MHC-II **(B)** and CD105 **(C)** in mixed-fibroblasts and mesothelial cells cultured in 2D. Cells for panel B were treated with IFN-γ.

4) Finally, given the marked differences in stromal cell expansion revealed by these new analyses, we expanded the Discussion to note that future iterations of this multi-stroma model may require adjustment of initial stromal cell ratios to favour mixed-fibroblasts and mesothelial cells to better recapitulate the stromal composition observed *in vivo* (lines 938-940).

We again thank the Reviewer for their thoughtful and constructive feedback. We believe that the additional analyses and revisions to the text have further improved the manuscript and enhanced its contribution to the field.

Reviewer #2 (Remarks to the Author):

The authors investigate how pancreatic cancer versus chronic pancreatic inflammation differentially shape epithelial-stromal interactions. This study integrates analyses of human and murine pancreatitis and pancreatic ductal adenocarcinoma (PDAC) samples with novel multi-stromal organoid co-cultures that include pancreatic stellate cells (PSCs), fibroblasts, and mesothelial cells. Using these models, the authors demonstrate that malignant and inflammatory epithelial cells drive distinct fibroblast heterogeneity and crosstalk. For example, chronic pancreatitis fibroblasts are shown to express higher levels of fibrotic/inflammatory markers such as PDGFRα and podoplanin, whereas PDAC cancer-associated fibroblasts (CAFs) exhibit a more myofibroblastic phenotype with elevated αSMA expression. The multi-stromal organoid co-cultures successfully recapitulate many in vivo gene expression features of pancreatitis and PDAC stromal cells. Cross-species single-cell RNA sequencing comparisons indicate that a combination of these in vitro and in vivo systems better captures human disease signatures than traditional models, and they reveal condition-specific stromal reprogramming signals (e.g. differential PDGF, TGF-β, NF-κB pathway activation). Notably, the authors infer that different stromal cell origins (PSC-derived vs. mesothelial-derived fibroblasts) preferentially contribute to distinct fibroblast subpopulations in PDAC and pancreatitis. Overall, this comprehensive study provides useful insights into how malignant vs. inflammatory epithelial cells reprogram the surrounding stromal compartment, and it offers a complementary platform to identify disease-specific fibroblast markers and epithelial-stromal dependencies.

We thank the Reviewer for their thoughtful and supportive assessment of our work. We appreciate their recognition of the conceptual and technical advances of the study, and their comments have helped us further clarify and strengthen our conclusions as well as expand the scope of the analyses and discussion. We believe that these revisions have enhanced the clarity, depth, and overall impact of the manuscript.

Critique R2.1: *The comparative approach between PDAC and pancreatitis models is novel, and it yields important insights into epithelial-stromal crosstalk. In particular, the finding that pancreatic cancer organoids induce a more myofibroblastic, collagen-producing fibroblast state (with reduced interferon-signaling) compared to pancreatitis organoids is compelling. This indicates that cancer cells send distinct cues to stromal cells. However, the mechanistic basis of these differences remains largely correlative. The manuscript would be elevated to the level of Nature Cell Biology by deeper exploration of specific signaling interactions. For instance, can the authors identify which tumor-derived factors (e.g. TGF-β, Hedgehog, PDGF ligands) drive the myofibroblastic shift, or conversely which inflammatory signals dominate in pancreatitis co-cultures? Demonstrating causation – for example, by inhibiting a candidate pathway in co-culture and showing a shift in fibroblast phenotype – would provide more concrete mechanistic insight beyond transcriptomic correlations. At a minimum, a more detailed discussion of how epithelial cells reprogram fibroblasts (perhaps referencing known pathways like TGF-β or IL-1 signaling in CAF activation) would help contextualize the findings. Overall, the study's data only hint at important mechanisms of tumor–stroma communication but clarifying these mechanisms would elevate the impact and justify publication in Nature Cell Biology.*

Response R2.1: We thank the Reviewer for this thoughtful and constructive critique, which prompted us to substantially deepen the mechanistic analysis of epithelial–stromal crosstalk in pancreatitis and PDAC. In response, we performed multiple new experiments and integrative

analyses that move beyond correlative transcriptomic observations and establish causal signalling mechanisms.

A) Mechanistic basis of the PDAC-dependent inflammatory PSC phenotype.

In the original manuscript, we observed higher *Il1a* expression in PDAC organoids relative to pancreatitis (P) organoids and hypothesised that this contributes to the enhanced inflammatory phenotype in pancreatic stellate cells (PSCs) when cultured with PDAC organoids. We have now directly tested this hypothesis (with main findings summarised in **Figure R2.1**).

1) We first examined *Tnf* expression, given its known role in inflammatory PSC activation (e.g., PMID 30366930). *Tnf* expression was not significantly different between PDAC and P organoid in monocultures or PSC co-cultures (**Figure R2.1A**), supporting a specific role for IL-1 signalling. These data are included in **new Extended Data (ED) Figure 7C**.

2) We found that PDAC organoids secrete significantly higher levels of IL-1α protein than P organoids, while producing comparable levels of TGF-β (**Figure R2.1B**). These data are included in **new Figure 6F** of the revised manuscript.

3) To directly test causality while avoiding confounding effects on epithelial cells, we used a validated organoid-conditioned media approach (e.g., PMID 30366930) combined with IL-1α neutralisation. In line with RNA-seq data of organoid/PSC co-cultures, conditioned media from both P and PDAC organoids induced inflammatory markers (*Il1a, Cxcl1, Csf3, Lif, Il6*) in PSCs, with a stronger response to PDAC-derived media (**Figure R2.1C**). Importantly, IL-1α neutralisation significantly attenuated this inflammatory phenotype—most prominently in PDAC organoid-conditioned media. Consistently, genetic ablation of *Il1r1* in PSCs impaired inflammatory marker induction in response to both P and PDAC organoid-conditioned media (**Figure R2.1D**). These results demonstrate that increased IL-1α secretion by PDAC epithelial cells is a key driver of enhanced inflammatory PSC activation relative to P conditions. These data are included in **new ED Figures 7G-H** of the revised manuscript.

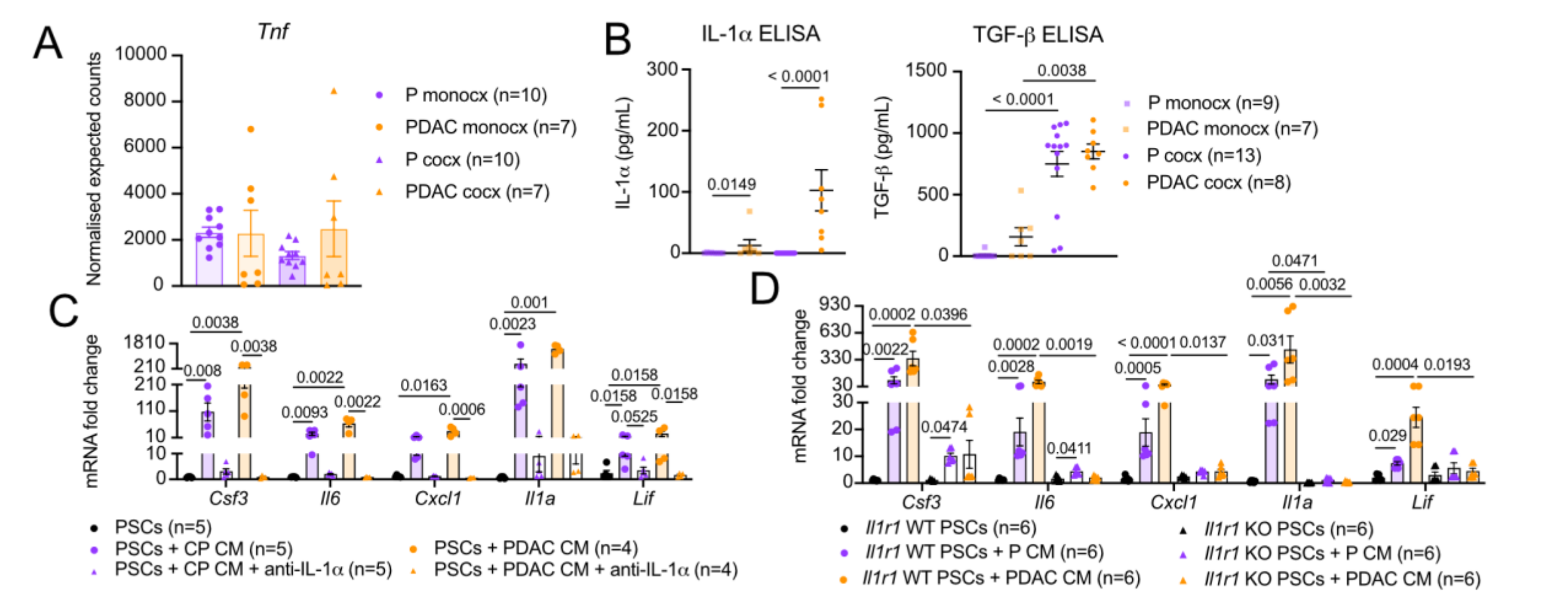

Figure R2.1. A. RNA-seq expression of *Tnf* in P or PDAC monocx and P or PDAC cocx. Results show mean ± SEM. No significance as assessed by Kruskal-Wallis test. **B.** ELISA of interleukin 1 alpha (IL-1α, left) and transforming growth factor beta (TGF-β, right) from media of P monocx (n=9), PDAC monocx (n=7), P cocx (n=13) and PDAC cocx (n=8). Results show mean ± SEM. *P adj*, Kruskal-Wallis test. **C.** qPCR analysis of PSCs cultured for 4 days in Matrigel with reduced media or with P or PDAC organoid–conditioned media (CM) in the presence of a neutralizing antibody targeting IL1α or an IgG control. Results show mean ± SEM. *P adj*, Kruskal-Wallis test. **D.** qPCR analysis of *Il1r1* wild-type (WT) or *Il1r1* knockout (KO) PSCs cultured for 4 days in Matrigel with reduced media or with P or PDAC organoid–conditioned media. Results show mean ± SEM. *P adj*, Kruskal-Wallis test.

B) Mechanistic basis of differential stromal responses to P versus PDAC epithelial-derived cues underlying inflammatory or myofibroblastic phenotypes.

1) CellChat analysis of the single-cell RNA-sequencing (scRNA-seq) dataset from organoid/multi-stroma co-cultures revealed increased communication between PDAC malignant cells and PSCs, but reduced interactions with mixed-fibroblasts (corresponding to the fibroblasts described in the original manuscript in response to **Critique R2.3**) and mesothelial cells, relative to P ductal cells (**Figure R2.2A**). This analysis also identified differential signalling through TGF-β, fibronectin (FN1) and IL-1 pathways in PDAC versus pancreatitis co-cultures (**Figure R2.2B-D**). In the IL-1 pathway, *Il1a* was inferred to be the dominant ligand mediating epithelial-to-stromal signalling in PDAC, consistent with our functional experiments. These data are included in **new ED Figures 9A-D** of the revised manuscript.

2) To determine whether stromal cell origin influences responsiveness to epithelial cues, and to further understand how the presence of mixed-fibroblasts and mesothelial cells helps mirror the *in vivo* enrichment of myofibroblastic features in multi-stroma co-cultures, we treated PSCs, mixed-fibroblasts and mesothelial cells with TGF-β or IL-1α to induce myofibroblastic or inflammatory markers, respectively. Mixed-fibroblasts and mesothelial cells were more responsive to TGF-β, upregulating myofibroblastic markers (*Tgfb1*, *Ctgf*, *Ncam1* – encoding for CD56) more significantly than PSCs (**Figure R2.2E**). Conversely, PSCs responded more strongly to IL-1α, inducing inflammatory markers (*Il1a*, *Il6*, *Csf3*) more significantly compared to mixed-fibroblasts and mesothelial cells (**Figure R2.2F**). These data are included in **new ED Figures 9E-F**.

3) Consistent with these findings, IL1-α treatment induced higher STAT3 activation, a known downstream effector of IL-1 signalling in PDAC CAFs (e.g., PMID 30366930), in PSCs compared to other stromal populations (**Figure R2.2G**). STAT3 activation was also higher in PSCs cultured with PDAC organoid-conditioned media, whereas mixed-fibroblasts and mesothelial cells showed stronger STAT3 activation in response to P organoid-conditioned media (**Figure R2.2H**). These data are included in **new ED Figures 9G-H** of the revised manuscript.

Together, these data suggest that mixed-fibroblasts and mesothelial cells are less responsive to PDAC-secreted IL-1α, a major driver of iCAF activation (e.g., PMID 30366930), thereby favouring enrichment of myofibroblastic features within the PDAC stroma. Furthermore, our data indicate that additional factors secreted by pancreatitis ductal cells promote a stronger inflammatory phenotype in mixed-fibroblasts and mesothelial cells, as reflected by enhanced STAT3 activation. This difference may contribute to why pancreatitis multi-stroma, but not PSC-only, co-cultures exhibit a more inflammatory phenotype than PDAC co-cultures. We therefore investigated this further:

4) We focused on leukemia inhibitory factor (LIF), which can mediate STAT3 activation in PDAC iCAFs (e.g., PMID 30366930), as a candidate epithelial-derived driver of inflammatory signalling in pancreatitis. *Lif* expression was higher in P organoids compared to PDAC organoids and was particularly enriched in pancreatitis Ductal 2 cells (**Figures R2.2I-J**). Consistently, LIF treatment robustly activated STAT3 in mixed-fibroblasts and mesothelial cells (**Figure R2.2K**). These data are included in **new ED Figures 9I-K** of the revised manuscript.

Together, these mechanistic data demonstrate that epithelial cells from pancreatitis and PDAC drive distinct stromal programmes through lineage-specific responsiveness to IL-1α, TGF-β,

and LIF signalling. These findings further highlight the need to consider stromal heterogeneity in modelling and understanding of these diseased conditions.

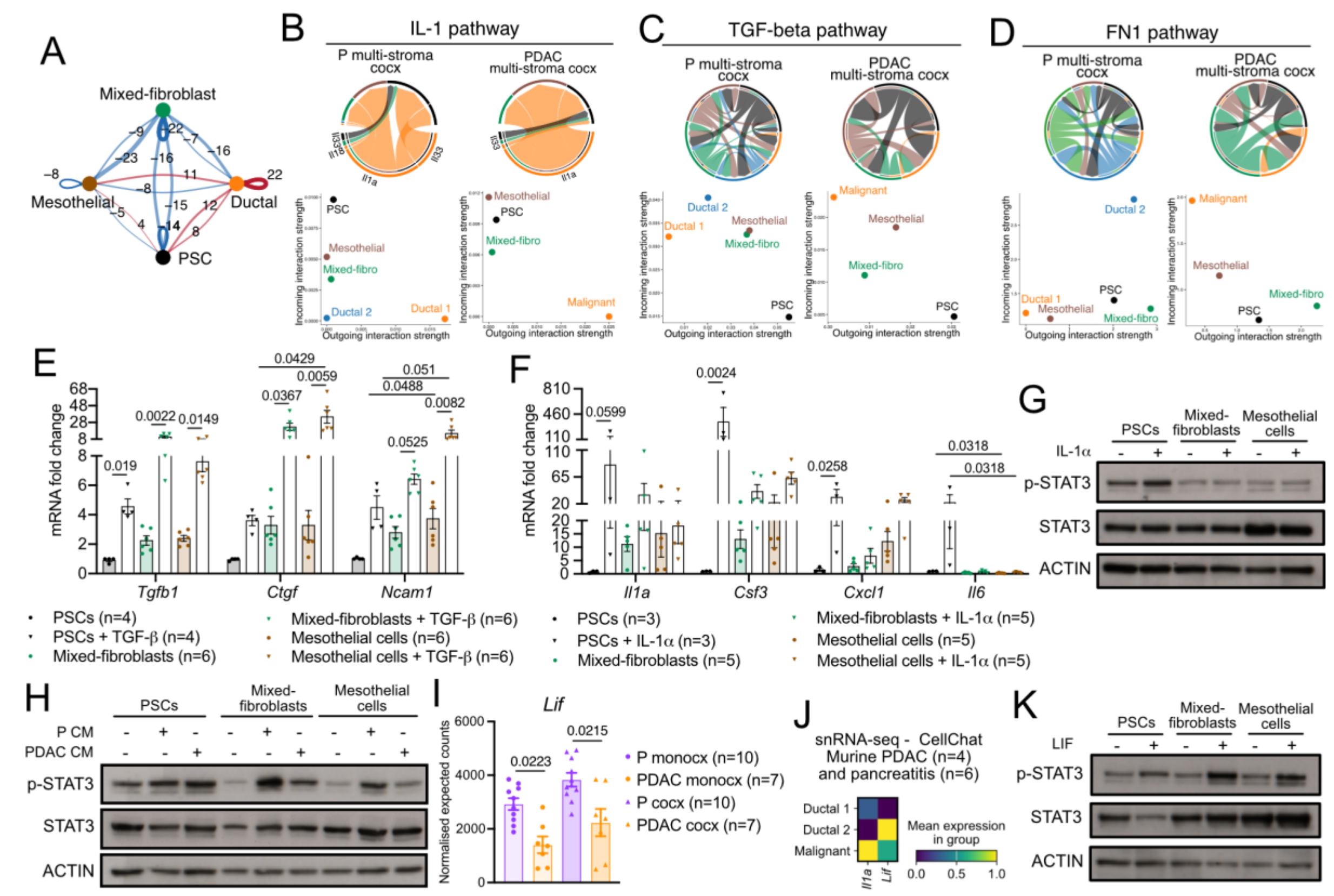

Figure R2.2. A. Cell-cell communication analysis using CellChat showing the difference in connections between cell types in PDAC organoid/multi-stroma co-cultures relative to P organoid/multi-stroma co-cultures, as assessed by scRNA-seq. Blue indicates connection that are lost in PDAC. Red indicates connections that are gained in PDAC. The thickness of the connection reflects the number of interactions. **B-D.** Top panels: selected IL-1 **(B)**, TGF-β **(C)** and fibronectin/FN1 **(D)** pathways in PDAC organoid/multi-stroma co-cultures and P organoid/multi-stroma co-cultures, as assessed by CellChat analysis. Bottom panels: Scatter plots showing the cell types most involved in outgoing and incoming signalling. **E.** qPCR analysis of *Tgfb1, Ctgf* and *Ncam1* in PSCs (n=4), mixed-fibroblasts (n=6) and mesothelial cells (n=6) cultured in Matrigel with reduced media in the presence or absence of 20 ng/mL TGF-β for 4 days. Results show mean ± SEM. *P adj*, Kruskal-Wallis test. **F.** qPCR analysis of *Il1a*, *Csf3, Cxcl1* and *Il6* in PSCs (n=3), mixed-fibroblasts (n=5) and mesothelial cells (n=5) cultured in Matrigel with reduced media in the presence or absence of 1-2 ng/mL IL-1α for 4 days. Results show mean ± SEM. *P adj*, Kruskal-Wallis test. **G.** Western blot of p-STAT3 and STAT3 in PSCs, mixed fibroblasts and mesothelial cells cultured in Matrigel with reduced media with or without 1 ng/mL IL-1α for 4 days. ACTIN, loading control. The samples were obtained and processed simultaneously. **H.** Western blot of p-STAT3 and STAT3 in PSCs, mixed fibroblasts and mesothelial cells cultured in Matrigel with reduced media, P organoid CM or PDAC organoid CM for 4 days. ACTIN, loading control. **I.** RNA-seq expression of *Lif* in P or PDAC monocx and cocx. Results show mean ± SEM. *P adj*, Kruskal-Wallis test. **J.** Heatmap of scaled expression of *Lif* in murine pancreatitis and PDAC ductal clusters. Data are scaled such that the cluster with the lowest average expression = 0 and the highest = 1 for each gene. **K.** Western blot of p-STAT3 and STAT3 in PSCs, mixed fibroblasts and mesothelial cells cultured in Matrigel with reduced media with or without 5 ng/mL LIF for 4 days. ACTIN, loading control.

Finally, we have expanded the text to more fully contextualise these findings in relation to established CAF activation pathways, including IL1-STAT3-driven inflammatory fibroblasts and TGF-β-driven myofibroblastic differentiation.

Critique R2.2: *Moreover, while pancreatitis and PDAC epithelial cells favor distinct fibroblast states, it remains unclear whether these states are modifiable. In particular, it is not addressed whether, and to what extent, PDAC cells can reprogram fibroblasts that were previously shaped by a pancreatitis environment. This is particularly relevant given that pancreatitis is a major risk factor for PDAC. To address this gap, the authors should perform sequential co-culture experiments, in which fibroblasts are first co-cultured with pancreatitis organoids and subsequently co-cultured with PDAC organoids. Such a sequential co-culture model would more closely mimic patients who have chronic pancreatitis and later develop PDAC. From a therapeutic standpoint, whether pancreatitis-educated fibroblasts resist or permit PDAC-driven reprogramming has major implications. Resistance would suggest that inflammatory fibroblasts in the pancreatitis environment may be protective, arguing against broad fibroblast-targeting strategies. In contrast, if pancreatitis-associated fibroblasts could be easily hijacked by PDAC cells, this would point to an early window for fibroblast-targeted therapy to prevent establishment of tumor-promoting CAFs, i.e., an interception strategy.*

Response R2.2: We thank the Reviewer for this suggestion, which addresses an important conceptual question regarding the plasticity of fibroblast states during disease progression from pancreatitis to PDAC. To determine whether stromal cells initially shaped by inflammatory cues respond differently to malignant signals than naïve stromal cells, we pursued two strategies.

1) We first established mixed-fibroblast lines from mice with pancreatitis (hereon termed 'P-mixed-fibroblasts'), and confirmed their elongated morphology as well as the absence of immune, endothelial, pericyte or epithelial contamination (**Figures R2.3A-D**). In 2D culture, P-mixed-fibroblasts resembled naïve mixed-fibroblasts, exhibiting significantly higher expression of myofibroblastic (*Acta2*, *Col1a1*) and mesothelial (*Wt1*, *Krt19*) markers than PSCs (**Figure R2.3E**). When cultured with organoid-conditioned media, P-mixed-fibroblasts upregulated inflammatory markers more robustly in response to P organoids than to PDAC organoids, although the magnitude of these changes was reduced compared to naïve stromal populations (**Figures R2.3F-H** and **Figure R2.1C**). These data are included in **new ED Figures 10A-H** of the revised manuscript.

2) To directly address the Reviewer's suggestion, we established a sequential co-culture system. Naïve multi-stroma (PSCs, mixed-fibroblasts and mesothelial cells) were first cultured with P organoids, after which stromal populations were isolated by GFP (for PSCs) and TdTomato (for mixed-fibroblasts and mesothelial cells) sorting and re-plated at a 1:2 ratio with PDAC organoids (with details in revised Methods section). We then performed bulk RNA-sequencing on flow-sorted stromal cells from these sequential pancreatitis-to-PDAC (P-to-PDAC) multi-stroma co-cultures, alongside standard P and PDAC multi-stroma co-cultures (**Figure R2.3I**). Projection along a transcriptional axis from P to PDAC revealed that P-to-PDAC multi-stroma occupies an intermediate state between P and PDAC multi-stroma (**Figure R2.3J**). Relative to P multi-stroma, P-to-PDAC multi-stroma upregulated both myofibroblastic (e.g., TGF-β signalling) and inflammatory (e.g., NFκB) programmes yet remained less myofibroblastic than multi-stroma in PDAC co-cultures (**Figures R2.3K-L**). These data are included in **new ED Figures 10I-L** and **new Supplementary Table 15**. We also added a discussion points highlighting the implications of this partial reprogramming (lines 909-917).

Together, these findings demonstrate that fibroblasts conditioned by pancreatitis are not irreversibly fixed but can be partially reprogrammed by malignant epithelial cues, while retaining an inflammatory "memory" of their prior state. This intermediate phenotype could have important

implications for understanding PDAC evolution in patients with chronic pancreatitis and for identifying potential windows for stromal-targeted interception strategies.

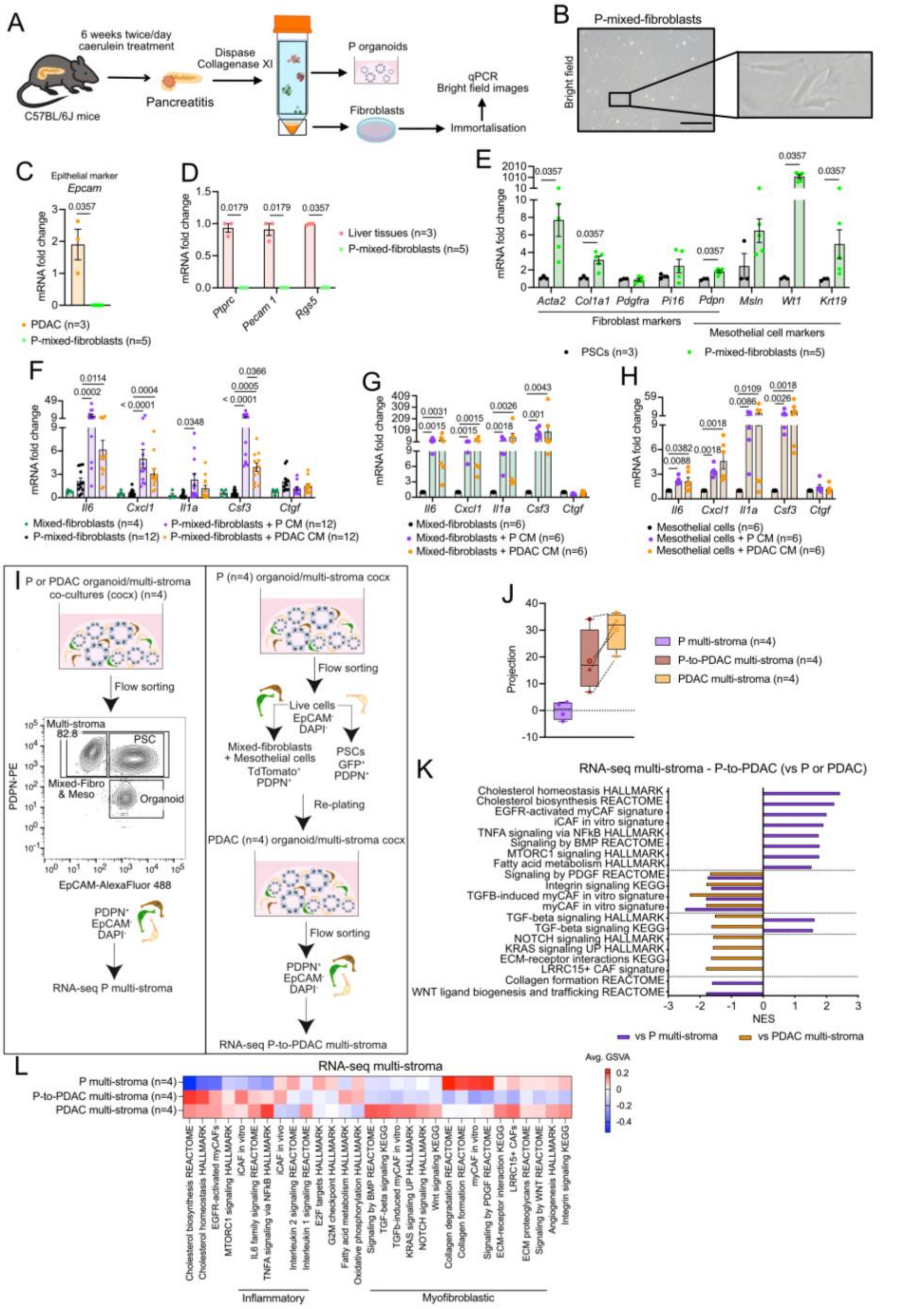

Figure R2.3. A. Schematic illustrating how pancreatitis mixed-fibroblast lines (hereon, P-mixed-fibroblasts) were generated and validated. **B.** Bright field images of murine P-mixed-fibroblasts cultured in 2D. Scale bar = 100 μm. Bottom: Insert shows magnification. **C-D.** RNA expression levels of epithelial, immune, endothelial and pericyte markers in P-mixed-fibroblasts (n=5) cultured in 2D and in liver tissues (n=3). Results show mean ± SEM. *P adj*, Kruskal-Wallis test. **E.** RNA expression levels of fibroblast and mesothelial cell markers in PSCs (n=3) and P-mixed-fibroblasts (n=5) cultured in 2D. Results show mean ± SEM. *P adj*, Kruskal-Wallis test. **F.** qPCR analysis of mixed-fibroblasts cultured for 4 days in Matrigel with reduced media (n=4), as well as in P-mixed-fibroblasts cultures in Matrigel with reduced media (n=12) or with P (n=12) or PDAC (n=12) organoid CM. Results show mean ± SEM. *P adj*, Kruskal-Wallis test. **G.** qPCR analysis of mixed-fibroblasts cultured for 4 days in Matrigel with reduced media (n=6), or with P (n=6) or PDAC (n=6) organoid CM. Results show mean ± SEM. *P adj*, Kruskal-Wallis test. **H.** qPCR analysis of mesothelial cells cultured for 4 days in Matrigel with reduced media (n=6), or with P (n=6) or PDAC (n=6) organoid CM. Results show mean ± SEM. *P adj*, Kruskal-Wallis test. **I.** Schematic of RNA-seq analyses of flow-sorted multi-stroma from P organoid/multi-stroma cocx, PDAC organoid/multi-stroma cocx and sequential P-to-PDAC organoid/multi-stroma cocx. **J.** Projection scores of P multi-stroma, P-to-PDAC multi-stroma and PDAC multi-stroma, as assessed by RNA-seq. Data are presented as a box-and-whisker plot where the box represents the 25th-75th percentiles, the whiskers represent min to max and the middle line represents the median. **K.** Significantly upregulated and downregulated pathways identified by GSEA of P-to-PDAC multi-stroma (n=4) compared to P multi-stroma (n=4) or PDAC multi-stroma (n=4), as assessed by RNA-seq. **L.** Heatmap showing GSVA enrichment scores (averaged per group) of selected pathways in P multi-stroma, P-to-PDAC multi-stroma and PDAC multi-stroma, as assessed by RNA-seq.

Critique R2.3: *The experimental design is comprehensive, employing both in vivo mouse models and in vitro organoid co-cultures, as well as human tissue data. The use of multiple independent organoid lines (five pancreatitis-derived organoid lines and two PDAC organoid lines) supports reproducibility. That said, the authors should ensure that all key findings are supported by adequate biological replicates and statistical analysis. For example, when quantifying fibroblast versus epithelial markers in co-cultures or in mice, are results consistent across different organoid lines and animals? In the co-culture experiments, it appears that pancreatitis organoids had a higher fibroblast-to-epithelial ratio than PDAC organoids – it would be helpful to report the replicate numbers and variance for such observations. Additionally, the study might benefit from including a normal pancreas baseline. While the focus is on disease vs. inflammation, showing how "normal" organoid–stroma interactions look would contextualize the changes seen in pancreatitis and PDAC. Minor point: please clarify in the text whether "fibroblasts" (as opposed to PSCs) refers to pancreatic fibroblasts isolated from healthy or other sources.*

Response R2.3: We thank the Reviewer for the opportunity to further clarify our statistical reporting and terminology, and to provide additional analyses.

1) We have clarified in the Statistical Analysis section that each data point shown in graphs represents an independent sample/measurement (e.g., distinct organoid-cell line combinations, individual mice) unless otherwise specified (only relevant to new ED Figure 7H). We have also added replicate numbers to figure legends where this information was not indicated in the original manuscript. For example, in quantifications of fibroblast versus epithelial markers, we clarified that each data point represents a distinct organoid-PSC combination (e.g., Figure 5D of the revised manuscript) or an individual mouse (e.g., ED Figure 6C of the revised manuscript). Finally, as also stated in the original manuscript, data are displayed as mean +/- standard error of the mean (SEM). To further address the Reviewer's comment and strengthen our results on fibroblast-to-epithelial ratios in pancreatitis and PDAC, we performed additional quantifications. Specifically, we analysed stromal/epithelial abundance in tissues using our optimised PDPN stain (included in **Response R1.1**) and CK19 immunohistochemistry (**Figure R2.4A**), which independently validates our original PDGFRα/CK19 immunohistochemistry-based analyses (**revised ED Figure 6C**). We additionally quantified stromal abundance in organoid/multi-stroma co-cultures using PDPN/CK19 and PDGFRα/CK19 multiplex immunofluorescence, and PDPN/EpCAM flow-sorting; **Figures R2.4B-C**).

Across these *in vivo* and *in vitro* analyses, we consistently observed a higher stromal abundance in pancreatitis organoid/multi-stroma co-cultures compared with PDAC organoid/multi-stroma co-cultures, in agreement with our original PSC co-culture findings. These data are included in **new Figures 7B** and **7D** and **new ED Figures 6D** and **8E-F**.

2) We appreciate the Reviewer's suggestion to include a normal pancreas baseline to contextualise epithelial cell-stromal interactions. We now provide more context and analyses to support our decision to focus the *in vitro* studies on pancreatitis and PDAC. In the original manuscript, immunohistochemical analyses demonstrated only a minor presence of ductal (CK19$^+$) and fibroblast (PDPN$^+$, αSMA$^+$, PDGFRα^+, collagen$^+$) populations in the normal pancreas. To further substantiate this observation, we performed Visium HD spatial transcriptomics on murine normal pancreas tissue (n=3). These data confirmed that both ductal cells and fibroblasts constitute minor populations within an acinar-dominated environment (**Figure R2.4D**). In parallel, in response to Reviewer #1, we performed Visium HD spatial transcriptomics on murine PDAC and pancreatitis

tissues. These analyses confirmed a high abundance of epithelial cells and fibroblasts in both PDAC and pancreatitis (included in **Response R1.2**). Together, these findings support our decision to focus the *in vitro* analyses on pancreatitis- and PDAC-associated fibroblast reprogramming, where epithelial-stromal interactions are prominent. In addition, we leveraged the Visium HD dataset to provide *in vivo* context for fibroblast reprogramming in PDAC and pancreatitis relative to fibroblasts in the normal pancreas (**Figure R2.4E**). All Visium HD data are included in **new Figures 2J-L**, **new ED Figures 2O-R** and **new Supplementary Table 5**.

3) We also appreciate the Reviewer's request for clarification regarding stromal cell generation and fibroblast terminology. In the original manuscript, we stated that *fibroblast and mesothelial cell lines were established from normal C57BL/6J pancreata*, consistent with PSC isolation protocols (**ED Figure 7H**). However, we acknowledge that this was not described with sufficient clarity. We have now explicitly clarified this point in both the Methods and Results sections. In addition, we have revised the terminology from 'fibroblasts' to 'mixed-fibroblasts' to better distinguish these *in vitro* populations from *in vivo* fibroblast populations and to avoid potential confusion. We retain the term 'fibroblasts' when referring to *in vivo* populations, in line with prevailing terminology in the field. We nevertheless welcome further guidance on nomenclature from the editors and reviewers.

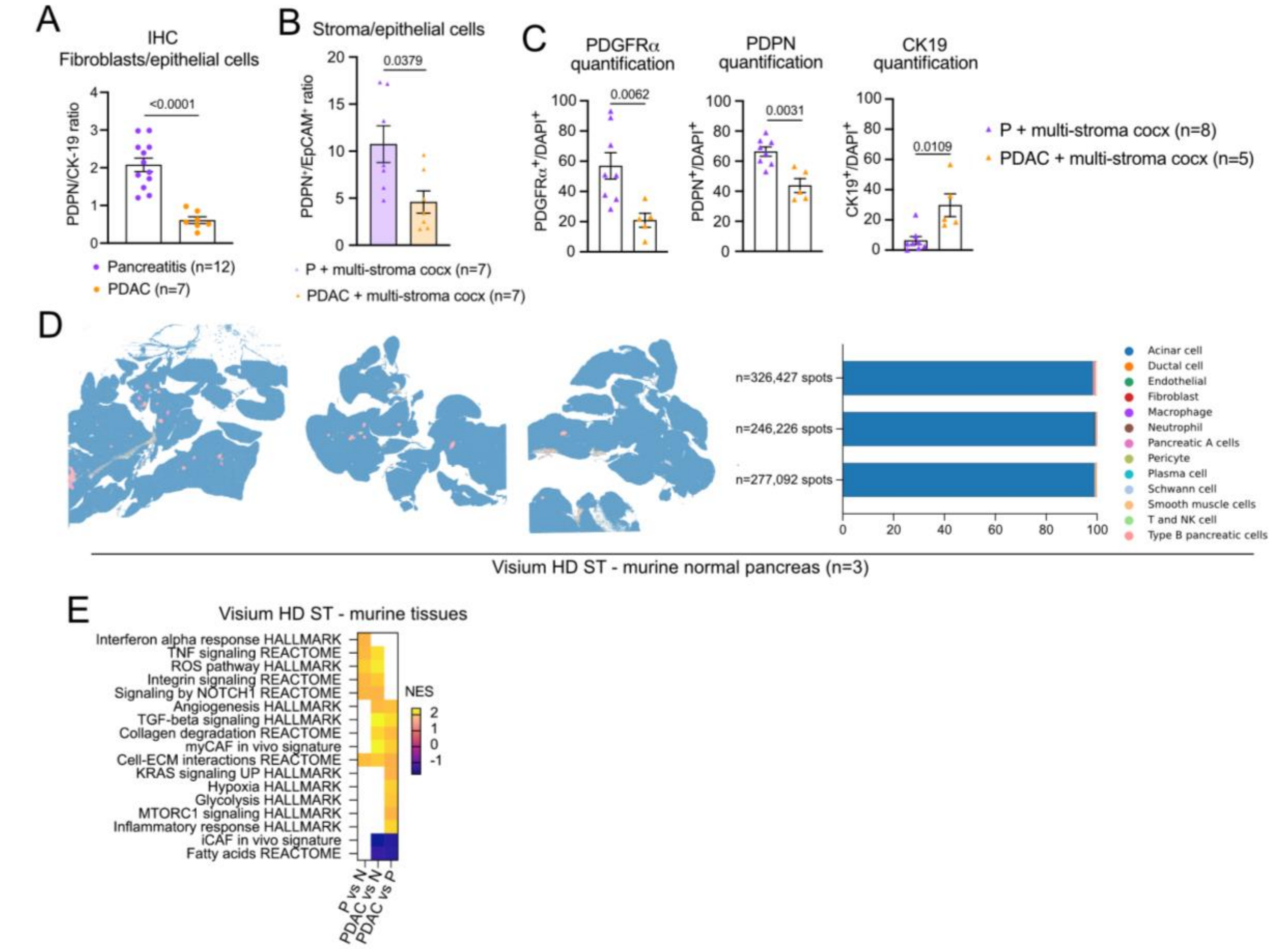

Figure R2.4. A. Fibroblast (PDPN$^+$)/epithelial cell (CK19$^+$) ratio as assessed by immunohistochemical analysis of pancreatitis (n=12) or PDAC (n=7) murine tissues (from Figure 2). Results show mean ± SEM. *P*, Mann-Whitney test. **B.** Stroma (PDPN$^+$EpCAM$^-$)/epithelial cell (EpCAM$^+$PDPN$^-$) ratio as assessed by flow sorting of P (n=7) or PDAC (n=7) multi-stroma cocx after 3.5 days in culture. Results show mean ± SEM. *P*, Mann-Whitney test. **C.** Multiplex immunofluorescence quantification of PDGFRα$^+$CK19$^-$ (left), PDPN$^+$CK19$^-$ (middle) and CK19$^+$PDPN$^-$ (right) DAPI$^+$ cells in P (n=8) or PDAC (n=5) organoid/multi-stroma co-cultures. Results show mean ± SEM. *P*, Mann-Whitney test. **D.** Visium HD spatial transcriptomics (ST) slide images (left) and corresponding deconvoluted cell type contribution (right) represented as bar plots showing the proportions of the different cell clusters in each murine tissue of normal pancreas. **E.** Significantly upregulated and downregulated pathways identified by Gene Set Enrichment Analysis of pancreatitis relative to normal fibroblasts (left), PDAC CAFs compared to normal fibroblasts (middle) and PDAC CAFs compared to pancreatitis fibroblasts (right), as assessed by pseudobulk analysis.

Critique R2.4: *A strength of the study is the integration of mouse and human data to demonstrate relevance of the models. The authors show that many fibroblast gene signatures from human pancreatitis and PDAC are captured in the corresponding mouse models, though the overlap is far from complete (only ~28% of human PDAC CAF markers overlap with mouse CAF markers, vs ~54% overlap for pancreatitis fibroblast markers). This partial concordance should be better highlighted as it suggests that, while the murine models mimic broad patterns, certain human-specific fibroblast programs are missing. As such the manuscript would benefit from a more nuanced discussion of these species differences. For instance, are there fibroblast subtypes present in human PDAC that mice lack, or vice versa? The authors conclude that the mouse models "significantly capture" human fibroblast markers, which is fair, but they should also acknowledge the limitations – e.g., the 72% of human PDAC CAF genes that did not overlap. It would be constructive to discuss possible reasons for the disparities … differences in disease stage, immune context, or technical factors in single-cell data (the immune issue is perhaps the most impt to emphasize). Encouragingly, the cross-species analysis extends to epithelial cells, identifying analogues of "Ductal 1" vs "Ductal 2" populations, which strengthens the validity of the organoid approach. Overall, the inclusion of human data increases the translational relevance of the findings, but the authors should avoid over-interpretation of the cross-species concordance and should clarify where the models might fall short of human biology as some may interpret the message as these models would replace other types of models … which they do not … all models bring visibility to the biological complexity of human disease. To conclude otherwise is somewhat naïve.*

Response R2.4: We thank the Reviewer for this thoughtful and important critique. We agree that the partial concordance between human and mouse fibroblast programmes warrants a more nuanced discussion, and we appreciate the opportunity to address this explicitly in the revised manuscript. In response, we performed additional analyses and expanded the Discussion to better contextualise both the strengths and limitations of the murine models.

1) To better understand which human fibroblast programmes are captured, and which are not, we performed subclustering analyses of human PDAC CAFs and pancreatitis fibroblasts from the scRNA-seq dataset included in the original manuscript (**new ED Figures 1E-H** of the revised manuscript, including **Figures R2.5A-B**). In parallel, we subclustered murine PDAC CAFs from the single-nuclei RNA-sequencing (snRNA-seq) dataset (**new ED Figures 3E-G**, including **Figures R2.5C-D**).

Mapping murine fibroblast signatures (top 50 markers relative to the other fibroblast subsets) onto the human scRNA-seq dataset, using AUCell scoring, revealed that Fibro 2 and Fibro 3 aligned more closely with human pancreatitis fibroblasts than Fibro 1 and Fibro 4, whereas murine CAF signatures better aligned with the human myCAF cluster. These data are included in **new ED Figures 4A-B** of the revised manuscript (representative UMAPs are shown in **Figures R2.5E-F**).

Reciprocal mapping of human fibroblast signatures (i.e., top 50 markers compared to other fibroblast subsets) onto the murine snRNA-seq dataset demonstrated that the human iCAF signature was better represented in murine PDAC CAFs than the human myCAF signature. Among human pancreatitis fibroblast populations, the *DCN*^{High}*DPP4*^{High} subset was most accurately captured by murine models. These data are included in **new ED Figures 4C-D** of the revised manuscript (UMAP examples are shown in **Figures R2.5G-H**).

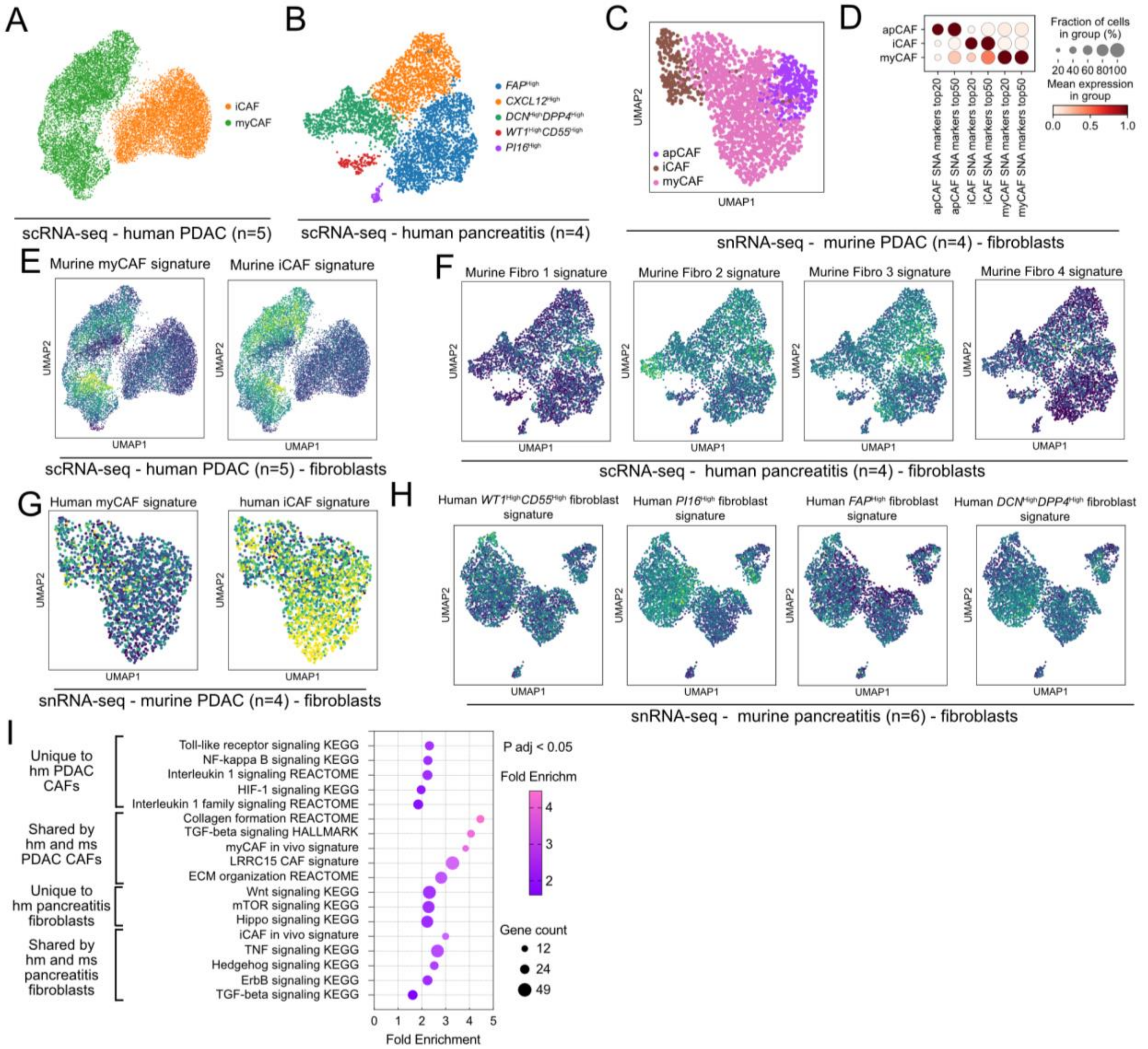

Figure R2.5. A-B. UMAP plots of fibroblasts from human PDAC (**A**, n=5) or human chronic pancreatitis (**B**, n=4). Different subsets are colour coded. **C.** UMAP plot of fibroblasts from murine PDAC (n=4). Different subsets are colour coded. **D.** Dot plot of scaled Aucell score enrichment of PDAC CAF subtype signatures in each PDAC fibroblast cluster. CAF subtype signatures (i.e., top 20 and top 50 markers) were derived from the snRNA-seq dataset of PDAC tumours from Mucciolo et al. **E.** UMAPs of murine PDAC CAF signatures (human orthologs), as identified by snRNA-seq, enriched in human fibroblasts from the PDAC scRNA-seq dataset. **F.** UMAPs of murine pancreatitis fibroblast signatures (human orthologs), as identified by snRNA-seq, enriched in human fibroblasts from the chronic pancreatitis scRNA-seq dataset. **G.** UMAPs of human PDAC CAF signatures (mouse orthologs), as identified by scRNA-seq, enriched in murine fibroblasts from the PDAC snRNA-seq dataset. **H.** UMAPs of human pancreatitis fibroblast signatures (mouse orthologs), as identified by scRNA-seq, enriched in murine fibroblasts from the pancreatitis snRNA-seq dataset. **I.** Significantly enriched pathways (P adj < 0.05) in genes 1) shared by murine and human PDAC CAFs, 2) unique to human relative to murine PDAC CAFs, 3) shared by murine and human pancreatitis fibroblasts, and 4) unique to human relative to murine pancreatitis fibroblasts.

2) We further assessed pathway-level conservation using enrichment analysis of shared genes (from **revised Supplementary Table 8**). These analyses revealed that murine pancreatitis models capture both myofibroblastic and inflammatory signatures of human pancreatitis (**Figure R2.5I**). In contrast, PDAC mouse models preferentially capture human myofibroblastic features and less robustly reflect inflammatory fibroblast signatures (**Figure R2.5I**). These data are included in **new ED Figure 4E** and **revised Supplementary Table 8** of the revised manuscript. These findings are particularly noteworthy given that patients in our PDAC cohort had an average age of > 65 years and that we have recently shown that aged mouse models of PDAC more closely recapitulate the biology of older patients, particularly inflammatory signatures (Araos et al *BioRxiv* 2026), than younger mice, which were used in this study. Therefore, incorporating different age diversity into future models will help fully capture the nuances of human fibroblast biology (lines 270-275).

3) Finally, we have substantially expanded the Discussion to explicitly acknowledge the limitations of cross-species comparisons and to avoid over-interpretation of model concordance. We now discuss potential contributors to partial overlap between human and murine fibroblast programmes, including differences in immune composition, age, and genetics (lines 895-905). We have also clarified that the murine and organoid-based models presented here are not intended to replace other experimental systems, but rather to provide complementary and tractable platforms that illuminate specific aspects of stroma heterogeneity and epithelial–stromal crosstalk in pancreatitis and PDAC (lines 932-935).

Together, these additions refine our interpretation of cross-species concordance, highlighting both the biological relevance and the limitations of current models, and emphasise that multiple experimental systems are required to fully capture the complexity of human stromal biology.

Critique R2.5: *That said, the use of multi-stromal organoid co-cultures (including PSCs, mesothelial cells, and fibroblasts) is innovative and goes beyond simpler two-cell type models. This approach clearly improved the recapitulation of in vivo fibroblast heterogeneity – the authors show that multi-stroma co-cultures capture pancreatitis and PDAC fibroblast features better than organoid cultures with PSCs alone. By incorporating multiple stromal sources, the authors also explore the intriguing idea that different CAF subpopulations may derive from different lineage origins (e.g. a mesothelial-derived subset vs. a PSC-derived subset). The data (e.g., transcriptional overlap of certain "Fibro" clusters with mesothelial signatures at ~85%) indeed suggest a mesothelial origin for some fibroblasts, consistent with recent lineage-tracing studies. However, the inference of distinct stromal origin contributions to CAF subtypes, while exciting, should be interpreted with great caution. In the absence of lineage tracing or specific in vivo fate-mapping, the link between a fibroblast's in vitro behavior and its origin remains indirect. The authors need to temper these conclusions – for example, phrasing that mesothelial cells "can give rise to" certain fibroblast subsets is an over-interpretation of the correlative data. It would suffice to say the results support the possibility of multiple origins. Additionally, the authors should discuss the limitations of the multi-stromal model: despite including three stromal cell types, it is critical to acknowledge that the model still omits immune and endothelial components that are known to influence fibroblast activation. The co-cultures were conducted for only ~3.5 days, which may not fully capture long-term differentiation or the maintenance of certain CAF phenotypes. Acknowledging these caveats would provide a balanced perspective on how closely the in vitro system can mimic the complex tumor microenvironment. More specifically, while immune signatures and inflammatory fibroblasts are discussed extensively (e.g. interferon response) in this study, immune cells are not directly*

incorporated into the co-culture model systems. Therefore, fibroblast subsets that depend on immune microenvironment may be missing or under-represented. The authors should acknowledge that the fibroblast states observed in their co-culture models may differ from those in immune microenvironment.

Response R2.5: We thank the reviewer for this thoughtful and important comment. We agree that conclusions regarding stromal cell origins must be interpreted with caution in the absence of *in vivo* lineage-tracing, and that the limitations of the multi-stromal organoid co-culture system should be made more explicit. We have therefore revised the manuscript accordingly.

1) We have amended the relevant Results section to avoid over-interpretation of stromal lineage contributions. Specifically, we removed the sentence implying a definitive mesothelial origin for specific fibroblast subsets (*this is consistent with a mesothelial cell of origin of these two fibro subsets*). We now also explicitly state that *in vivo* lineage-tracing experiments will be required to determine stromal origin contributions to pancreatitis fibroblast and PDAC CAF subtypes, and that our data support the possibility of multiple stromal cells of origin rather than definitive lineage relationships (lines 870-872).

2) We have expanded the Discussion to clearly articulate the scope of the multi-stromal organoid co-culture model and to highlight areas where future refinements may further enhance its interpretative power. We now highlight that, despite incorporating three stromal populations, the model does not include endothelial or immune cells, both of which are known to influence fibroblast activation and inflammatory signalling. Thus, immune-dependent fibroblast phenotypes may be under-represented in the current models. We also note that the relatively short duration of the co-culture experiments may limit the ability to capture long-term differentiation or maintenance of certain CAF phenotypes. To address these points, we added a dedicated paragraph outlining future directions, including the need for longer-term cultures and incorporation of additional cell types such as endothelial and immune cells (lines 938-946).

Together, these revisions clarify that the multi-stromal organoid co-culture model is not intended to replace other experimental systems, but rather to serve as a complementary platform that enables controlled interrogation of epithelial-stromal interactions while capturing aspects of stromal heterogeneity that are missed in simpler models.

Critique R2.6: *The manuscript touches on the translational implications of distinguishing fibroblast responses in chronic pancreatitis versus PDAC. This is a key point – identifying stromal differences could have diagnostic or therapeutic relevance. For example, the finding that pancreatitis is enriched in PDPN+/PDGFRα+ fibroblasts whereas PDAC has more αSMA+ myofibroblasts might be leveraged as a biomarker signature to help pathologists distinguish cancer from benign inflammation in preoperative biopsies. The authors also note distinct ligand–receptor interactions (such as PDGF and TGF-β signaling pathways) that are preferentially active in one condition over the other. One would like to know if these pathways could be targeted: e.g., could inhibiting TGF-β or PDGF signaling selectively alter the tumor-associated stroma without impairing normal injury repair? While such therapeutic experiments are beyond the scope of this study, the authors should expand their discussion on how the insights gained might guide clinical strategies. For instance, they could discuss whether any of the identified gene markers or pathways have existing inhibitors*

or if they correlate with patient outcomes in PDAC. Additionally, the co-culture platform itself could be a valuable tool for drug testing in the future – this could be mentioned as a forward-looking statement. Emphasizing the clinical context (e.g., chronic pancreatitis patients are at elevated risk for PDAC) and how understanding stromal reprogramming might inform surveillance or prevention would also strengthen the impact. In summary, the findings are potentially relevant to improving PDAC diagnosis and treatment, but the manuscript should do a slightly better job at articulating these real-world connections so that a broad Nature Cell Biology audience appreciates the significance.

Response R2.6: We thank the Reviewer for this suggestion and for helping us further highlight the translational relevance of distinguishing stromal responses in chronic pancreatitis versus PDAC. In response, we have expanded the Discussion to more clearly articulate how our findings may inform future diagnostic, surveillance, and therapeutic strategies.

1) We expanded the Discussion to highlight the potential of stromal-based biomarkers for PDAC, particularly in light of our new data spanning PDACs with distinct genetic profiles in response to Reviewer #3 (**revised Figure 3** and **revised ED Figures 3, 8** and **9**; e.g., **Figure R2.7**). Notably, our analyses suggest that stromal-specific markers are more consistently conserved across distinct PDAC genetic profiles than malignant epithelial markers. This observation provides a rationale for future development of stromal-based biomarker strategies for early PDAC detection in high-risk chronic pancreatitis patients. We now discuss how combinatorial marker approaches (e.g., FN1$^{+}\alpha$SMA^{+}PDGFRA^{-}CD34^{-}) may help distinguish malignancy from benign inflammation in clinical specimens, and how such markers could be evaluated for associations with patient outcomes and therapeutic responses (lines 917-923).

2) We also expanded the Discussion to emphasize how the distinct epithelial-stromal signalling pathways identified in our revised study (e.g., new Figure 6 and new ED Figures 7 and 9) could guide future clinical strategies aimed at both PDAC treatment and prevention. Specifically, we highlight that differential activation of pathways such as TGF-β, IL-1 and LIF/STAT3 signalling, which include components with existing pharmacological inhibitors, may represent opportunities for targeting tumour-associated stroma or intercepting fibroblast reprogramming in chronic pancreatitis patients at elevated risk for PDAC (lines 923-928).

3) Finally, we added a forward-looking statement noting that the organoid/multi-stroma co-culture system established here could serve as a platform for future screening of candidate therapeutically actionable pathways, enabling functional testing of epithelial-stromal interactions in a disease-specific manner (lines 935-937).

Together, these additions more clearly frame the clinical significance of our findings, highlighting how insights into disease-specific stromal reprogramming may inform PDAC diagnosis, surveillance in high-risk chronic pancreatitis patients, and the development of future stromal-targeted therapeutic strategies.

Critique R2.7: *The KPC PDAC model used in this study is driven by KRAS G12D expression and Trp53 LOH and R172H gain of function mutation. However, the pancreatitis model is driven by caerulein treatment and lack of genetic drivers. The author should acknowledge this limitation and*

discuss the extent to which these genetic drivers may induce fibroblast reprogramming.

Response R2.7: We agree with the Reviewer that this is an important consideration. In the original manuscript, we already noted this in the Discussion ("*Finally, it remains to be determined how fibroblast-epithelial cell cross-talks evolve from inflammation to malignancy in the context of oncogenic drivers.*"). We have now expanded the revised Discussion to more explicitly acknowledge that malignant epithelial genetic profiles can shape epithelial–stromal communication and to outline experimental strategies that could further address this in future studies. Specifically, we now discuss the potential of combining genetically engineered mouse models (GEMMs) of PDAC with inflammatory cues, for example by treating KC (*Kras*$^{LSL\text{-}G12D/+}$; *Pdx1*-Cre) GEMMs with caerulein, to delineate which epithelial–to-fibroblast signalling programmes are driven by oncogenic KRAS mutation versus by inflammation per se. This expanded discussion more clearly highlights that PDAC genetic drivers can influence epithelial–stromal crosstalk (lines 960-963).

To further explore this experimentally, and to reflect both the KRAS and p53 mutations present in the PDAC models analysed in our manuscript, we performed a pilot study in which 6-week-old KPC (*Kras*$^{LSL\text{-}G12D/+}$; *Trp53*$^{LSL\text{-}R172H/+}$; *Pdx1*-Cre) GEMMs and *Kras* wild-type littermate controls were treated with caerulein twice daily (**Figure R2.6A**). In KPC mice, caerulein administration led to rapid onset of severe clinical symptoms, necessitating humane endpoint criteria after one week, whereas control mice were unaffected. Notably, despite the young age of the animals and the short treatment duration, caerulein treatment was sufficient to induce tumour formation and pre-neoplastic lesions in KPC mice (**Figure R2.6B**).

While longer-term inflammatory treatments can be performed in KC mice and are commonly used to promote PDAC progression (e.g., PMID:22975374), we believe such experiments are more appropriately addressed in future work. This is because the current study is focused on comparing fibroblast responses to inflammatory cues in the absence of genetic mutations with responses to fully malignant epithelial cues, rather than dissecting intermediate disease states.

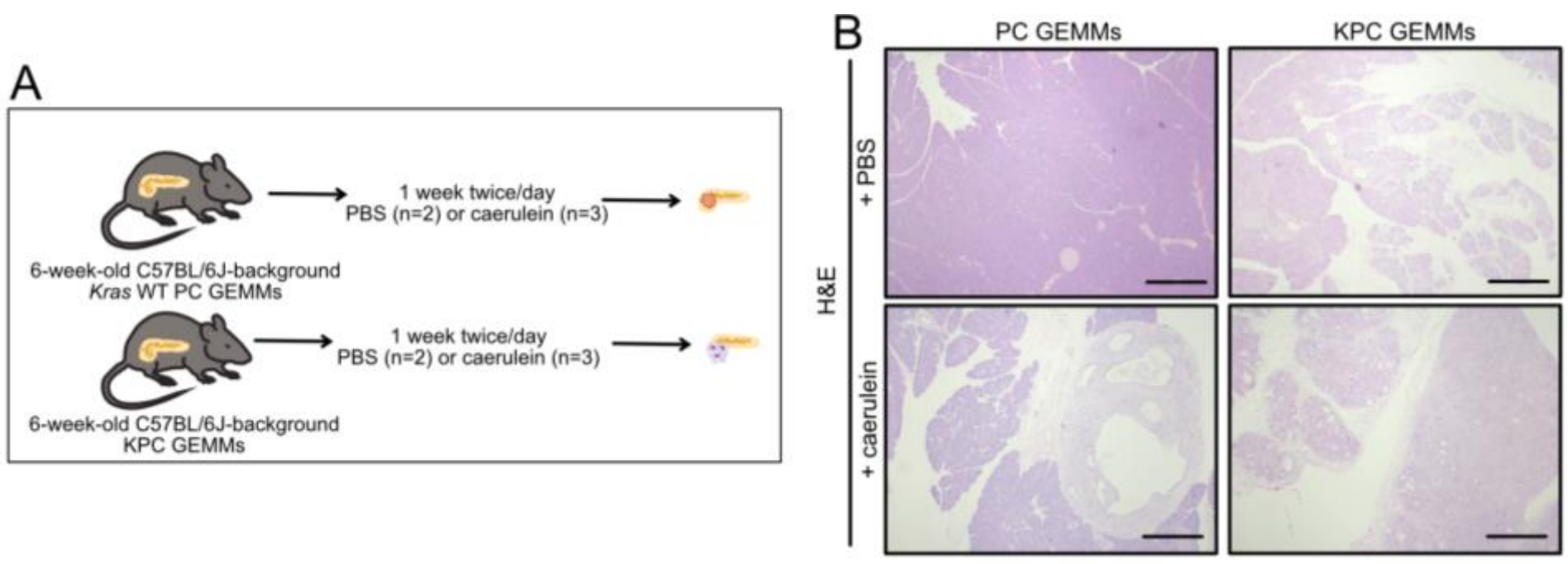

Figure R2.6. A. Schematic of models used for analysis of 1-week PBS- or caerulein-treated KPC GEMMs or *Kras* wild-type littermate controls (PC). **B.** Representative H&E images of the pancreata of 1-week PBS- or caerulein-treated KPC or PC GEMMs. Scale bars, 100 μm.

Critique R2.8: *In this study, fibroblast subsets are extensively characterized by transcriptomic analysis without functional characterization (e.g., ECM deposition for myCAFs, cytokine secretion for iCAFs). Thus, it is less clear whether the observed transcriptional differences of fibroblasts translate into biologically meaningful differences. The authors should validate the function of fibroblast subsets at minimal by IHC analysis*.

Response R2.8: We thank the Reviewer for highlighting the importance of protein-level validation of fibroblast subsets. In the original manuscript, we characterised PDAC and pancreatitis fibroblasts at the protein level using immunohistochemistry and multiplex immunofluorescence (IF), including analysis of PDPN, PDGFRα, αSMA, FN1, collagen (**revised Figures 2** and **8** and **revised ED Figure 2**). However, we agree that this analysis was limited in scope. To address this concern, we have substantially expanded our functional validation as follows:

1) **Expanded multiplex IF validation of disease-specific fibroblast markers in mouse models.** We performed extensive additional multiplex IF analyses to include markers identified as differentially expressed by transcriptomic profiling. These included myofibroblastic markers enriched in PDAC fibroblasts (*Tnc*, *Ncam1* encoding CD56, *Runx1*) and markers enriched in pancreatitis fibroblasts (*Cd34*) (**Figures R2.7A-H**). These data are included in **new Figures 8J-K** and **new ED Figures 9O-T** of the revised manuscript.

In addition, for both previously analysed markers (*Pdgfra*, *Acta2*, *Fn1*) and newly included markers, we extended multiplex IF analyses to two additional murine PDAC models ($KRAS^{G12D}$ p53-null and SMAD4-deficient $KRAS^{G12D}$ p53-mutant) in response to Reviewer #3 (**new ED Figures 9L-N** of the revised manuscript; **Figure R3.3**). These analyses further strengthened and generalised our original findings across PDAC models.

2) **Validation in human PDAC and pancreatitis tissues.** We next performed histological and multiplex IF analysis on human PDAC tissues and matched adjacent pancreas with chronic pancreatitis. These analyses revealed only modest differences in overall collagen deposition but demonstrated higher αSMA expression in PDAC CAFs and higher PDGFRα expression in pancreatitis fibroblasts (e.g., **Figures R2.7I-J**).

Consistent with our murine data, multiplex IF analysis of these tissues revealed an increased abundance of $CD34^{+}\alpha SMA^{-}PDFGR\alpha^{+}$ fibroblasts in chronic pancreatitis, whereas $FN1^{+}PDGFR\alpha^{-}\alpha SMA^{+}$ fibroblasts were enriched in PDAC tissues (**Figures R2.7K-N**). These data are included in **new Figures 1A-B** and **8L-O**, **new ED Fig. 1A** and **new Supplementary Table 1**.

3) ***In situ* validation of marker co-expression in pancreatitis fibroblast subsets.** Finally, to strengthen the relevance of transcriptomically defined fibroblast subsets, we performed multiplex RNA *in situ* hybridization combined with immunofluorescence in murine pancreatitis tissues. This approach confirmed co-expression of secreted factors, as identified by snRNA-seq analysis, which could mediate subset-specific functions. Specifically, we identified $Il34^{High}WT1^{High}$ Fibro 1, $Pi16^{High}PDGFR\alpha^{High}$ Fibro 2, $Vegfc^{High}Fap^{High}$ and $Cxcl12^{High}Fap^{High}$ Fibro 3 and $Muc16^{High}WT1^{High}$ Fibro 4 populations, as well as $SPP1^{Low}$ Ductal 1 and $SPP1^{High}$ Ductal 2 cells (e.g., **Figure R2.7O**). These data are included in **new Figures 3C-D** and **new ED Figures 3K** and **4L** of the revised manuscript.

We have also expanded the Discussion to address the potential functional roles of these fibroblast subsets based on their transcriptomic and spatial profiles (lines 973-985). Specifically, we

acknowledge that further work will be required to define the precise functions of fibroblast subsets in pancreatitis, while highlighting that our study provides insights into their potential roles by linking distinct fibroblast populations in both murine and human pancreatitis to markers with known functions in diseased and malignant contexts.

In summary, these new analyses substantially strengthen and expand our original conclusions, demonstrating that cross-species and cross-model transcriptomic analyses can be effectively leveraged to identify disease-specific fibroblast markers that translate to protein-level and spatial validation in both pancreatitis and PDAC tissues.

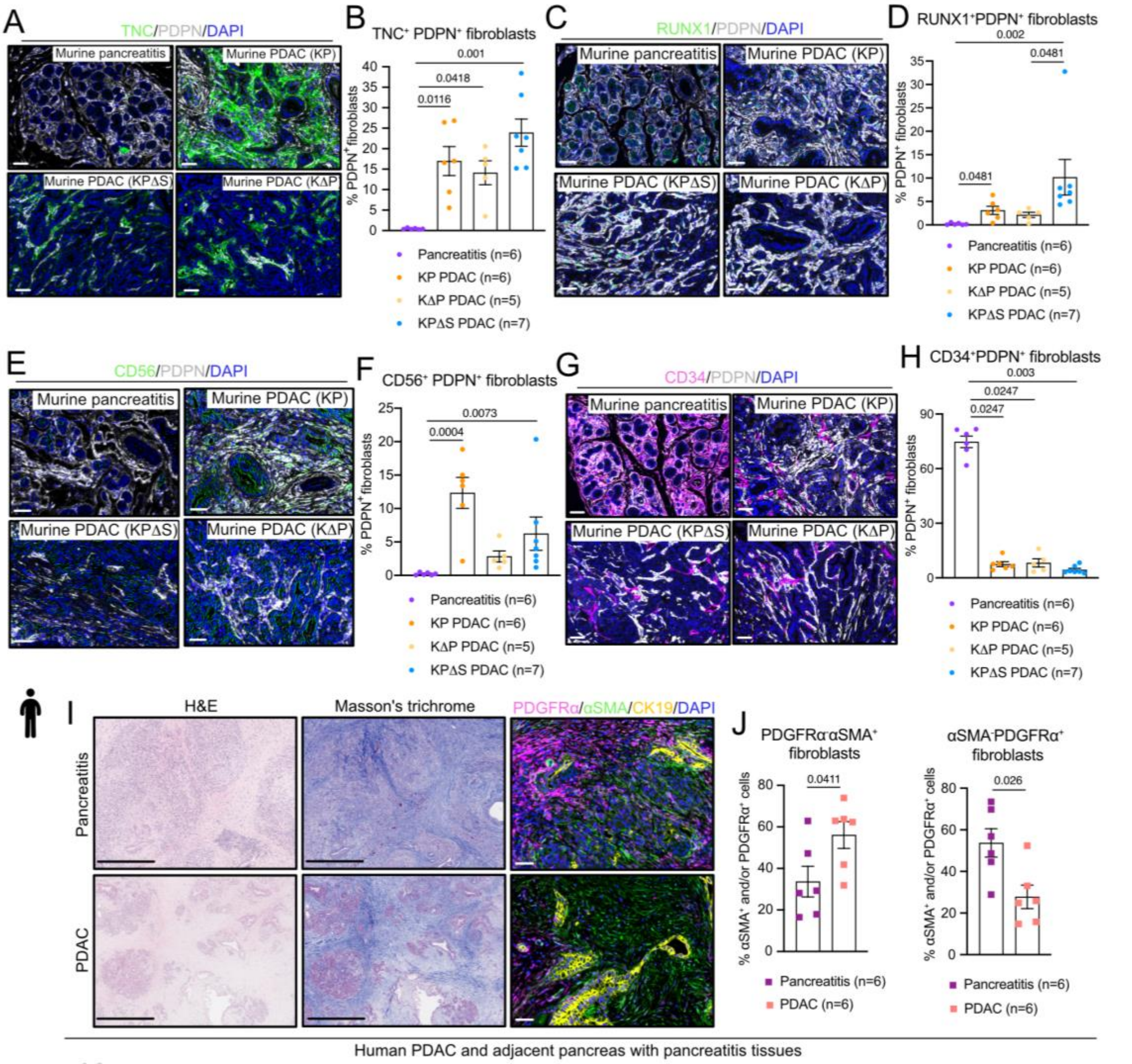

Figure R2.7 [Part 1]. A-H. Representative multiplex IF images of PDPN (white) and DAPI (blue), as well as tenascin (TNC, green, **A**) or RUNX1 (green, **C**) or CD56 (green, **E**) or CD34 (pink, **G**) in murine pancreatitis, PDAC tissues from KPC GEMMs (i.e., KP PDAC), as well as PDAC tissues derived from orthotopic transplantation models of KRAS^{G12D}, p53-null organoids (i.e., KΔP PDAC) or *Smad4*-deleted, KRAS^{G12D}, p53^{R172H} organoids (i.e., KPΔS PDAC). Scale bars = 50 μm. Quantification of TNC^{+}PDPN^{+}DAPI^{+}CK19^{-} cells **(B)** or nuclear-RUNX1^{+}PDPN^{+}DAPI^{+}CK19^{-} cells **(D)** or CD56^{+}PDPN^{+}DAPI^{+}CK19^{-} cells **(F)** or CD34^{+}PDPN^{+}DAPI^{+}CK19^{-} cells **(H)** relative to PDPN^{+}DAPI^{+}CK19^{-} fibroblasts in murine pancreatitis (n=6), KP PDAC (n=6), KΔP PDAC (n=5) and KPΔS PDAC (n=7) tissues. Results show mean ± SEM. *P adj*, Kruskal-Wallis test. **I.** Representative Hematoxylin & Eosin (H&E), Masson's trichrome (for collagen deposition) stains, and multiplex immunofluorescence (IF) images of CK19 (yellow), αSMA (green), PDGFRα (pink) and DAPI (blue) in human PDAC and adjacent pancreas with chronic pancreatitis tissues. Scale bars = 500 μm (H&E and MT) or 50 μm (multiplex IF). **J.** Quantification of αSMA^{+}PDGFRα^{-}DAPI^{+} (left) and PDGFRα^{+}αSMA^{-}DAPI^{+} (right) CK19^{-} cells relative to αSMA^{+} and/or PDGFRα^{+} CK19^{-}DAPI^{+} fibroblasts in human PDAC (n=6) and matched adjacent pancreas with chronic pancreatitis (n=6) tissues, as assessed by multiplex IF analysis. Results show mean ± SEM. *P*, Mann-Whitney test.

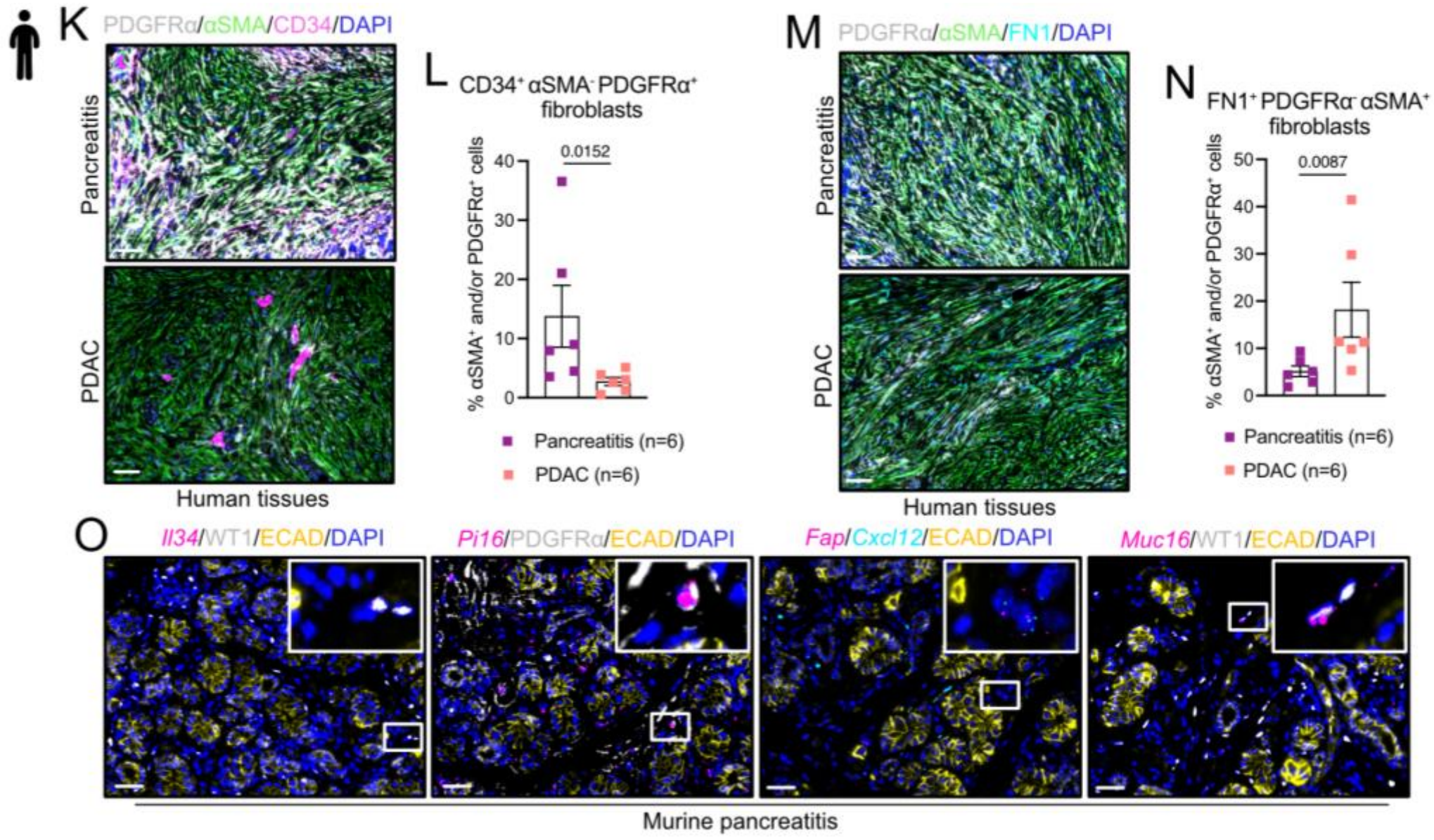

Figure R2.7 [Part 2]. K. Representative multiplex IF images of αSMA (green), PDGFRα (white), CD34 (pink) and DAPI (blue) in human PDAC and adjacent pancreata with chronic pancreatitis tissues. Scale bars = 50 μm. **L.** Quantification of $CD34^+\alpha SMA^-PDGFR\alpha^+DAPI^+CK19^-$ fibroblasts relative to αSMA^+ and/or $PDGFR\alpha^+$ $DAPI^+CK19^-$ fibroblasts in human PDAC (n=6) and adjacent pancreata with chronic pancreatitis (n=6) tissues. Results show mean ± SEM. *P*, Mann-Whitney test. **M.** Representative multiplex IF images of αSMA (green), PDGFRα (white), CD34 (pink) and DAPI (blue) in human PDAC and adjacent pancreata with chronic pancreatitis tissues. Scale bars = 50 μm. **N.** Quantification of $FN1^+PDGFR\alpha^-\alpha SMA^+DAPI^+CK19^-$ fibroblasts relative to αSMA^+ and/or $PDGFR\alpha^+$ $DAPI^+CK19^-$ fibroblasts in human PDAC (n=6) and adjacent pancreata with chronic pancreatitis (n=6) tissues. Results show mean ± SEM. *P*, Mann-Whitney test. **O.** Representative multiplex IF and RNA *in situ* hybridization images of *Il34* (pink) and WT1 (white) to identify the Fibro 1 subset (far left), *Pi16* (pink) and PDGFRα (white) to identify the Fibro 2 subset (left), *Cxcl12* (cyan) and *Fap* (pink) to identify the Fibro 3 subset (right) or *Muc16* (pink) and WT1 (white) to identify the Fibro 4 subset (far right) in murine pancreatitis tissues. DAPI (blue), ECAD (yellow). Scale bars = 20 μm.

Critique R2.9: *The terminology for fibroblast subpopulations should be clarified for readers. Throughout the text, terms like "PSCs," "fibroblasts," "CAFs," and "mesothelial cells" are all used – sometimes interchangeably in co-cultures. It would help to explicitly define each cell source (e.g., PSCs isolated from pancreas, vs. "fibroblasts" from another tissue or an immortalized line) in the Methods or Results. This will prevent confusion, especially when interpreting which cell type is driving a given effect in co-culture.*

Response R2.9: We thank the reviewer for this helpful comment and the opportunity to further clarify fibroblast terminology throughout the manuscript. As also mentioned in **Response 2.3**, in the original manuscript (main text, Methods, and **original Figure 6A**), we described that pancreatic stellate cells (PSCs), fibroblasts, and mesothelial cells used for *in vitro* experiments were all derived from normal pancreata of untreated C57BL/6J mice. We appreciate that this was not sufficiently explicit and have now clarified this point by specifying "untreated normal pancreata" in the main text, Methods, and relevant figure panels.

We also agree that the use of the term "fibroblasts" to refer both to (i) a specific *in vitro* stromal population distinct from PSCs and mesothelial cells, and (ii) *in vivo* fibroblast populations could lead to confusion. To address this, we have revised the terminology throughout the

manuscript and now refer to the *in vitro* fibroblast lines as “mixed-fibroblasts”. This change was implemented to clearly distinguish these cells from both PSCs and mesothelial cells *in vitro*, and from the broader fibroblast populations referred to *in vivo*. We have also clarified that ‘multi-stroma’ comprises of PSCs, mesothelial cells and mixed-fibroblasts.

As also mentioned in **Response 2.3**, we retain the term ‘fibroblasts’ when referring to *in vivo* populations, in line with prevailing terminology in the field. We nevertheless welcome further guidance on nomenclature from the editors and reviewers.

Critique R2.10: *Please provide details on the sample sizes and replicates for key experiments.*

Response R2.10: We have now added explicit information on sample sizes and replicates to all relevant figure legends and panels where this information was previously missing.

In addition, we expanded the number of independent cell and organoid lines in several key experiments. For example, western blot analyses were increased to n=3 biological replicates per group (e.g., **revised ED Figure 5G** and **revised Figures 5H** and **5L**), and additional qPCR and flow-cytometry analyses were performed using expanded cohorts (e.g., **revised ED Figures 7M-Q**).

Finally, all newly added experiments (e.g., qPCR, bulk RNA-sequencing, western blot, multiplex IF, scRNA-seq, snRNA-seq, Visium HD spatial transcriptomics) were conducted with at least n=3 independent tissues, cell lines, organoid lines, line/organoid pairs, mice or other samples (e.g., **new Figures 1A-B, H-L, 2J-L, 3L-M, 8J-O** and **new ED Figures 3O-U, 7G-H, 8K-P, 9E-T, 10C-L**).

Altogether, these experimental additions and textual clarifications have strengthened and refined our conclusions.

Critique R2.11: *Some of the figure presentations could be improved for clarity … labeling, font sizes.*

Response R2.11: We have revised figure layouts across the manuscript to improve clarity, including adjusting labelling, font sizes, and panel organisation where appropriate. In light of the substantial new data added during revision, we have taken care to maximise readability while mataining the completeness of the analyses. We would of course welcome any further specific guidance on figure presentation that could help improve clarity.

Critique R2.12: *Line 153: “FapHigh Fibro 3 subset was most similar to CAFs” should read “……most similar to PDAC CAFs.*

Response R2.12: This has now been amended as suggested, thank you.

Overall, the manuscript presents a wealth of interesting data but also raises several questions that should be addressed. I recommend a major revision prior to publication in Nature Cell Biology. The

central topic of how stromal-epithelial interactions differ between cancer and inflammation is highly relevant and the experimental framework is strong. However, as detailed above, there are significant issues regarding mechanistic depth, data interpretation, and presentation that need improvement. With revisions that address the major comments (particularly providing clearer mechanistic links and acknowledging limitations), I would strongly support the manuscript for NCB as the work has potential impact in the pancreatic cancer and stromal biology field.

We once again thank the Reviewer for their constructive and supportive assessment. We believe that the extensive revisions, which address the key points regarding mechanistic depth, data interpretation, and presentation, have substantially strengthened the manuscript and clarified its significance. We hope the Reviewer will now find the revised study suitable for publication in *Nature Cell Biology*.

Reviewer #3 (Remarks to the Author):

In this manuscript, Li et al investigate the interaction and heterogeneity of fibroblasts communicating with either malignant (PDAC) or inflammatory (chronic pancreatitis, CP) epithelial cells. Overall, this is a timely approach given the increasingly recognized role of fibroblasts contributing significantly to disease phenotypes. As such, the authors present a cross-species cross-disease comparative approach dissecting the (largely transcriptomic) characteristics of epithelial and stromal cells ex vivo and in vitro using organoid-based model systems.

We thank the Reviewer for their careful assessment of our work and for recognising the timeliness of this study. We appreciate their constructive suggestions aimed at further strengthening the manuscript, broadening its scope, and enhancing its impact.

While this is a comprehensive analysis of (mainly) various in vitro models, the usefulness or key novel insights remain a bit elusive. Specific issues are:

Critique R3.1: *This work analyses transcriptomes and respective genes and pathways at multiple levels, yet it is unclear what the identified differences in gene expression really mean. The authors argue for guiding preventative, diagnostic and therapeutic strategies for PDAC by better understanding of epithelial-fibroblast crosstalk. However, beyond the mostly transcriptomic analysis, any functional evidence for key genes/pathways to be candidates for differences between CP and PDAC fibroblast regulation is missing.*

Response R3.1: We thank the Reviewer for highlighting the need to strengthen the mechanistic interpretation of the transcriptomic differences identified in this study. In response, we performed extensive new functional and protein-level analyses that move beyond descriptive transcriptional profiling and establish causal links between epithelial-derived signals and stromal reprogramming in pancreatitis and PDAC.

A) Mechanistic basis of PDAC-dependent inflammatory PSC activation.

In the original manuscript, we observed higher *Il1a* expression in PDAC organoids relative to pancreatitis (P) organoids and hypothesised that this contributes to the enhanced inflammatory phenotype observed in pancreatic stellate cells (PSCs) when cultured with PDAC organoids. We have now directly tested this hypothesis (with data summarised in **Figure R3.1**).

1) We first assessed *Tnf* expression, given its known role in inflammatory PSC activation (e.g., PMID 30366930). *Tnf* levels were not significantly different between PDAC and P organoids in monoculture or PSC co-culture (**Figure R3.1A**), strengthening our rationale to focus on IL-1 signalling rather than TNF-driven effects. These data are included in **new Extended Data (ED) Figure 7C** of the revised manuscript.

2) We found that PDAC organoids secrete significantly higher levels of IL-1α protein compared with P organoids, while secreting comparable levels of TGF-β (**Figure R3.1B**), establishing ligand-level differences between the two epithelial states. These data are included in **new Figure 6F** of the revised manuscript.

3) To causally test whether elevated IL-1α drives the inflammatory PSC phenotype, we used a validated organoid-conditioned media approach to avoid confounding effects of pathway inhibition on epithelial cells. In line with RNA-seq data of organoid/PSC co-cultures, conditioned media from both P and PDAC organoids induced inflammatory markers (*Il1a, Cxcl1, Csf3, Lif, Il6*) in PSCs, with stronger induction by PDAC-derived media (**Figure R3.1C**). Importantly, IL-1α neutralisation markedly reduced this inflammatory response, and more prominently in the PDAC condition. Consistently, PSCs lacking *Il1r1* failed to upregulate inflammatory markers in response to both P and PDAC organoid-conditioned media (**Figure R3.1D**). Together, these data demonstrate that elevated IL-1α secretion by PDAC malignant cells is a key driver of enhanced inflammatory PSC reprogramming relative to P organoids. These data are included in **new ED Figures 7G-H**.

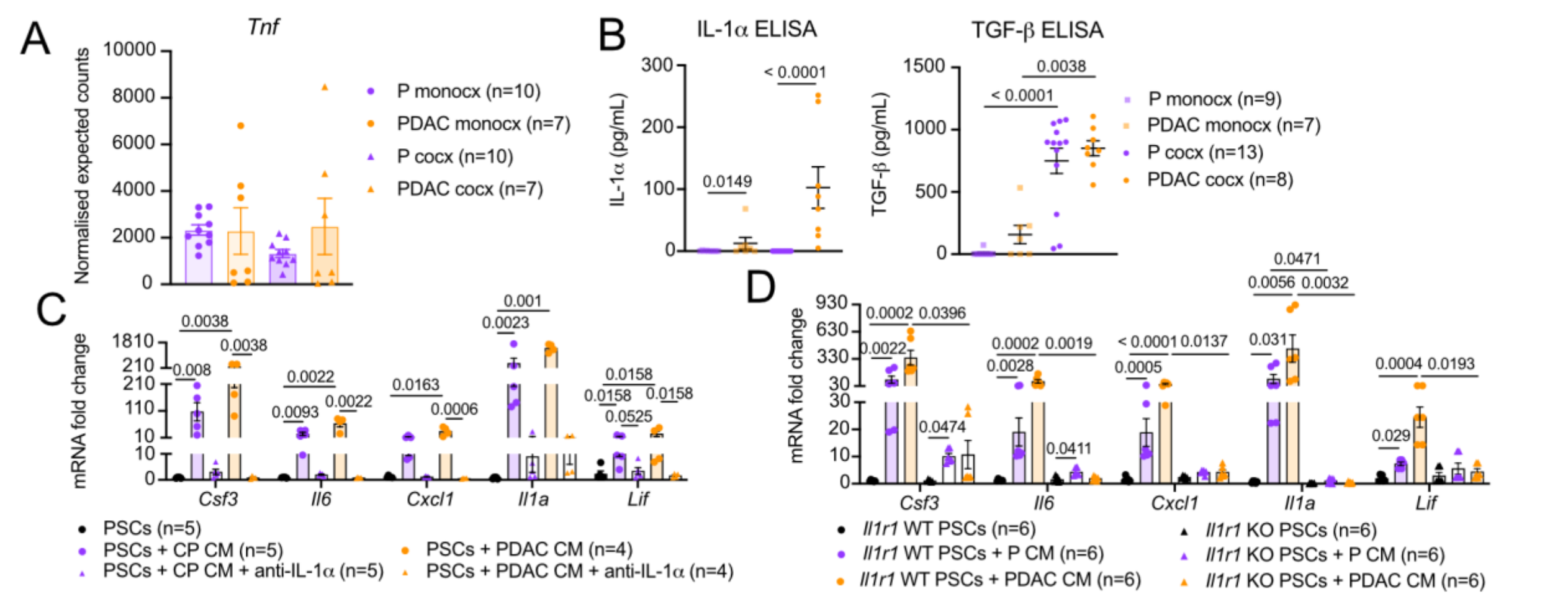

Figure R3.1. A. RNA-seq expression of *Tnf* in P or PDAC monocx and P or PDAC cocx. Results show mean ± SEM. *P adj*, Kruskal-Wallis test**. B.** ELISA of interleukin 1 alpha (IL-1α, left) and transforming growth factor beta (TGF-β, right) from media of P monocx (n=9), PDAC monocx (n=7), P cocx (n=13) and PDAC cocx (n=8). Results show mean ± SEM. *P adj*, Kruskal-Wallis test. **C.** qPCR analysis of PSCs cultured for 4 days in Matrigel with reduced media or with P or PDAC organoid–conditioned media (CM) in the presence of a neutralizing antibody targeting IL1α or an IgG control. Results show mean ± SEM. *P adj*, Kruskal-Wallis test. **D.** qPCR analysis of *Il1r1* wild-type (WT) or *Il1r1* knockout (KO) PSCs cultured for 4 days in Matrigel with reduced media or with P or PDAC organoid–conditioned media. Results show mean ± SEM. *P adj*, Kruskal-Wallis test.

B) Mechanistic basis of differential stromal responses to P or PDAC epithelial-derived cues underlying inflammatory or myofibroblastic phenotypes.

To determine how different stromal populations interpret epithelial-derived signals, we combined ligand–receptor inference with functional perturbation experiments.

1) CellChat analysis of the single-cell RNA-sequencing (scRNA-seq) data from organoid/multi-stroma co-cultures inferred enhanced communication between PDAC malignant cells and PSCs, but reduced interaction with mixed-fibroblasts (corresponding to the fibroblast lines described in the original manuscript, as clarified in response to Reviewer #2) and mesothelial cells, compared with pancreatitis ductal cells (**Figure R3.2A**). This analysis also identified differential signalling for TGF-β, fibronectin (FN1) and IL-1 pathways in PDAC versus pancreatitis co-cultures (**Figures R3.2B-D**). In the IL-1 pathway, *Il1a* was inferred to be the dominant epithelial-to-stromal ligand in PDAC, consistent with our functional experiments. These analyses are included in **new ED Figures 9A-D** of the revised manuscript.

2) To functionally test lineage-specific responsiveness and understand how the presence of mixed-fibroblasts and mesothelial cells in multi-stroma co-cultures helps mirror the *in vivo* enrichment of myofibroblastic features in PDAC, we treated PSCs, mixed-fibroblasts and mesothelial cells with TGF-β or IL-1α, to induce myofibroblastic or inflammatory markers, respectively (based on e.g., PMID 30366930). Mixed-fibroblasts and mesothelial cells preferentially responded to TGF-β, inducing myofibroblastic markers (*Tgfb1*, *Ctgf*, *Ncam1* – encoding for CD56; **Figure R3.2E**). Conversely, PSCs were more responsive to IL-1α treatment, upregulating inflammatory genes (*Il1a*, *Il6*, *Csf3*) more significantly compared to mixed-fibroblasts and mesothelial cells (**Figure R3.2F**). These data are included in **new ED Figures 9E-F** of the revised manuscript.

3) Consistent with these findings, STAT3, an established downstream effector of IL-1 signalling in PDAC CAFs (e.g., PMID 30366930), was preferentially activated in PSCs relative to other stromal cells upon IL1-α treatment and following exposure to PDAC organoid-conditioned media (**Figures R3.2G-H**). In contrast, STAT3 activation in mixed-fibroblasts and mesothelial cells was higher in response to P organoid-conditioned media (**Figure R3.2H**). These data are included in **new ED Figures 9G-H** of the revised manuscript.

Together, these data suggest that mixed-fibroblasts and mesothelial cells are less responsive to PDAC-secreted IL-1α, a major driver of iCAF activation (e.g., PMID 30366930), thereby favouring enrichment of myofibroblastic features within the PDAC stroma. Furthermore, our data indicate that additional factors secreted by pancreatitis ductal cells promote a stronger inflammatory phenotype in mixed-fibroblasts and mesothelial cells, as reflected by enhanced STAT3 activation. This difference may contribute to why pancreatitis multi-stroma, but not PSC-only, co-cultures exhibit a more inflammatory phenotype than PDAC co-cultures. We therefore investigated this further:

4) We focused on leukemia inhibitory factor (LIF), which can mediate STAT3 activation in PDAC iCAFs (e.g., PMID 30366930), as a candidate epithelial-derived driver of inflammatory signalling in pancreatitis. *Lif* expression was higher in P organoids compared to PDAC organoids and was particularly enriched in pancreatitis Ductal 2 cells (**Figures R3.2I-J**). Consistently, LIF treatment robustly activated STAT3 in mixed-fibroblasts and mesothelial cells (**Figure R3.2K**). These data are included in **new ED Figures 9I-K** of the revised manuscript.

Together, these mechanistic data demonstrate that epithelial cells from pancreatitis and PDAC drive distinct stromal programmes through lineage-specific responsiveness to IL-1α, TGF-β, and LIF signalling. These findings further highlight the need to consider stromal heterogeneity in modelling and understanding of these diseased conditions.

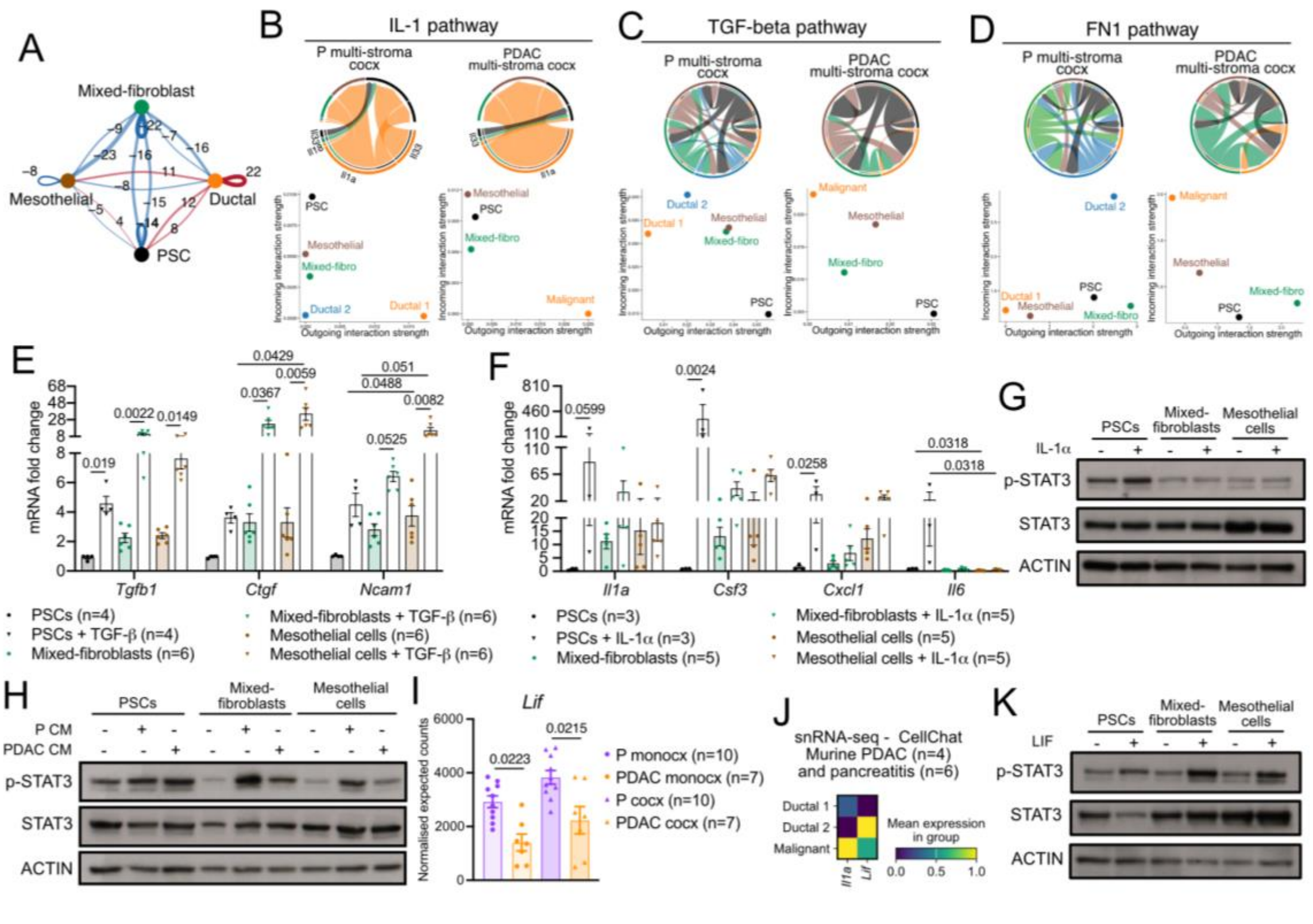

Figure R3.2. A. Cell-cell communication analysis using CellChat showing the difference in connections between cell types in PDAC organoid/multi-stroma co-cultures relative to P organoid/multi-stroma co-cultures, as assessed by scRNA-seq. Blue indicates connection that are lost in PDAC. Red indicates connections that are gained in PDAC. The thickness of the connection reflects the number of interactions. **B-D.** Top panels: selected IL-1 **(B)**, TGF-β **(C)** and fibronectin/FN1 **(D)** pathways in PDAC organoid/multi-stroma co-cultures and P organoid/multi-stroma co-cultures, as assessed by CellChat analysis. Bottom panels: Scatter plots showing the cell types most involved in outgoing and incoming signalling. **E.** qPCR analysis of *Tgfb1, Ctgf* and *Ncam1* in PSCs (n=4), mixed-fibroblasts (n=6) and mesothelial cells (n=6) cultured in Matrigel with reduced media in the presence or absence of 20 ng/mL TGF-β for 4 days. Results show mean ± SEM. *P adj*, Kruskal-Wallis test. **F.** qPCR analysis of *Il1a*, *Csf3, Cxcl1* and *Il6* in PSCs (n=3), mixed-fibroblasts (n=5) and mesothelial cells (n=5) cultured for 4 days in Matrigel with reduced media in the presence or absence of 1-2 ng/mL IL-1α. Results show mean ± SEM. *P adj*, Kruskal-Wallis test. **G.** Western blot of p-STAT3 and STAT3 in PSCs, mixed fibroblasts and mesothelial cells cultured for 4 days in Matrigel with reduced media with or without 1 ng/mL IL-1α. ACTIN, loading control. The samples were obtained and processed simultaneously. **H.** Western blot of p-STAT3 and STAT3 in PSCs, mixed fibroblasts and mesothelial cells cultured in Matrigel with reduced media, P organoid CM or PDAC organoid CM for 4 days. ACTIN, loading control. **I.** RNA-seq expression of *Lif* in P or PDAC monocx and cocx. Results show mean ± SEM. *P adj*, Kruskal-Wallis test. **J.** Heatmap of scaled expression of *Lif* in murine pancreatitis and PDAC ductal clusters. Data are scaled such that the cluster with the lowest average expression = 0 and the highest = 1 for each gene. **K.** Western blot of p-STAT3 and STAT3 in PSCs, mixed fibroblasts and mesothelial cells cultured for 4 days in Matrigel with reduced media with or without 5 ng/mL LIF. ACTIN, loading control.

C) Substantial protein-level validation and additional *in vivo* analyses

Finally, we have substantially expanded both our protein-level validation and *in vivo* analyses to further address the Reviewer's concern regarding functional relevance:

1) **Expanded protein-level validation of disease-specific epithelial markers.** In **new Figures 7K-L**, we performed additional multiplex immunofluorescence (IF) analyses to validate CD44, a marker identified as differentially expressed in PDAC malignant cells relative to pancreatitis ductal cells by single-nuclei RNA-sequencing (snRNA-seq; **Figures R3.3A-B**). In addition, for all previously and newly validated epithelial markers (SOX9, STAT1, MUC5AC, CD44), we extended multiplex IF analyses to two additional murine PDAC models with distinct genetic profiles (KRAS^{G12D} p53-null and SMAD4-deficient KRAS^{G12D} p53-mutant; related to **Response R3.3**). These data are

included in **new ED Figures 8J-P** and further strengthen the robustness and generalisability of our original findings.

2) **Expanded protein-level validation of disease-specific fibroblast markers.** We performed additional multiplex IF analyses to validate markers differentially expressed in PDAC fibroblasts (*Tnc*, *Ncam1* encoding CD56, and *Runx1*) and pancreatitis fibroblasts (*Cd34*), as identified by transcriptomic profiling (**Figures R3.3C-J**; **new Figures 8J-K** and **new ED Figures 9O-T**). In addition, for both previously analyses (*Pdgfra*, *Acta2*, *Fn1*) and newly included fibroblast markers, we extended multiplex IF analyses to the two additional murine PDAC models with distinct genetic profiles, further reinforcing our conclusions (**Figures R3.3K-M**; **new ED Figures 9L-N**).

3) **Protein-level validation of disease-specific fibroblast markers in human PDAC and chronic pancreatitis.** We next performed histological and multiplex IF analyses on human PDAC tissues and matched adjacent pancreas with chronic pancreatitis. While overall collagen deposition differed modestly between conditions, PDAC CAFs displayed higher αSMA levels, whereas pancreatitis fibroblasts showed increased PDGFRα expression (e.g., **Figures R3.3N-O**).

Consistent with our murine findings, human tissues also showed enrichment of CD34$^{+}\alpha$SMA^{-} PDFGRα^{+} fibroblasts in chronic pancreatitis, whereas FN1^{+}PDGFR$\alpha^{-}\alpha$SMA^{+} fibroblasts were more abundant in PDAC tissues (**Figures R3.3P-S**). These data are included in **new Figures 1A-B** and **8L-O**, **new ED Figure 1A**, and **new Supplementary Table 1**.

4) ***In situ* validation of marker co-expression in fibroblast subsets.** To further validate the functional relevance of transcriptomically defined fibroblast populations, we performed multiplex RNA *in situ* hybridization combined with IF in murine pancreatitis tissues. This analysis identified *Il34*^{High}WT1^{High} Fibro 1, *Pi16*^{High}PDGFRα^{High} Fibro 2, *Vegfc*^{High}*Fap*^{High} and/or *Cxcl12*^{High}*Fap*^{High} Fibro 3 and *Muc16*^{High}WT1^{High} Fibro 4 populations (e.g., **Figures R3.3T-U**). These data are included in **new Figures 3C-D** and **new ED Figures 3K** and **4L**. We have also expanded the Discussion to address the potential functional roles of these fibroblast subsets based on their transcriptomic and spatial profiles (lines 973-985). Specifically, we acknowledge that further work will be required to define the precise functions of fibroblast subsets in pancreatitis, while highlighting that our study provides insights into their potential roles by linking distinct fibroblast populations in both murine and human pancreatitis to markers with known functions in diseased and malignant contexts.

5) Finally, we included additional murine and human datasets (Visium HD spatial transcriptomic and snRNA-seq datasets) to expand our *in vivo* analyses (included in Responses below).

Altogether, our new analyses strengthened and expanded our previous conclusion that cross-species and cross-model transcriptomic analyses can be effectively leveraged to identify disease-specific markers for validation in pancreatitis and PDAC tissues.

Finally, we have expanded the Discussion about the potential of identifying stromal-based biomarkers of PDAC, particularly in light of our new data on PDACs with distinct genetic profiles – see also Response R3.3 (lines 917-923). We have also discussed how future targeting of distinct epithelial-stromal signalling identified in our study could guide clinical strategies to both target PDAC, as well as prevent PDAC by targeting chronic pancreatitis in this high-risk patient group (lines 923-928).

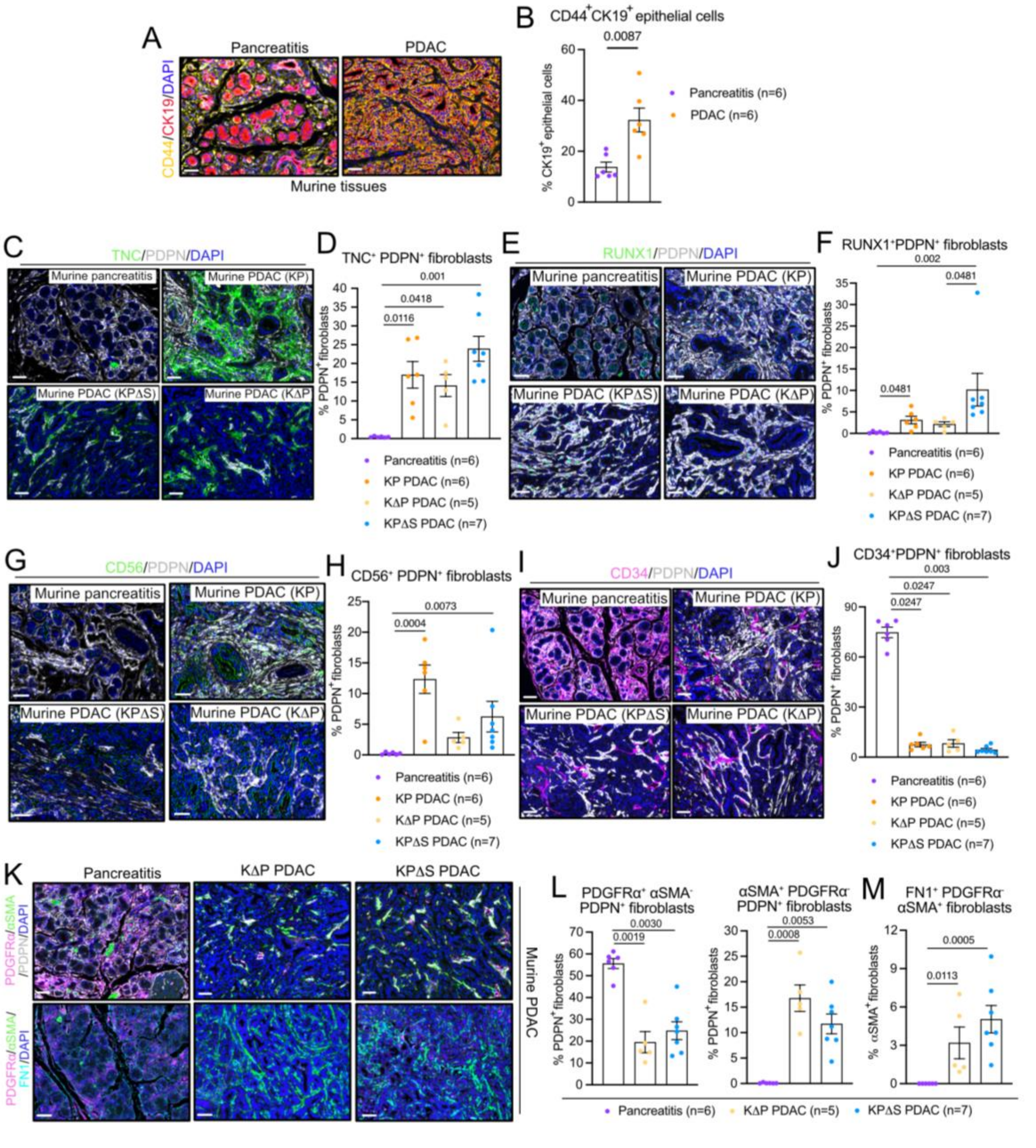

Figure R3.3 [Part 1]. A. Representative multiplex immunofluorescence (IF) images of CD44 (yellow), CK19 (red) and DAPI (blue) in murine pancreatitis and PDAC tissues. Scale bars = 50 μm. **B.** Quantification of cytoplasmic-$CD44^+CK19^+DAPI^+\alpha SMA^-PDGFR\alpha^-$ cells relative to $DAPI^+CK19^+\alpha SMA^-PDGFR\alpha^-$ epithelial cells in murine pancreatitis (n=6) and PDAC (n=6) tissues. Results show mean ± SEM. *P*, Mann-Whitney test. **C-J.** Representative multiplex IF images of PDPN (white) and DAPI (blue), as well as tenascin (TNC, green, **C**) or RUNX1 (green, **E**) or CD56 (green, **G**) or CD34 (pink, **I**) in murine pancreatitis, PDAC tissues from KPC GEMMs (i.e., KP PDAC), as well as PDAC tissues derived from orthotopic transplantation models of $KRAS^{G12D}$, p53-null organoids (i.e., KΔP PDAC) or *Smad4*-deleted, $KRAS^{G12D}$, $p53^{R172H}$ organoids (i.e., KPΔS PDAC). Scale bars = 50 μm. Quantification of $TNC^+PDPN^+DAPI^+CK19^-$ cells **(D)** or nuclear-$RUNX1^+PDPN^+DAPI^+CK19^-$ cells **(F)** or $CD56^+PDPN^+DAPI^+CK19^-$ cells **(H)** or $CD34^+PDPN^+DAPI^+CK19^-$ cells **(J)** relative to $PDPN^+DAPI^+CK19^-$ fibroblasts in murine pancreatitis (n=6), KP PDAC (n=6), KΔP PDAC (n=5) and KPΔS PDAC (n=7) tissues. Results show mean ± SEM. *P adj*, Kruskal-Wallis test. **K.** Representative multiplex IF images of PDPN (white), αSMA (green), PDGFRα (pink) and DAPI (blue) (top panels), or of FN1 (cyan), αSMA (green), PDGFRα (pink) and DAPI (blue) (bottom panels), in murine pancreatitis (left), KΔP PDAC (middle) and KPΔS PDAC (right) tissues. Scale bars = 50 μm. **L.** Quantification of $PDGFR\alpha^+\alpha SMA^-PDPN^+DAPI^+$ (left) and $\alpha SMA^+PDGFR\alpha^-PDPN^+DAPI^+$ (right) $CK19^-$ cells relative to $PDPN^+CK19^-DAPI^+$ fibroblasts in murine pancreatitis (n=6), KΔP PDAC (n=5) and KPΔS PDAC (n=7) tissues, as assessed by multiplex IF analysis. Pancreatitis samples derive from three independent treatment cohorts. Results show mean ± SEM. *P adj*, Kruskal-Wallis test. **M.** Quantification of $FN1^+\alpha SMA^+PDGFR\alpha^-DAPI^+CK19^-$ relative to $\alpha SMA^+CK19^-DAPI^+$ fibroblasts in murine pancreatitis (n=6), KΔP PDAC (n=5) and KPΔS PDAC (n=7) tissues, as assessed by multiplex IF analysis. Pancreatitis samples derive from three independent treatment cohorts. Results show mean ± SEM. *P adj*, Kruskal-Wallis test.

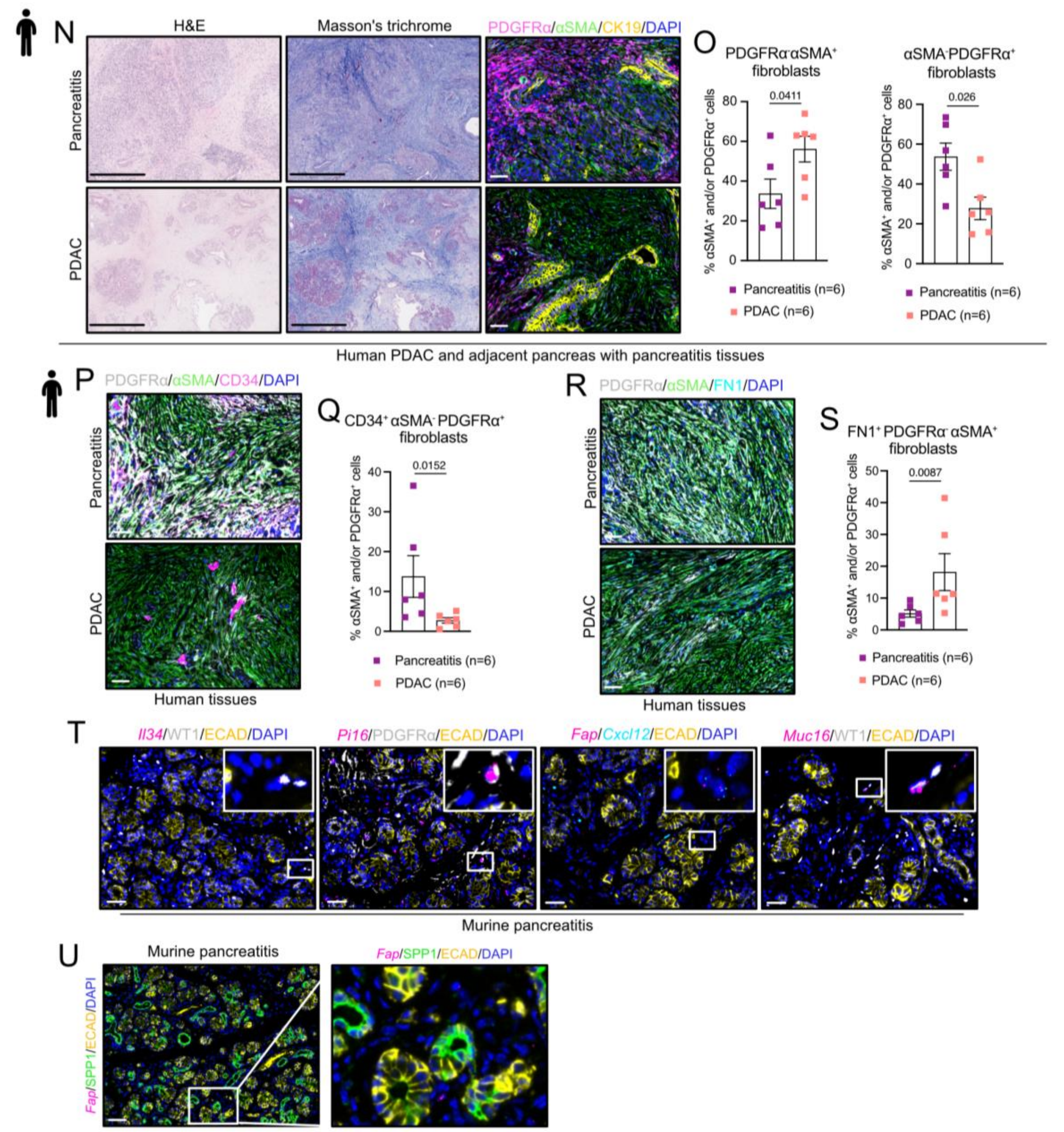

Figure R3.3 [Part 2]. N. Representative Hematoxylin & Eosin (H&E), Masson's trichrome (for collagen deposition) stains, and multiplex immunofluorescence (IF) images of CK19 (yellow), αSMA (green), PDGFRα (pink) and DAPI (blue) in human PDAC and adjacent pancreas with chronic pancreatitis tissues. Scale bars = 500 μm (H&E and MT) or 50 μm (multiplex IF). **O.** Quantification of αSMA$^+$PDGFRα$^-$DAPI$^+$ (left) and PDGFRα$^+$αSMA$^-$DAPI$^+$ (right) CK19$^-$ cells relative to αSMA$^+$ and/or PDGFRα$^+$ CK19$^-$DAPI$^+$ fibroblasts in human PDAC (n=6) and matched adjacent pancreas with chronic pancreatitis (n=6) tissues, as assessed by multiplex IF analysis. Results show mean ± SEM. *P*, Mann-Whitney test. **P.** Representative multiplex IF images of αSMA (green), PDGFRα (white), CD34 (pink) and DAPI (blue) in human PDAC and adjacent pancreata with chronic pancreatitis tissues. Scale bars = 50 μm. **Q.** Quantification of CD34$^+$αSMA$^-$PDGFRα$^+$DAPI$^+$CK19$^-$ fibroblasts relative to αSMA$^+$ and/or PDGFRα$^+$ DAPI$^+$CK19$^-$ fibroblasts in human PDAC (n=6) and adjacent pancreata with chronic pancreatitis (n=6) tissues. Results show mean ± SEM. *P*, Mann-Whitney test. **R.** Representative multiplex IF images of αSMA (green), PDGFRα (white), CD34 (pink) and DAPI (blue) in human PDAC and adjacent pancreata with chronic pancreatitis tissues. Scale bars = 50 μm. **S.** Quantification of FN1$^+$PDGFRα$^-$αSMA$^+$DAPI$^+$CK19$^-$ fibroblasts relative to αSMA$^+$ and/or PDGFRα$^+$ DAPI$^+$CK19$^-$ fibroblasts in human PDAC (n=6) and adjacent pancreata with chronic pancreatitis (n=6) tissues. Results show mean ± SEM. *P*, Mann-Whitney test. **T.** Representative multiplex IF and RNA *in situ* hybridization images of *Il34* (pink) and WT1 (white) to identify the Fibro 1 subset (far left), *Pi16* (pink) and PDGFRα (white) to identify the Fibro 2 subset (left), *Cxcl12* (cyan) and *Fap* (pink) to identify the Fibro 3 subset (right) or *Muc16* (pink) and WT1 (white) to identify the Fibro 4 subset (far right) in murine pancreatitis tissues. DAPI (blue), ECAD (yellow). Scale bars = 20 μm. **U.** Representative multiplex IF and RNA *in situ* hybridization image of DAPI (blue), *Fap* (pink), SPP1 (green) and ECAD (yellow) to identify *Fap*^{High} Fibro 3, SPP1^{Low}ECAD$^+$ Ductal 1 and SPP1^{High}ECAD$^+$ Ductal 2 populations in murine pancreatitis tissues. Right panel: insert. Scale bars = 50 μm. SPP1 was chosen to distinguish between Ductal 1 and Ductal 2 cells based on the snRNA-seq data of murine pancreatitis tissues (Extended Data Figure 3I).

Altogether, these extensive new functional and protein-level analyses directly address the Reviewer's concern by demonstrating that the transcriptomic differences identified between pancreatitis and PDAC epithelial–stromal interactions correspond to specific, causal signalling mechanisms with clear biological interpretation and translational relevance.

Critique R3.2: *Overall, the methodology and quality of data and its representation is well described and performed. In some experiments, the sample size analyzed is considerable small (e.g. n=2 with 2 technical replicates in quite some experimental approaches).*

Response R3.2: We thank the Reviewer for acknowledging the overall quality of the data and its presentation. To address concerns regarding sample sizes in specific experiments, we have clarified replicate information throughout the manuscript and, where appropriate, increased biological replication or incorporated additional orthogonal validation strategies.

Specifically, we expanded the number of independent cell and organoid lines in several key experiments. For example, western blot analyses were increased to n=3 biological replicates per group (e.g., **revised ED Figure 5G** and **revised Figures 5H** and **5L**), and additional qPCR and flow-cytometry analyses were performed using expanded cohorts (e.g., **revised ED Figures 7M-Q**).

Furthermore, we realized our previous use of the term 'technical replicate' caused confusion. We now clarified in the methods and figure legends that all data points in the original manuscript reflected individual measurements. Specifically, measurements of the same cell line at different passages – previously defined as technical replicates- are now accurately described. We now also explicitly state the number of independent lines (and passages, when relevant) for each experiment in the respective figure legends.

Finally, all newly added qPCR, bulk RNA-seq, western blot, multiplex IF, scRNA-seq, snRNA-seq, and Visium HD spatial transcriptomics experiments were conducted with at least n=3 independent tissues, cell lines, organoid lines, organoid/line pairs, mice or other samples (e.g., **new Figures 1A-B**, **1H-L**, **2J-L**, **3L-M**, **8L-O** and **new ED Figures 3O-U**, **7G-H**, **8K-P**, **9E-T**, **10C-L**).

Altogether, these experimental additions and textual clarifications have strengthened and refined our conclusions.

Critique R3.3: *While this is a well-performed cross-species comparison of murine and human epithelial – fibroblast cells in PDAC and CP, a limitation is the considerable small dataset of human samples (n=5 and 4 respectively) as well as the more uniform KPC model, thus challenging generalizability across various PDACs.*

Response R3.3: We thank the Reviewer for raising this important point regarding human sample size and generalisability across pre-clinical models. In response, we performed additional analyses that strengthen and broaden our original conclusions. We also expanded the Discussion to place our study in the context of challenges associated with the availability of human pancreatitis samples.

A) Expanded analysis of human tissues

We have clarified in the revised manuscript that the availability of human chronic pancreatitis samples from non-cancer patients is inherently limited, as surgical intervention in chronic pancreatitis is relatively rare. Although large-scale PDAC datasets are available, direct computational comparison between large PDAC cohorts and limited pancreatitis samples would be inappropriate and potentially misleading. We have also expanded the Discussion to clarify the scarcity of human chronic pancreatitis samples and datasets, thereby strengthening the rationale for leveraging complementary mouse and organoid-derived models (e.g., lines 891-895).

At present, the Dimitrieva et al dataset analysed in the original manuscript represents the only publicly available single-cell and single-nuclei RNA-sequencing dataset comprising more than 3 samples for both human PDAC and chronic pancreatitis and containing both epithelial and stromal populations. Thus, to strengthen the human data component, we integrated two publicly available single-nuclei RNA-sequencing datasets: one from Tosti et al (PMID 33212097) including chronic pancreatitis samples, and one from Chen et al (PMID 39048054) including 1 chronic pancreatitis and 4 PDAC samples. In line with our prior findings, analysis of the integrated dataset confirmed increased proliferation-associated and inflammatory pathways in PDAC malignant cells relative to pancreatitis ductal cells. These analyses also revealed enrichment of inflammatory signalling (e.g., interferon response) in pancreatitis fibroblasts compared with PDAC CAFs (**Figures R3.4A-D**), independently validating and strengthening our original conclusion that PDAC CAFs exhibit a more myofibroblastic phenotype, whereas pancreatitis fibroblasts are more inflammatory. These analyses are included in **new Figures 1H-L**, **new ED Figures 1I-K** and **new Supplementary Table 3**.

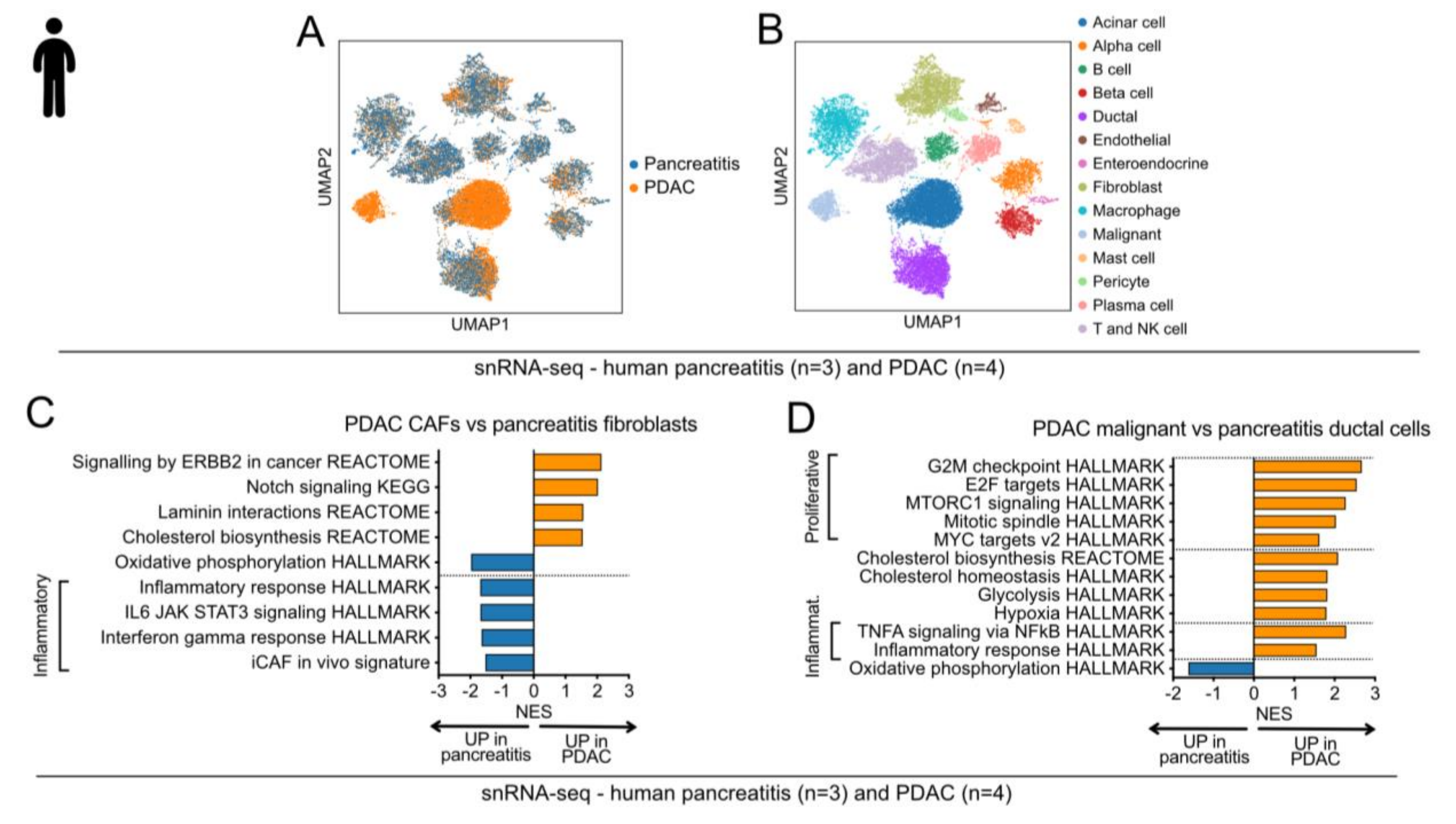

Figure R3.4. A-B. UMAP plots of cell types in the combined single-nuclei RNA-sequencing (snRNA-seq) dataset from human pancreatic ductal adenocarcinoma (PDAC) (n=4) and chronic pancreatitis (n=3) tissues. Different conditions **(A)** and cell types **(B)** are colour coded. **C.** Significantly upregulated pathways identified by GSEA of PDAC CAFs compared to pancreatitis fibroblasts, as assessed by MAST from the snRNA-seq dataset. **D.** Significantly upregulated pathways identified by GSEA of PDAC malignant cells compared to pancreatitis ductal cells, as assessed by MAST. Inflammat, inflammatory; Proliferat., proliferative.

We also sought to include an additional published single-cell RNA-sequencing dataset of human chronic pancreatitis (PMID 34523348); however, this dataset is not publicly available.

Furthermore, we now include multiplex IF analyses on human PDAC tissues and matched adjacent pancreas with chronic pancreatitis (n=6) (e.g., **Figure R3.3**), which independently validate key findings from our murine models.

Together, these efforts expand the available human data supporting our conclusions, while also reflecting the well-recognised challenges of assembling large chronic pancreatitis cohorts. Generation of these datasets remains an important future goal that will require multi-centre collaborative efforts.

B) Expanded analysis across distinct murine PDAC genotypes

We agree with the Reviewer that, while much of the existing literature relies heavily on the KPC model, exclusive reliance on this model is limiting due to its uniform genetic background ($KRAS^{G12D}$ *Trp53*-mutant, i.e. KP). To address this, we expanded our analyses to include two additional PDAC mouse models with distinct genetic profiles: $KRAS^{G12D}$ *Trp53*-null and $KRAS^{G12D}$ *Trp53*-mutant with *Smad4* loss, which we have previously shown differentially shape epithelial and stromal programmes (PMIDs 39841099 and 32633781). Using orthotopic organoid-derived mouse models, we analysed PDAC driven by $KRAS^{G12D}$ p53-null (KΔP PDAC) and SMAD4-deficient $KRAS^{G12D}$ p53-mutant (KPΔS PDAC; **new ED Figures 3R** and **8J**).

1) Extensive multiplex IF analyses demonstrated that epithelial and fibroblast markers distinguishing pancreatitis from PDAC in the KPC model were similarly altered across these additional genetic contexts, though with genotype-specific variation (**Figures R3.4A-F**). Consistent with our previous work (PMIDs: 39841099 and 32633781), these data show that while PDAC malignant cells with distinct genetic profiles share common features that distinguish them from non-malignant inflamed epithelial cells, they also possess distinct biology. These data are included in **new ED Figures 8K-P** of the revised manuscript.

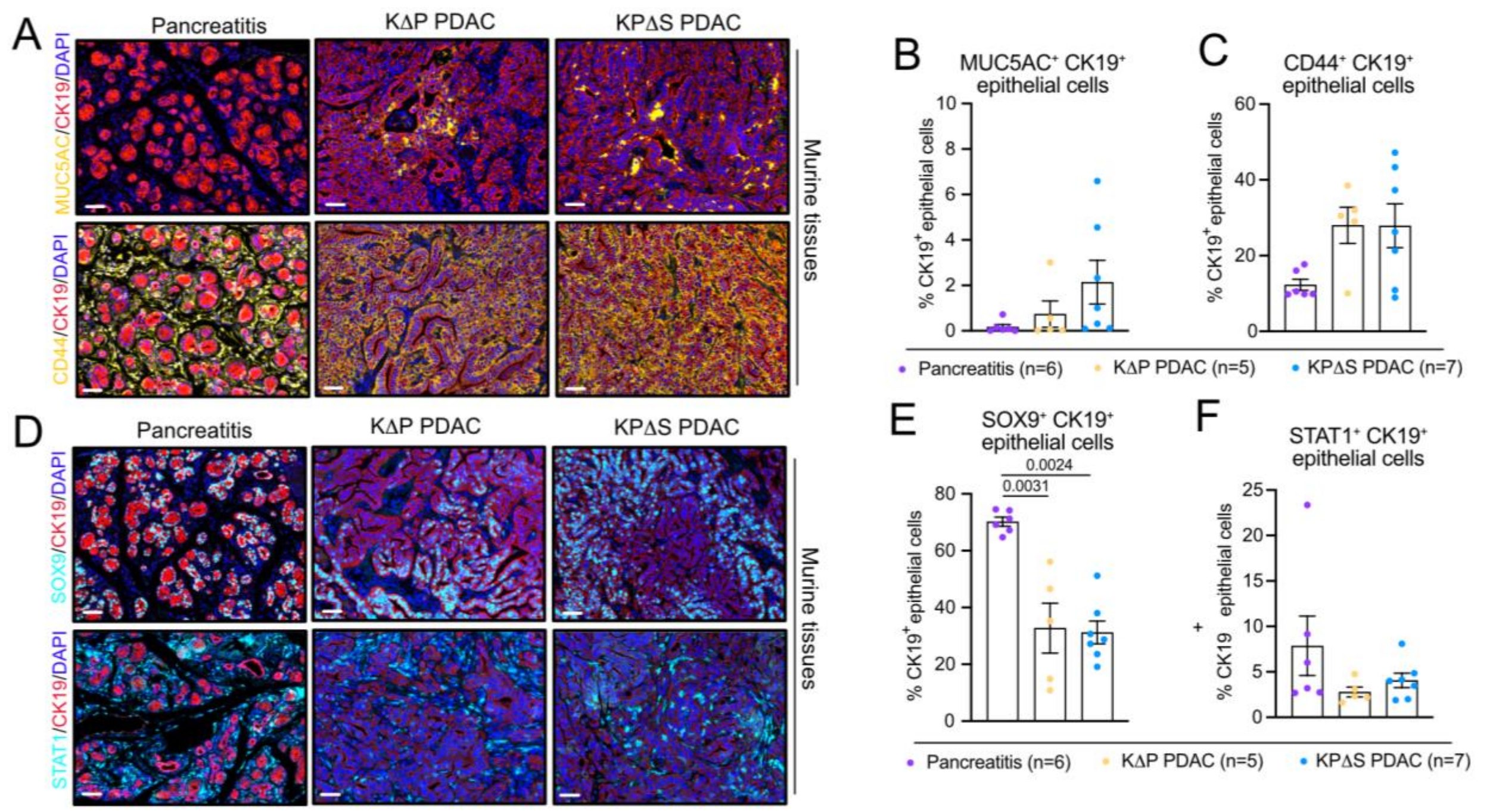

Figure R3.5. A. Representative multiplex IF images of MUC5AC or CD44 (yellow), CK19 (red) and DAPI (nuclear stain, blue) (top panels) in murine pancreatitis, as well as KΔP and KPΔS PDAC tissues. Scale bars = 50 μm. **B-C.** Quantification of $MUC5AC^+CK19^+$ **(B)** and cytoplasmic-$CD44^+CK19^+$ **(C)** $DAPI^+\alpha SMA^-PDGFR\alpha^-$ cells relative to $DAPI^+CK19^+\alpha SMA^-PDGFR\alpha^-$ epithelial cells in murine pancreatitis (n=6), KΔP PDAC (n=5) and KPΔS PDAC (n=7) tissues, as assessed by multiplex IF analysis. Results show mean ± SEM. *P adj*, Kruskal-Wallis test. **D.** Representative multiplex IF images of SOX9 or STAT1 (cyan), CK19 (red) and DAPI (blue) in murine pancreatitis, KΔP and KPΔS PDAC tissues. Scale bars = 50 μm. **E-F.** Quantification of $SOX9^+CK19^+$ **(E)** and $STAT1^+CK19^+$ **(F)** $DAPI^+\alpha SMA^-PDGFR\alpha^-$ cells relative to $DAPI^+CK19^+\alpha SMA^-PDGFR\alpha^-$ epithelial cells in murine pancreatitis (n=6), KΔP PDAC (n=5) and KPΔS PDAC (n=7) tissues, as assessed by multiplex IF analysis. Results show mean ± SEM. *P adj*, Kruskal-Wallis test.

Notably, myofibroblastic markers (e.g., FN1, αSMA, TNC, CD56) were consistently enriched across PDAC models of distinct genetic profiles compared with pancreatitis tissues, and pancreatitis-associated fibroblast markers (e.g., PDGFRα, CD34) remained elevated relative to PDACs irrespective of their genotype (included in **Figure R3.3**; **new Figures 8J-K** and **new ED Figures 9L-T**).

As discussed in Response R3.1, these findings suggest that stromal signatures are more conserved across PDAC genotypes than malignant epithelial programmes and support the potential utility of stromal-based biomarkers for distinguishing malignancy from benign inflammation in high-risk chronic pancreatitis patients. We discuss this point in the revised manuscript (lines 917-923).

2) To further test generalisability at the transcriptomic level, we performed single-nuclei RNA-sequencing of orthotopic organoid-derived KP and KPΔS PDACs (n = 3 per genotype) and integrated these data with our murine pancreatitis single-nuclei RNA-sequencing dataset (**Figures R3.6A-C**). We focused on KPΔS PDAC because we previously showed that SMAD4-deficient PDACs exhibits a shift from myofibroblastic CAFs to inflammatory CAFs (PMID 39841099).

Consistent with our prior observations in KPC GEMMs, CAF signatures from both KP and KPΔS models mapped predominantly to the $\mathit{Fap}^{\mathrm{High}}$ Fibro 3 subset and displayed enrichment of myofibroblastic programmes (e.g., smooth muscle contraction, collagen formation) relative to pancreatitis fibroblasts, regardless of *Smad4* status (**Figures R3.6D-F**). These analyses are included in **new Figures 3L-M, new ED Figures 3P-U** and **new Supplementary Table 7**.

Analysis of the epithelial cell cluster also confirmed key findings originally made in KPC GEMMs, including the inference of a dominant pseudotemporal trajectory connecting pancreatitis Ductal 1 cells to PDAC malignant cells, regardless of *Smad4* status (**Figures R3.6G-I**). These data are included in **new ED Figures 4G-I** of the revised manuscript.

Finally, we expanded the Discussion to explicitly note that future incorporation of additional genetic profiles, as well as ages, will be important to fully capture the nuances of human fibroblast biology and disease heterogeneity (lines 902-905).

Together, these new analyses demonstrate that the key disease-specific epithelial-stromal distinctions identified in this study are robust across available human datasets and genetically distinct murine PDAC models, while highlighting future opportunities for further refinement and expansion.

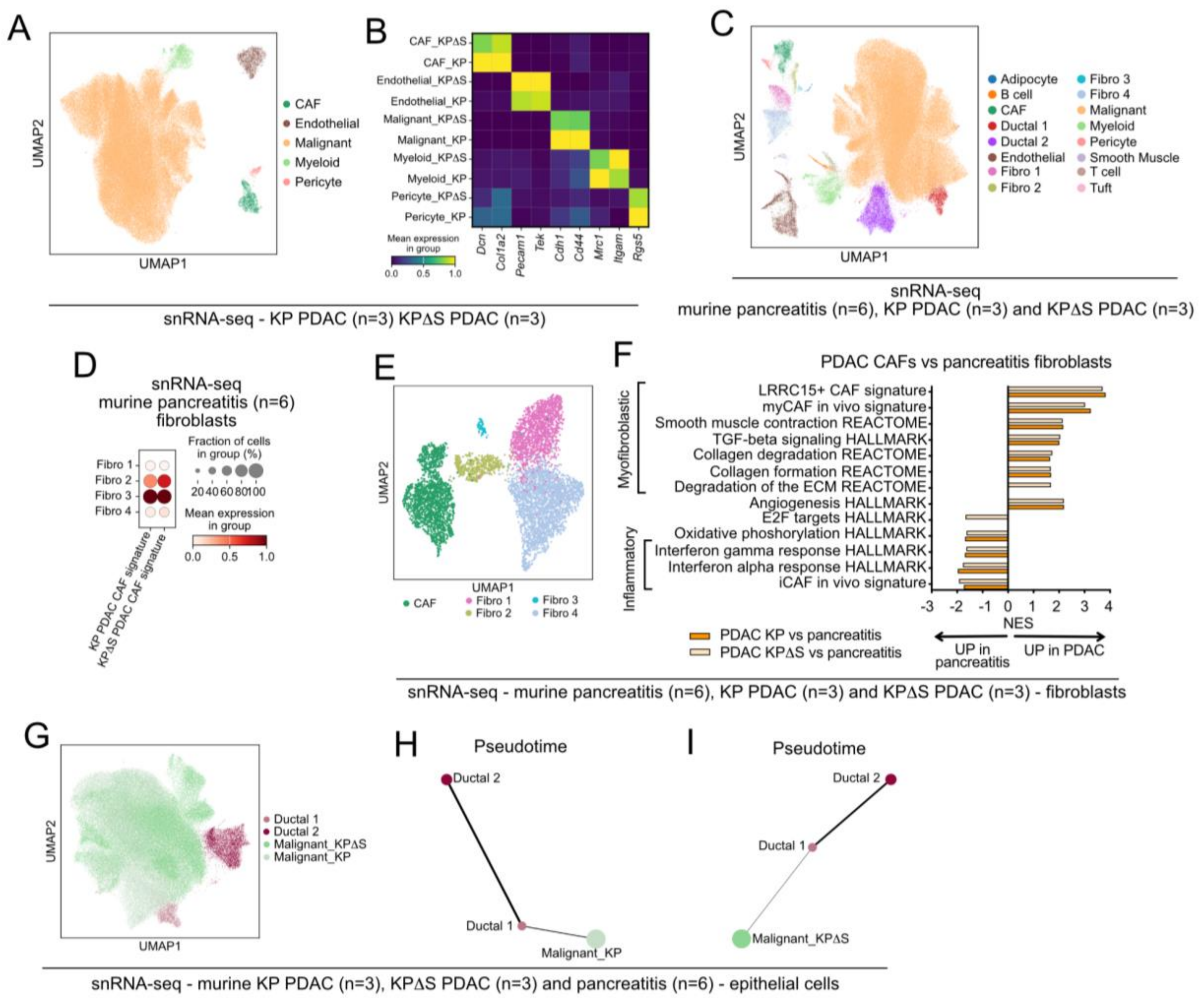

Figure R3.6. A. UMAP plot of cell types identified by snRNA-seq in murine KP and KPΔS PDACs. Different cell types are colour coded. **B.** Heatmap of scaled expression of cell type-specific markers in each cell cluster of murine KP and KPΔS PDAC. Data are scaled such that the cluster with the lowest average expression = 0 and the highest = 1 for each gene. **C.** UMAP plot of cell types identified by snRNA-seq in murine KP and KPΔS PDAC, and pancreatitis. **D.** Dot plot of scaled Aucell score enrichment of KP and KPΔS PDAC CAF signatures in each pancreatitis fibroblast cluster. **E.** UMAP plot of fibroblasts in the combined snRNA-seq dataset from murine KP and KPΔS PDACs and pancreatitis. **F.** Significantly upregulated and downregulated pathways identified by GSEA of KP or KPΔS PDAC CAFs compared to pancreatitis fibroblasts, as assessed by MAST. **G.** UMAP plot of epithelial cells in the combined snRNA-seq dataset from KP PDAC (n=3), KPΔS PDAC (n=3) and pancreatitis (n=6). Different epithelial cell subsets are colour coded. **H-I.** PAGA illustrating the inferred pseudotemporal trajectory among pancreatitis Ductal 1 and Ductal 2 cells and KP **(H)** or KPΔS **(I)** PDAC malignant cells. Nodes (i.e., circles) represent cell subsets, and their size is scaled to the number of cells. Edges (i.e., line) represent the strength of connectivity (i.e., thickness).

Critique R3.4: *Establishment and thorough transcriptomic characterization of organoid co-culture models (especially for CP) is an advancement. Yet the question remains how much such a model reflects the very heterogeneous nature of PDAC (e.g. hypoxia-rich and -poor areas, metastatic lesions vs. primary, etc). While this manuscript can certainly not address many of them in detail, the very descriptive listing of genes and pathways and their overlap (or not) between the used models may fall short of convincingly showing an advance for the model systems in reflecting important disease contributions.*

Response R3.4: We thank the Reviewer for this thoughtful comment. We agree that no individual *in vitro* system can fully recapitulate the spatial and temporal heterogeneity of human PDAC, including hypoxic gradients, metastatic niches, or tumour evolution. Accordingly, we position the multi-stromal organoid co-culture system presented here not as a comprehensive replacement for *in vivo* models, but as a complementary and mechanistically tractable platform.

Importantly, the advance of the current system lies in its ability to model disease-specific epithelial–stromal signalling programmes across inflammatory and malignant contexts, rather than in capturing all dimensions of PDAC heterogeneity simultaneously. In this respect, the organoid/multi-stroma co-cultures enable direct dissection of stromal lineage-dependent responses under controlled conditions that are difficult to isolate *in vivo*. To directly address the Reviewer's concern regarding descriptive transcriptomic overlap, and as detailed in **Response R3.1**, we have substantially expanded the mechanistic and protein-level validation of key transcriptomic findings. These analyses demonstrate that the organoid/multi-stroma co-culture models identify specific signalling axes, including IL-1α, TGF-β, and LIF/STAT3, and lineage-dependent stromal programmes that mirror *in vivo* biology better than simpler models. These functional insights move beyond descriptive gene-set overlap and illustrate how cross-model analyses can reveal disease-relevant mechanisms and candidate therapeutic targets.

We further agree that incorporating additional models and complementary systems will be essential to capture the full breadth of human PDAC heterogeneity. While our laboratory is actively investigating several of these aspects, including primary versus metastatic PDAC, such analyses are beyond the scope of the current manuscript, which is intentionally focused on pancreatic epithelial–stromal interactions in inflammation and malignancy at the level of the primary organ.

Finally, we have expanded the Discussion to outline how future iterations of the multi-stromal organoid platform could incorporate additional disease-relevant features, such as expanded stromal complexity, and increased genetic diversity, to further refine modelling of PDAC heterogeneity (lines 938-946).

Together, our study positions the multi-stromal organoid platform as a valuable complement to *in vivo* models for uncovering disease-relevant epithelial-stromal signalling mechanisms.

Critique R3.5: *While the authors describe four Fibro populations in pancreatitis, CAFs are not subclassified. (e.g. Fig. 2H). While the authors cite recent work on distinct CAF populations beyond myCAF and iCAF (e.g. LRRC15+ cells), no further insights are shared regarding novel useful signatures for specific fibroblast populations/phenotypes which could be of interest, and which could be validated in external data sets as well as functionally regarding their mechanistic role.*

Response R3.5: We thank the Reviewer for this constructive suggestion and the opportunity to expand the scope and interpretability of our original analyses. In the original manuscript, we demonstrated enrichment of myofibroblastic signatures in PDAC CAFs and validated myofibroblastic markers (αSMA, FN1). In the revised manuscript, in addition to the new protein-level and spatial analyses detailed in **Response R3.1**, we have now substantially extended these analyses to provide deeper subclassification of PDAC CAF populations, identify disease-specific fibroblast signatures, and validate their relevance across species, datasets and experimental modalities. Below, we outline key new analyses and insights.

A) Sub-clustering of murine and human PDAC CAFs and human pancreatitis fibroblasts

1) We have now sub-clustered murine PDAC CAFs from our snRNA-seq dataset into established CAF subtypes (myCAFs, iCAFs and apCAFs; **Figures R3.7A-C**), leveraging our previously published snRNA-seq dataset of murine PDACs (PMID 38157863).

In the original manuscript, semi-supervised scANVI mapping indicated that pancreatitis Fibro 3 showed the highest transcriptional similarity to PDAC CAFs (31%; now **Figure 3E**). PDAC CAF sub-clustering refined this analysis and revealed that Fibro 3 aligned most closely with iCAFs (43%), Fibro 2 with myCAFs (42%) and Fibro 1 and Fibro 4 with apCAFs (56% and 82%, respectively; **Figure R3.7D**).

These data have been included in **new ED Figures 3D-F** and **3I** or the revised manuscript.

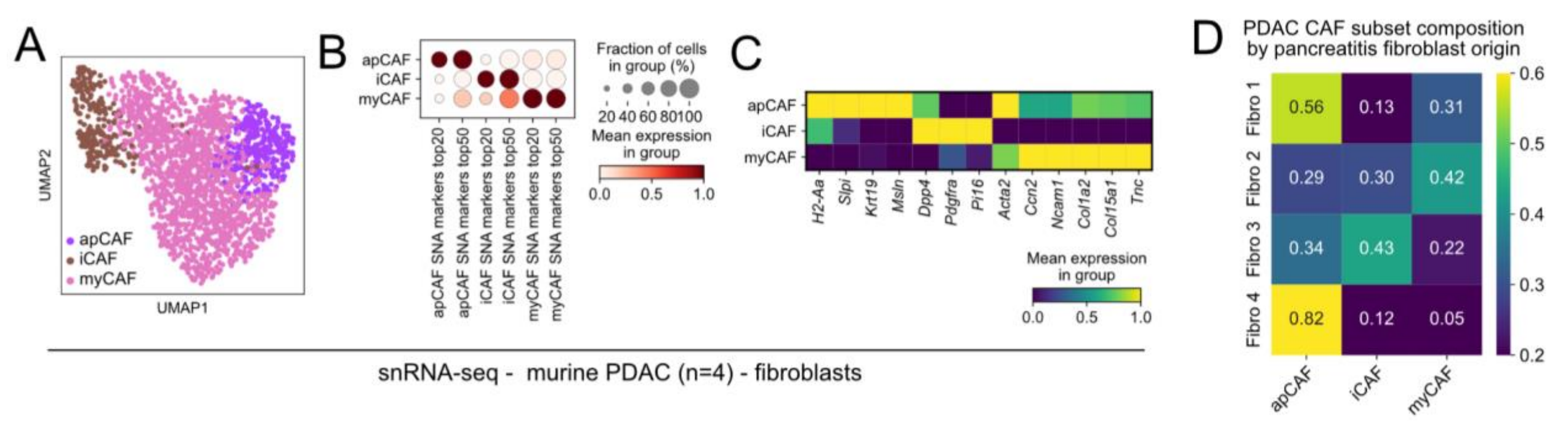

Figure R3.7. A. UMAP plot of fibroblasts from murine PDAC (n=4). Different subsets are colour coded. **B.** Dot plot of scaled Aucell score enrichment of PDAC CAF subtype signatures in each PDAC fibroblast cluster. CAF subtype signatures (i.e., top 20 and top 50 markers) were derived from the snRNA-seq dataset of PDAC tumours from Mucciolo et al. **C.** Heatmap of scaled expression of CAF subtype-specific markers in each fibroblast cluster of murine PDAC. Data are scaled such that the cluster with the lowest average expression = 0 and the highest = 1 for each gene. **D.** Heatmap showing the fractional contribution of *in vivo* pancreatitis fibroblast subclusters to *in vivo* PDAC CAF subsets derived from averaged scANVI soft label probabilities.

2) We also performed sub-clustering analyses of human PDAC CAFs and human pancreatitis fibroblasts from the scRNA-seq dataset included in the original manuscript (**Figures R3.8A-D**). These analyses are presented in **new ED Figures 1E-H**.

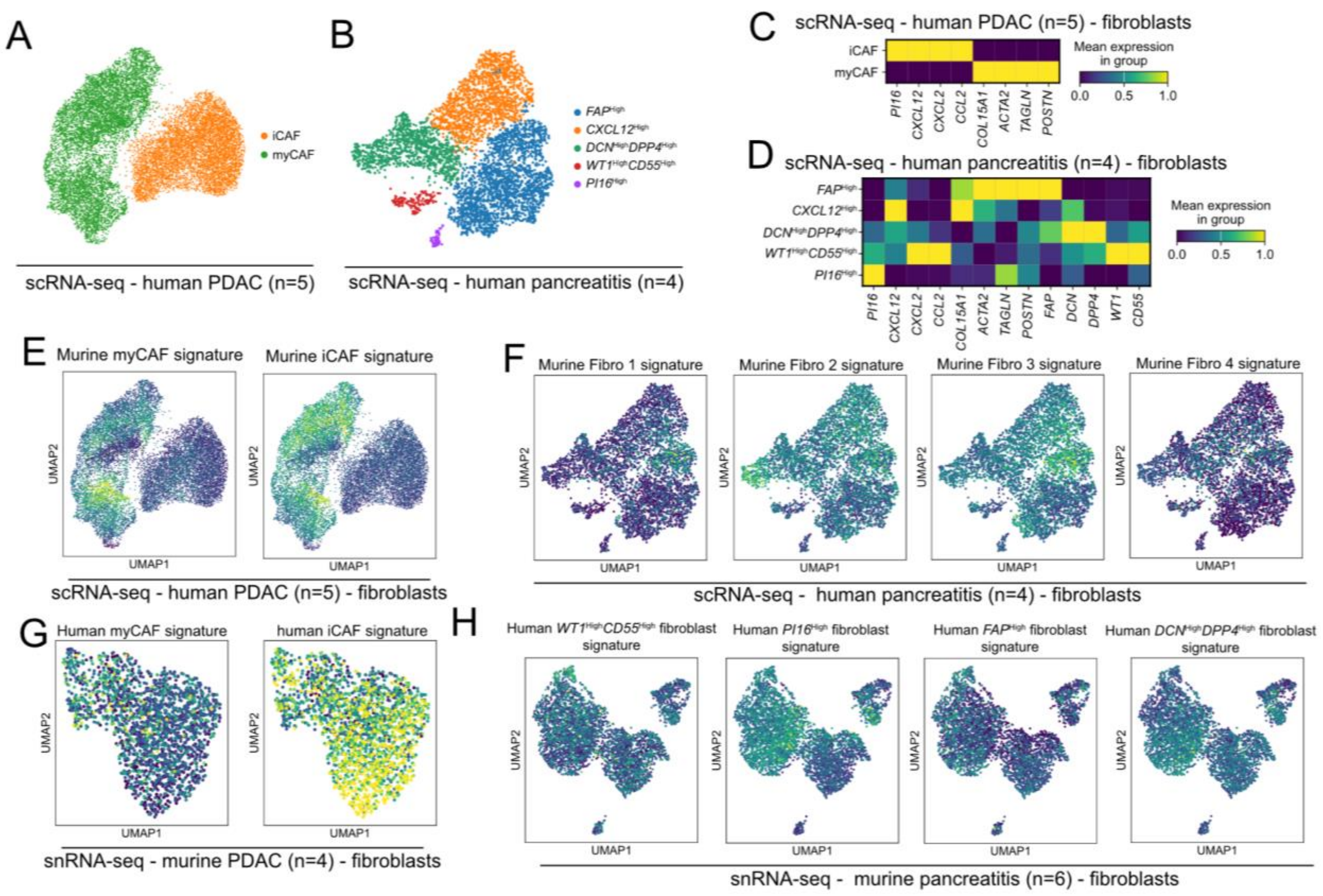

Figure R3.8. A-B. UMAP plots of fibroblasts from human PDAC (**A**, n=5) or human chronic pancreatitis (**B**, n=4). Different subsets are colour coded. **C-D** Heatmaps of scaled expression of CAF subtype-specific markers in each fibroblast cluster of human PDAC **(C)** or of fibroblast subtype-specific markers in each fibroblast cluster of human chronic pancreatitis **(D)**. Data are scaled such that the cluster with the lowest average expression = 0 and the highest = 1 for each gene. **E.** UMAPs of murine PDAC CAF signatures (human orthologs), as identified by snRNA-seq, enriched in human fibroblasts from the PDAC scRNA-seq dataset. **F.** UMAPs of murine pancreatitis fibroblast signatures (human orthologs), as identified by snRNA-seq, enriched in human fibroblasts from the chronic pancreatitis scRNA-seq dataset. **G.** UMAPs of human PDAC CAF signatures (mouse orthologs), as identified by scRNA-seq, enriched in murine fibroblasts from the PDAC snRNA-seq dataset. **H.** UMAPs of human pancreatitis fibroblast signatures (mouse orthologs), as identified by scRNA-seq, enriched in murine fibroblasts from the pancreatitis snRNA-seq dataset. **I.** Significantly enriched pathways (P adj < 0.05) in genes 1) shared by murine and human PDAC CAFs, 2) unique to human relative to murine PDAC CAFs, 3) shared by murine and human pancreatitis fibroblasts, and 4) unique to human relative to murine pancreatitis fibroblasts.

B) Murine and human PDAC and pancreatitis fibroblast signatures for cross-species analyses

We have now defined scRNA-seq and snRNA-seq-derived signatures (top 50 markers per subset relative to other fibroblast subsets) for murine and human PDAC CAFs and pancreatitis fibroblast populations, provided in **revised Supplementary Tables 2** and **9**.

Reciprocal mapping analyses highlights cross-species conserved signatures and disease specificity. For example, mapping murine fibroblast signatures onto human scRNA-seq data, using AUCell scoring, revealed that Fibro 2 and Fibro 3 aligned more closely with human pancreatitis fibroblasts, whereas murine CAF signatures preferentially aligned the human myCAFs (**new ED Figures 4A-B**; representative UMAPs in **Figures R3.8E-F**). Conversely, mapping human fibroblast signatures onto murine snRNA-seq data showed stronger capture of the human iCAF signature in murine PDAC CAFs, while, among human pancreatitis fibroblasts, the *DCN*^{High}*DPP4*^{High} subset was best represented in murine models (**new ED Figures 4C-D;** representative UMAPs in **Figures R3.8G-H**).

C) Validation in external murine datasets

Publicly available single-cell or single-nuclei RNA-sequencing datasets of murine pancreatitis containing fibroblasts from prolonged caerulein treatment are lacking, making our 6-week caerulein-induced pancreatitis snRNA-seq dataset a unique resource. Although no publicly available scRNA-seq or snRNA-seq datasets from prolonged caerulein-induced pancreatitis with fibroblast representation were identified, we analysed an ethanol-dependent, 3-week caerulein pancreatitis scRNA-seq dataset (PMID: 35738281) as the closest available comparator.

Consistent with the reduced disease severity observed in this model (PMID: 35738281), fibroblasts in this dataset were less activated than in our models as they segregated into *Pi16*⁺ and *Col15a1*⁺ progenitor-like fibroblast populations (PMID: 33981032; **Figures R3.9A-C**, for review only). Despite these differences, integration of this pancreatitis dataset with our orthotopic KPC organoid-derived PDAC scRNA-seq data (KP PDAC, n=8 age-matched young C57BL/6J mice; Araos et al *BioRxiv* 2026) confirmed increased *Pdgfra* expression in pancreatitis fibroblasts and elevated *Acta2* expression with enrichment of myofibroblastic pathways in PDAC CAFs (**Figures R3.9D-F**). While these analyses are not included in the revised manuscript due to space constraints and the comparatively limited disease severity and shorter caerulein-treatment of this ethanol-dependent pancreatitis model, we would be pleased to incorporate them should the Reviewer and Editors find this informative.

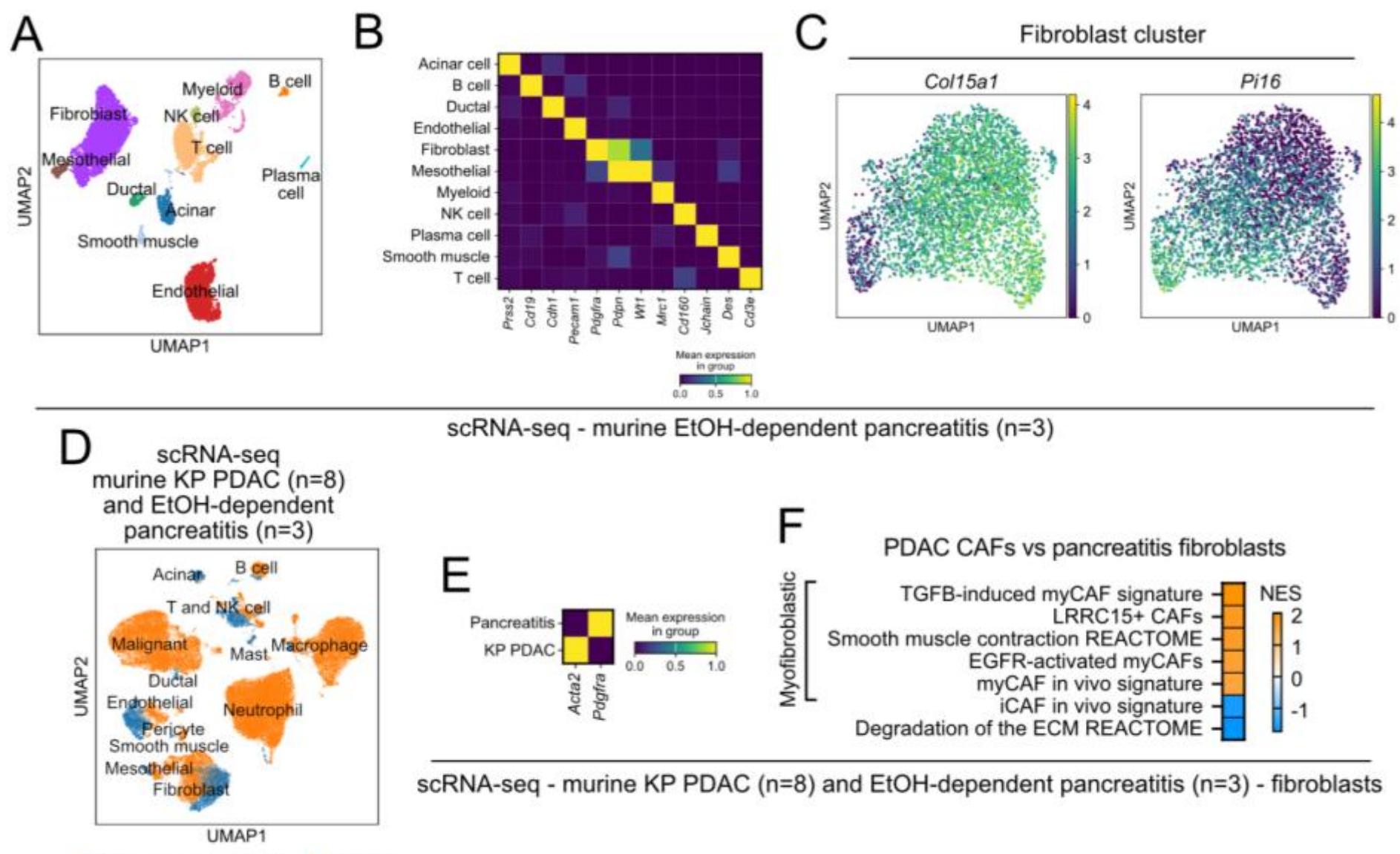

Figure R3.9. A. UMAP plot of cell types identified by scRNA-seq of murine ethanol-dependent pancreatitis. Different cell types are colour coded. **B.** Heatmap of scaled expression of cell type-specific markers in each cell cluster of murine ethanol-dependent pancreatitis. **C.** UMAP plots of *Pi16* and *Col15a1* in fibroblasts from murine ethanol-dependent pancreatitis. **D.** UMAP plot of cell types identified by scRNA-seq of murine ethanol-dependent pancreatitis and KP PDAC. Different conditions are colour coded. **E.** Heatmap of scaled expression of *Acta2*, *Pdgfra* in fibroblasts from murine pancreatitis and PDAC. **F.** Significantly upregulated and downregulated pathways identified by GSEA of KP PDAC CAFs compared to pancreatitis fibroblasts, as assessed by MAST.

D) Functional insights into pancreatitis fibroblast subsets

We have substantially expanded functional interpretation of pancreatitis fibroblast subsets. As outlined in **Response R3.1**, multiplex RNA *in situ* hybridization combined with immunofluorescence validated co-expression of secreted mediators in distinct fibroblast populations, including *Il34*^{High}WT1^{High} Fibro 1, *Pi16*^{High}PDGFRα^{High} Fibro 2, *Vegfc*^{High}*Fap*^{High} and/or

Cxcl12^{High}*Fap*^{High} Fibro 3 and *Muc16*^{High}WT1^{High} Fibro 4 (**new Figures 3C-D** and **new ED Figure 3K** and **4L**). Additionally, our expanded multiplex IF analyses identified several disease-specific fibroblast markers (**revised Figure 8** and **revised ED Figure 9**).

These findings informed expanded Discussion sections (lines 973-985), linking fibroblast subsets to plausible functional roles. Notably, WT1-expressing Fibro 1 and Fibro 4 subsets may relate to recently described PDAC-promoting WT1^{+} CAF lineages (Boschoff et al *Cancer Res* 2026), *Muc16* expression in Fibro 4 also suggests relevance to cancer progression, while *Il34* expression implicates Fibro 1 in fibroblast-monocyte crosstalk. *Fap*^{High} Fibro 3 displays signatures consistent with immunomodulatory and angiogenic functions (PMID 24277834). *Pi16*^{High} Fibro 2 could correspond to *Pi16*$^{+}$ progenitor-like 'universal' fibroblasts described across tissues (PMID 33981032).

Finally, new CellChat and NicheNet analyses of murine pancreatitis and PDAC snRNA-seq datasets (**Figure R3.10**; **revised Figure 3J, new Figure 3K, new ED Figure 3O** and **4J**) further define epithelial-stromal crosstalk and reinforce the conclusion that PDAC CAF signalling is dominated by myofibroblastic programmes (e.g., *Tgfb1*, FN1).

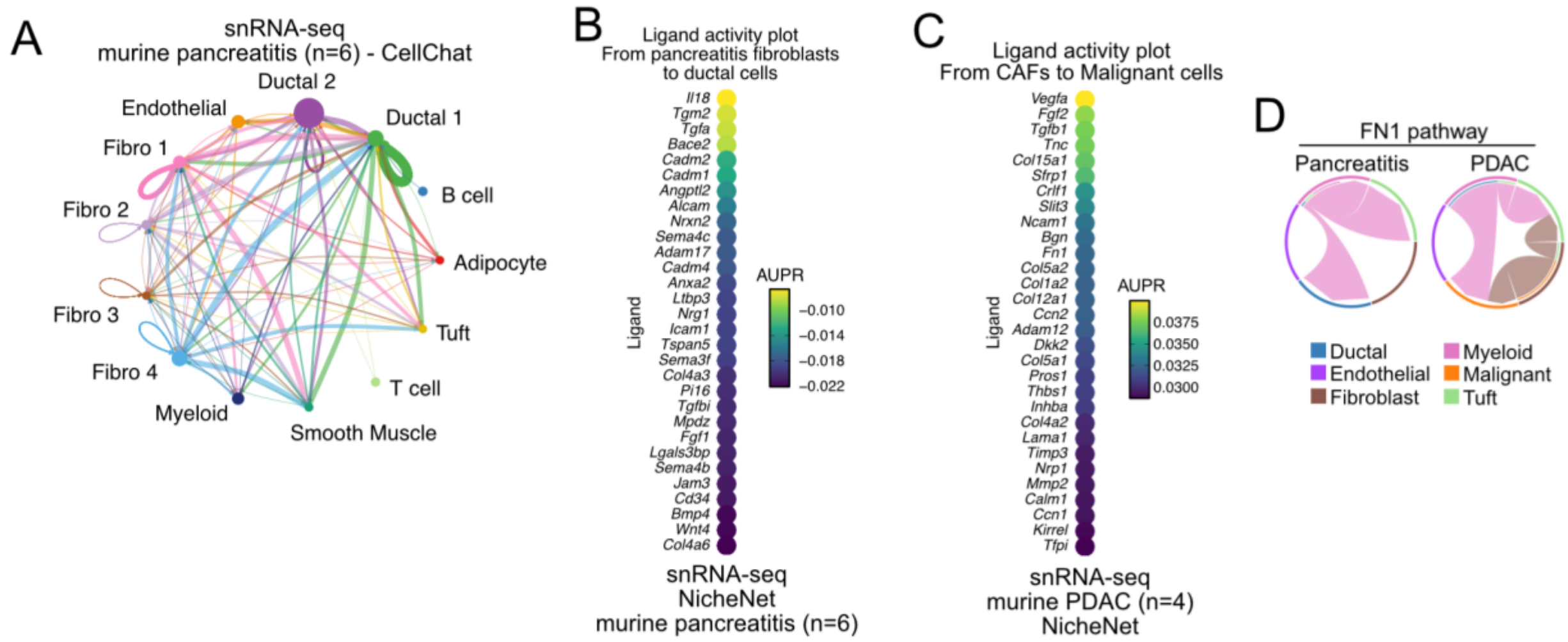

Figure R3.10. A. Visualization of inferred cell-cell communication using CellChat showing the connections between all cell types identified in murine pancreatitis, as assessed by snRNA-seq. The thickness of the connection reflects the number of interactions. **B.** Ligand activity plot shows the top ligands of fibroblasts inferred to regulate target genes in ductal cells in murine pancreatitis, as assessed by NicheNet analysis of snRNA-seq. AUPR, area under the precision-recall curve. **C.** Ligand activity plot shows the top ligands of CAFs inferred to regulate target genes in malignant cells in murine PDAC, as assessed by NicheNet analysis of snRNA-seq. AUPR, area under the precision-recall curve. **D.** Selected fibronectin (FN1) pathway with different connections in murine pancreatitis and PDAC as assessed by CellChat analysis.

Together, these new analyses substantially extend fibroblast subclassification, define cross-species fibroblast signatures, and provide functional insights into disease-specific stromal phenotypes.

Critique R3.6: *How do classical and basal-like PDAC cells respectively interact with (and reprogram?) the different fibroblast populations in the co-culture model?*

Response R3.6: We thank the Reviewer for raising this point. We agree that understanding how classical and basal-like PDAC cells differentially interact with and reprogramme fibroblast populations is interesting. A comprehensive dissection of these interactions would require further dedicated investigation and is beyond the scope of the present study. Nonetheless, to begin addressing this point, we performed additional analyses using our existing datasets. Specifically, we further analysed the scRNA-seq dataset generated from PDAC multi-stromal organoid co-cultures. We mapped established classical/progenitor and basal/squamous PDAC signatures derived from human datasets (PMID 26343385) onto the epithelial compartment of the PDAC organoid multi-stroma co-cultures. This analysis revealed proportions of progenitor and basal epithelial populations comparable to those observed in both *in vivo* murine models and human PDAC specimens (**Figures R3.11A-C**, for review only), allowing us to confidently identify basal-like and progenitor/classical epithelial states within the co-culture system (**Figure R3.11D**). Using CellChat analysis, we then examined ligand-receptor signalling differences between these epithelial subtypes. This revealed distinct signalling preferences: progenitor/classical epithelial cells were inferred to be the predominant source of IL-1-mediated malignant-to-stromal signalling, whereas basal epithelial cells more strongly engaged TGF-β and fibronectin (FN1) pathways (**Figures R3.11E-G**). These findings suggest that classical and basal-like PDAC epithelial cells interact differently with stromal populations of distinct origins and likely contribute to distinct modes of stromal reprogramming. Due to space limitations in the current figures and because these analyses extend beyond the primary focus of the present study, we have not included them in the revised manuscript. However, we would be pleased to incorporate these data should the Reviewer and Editors feel they would be informative.

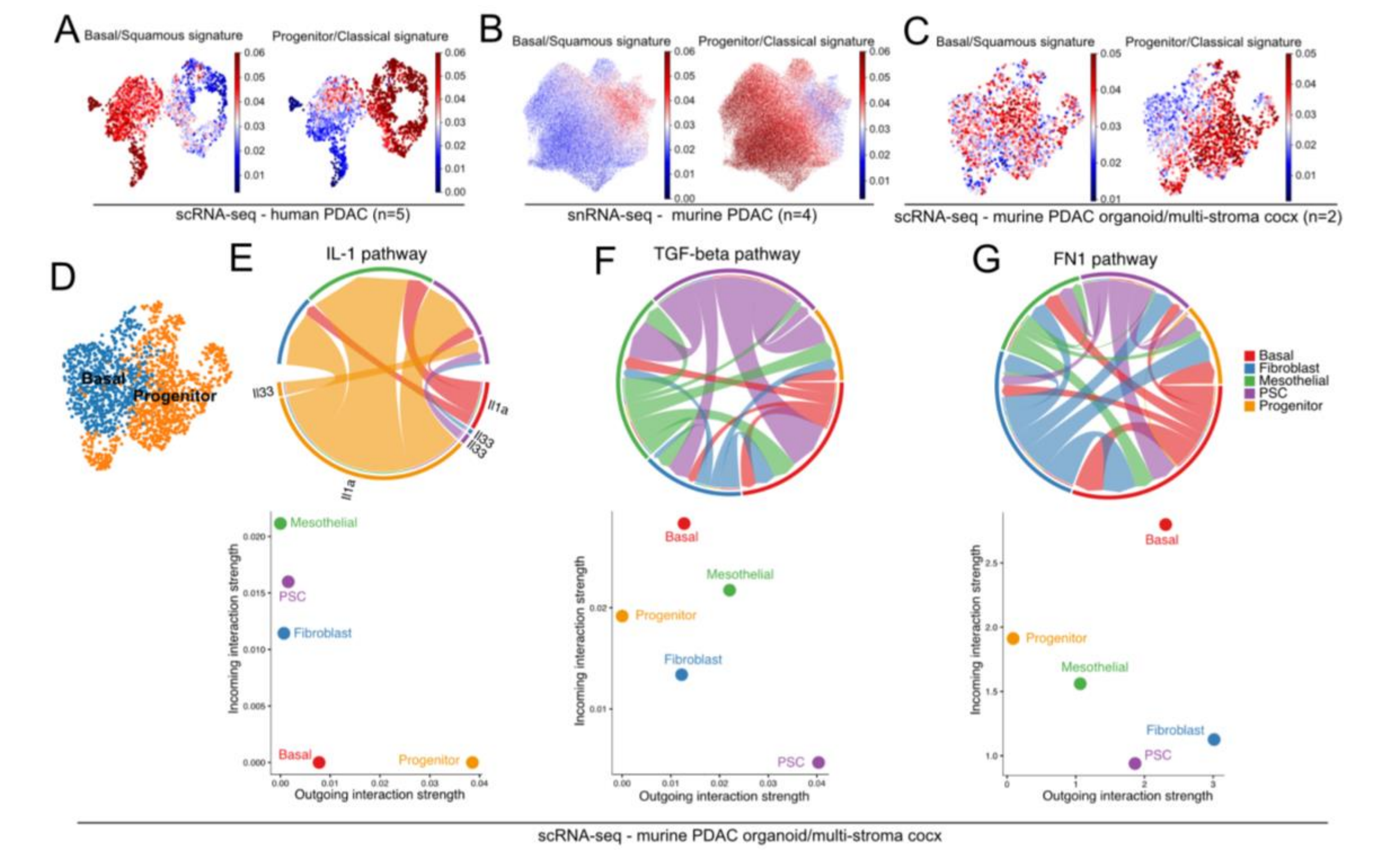

Figure R3.11. A. UMAPs of human basal and classical signatures enriched in epithelial cells from human PDAC scRNA-seq data. **B-D.** UMAPs of human basal and classical signatures (orthologs) enriched in epithelial cells from murine PDAC snRNA-seq data **(B)** or PDAC organoid/multi-stroma co-culture scRNA-seq data **(C-D)**. **E-G.** Top panels: selected IL-1 **(E)**, TGF-β **(F)** and fibronectin/FN1 **(G)** pathways in PDAC organoid/multi-stroma co-cultures, as assessed by CellChat analysis. Bottom panels: Scatter plots showing the cell types most involved in outgoing and incoming signalling.

Critique R3.7: *Do the authors consider transdifferentiation e.g. of epithelial (or mesothelial) cells into distinct fibroblast populations to occur? A robust cell-lineage approach would be of interest to further clarify the extent of such here.*

Response R3.7: We thank the Reviewer for the opportunity to expand our discussion on this important point. Our data indicate that mesothelial cells contribute to Fibro 1 and Fibro 4 populations, as well as to PDAC CAFs (**Figures 8P-S** in the revised manuscript). These findings are consistent with a prior study demonstrating that mesothelial cells represent one origin of PDAC apCAFs using cell-lineage tracing models (PMID 35523176). Moreover, our expanded characterisation of fibroblast subsets provides additional insights into the potential contribution of Fibro 1 and Fibro 4 populations to PDAC apCAFs (**Response R3.5**, **Figure R3.7D**).

To assess whether pancreatitis fibroblast subsets or PDAC CAFs might arise through transdifferentiation from epithelial cells, we further analysed our murine pancreatitis and PDAC snRNA-seq datasets. Pseudotemporal trajectory and marker expression analyses did not support epithelial-to-fibroblast transdifferentiation in either disease context (**Figure R3.12**, for review only). While these analyses suggest that such transdifferentiation is not a dominant mechanism in our models, we agree that definitive resolution of this question will require *in vivo* lineage-tracing approaches. Accordingly, we now explicitly state in the revised manuscript that *in vivo* lineage-tracing experiments will be required to determine the cellular origins of pancreatitis fibroblasts and PDAC CAF subtypes (lines 870-872).

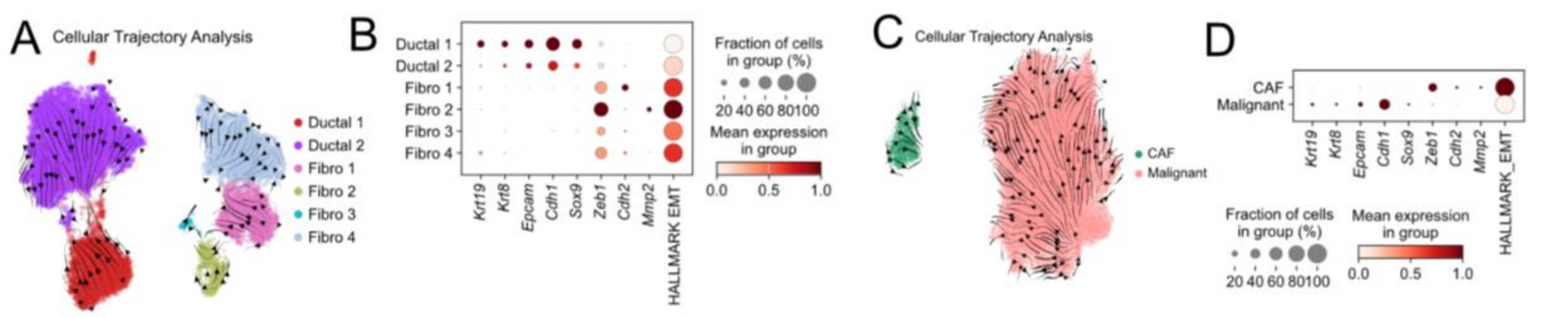

Figure R3.12. A. Pseudotime (Cytotrace) of ductal cell and fibroblast clusters in murine pancreatitis, as assessed by snRNA-seq. Cytotrace tries to find the trajectory from the least to the most differentiated cells. The arrows indicate the directions. **B.** Dot plot of scaled expression of epithelial and epithelial-to-mesenchymal transition (EMT) markers in each ductal cell and fibroblast clusters in murine pancreatitis. The colour intensity represents the expression level, and the size of the dots represents the percentage of expressing cells. **C.** Pseudotime (Cytotrace) of malignant cell and CAF clusters in murine PDAC, as assessed by snRNA-seq. **D.** Dot plot of scaled expression of epithelial and EMT markers in each malignant cell and CAF clusters in murine PDAC. The colour intensity represents the expression level, and the size of the dots represents the percentage of expressing cells.

Response to the Reviewers

Reviewer #1:
Remarks to the Author:
The authors have very thoroughly addressed my comments on the original submission.

Thank you.

Reviewer #2:
Remarks to the Author:
I have reviewed the revised manuscript and the authors' point-by-point response. In my view, the authors have addressed the reviewer concerns satisfactorily. The revision includes substantial new experimental and analytical work, including additional tissue validation, spatial analyses, improved quantification, mechanistic interrogation of epithelial–stromal signaling, sequential co-culture experiments, and a more balanced discussion of model limitations.

I do not see any major outstanding concerns. The remaining caveats are appropriately acknowledged and do not materially weaken the central conclusions. I would support acceptance of the revised manuscript.

Thank you.

Reviewer #3:
Remarks to the Author:
In the revised manuscript and the rebuttal, the authors provide a lot of additional data and analyses to address my and the other reviewers´ issues. This includes a wealth of new data and methodology (e.g. spatial transcriptomics) as well as more mechanistic data. The authors should be gratulated for their important work and provision of the revised and new data. I agree with the authors that the figures R3.9 and 3.11 may not be included in the manuscript. Overall, I support acceptance of this work for publication in NCB.

Thank you.
